# Supplementary material for: Swarm v2: highly-scalable and high-resolution amplicon clustering
Source: PeerJ. 2015 Dec 10;3:e1420. doi: 10.7717/peerj.1420 (PMC4690345; doi:10.7717/peerj.1420)
Supplement: File S1 [file peerj-03-1420-s001.zip › supplementary_file.html]

xml version="1.0" encoding="utf-8"?


Swarm v2: highly-scalable and high-resolution amplicon clustering


# Swarm v2: highly-scalable and high-resolution amplicon clustering

Frédéric Mahé, Torbjørn Rognes, Christopher Quince, Colomban de Vargas, Micah Dunthorn

Supplementary file

## Table of Contents

- 1 Time and space benchmarks (Earth Microbiome Project 16S rRNA) 
  - 1.1 Obtain the datasets
  - 1.2 Dereplicate
  - 1.3 Clustering
  - 1.4 Timings and memory usage
  - 1.5 Timmings and OTUs with/without breaking
- 2 Statistics on mock-communities
- 3 OTU visualizations (BioMarKs 18S rRNA V4 and V9 datasets) 
  - 3.1 Obtain the BioMarKs datasets
  - 3.2 Clustering and Visualization 
    - 3.2.1 Graph plotting script
  - 3.3 Compare swarm and usearch 
    - 3.3.1 BioMarKs 18S rRNA V9 OTU 23 fasta file
    - 3.3.2 BioMarKs 18S rRNA V4 OTU 54 fasta file

## 1 Time and space benchmarks (Earth Microbiome Project 16S rRNA)

### 1.1 Obtain the datasets

Links for the partial dataset (1.2 billion reads)

ftp://thebeast.colorado.edu/pub/QIIME\\_DB\\_Public\\_Studies/study\\_550\\_split\\_library\\_seqs\\_and\\_mapping.zip
ftp://thebeast.colorado.edu/pub/QIIME\\_DB\\_Public\\_Studies/study\\_638\\_split\\_library\\_seqs\\_and\\_mapping.tgz
ftp://thebeast.colorado.edu/pub/QIIME\\_DB\\_Public\\_Studies/study\\_662\\_split\\_library\\_seqs\\_and\\_mapping.zip
ftp://thebeast.colorado.edu/pub/QIIME\\_DB\\_Public\\_Studies/study\\_678\\_split\\_library\\_seqs\\_and\\_mapping.zip
ftp://thebeast.colorado.edu/pub/QIIME\\_DB\\_Public\\_Studies/study\\_722\\_split\\_library\\_seqs\\_and\\_mapping.tgz
ftp://thebeast.colorado.edu/pub/QIIME\\_DB\\_Public\\_Studies/study\\_723\\_split\\_library\\_seqs\\_and\\_mapping.tgz
ftp://thebeast.colorado.edu/pub/QIIME\\_DB\\_Public\\_Studies/study\\_776\\_split\\_library\\_seqs\\_and\\_mapping.tgz
ftp://thebeast.colorado.edu/pub/QIIME\\_DB\\_Public\\_Studies/study\\_805\\_split\\_library\\_seqs\\_and\\_mapping.tgz
ftp://thebeast.colorado.edu/pub/QIIME\\_DB\\_Public\\_Studies/study\\_808\\_split\\_library\\_seqs\\_and\\_mapping.zip
ftp://thebeast.colorado.edu/pub/QIIME\\_DB\\_Public\\_Studies/study\\_809\\_split\\_library\\_seqs\\_and\\_mapping.tgz
ftp://thebeast.colorado.edu/pub/QIIME\\_DB\\_Public\\_Studies/study\\_810\\_split\\_library\\_seqs\\_and\\_mapping.tgz
ftp://thebeast.colorado.edu/pub/QIIME\\_DB\\_Public\\_Studies/study\\_829\\_split\\_library\\_seqs\\_and\\_mapping.tgz
ftp://thebeast.colorado.edu/pub/QIIME\\_DB\\_Public\\_Studies/study\\_864\\_split\\_library\\_seqs\\_and\\_mapping.tgz
ftp://thebeast.colorado.edu/pub/QIIME\\_DB\\_Public\\_Studies/study\\_894\\_split\\_library\\_seqs\\_and\\_mapping.tgz
ftp://thebeast.colorado.edu/pub/QIIME\\_DB\\_Public\\_Studies/study\\_905\\_split\\_library\\_seqs\\_and\\_mapping.tgz
ftp://thebeast.colorado.edu/pub/QIIME\\_DB\\_Public\\_Studies/study\\_925\\_split\\_library\\_seqs\\_and\\_mapping.tgz
ftp://thebeast.colorado.edu/pub/QIIME\\_DB\\_Public\\_Studies/study\\_933\\_split\\_library\\_seqs\\_and\\_mapping.zip
ftp://thebeast.colorado.edu/pub/QIIME\\_DB\\_Public\\_Studies/study\\_940\\_split\\_library\\_seqs\\_and\\_mapping.tgz
ftp://thebeast.colorado.edu/pub/QIIME\\_DB\\_Public\\_Studies/study\\_958\\_split\\_library\\_seqs\\_and\\_mapping.tgz
ftp://thebeast.colorado.edu/pub/QIIME\\_DB\\_Public\\_Studies/study\\_963\\_split\\_library\\_seqs\\_and\\_mapping.tgz
ftp://thebeast.colorado.edu/pub/QIIME\\_DB\\_Public\\_Studies/study\\_990\\_split\\_library\\_seqs\\_and\\_mapping.tgz
ftp://thebeast.colorado.edu/pub/QIIME\\_DB\\_Public\\_Studies/study\\_1001\\_split\\_library\\_seqs\\_and\\_mapping.tgz
ftp://thebeast.colorado.edu/pub/QIIME\\_DB\\_Public\\_Studies/study\\_1030\\_split\\_library\\_seqs\\_and\\_mapping.tgz
ftp://thebeast.colorado.edu/pub/QIIME\\_DB\\_Public\\_Studies/study\\_1031\\_split\\_library\\_seqs\\_and\\_mapping.zip
ftp://thebeast.colorado.edu/pub/QIIME\\_DB\\_Public\\_Studies/study\\_1033\\_split\\_library\\_seqs\\_and\\_mapping.tgz
ftp://thebeast.colorado.edu/pub/QIIME\\_DB\\_Public\\_Studies/study\\_1034\\_split\\_library\\_seqs\\_and\\_mapping.zip
ftp://thebeast.colorado.edu/pub/QIIME\\_DB\\_Public\\_Studies/study\\_1035\\_split\\_library\\_seqs\\_and\\_mapping.zip
ftp://thebeast.colorado.edu/pub/QIIME\\_DB\\_Public\\_Studies/study\\_1036\\_split\\_library\\_seqs\\_and\\_mapping.zip
ftp://thebeast.colorado.edu/pub/QIIME\\_DB\\_Public\\_Studies/study\\_1037\\_split\\_library\\_seqs\\_and\\_mapping.zip
ftp://thebeast.colorado.edu/pub/QIIME\\_DB\\_Public\\_Studies/study\\_1038\\_split\\_library\\_seqs\\_and\\_mapping.zip
ftp://thebeast.colorado.edu/pub/QIIME\\_DB\\_Public\\_Studies/study\\_1039\\_split\\_library\\_seqs\\_and\\_mapping.zip
ftp://thebeast.colorado.edu/pub/QIIME\\_DB\\_Public\\_Studies/study\\_1041\\_split\\_library\\_seqs\\_and\\_mapping.tgz
ftp://thebeast.colorado.edu/pub/QIIME\\_DB\\_Public\\_Studies/study\\_1043\\_split\\_library\\_seqs\\_and\\_mapping.tgz
ftp://thebeast.colorado.edu/pub/QIIME\\_DB\\_Public\\_Studies/study\\_1046\\_split\\_library\\_seqs\\_and\\_mapping.zip
ftp://thebeast.colorado.edu/pub/QIIME\\_DB\\_Public\\_Studies/study\\_1056\\_split\\_library\\_seqs\\_and\\_mapping.tgz
ftp://thebeast.colorado.edu/pub/QIIME\\_DB\\_Public\\_Studies/study\\_1098\\_split\\_library\\_seqs\\_and\\_mapping.tgz
ftp://thebeast.colorado.edu/pub/QIIME\\_DB\\_Public\\_Studies/study\\_1198\\_split\\_library\\_seqs\\_and\\_mapping.tgz
ftp://thebeast.colorado.edu/pub/QIIME\\_DB\\_Public\\_Studies/study\\_1222\\_split\\_library\\_seqs\\_and\\_mapping.zip
ftp://thebeast.colorado.edu/pub/QIIME\\_DB\\_Public\\_Studies/study\\_1235\\_split\\_library\\_seqs\\_and\\_mapping.zip
ftp://thebeast.colorado.edu/pub/QIIME\\_DB\\_Public\\_Studies/study\\_1240\\_split\\_library\\_seqs\\_and\\_mapping.zip
ftp://thebeast.colorado.edu/pub/QIIME\\_DB\\_Public\\_Studies/study\\_1242\\_split\\_library\\_seqs\\_and\\_mapping.zip
ftp://thebeast.colorado.edu/pub/QIIME\\_DB\\_Public\\_Studies/study\\_1288\\_split\\_library\\_seqs\\_and\\_mapping.zip
ftp://thebeast.colorado.edu/pub/QIIME\\_DB\\_Public\\_Studies/study\\_1289\\_split\\_library\\_seqs\\_and\\_mapping.zip
ftp://thebeast.colorado.edu/pub/QIIME\\_DB\\_Public\\_Studies/study\\_1453\\_split\\_library\\_seqs\\_and\\_mapping.tgz
ftp://thebeast.colorado.edu/pub/QIIME\\_DB\\_Public\\_Studies/study\\_1526\\_split\\_library\\_seqs\\_and\\_mapping.tgz
ftp://thebeast.colorado.edu/pub/QIIME\\_DB\\_Public\\_Studies/study\\_1578\\_split\\_library\\_seqs\\_and\\_mapping.tgz
ftp://thebeast.colorado.edu/pub/QIIME\\_DB\\_Public\\_Studies/study\\_1579\\_split\\_library\\_seqs\\_and\\_mapping.tgz
ftp://thebeast.colorado.edu/pub/QIIME\\_DB\\_Public\\_Studies/study\\_1580\\_split\\_library\\_seqs\\_and\\_mapping.tgz
ftp://thebeast.colorado.edu/pub/QIIME\\_DB\\_Public\\_Studies/study\\_1621\\_split\\_library\\_seqs\\_and\\_mapping.tgz
ftp://thebeast.colorado.edu/pub/QIIME\\_DB\\_Public\\_Studies/study\\_1622\\_split\\_library\\_seqs\\_and\\_mapping.tgz
ftp://thebeast.colorado.edu/pub/QIIME\\_DB\\_Public\\_Studies/study\\_1627\\_split\\_library\\_seqs\\_and\\_mapping.tgz
ftp://thebeast.colorado.edu/pub/QIIME\\_DB\\_Public\\_Studies/study\\_1632\\_split\\_library\\_seqs\\_and\\_mapping.tgz
ftp://thebeast.colorado.edu/pub/QIIME\\_DB\\_Public\\_Studies/study\\_1642\\_split\\_library\\_seqs\\_and\\_mapping.tgz
ftp://thebeast.colorado.edu/pub/QIIME\\_DB\\_Public\\_Studies/study\\_1673\\_split\\_library\\_seqs\\_and\\_mapping.tgz
ftp://thebeast.colorado.edu/pub/QIIME\\_DB\\_Public\\_Studies/study\\_1692\\_split\\_library\\_seqs\\_and\\_mapping.tgz
ftp://thebeast.colorado.edu/pub/QIIME\\_DB\\_Public\\_Studies/study\\_1694\\_split\\_library\\_seqs\\_and\\_mapping.tgz
ftp://thebeast.colorado.edu/pub/QIIME\\_DB\\_Public\\_Studies/study\\_1702\\_split\\_library\\_seqs\\_and\\_mapping.tgz
ftp://thebeast.colorado.edu/pub/QIIME\\_DB\\_Public\\_Studies/study\\_1734\\_split\\_library\\_seqs\\_and\\_mapping.tgz
ftp://thebeast.colorado.edu/pub/QIIME\\_DB\\_Public\\_Studies/study\\_1747\\_split\\_library\\_seqs\\_and\\_mapping.tgz
ftp://thebeast.colorado.edu/pub/QIIME\\_DB\\_Public\\_Studies/study\\_1748\\_split\\_library\\_seqs\\_and\\_mapping.tgz

Additional links for the full dataset (2.3 billion reads)

ftp://thebeast.colorado.edu/pub/QIIME\\_DB\\_Public\\_Studies/study\\_550\\_split\\_library\\_seqs\\_and\\_mapping.tgz
ftp://thebeast.colorado.edu/pub/QIIME\\_DB\\_Public\\_Studies/study\\_632\\_split\\_library\\_seqs\\_and\\_mapping.tgz
ftp://thebeast.colorado.edu/pub/QIIME\\_DB\\_Public\\_Studies/study\\_638\\_split\\_library\\_seqs\\_and\\_mapping.tgz
ftp://thebeast.colorado.edu/pub/QIIME\\_DB\\_Public\\_Studies/study\\_659\\_split\\_library\\_seqs\\_and\\_mapping.tgz
ftp://thebeast.colorado.edu/pub/QIIME\\_DB\\_Public\\_Studies/study\\_662\\_split\\_library\\_seqs\\_and\\_mapping.tgz
ftp://thebeast.colorado.edu/pub/QIIME\\_DB\\_Public\\_Studies/study\\_678\\_split\\_library\\_seqs\\_and\\_mapping.tgz
ftp://thebeast.colorado.edu/pub/QIIME\\_DB\\_Public\\_Studies/study\\_713\\_split\\_library\\_seqs\\_and\\_mapping.tgz
ftp://thebeast.colorado.edu/pub/QIIME\\_DB\\_Public\\_Studies/study\\_722\\_split\\_library\\_seqs\\_and\\_mapping.tgz
ftp://thebeast.colorado.edu/pub/QIIME\\_DB\\_Public\\_Studies/study\\_723\\_split\\_library\\_seqs\\_and\\_mapping.tgz
ftp://thebeast.colorado.edu/pub/QIIME\\_DB\\_Public\\_Studies/study\\_755\\_split\\_library\\_seqs\\_and\\_mapping.tgz
ftp://thebeast.colorado.edu/pub/QIIME\\_DB\\_Public\\_Studies/study\\_776\\_split\\_library\\_seqs\\_and\\_mapping.tgz
ftp://thebeast.colorado.edu/pub/QIIME\\_DB\\_Public\\_Studies/study\\_804\\_split\\_library\\_seqs\\_and\\_mapping.tgz
ftp://thebeast.colorado.edu/pub/QIIME\\_DB\\_Public\\_Studies/study\\_805\\_split\\_library\\_seqs\\_and\\_mapping.tgz
ftp://thebeast.colorado.edu/pub/QIIME\\_DB\\_Public\\_Studies/study\\_807\\_split\\_library\\_seqs\\_and\\_mapping.tgz
ftp://thebeast.colorado.edu/pub/QIIME\\_DB\\_Public\\_Studies/study\\_808\\_split\\_library\\_seqs\\_and\\_mapping.tgz
ftp://thebeast.colorado.edu/pub/QIIME\\_DB\\_Public\\_Studies/study\\_809\\_split\\_library\\_seqs\\_and\\_mapping.tgz
ftp://thebeast.colorado.edu/pub/QIIME\\_DB\\_Public\\_Studies/study\\_810\\_split\\_library\\_seqs\\_and\\_mapping.tgz
ftp://thebeast.colorado.edu/pub/QIIME\\_DB\\_Public\\_Studies/study\\_829\\_split\\_library\\_seqs\\_and\\_mapping.tgz
ftp://thebeast.colorado.edu/pub/QIIME\\_DB\\_Public\\_Studies/study\\_846\\_split\\_library\\_seqs\\_and\\_mapping.tgz
ftp://thebeast.colorado.edu/pub/QIIME\\_DB\\_Public\\_Studies/study\\_861\\_split\\_library\\_seqs\\_and\\_mapping.tgz
ftp://thebeast.colorado.edu/pub/QIIME\\_DB\\_Public\\_Studies/study\\_864\\_split\\_library\\_seqs\\_and\\_mapping.tgz
ftp://thebeast.colorado.edu/pub/QIIME\\_DB\\_Public\\_Studies/study\\_889\\_split\\_library\\_seqs\\_and\\_mapping.tgz
ftp://thebeast.colorado.edu/pub/QIIME\\_DB\\_Public\\_Studies/study\\_894\\_split\\_library\\_seqs\\_and\\_mapping.tgz
ftp://thebeast.colorado.edu/pub/QIIME\\_DB\\_Public\\_Studies/study\\_905\\_split\\_library\\_seqs\\_and\\_mapping.tgz
ftp://thebeast.colorado.edu/pub/QIIME\\_DB\\_Public\\_Studies/study\\_910\\_split\\_library\\_seqs\\_and\\_mapping.tgz
ftp://thebeast.colorado.edu/pub/QIIME\\_DB\\_Public\\_Studies/study\\_925\\_split\\_library\\_seqs\\_and\\_mapping.tgz
ftp://thebeast.colorado.edu/pub/QIIME\\_DB\\_Public\\_Studies/study\\_933\\_split\\_library\\_seqs\\_and\\_mapping.tgz
ftp://thebeast.colorado.edu/pub/QIIME\\_DB\\_Public\\_Studies/study\\_940\\_split\\_library\\_seqs\\_and\\_mapping.tgz
ftp://thebeast.colorado.edu/pub/QIIME\\_DB\\_Public\\_Studies/study\\_945\\_split\\_library\\_seqs\\_and\\_mapping.tgz
ftp://thebeast.colorado.edu/pub/QIIME\\_DB\\_Public\\_Studies/study\\_958\\_split\\_library\\_seqs\\_and\\_mapping.tgz
ftp://thebeast.colorado.edu/pub/QIIME\\_DB\\_Public\\_Studies/study\\_963\\_split\\_library\\_seqs\\_and\\_mapping.tgz
ftp://thebeast.colorado.edu/pub/QIIME\\_DB\\_Public\\_Studies/study\\_990\\_split\\_library\\_seqs\\_and\\_mapping.tgz
ftp://thebeast.colorado.edu/pub/QIIME\\_DB\\_Public\\_Studies/study\\_1001\\_split\\_library\\_seqs\\_and\\_mapping.tgz
ftp://thebeast.colorado.edu/pub/QIIME\\_DB\\_Public\\_Studies/study\\_1030\\_split\\_library\\_seqs\\_and\\_mapping.tgz
ftp://thebeast.colorado.edu/pub/QIIME\\_DB\\_Public\\_Studies/study\\_1031\\_split\\_library\\_seqs\\_and\\_mapping.tgz
ftp://thebeast.colorado.edu/pub/QIIME\\_DB\\_Public\\_Studies/study\\_1033\\_split\\_library\\_seqs\\_and\\_mapping.tgz
ftp://thebeast.colorado.edu/pub/QIIME\\_DB\\_Public\\_Studies/study\\_1034\\_split\\_library\\_seqs\\_and\\_mapping.tgz
ftp://thebeast.colorado.edu/pub/QIIME\\_DB\\_Public\\_Studies/study\\_1035\\_split\\_library\\_seqs\\_and\\_mapping.tgz
ftp://thebeast.colorado.edu/pub/QIIME\\_DB\\_Public\\_Studies/study\\_1036\\_split\\_library\\_seqs\\_and\\_mapping.tgz
ftp://thebeast.colorado.edu/pub/QIIME\\_DB\\_Public\\_Studies/study\\_1037\\_split\\_library\\_seqs\\_and\\_mapping.tgz
ftp://thebeast.colorado.edu/pub/QIIME\\_DB\\_Public\\_Studies/study\\_1038\\_split\\_library\\_seqs\\_and\\_mapping.tgz
ftp://thebeast.colorado.edu/pub/QIIME\\_DB\\_Public\\_Studies/study\\_1039\\_split\\_library\\_seqs\\_and\\_mapping.tgz
ftp://thebeast.colorado.edu/pub/QIIME\\_DB\\_Public\\_Studies/study\\_1041\\_split\\_library\\_seqs\\_and\\_mapping.tgz
ftp://thebeast.colorado.edu/pub/QIIME\\_DB\\_Public\\_Studies/study\\_1043\\_split\\_library\\_seqs\\_and\\_mapping.tgz
ftp://thebeast.colorado.edu/pub/QIIME\\_DB\\_Public\\_Studies/study\\_1056\\_split\\_library\\_seqs\\_and\\_mapping.tgz
ftp://thebeast.colorado.edu/pub/QIIME\\_DB\\_Public\\_Studies/study\\_1064\\_split\\_library\\_seqs\\_and\\_mapping.tgz
ftp://thebeast.colorado.edu/pub/QIIME\\_DB\\_Public\\_Studies/study\\_1098\\_split\\_library\\_seqs\\_and\\_mapping.tgz
ftp://thebeast.colorado.edu/pub/QIIME\\_DB\\_Public\\_Studies/study\\_1197\\_split\\_library\\_seqs\\_and\\_mapping.tgz
ftp://thebeast.colorado.edu/pub/QIIME\\_DB\\_Public\\_Studies/study\\_1198\\_split\\_library\\_seqs\\_and\\_mapping.tgz
ftp://thebeast.colorado.edu/pub/QIIME\\_DB\\_Public\\_Studies/study\\_1222\\_split\\_library\\_seqs\\_and\\_mapping.tgz
ftp://thebeast.colorado.edu/pub/QIIME\\_DB\\_Public\\_Studies/study\\_1235\\_split\\_library\\_seqs\\_and\\_mapping.tgz
ftp://thebeast.colorado.edu/pub/QIIME\\_DB\\_Public\\_Studies/study\\_1240\\_split\\_library\\_seqs\\_and\\_mapping.tgz
ftp://thebeast.colorado.edu/pub/QIIME\\_DB\\_Public\\_Studies/study\\_1242\\_split\\_library\\_seqs\\_and\\_mapping.tgz
ftp://thebeast.colorado.edu/pub/QIIME\\_DB\\_Public\\_Studies/study\\_1288\\_split\\_library\\_seqs\\_and\\_mapping.tgz
ftp://thebeast.colorado.edu/pub/QIIME\\_DB\\_Public\\_Studies/study\\_1289\\_split\\_library\\_seqs\\_and\\_mapping.tgz
ftp://thebeast.colorado.edu/pub/QIIME\\_DB\\_Public\\_Studies/study\\_1453\\_split\\_library\\_seqs\\_and\\_mapping.tgz
ftp://thebeast.colorado.edu/pub/QIIME\\_DB\\_Public\\_Studies/study\\_1481\\_split\\_library\\_seqs\\_and\\_mapping.tgz
ftp://thebeast.colorado.edu/pub/QIIME\\_DB\\_Public\\_Studies/study\\_1521\\_split\\_library\\_seqs\\_and\\_mapping.tgz
ftp://thebeast.colorado.edu/pub/QIIME\\_DB\\_Public\\_Studies/study\\_1526\\_split\\_library\\_seqs\\_and\\_mapping.tgz
ftp://thebeast.colorado.edu/pub/QIIME\\_DB\\_Public\\_Studies/study\\_1578\\_split\\_library\\_seqs\\_and\\_mapping.tgz
ftp://thebeast.colorado.edu/pub/QIIME\\_DB\\_Public\\_Studies/study\\_1579\\_split\\_library\\_seqs\\_and\\_mapping.tgz
ftp://thebeast.colorado.edu/pub/QIIME\\_DB\\_Public\\_Studies/study\\_1580\\_split\\_library\\_seqs\\_and\\_mapping.tgz
ftp://thebeast.colorado.edu/pub/QIIME\\_DB\\_Public\\_Studies/study\\_1621\\_split\\_library\\_seqs\\_and\\_mapping.tgz
ftp://thebeast.colorado.edu/pub/QIIME\\_DB\\_Public\\_Studies/study\\_1622\\_split\\_library\\_seqs\\_and\\_mapping.tgz
ftp://thebeast.colorado.edu/pub/QIIME\\_DB\\_Public\\_Studies/study\\_1627\\_split\\_library\\_seqs\\_and\\_mapping.tgz
ftp://thebeast.colorado.edu/pub/QIIME\\_DB\\_Public\\_Studies/study\\_1642\\_split\\_library\\_seqs\\_and\\_mapping.tgz
ftp://thebeast.colorado.edu/pub/QIIME\\_DB\\_Public\\_Studies/study\\_1665\\_split\\_library\\_seqs\\_and\\_mapping.tgz
ftp://thebeast.colorado.edu/pub/QIIME\\_DB\\_Public\\_Studies/study\\_1673\\_split\\_library\\_seqs\\_and\\_mapping.tgz
ftp://thebeast.colorado.edu/pub/QIIME\\_DB\\_Public\\_Studies/study\\_1674\\_split\\_library\\_seqs\\_and\\_mapping.tgz
ftp://thebeast.colorado.edu/pub/QIIME\\_DB\\_Public\\_Studies/study\\_1692\\_split\\_library\\_seqs\\_and\\_mapping.tgz
ftp://thebeast.colorado.edu/pub/QIIME\\_DB\\_Public\\_Studies/study\\_1694\\_split\\_library\\_seqs\\_and\\_mapping.tgz
ftp://thebeast.colorado.edu/pub/QIIME\\_DB\\_Public\\_Studies/study\\_1696\\_split\\_library\\_seqs\\_and\\_mapping.tgz
ftp://thebeast.colorado.edu/pub/QIIME\\_DB\\_Public\\_Studies/study\\_1702\\_split\\_library\\_seqs\\_and\\_mapping.tgz
ftp://thebeast.colorado.edu/pub/QIIME\\_DB\\_Public\\_Studies/study\\_1711\\_split\\_library\\_seqs\\_and\\_mapping.tgz
ftp://thebeast.colorado.edu/pub/QIIME\\_DB\\_Public\\_Studies/study\\_1713\\_split\\_library\\_seqs\\_and\\_mapping.tgz
ftp://thebeast.colorado.edu/pub/QIIME\\_DB\\_Public\\_Studies/study\\_1714\\_split\\_library\\_seqs\\_and\\_mapping.tgz
ftp://thebeast.colorado.edu/pub/QIIME\\_DB\\_Public\\_Studies/study\\_1716\\_split\\_library\\_seqs\\_and\\_mapping.tgz
ftp://thebeast.colorado.edu/pub/QIIME\\_DB\\_Public\\_Studies/study\\_1717\\_split\\_library\\_seqs\\_and\\_mapping.tgz
ftp://thebeast.colorado.edu/pub/QIIME\\_DB\\_Public\\_Studies/study\\_1734\\_split\\_library\\_seqs\\_and\\_mapping.tgz
ftp://thebeast.colorado.edu/pub/QIIME\\_DB\\_Public\\_Studies/study\\_1736\\_split\\_library\\_seqs\\_and\\_mapping.tgz
ftp://thebeast.colorado.edu/pub/QIIME\\_DB\\_Public\\_Studies/study\\_1740\\_split\\_library\\_seqs\\_and\\_mapping.tgz
ftp://thebeast.colorado.edu/pub/QIIME\\_DB\\_Public\\_Studies/study\\_1747\\_split\\_library\\_seqs\\_and\\_mapping.tgz
ftp://thebeast.colorado.edu/pub/QIIME\\_DB\\_Public\\_Studies/study\\_1748\\_split\\_library\\_seqs\\_and\\_mapping.tgz
ftp://thebeast.colorado.edu/pub/QIIME\\_DB\\_Public\\_Studies/study\\_1774\\_split\\_library\\_seqs\\_and\\_mapping.tgz
ftp://thebeast.colorado.edu/pub/QIIME\\_DB\\_Public\\_Studies/study\\_1799\\_split\\_library\\_seqs\\_and\\_mapping.tgz
ftp://thebeast.colorado.edu/pub/QIIME\\_DB\\_Public\\_Studies/study\\_1883\\_split\\_library\\_seqs\\_and\\_mapping.tgz
ftp://thebeast.colorado.edu/pub/QIIME\\_DB\\_Public\\_Studies/study\\_2080\\_split\\_library\\_seqs\\_and\\_mapping.tgz
ftp://thebeast.colorado.edu/pub/QIIME\\_DB\\_Public\\_Studies/study\\_2182\\_split\\_library\\_seqs\\_and\\_mapping.tgz
ftp://thebeast.colorado.edu/pub/QIIME\\_DB\\_Public\\_Studies/study\\_2192\\_split\\_library\\_seqs\\_and\\_mapping.tgz
ftp://thebeast.colorado.edu/pub/QIIME\\_DB\\_Public\\_Studies/study\\_2229\\_split\\_library\\_seqs\\_and\\_mapping.tgz
ftp://thebeast.colorado.edu/pub/QIIME\\_DB\\_Public\\_Studies/study\\_2338\\_split\\_library\\_seqs\\_and\\_mapping.tgz

### 1.2 Dereplicate

From this point, we only describe how to construct and analyze the
full dataset. We use vsearch for dereplication and hashing (v1.3.3 or
superior).

```
cd ${HOME}/Earth_Microbiome_Project/

TEMP=$(mktemp --tmpdir=.)

# Dereplication at the study level and hashing
for f in ./study_*/*.fna.gz ; do
    gunzip -c "${f}" > "${f/.gz/}"
    vsearch \
        --derep_fulllength "${f/.gz/}" \
        --sizeout \
        --relabel_sha1 \
        --fasta_width 0 \
        --output "${f/.fna.gz/.fas}"
    cat "${f/.fna.gz/.fas}" >> "${TEMP}"
    rm "${f/.fna.gz/.fas}"
done

# Global dereplication
vsearch \
    --derep_fulllength "${TEMP}" \
    --sizein --sizeout \
    --fasta_width 0 \
    --output EMP_92_studies.fas
rm "${TEMP}"
```

We have a total of 314,871,149 unique sequences (representing
2,254,207,945 reads), avg cluster 7.2, median 1, max 17,084,050. We
have 34,768,030 amplicons with an abundance of at least 2, and
65,246,957 with an abundance of at least 1. Memory consumption maxed
at 40% of the RAM (256 GB total), so ca. 100 GB, for a 53 GB input
file (apparently twice the input size).

### 1.3 Clustering

Swarm is freely available.

```
cd ${HOME}/Earth_Microbiome_Project/

FASTA="EMP_92_studies.fas"
SWARM="/usr/local/bin/swarm -d 1 -t 8 -z"
MAX_MEMORY="245760" # in MB

# Swarm (without the fastidious option)
${SWARM} \
    -w "${FASTA/.fasta/_1_representatives.fasta}" \
    -s "${FASTA/.fasta/_1.stats}" \
    -i "${FASTA/.fasta/_1.struct}" \
    -o "${FASTA/.fasta/_1.swarms}" \
    "${FASTA}"

# Swarm with the fastidious option (recommended)
${SWARM} \
    -f \
    -c "${MAX_MEMORY}" \
    -w "${FASTA/.fasta/_1f_representatives.fasta}" \
    -s "${FASTA/.fasta/_1f.stats}" \
    -i "${FASTA/.fasta/_1f.struct}" \
    -o "${FASTA/.fasta/_1f.swarms}" \
    "${FASTA}"
```

### 1.4 Timings and memory usage

**Machine/OS details**

Machine "hugemem" node in Abel cluster at University of Oslo
CPU Intel(R) Xeon(R) CPU E5-4620 0 @ 2.20GHz
CPUs 4
Cores/CPU 8
Virtual cores 2 Hyperthreading
Kernel Linux version 3.19.1
OS Red Hat CentOS release 6.6
Memory 1024 GB

**Four test runs**

| Dataset | Fastidious | Elapsed time (hh:mm:ss) | Max resident memory (blocks) | Max resident memory (GB) | Time (s) | OTUs | Sequences |  |
| --- | --- | --- | --- | --- | --- | --- | --- | --- |
| small | N | 01:45:05 | 173,471,632 | 41 | 6,305 | 49,138,023 | 154,896,650 |  |
| small | Y | 04:59:35 | 477,742,608 | 114 | 17,975 | 29,485,078 | 154,896,650 |  |
| large | N | 03:40:45 | 358,783,376 | 86 | 13,245 | 107,620,192 | 314,871,149 |  |
| large | Y | 11:28:55 | 1,001,843,952 | 239 | 41,335 | 65,520,237 | 314,871,149 |  |
| small | N | 01:45:34 | 173,471,648 | 41 | 6,334 | 49,138,023 | 154,896,650 |  |
| small | Y | 04:59:00 | 477,742,608 | 114 | 17,940 | 29,485,078 | 154,896,650 |  |
| large | N | 03:42:13 | 358,783,392 | 86 | 13,333 | 107,620,192 | 314,871,149 |  |
| large | Y | 11:31:53 | 1,001,844,000 | 239 | 41,513 | 65,520,237 | 314,871,149 |  |
| small | N | 01:44:31 | 173,471,632 | 41 | 6,271 | 49,138,023 | 154,896,650 |  |
| small | Y | 04:59:53 | 477,742,608 | 114 | 17,993 | 29,485,078 | 154,896,650 |  |
| large | N | 03:41:18 | 358,783,392 | 86 | 13,278 | 107,620,192 | 314,871,149 |  |
| large | Y | 11:29:07 | 1,001,843,968 | 239 | 41,347 | 65,520,237 | 314,871,149 |  |
| small | N | 01:45:19 | 173,471,632 | 41 | 6,319 | 49,138,023 | 154,896,650 |  |
| small | Y | 04:57:53 | 477,742,624 | 114 | 17,873 | 29,485,078 | 154,896,650 |  |
| large | N | 03:40:47 | 358,783,408 | 86 | 13,247 | 107,620,192 | 314,871,149 |  |
| large | Y | 11:23:47 | 1,001,843,984 | 239 | 41,027 | 65,520,237 | 314,871,149 |  |

**Averages**

| Dataset | Fastidious | Elapsed time (hh:mm:ss) | Max resident memory (blocks) | Max resident memory (GB) | Time (s) | OTUs | Sequences | OTU reduction |
| --- | --- | --- | --- | --- | --- | --- | --- | --- |
| small | N | 1:45:07 | 173,471,636 | 41 | 6,307 | 49,138,023 | 154,896,650 |  |
| small | Y | 4:59:05 | 477,742,612 | 114 | 17,945 | 29,485,078 | 154,896,650 | 40,0% |
| large | N | 3:41:16 | 358,783,392 | 86 | 13,276 | 107,620,192 | 314,871,149 |  |
| large | Y | 11:28:26 | 1,001,843,976 | 239 | 41,306 | 65,520,237 | 314,871,149 | 39,1% |

### 1.5 Timmings and OTUs with/without breaking

```
# kl
SWARM="/usr/local/bin/swarm -t 8"
TARA="TARA_V9_264_samples.fas"
TIME="/usr/bin/time"

cd ${HOME}/TARA/data/
for i in {1..3} ; do
    "${TIME}" "${SWARM}" -n < "${TARA}"  # no breaking
    "${TIME}" "${SWARM}" < "${TARA}"     # breaking
    "${TIME}" "${SWARM}" -f < "${TARA}"  # breaking and fastidious
done > /dev/null
```

|  | speed (s) | OTUs |
| --- | --- | --- |
| no breaking | 549 ± 4 | 1,646,172 |
| breaking | 545 ± 4 | 1,720,051 |
| breaking + fastidious | 1,005 ± 150 | 961,498 |

## 2 Statistics on mock-communities

(please see swarm's first paper and supplementary file for technical
details)

The effect of the fastidious option is tested on two mock communities
(even and uneven). Global clustering threshold is set to 99% for
CD-HIT, DNAclust and Usearch. Local clustering threshold is set to *d*
= 1 for swarm v1 and v2. Precision and recall are assessed using
taxonomic assignments as ground-truth. The adjusted Rand summarizes
precision and recall.

| Even | cdhit | dnaclust | swarm v1 | swarm v2 -f | usearch nopresorting | usearch presorting |
| --- | --- | --- | --- | --- | --- | --- |
| Adjusted Rand | 0.833911 | 0.791682 | 0.890808 | 0.900771 | 0.730726 | 0.764851 |
| precision | 0.9917455 | 0.993833 | 0.989226 | 0.989040 | 0.9992455 | 0.9979645 |
| recall | 0.817086 | 0.805298 | 0.900424 | 0.910229 | 0.7290765 | 0.7770075 |

| Uneven | cdhit | dnaclust | Swarm v1 | swarm v2 -f | usearch\_nopresorting | usearch\_presorting |
| --- | --- | --- | --- | --- | --- | --- |
| Adjusted Rand | 0.916296 | 0.88293 | 0.958151 | 0.968902 | 0.7795105 | 0.8055815 |
| precision | 0.992909 | 0.993072 | 0.99204 | 0.991910 | 0.9993245 | 0.995633 |
| recall | 0.90031 | 0.882763 | 0.949209 | 0.959101 | 0.777604 | 0.81194 |

Even = Fusion Golay Uneven = Mirce

| Even | swarm v1 | swarm v2 -f | Diff |
| --- | --- | --- | --- |
| Adjusted Rand | 0.890808 | 0.900771 | 0.009973 |
| precision | 0.989226 | 0.989040 | -0.000186 |
| recall | 0.900424 | 0.910229 | 0.009805 |

| Uneven | Swarm v1 | swarm v2 -f | Diff |
| --- | --- | --- | --- |
| Adjusted Rand | 0.958151 | 0.968902 | 0.010751 |
| precision | 0.99204 | 0.991910 | -0.000130 |
| recall | 0.949209 | 0.959101 | 0.009892 |

The fastidious option improves recall (i.e. limits under-grouping) by
0.009805 (even community) and 0.009892 (uneven community). The
fastidious reduces precision (i.e. some over-grouping) by 0.000186
(even community) and 0.000094 (uneven community). The slight
over-grouping effect of the fastidious option is largely compensated
by the reduction of under-grouping. That result suggests that the
fastidious process correctly grafts small OTUs onto their original
larger OTUs in most cases.

## 3 OTU visualizations (BioMarKs 18S rRNA V4 and V9 datasets)

### 3.1 Obtain the BioMarKs datasets

The dereplicated fasta datasets are available on demand
(mahe@rhrk.uni-kl.de).

### 3.2 Clustering and Visualization

Swarm is ran without the fastidious option, and breaking is
deactivated to illustrate the role of that phase.

We selected two OTUs, one for each marker, to illustrate the way swarm
works and the differences with classic clustering (here represented by
uclust as implemented in usearch).

Code snippets below apply to the V9 dataset. To replicate the analyzes
for the V4 dataset, just replace "v9" with "v4" and target the 54th
OTU.

```
cd ${HOME}/BioMarKs/

# Clustering
FASTA="biomarks_v9.fas"
swarm \
    -d 1 \
    -n \
    -w "${FASTA/.fasta/_1n_representatives.fasta}" \
    -s "${FASTA/.fasta/_1n.stats}" \
    -i "${FASTA/.fasta/_1n.struct}" \
    -o "${FASTA/.fasta/_1n.swarms}" \
    "${FASTA}"

# Visualization (of the 23rd OTU)
OTU=23
GRAPH_PLOT="graph_plot.py"
SWARMS="${FASTA}_1n.swarms"
STRUCT="${FASTA}_1n.struct}"
python "${GRAPH_PLOT}" -s "${SWARMS}" -i "${STRUCT}" -o "${OTU}" -d 1
rm *.graphml
```

#### 3.2.1 Graph plotting script

This python script is available in the swarm repository.

### 3.3 Compare swarm and usearch

```
cd ${HOME}/BioMarKs/

FASTA="biomarks_v9.fas"
SWARMS="${FASTA/.fas/}_1n.swarms"

TMP=$(mktemp)
ALN=$(mktemp)
STATS=$(mktemp)
UC=$(mktemp)
CENTROIDS=$(mktemp)
OTU=23

OTU_FASTA="${FASTA/.fas/}_1n_OTU_${OTU}.fas"

# Extract OTU in fasta format
grep -A 1 -F \
     -f <(head -n "${OTU}" "${SWARMS}" | tail -n 1 | tr " " "\n" | sed -e 's/^/>/') \
     "${FASTA}" | \
    sed -e '/^--$/d' > "${OTU_FASTA}"

# Swarm (with breaking and fastidious)
swarm -d 1 -f -s "${STATS}" < "${OTU_FASTA}" > /dev/null
cat "${STATS}"

# Vsearch pairwise comparison
head -n 6 "${OTU_FASTA}" | sed -e 's/_/;size=/' > "${TMP}"
vsearch --allpairs_global "${TMP}" --alnout "${ALN}" --acceptall --rowlen 0
cat "${ALN}"

# Convert to usearch format
sed -e '/^>/ s/_/;size=/' -e '/^>/ ! y/acgt/ACGT/' "${OTU_FASTA}" > "${TMP}"

# Usearch (3 last main versions, with a global threshold of 97%)
USEARCH8="usearch8.0.1623_i86linux32"
"${USEARCH8}" -cluster_smallmem "${TMP}" -uc "${UC}" \
              -id 0.97 -sortedby size -centroids "${CENTROIDS}"
head "${CENTROIDS}"
echo
echo "Usearch 6"
USEARCH6="usearch6.0.203_i86linux32"
"${USEARCH6}" -cluster_smallmem "${TMP}" -uc "${UC}" \
              -id 0.97 -usersort -centroids "${CENTROIDS}"
head "${CENTROIDS}"
echo
echo "Usearch 7"
USEARCH7="usearch7.0.1001_i86linux32"
"${USEARCH7}" -cluster_smallmem "${TMP}" -uc "${UC}" \
              -id 0.97 -usersort -centroids "${CENTROIDS}"
head "${CENTROIDS}"
echo

# Cleaning
rm "${TMP}" "${ALN}" "${STATS}" "${UC}" "${CENTROIDS}"
```

#### 3.3.1 BioMarKs 18S rRNA V9 OTU 23 fasta file

To simplify replication of our results, we provide the OTU 23 in fasta
format:

>0407735d01645afa65eb6590ed4a099c\_3261
gctcctaccgattggatgagttggtgagtgaattggagtaacagctatctttctgaaagattgttgtattttttaaaatttgcaaactagattatctagaggaaggagaagtcgtaacaaggtttcc
>b006687ac73dce9f8bca2f1f9a98dade\_2015
gctcctaccgattggatgagttggtgagtgaattggagtaacaggtatctttctgaaagattgttgcattttttaaaatttgcaaactagattatctagaggaaggagaagtcgtaacaaggtttcc
>7700225d0c9910e51cb0148e08c9ce75\_821
gctcctaccgattggatgagttggtgagtgaattggagtaacaggtatctttctgaaagattgttgtattttttaaaatttgcaaactagattatctagaggaaggagaagtcgtaacaaggtttcc
>79dd6a535c70c0227ca8a3188238476d\_628
gctcctaccgattggatgagttggtgagtgaattggagtaacagctatctttctgaaagattgttgttattttttaaaatttgcaaactagattatctagaggaaggagaagtcgtaacaaggtttcc
>d20f68c09168a0194afe4b123b6fb270\_345
gctcctaccgattggatgagttggtgagtgaattggagtaacagctatctttctgaaagattgttgtatttttaaaatttgcaaactagattatctagaggaaggagaagtcgtaacaaggtttcc
>c83a74942a554c0eff63294b79c421b1\_317
gctcctaccgattggatgagttggtgagtgaattggagtaacaggtatctttctgaaagattgttgcatttttttaaaatttgcaaactagattatctagaggaaggagaagtcgtaacaaggtttcc
>bd62c4b4816ced63d0bd3e1fdd8bb230\_150
gctcctaccgattggatgagttggtgagtgaattggagtaacagctatctttctgaaagattgttgtatttttaaaaatttgcaaactagattatctagaggaaggagaagtcgtaacaaggtttcc
>13a601b2989208f730c7fc9ffaadf621\_132
gctcctaccgattggatgagttggtgagtgaattggagtaacaggtatctttctgaaagattgttgtatttttaaaatttgcaaactagattatctagaggaaggagaagtcgtaacaaggtttcc
>c37588fa992ef1b1c78d6c556dc5a210\_101
gctcctaccgattggatgagttggtgagtgaattggagtaacaggtatctttctgaaagattgttgcattttttaaaatttgcaaactagattatctagaggaaggagaagtcgtaacaaggttttcc
>99a43800b7682574964769f27977e5ac\_81
gctcctaccgattggatgagttggtgagtgaattggagtaacaggtatctttctgaaagattgttgctattttttaaaatttgcaaactagattatctagaggaaggagaagtcgtaacaaggtttcc
>8e3e395b254a5413ae785714633b35d2\_65
gctcctaccgattggatgagttggtgagtggattggagtaacagctatcttcctgtaagattgttgtatttttttaaaatttgcaaactagattatctagaggaaggagaagtcgtaacaaggtttcc
>bb0a8d4579f94f1c96b9d54c9bc5944b\_64
gctcctaccgattggatgagttggtgagtggattggagtaacagctatcttcctgtaagattgttgtattttttaaaatttgcaaactagattatctagaggaaggagaagtcgtaacaaggtttcc
>089fe655d493ec5f2eacea011e6c2317\_50
gctcctaccgattggatgagttggtgagtgaattggagtaacaggtatctttctgaaagattgttgcattttttaaaaatttgcaaactagattatctagaggaaggagaagtcgtaacaaggtttcc
>1ee57f34ee06b90d7511c46527f4462b\_43
gctcctaccgattggatgagttggtgagtgaattggagtaacaggtatctttctgaaagattgttgcattttttaaaatttgcaaaactagattatctagaggaaggagaagtcgtaacaaggtttcc
>f464592b9fcbad0be632dbca0d25d82f\_43
gctcctaccgattggatgagttggtgagtgaattggagtaacaggtatctttctgaaagattgttgcatttttaaaatttgcaaactagattatctagaggaaggagaagtcgtaacaaggtttcc
>254dda8c183e168b8fec3468d552a529\_39
gctcctaccgattggatgagttggtgagtgaattggagtaacaggtatctttctgaaagattgttgcatttttttaaaatttgcaaactagattatctagaggaaggagaagtcgtaacaaggttttcc
>0de8f97af7c188e8f01fcdbdf2af8b09\_36
gctcctaccgattggatgagttggtgagtgaattggagtaacagctatcttcctgaaagattgttgtattttttaaaatttgcaaactagattatctagaggaaggagaagtcgtaacaaggtttcc
>03545f2aa099cd8e1f043850bc7f55f8\_32
gctcctaccgattggatgagttggtgagtgaattggagtaacagctatctttctgaaagattgttgtattttttaaaattgcaaactagattatctagaggaaggagaagtcgtaacaaggtttcc
>bb2f7874863370c9ec983492d43f85b2\_29
gctcctaccgattggatgagttggtgagtgaattggagtaacagctatctttctgaaagattgttatattttttaaaatttgcaaactagattatctagaggaaggagaagtcgtaacaaggtttcc
>23931d5321d5946670c32889b6fb8287\_28
gcttctaccgattggatgagttggtgagtgaattggagtaacaggtatctttctgaaagattgttgtattttttaaaatttgcaaactagattatctagaggaaggagaagtcgtaacaaggtttcc
>a9be502270843c24272066d56acd8bcd\_28
gctcctaccgattggatgagttggtgagtgaattggagtaacagctatctttctgaaagattgttgttaattttttaaaatttgcaaactagattatctagaggaaggagaagtcgtaacaaggtttcc
>156d430c7760d8279740556afa7e6dc1\_23
gctcctaccgattggatgagttggtgagtgaattggagtaacaggtatctttctgaaagattgttgcattttttaaaaatttgcaaactagattatctagaggaaggagaagtcgtaacaaggttttcc
>21d2e091268b453e3813ba3b13c6c3c1\_22
gctcctaccgattggatgagttggtgagtgaattggagtaacagctatctttctgaaagattgttgtattttttaaaatttgcaaactagattatctagaggaaggagaagtcgtaacaaggttttcc
>df119620d321c4969581ed7d10c2d445\_22
gctcctaccgattggatgagttggtgagtgaattggagtaacaggtatctttctgaaagattgttgcattttttaaaaatttgcaaaactagattatctagaggaaggagaagtcgtaacaaggtttcc
>1fd96a28f4a18babf4aeb0c025fb3e17\_20
gctcctaccgattggatgagttggtgagtgaattggagtaacaggtatctttctgaaagattgttgcattttttaaaatttgcaaactagattatctagatgaaggagaagtcgtaacaaggtttcc
>aa1496be522541215b5609ff3c3eec69\_20
gctcctaccgattggatgagttggtgagtgaattggagtaacaggtatctttctgaaagattgttgcaattttttaaaatttgcaaactagattatctagaggaaggagaagtcgtaacaaggtttcc
>2942f04d19da4a86adaa8adcc3ac5dc5\_19
gctcctaccgattggatgagttggtgagtgaattggagtaacaggtatctttctgaaagattgttgctatttttttaaaatttgcaaactagattatctagaggaaggagaagtcgtaacaaggtttcc
>b2a4d67ad311f9c1afa3f404f67ba746\_19
gctcctaccgattggatgagttggtgagtggattggagtaacagctatcttcctgtaagattgttgttattttttaaaatttgcaaactagattatctagaggaaggagaagtcgtaacaaggtttcc
>693a7a07d7aef949f19fa1f9d01b197f\_18
gctcctaccgattggatgagttggtgagtgaattggagtaacagctatctttctgaaagattgttgcattttttaaaatttgcaaactagattatctagaggaaggagaagtcgtaacaaggtttcc
>55bb445eac7e0a51dce638db482ed691\_17
actcctaccgattggatgagttggtgagtgaattggagtaacagctatctttctgaaagattgttgtattttttaaaatttgcaaactagattatctagaggaaggagaagtcgtaacaaggtttcc
>82f3a739acc2bb64ff45f5c1276be8be\_17
gctcctaccgattggatgagttggtgagtgaattggagtaacagctatctttctgaaagattgttgtattttaaaatttgcaaactagattatctagaggaaggagaagtcgtaacaaggtttcc
>b8edf5e215b4b86eb6f408db9c4cfe9f\_17
gctcctaccgattggatgagttggtgagtgaattggagtaacaggtatctttctgaaagattgttgcatttttttaaaaatttgcaaactagattatctagaggaaggagaagtcgtaacaaggttttcc
>b71135e67f7a846149240dc40f3ab845\_16
gcttctaccgattggatgagttggtgagtgaattggagtaacagctatctttctgaaagattgttgtattttttaaaatttgcaaactagattatctagaggaaggagaagtcgtaacaaggtttcc
>11e754aac088fd8018bb9f39ec7bb38f\_14
gctcctaccgattggatgagttggtgagtgaattggagtaacaggtatctttctgaaagattgttgcatttttttaaaaatttgcaaactagattatctagaggaaggagaagtcgtaacaaggtttcc
>29b2e004c17f4c6b5d26ac23a2c8843d\_13
gctcctaccgattggatgagttggtgagtgaattggagtaatagctatctttctgaaagattgttgtattttttaaaatttgcaaactagattatctagaggaaggagaagtcgtaacaaggtttcc
>2e08c5a8f1f12de29b3666844d40e05a\_13
gctcctaccgattggatgagttggtgagtgaattggagtaacagctatcttcctgaaagattgttgtatttttttaaaatttgcaaactagattatctagaggaaggagaagtcgtaacaaggtttcc
>801fd4038a9c1e911281c6b69ca9d730\_13
gctcctaccgattggatgagttggtgagtgaattggagtaacagctatctttctgaaagattgttgttatttttaaaatttgcaaactagattatctagaggaaggagaagtcgtaacaaggtttcc
>e24e78db35472de11150619a6e3ccc76\_13
gctcctaccgattggatgagttggtgagtgaattggagtaacaggtatcttctgaaagattgttgcattttttaaaatttgcaaactagattatctagaggaaggagaagtcgtaacaaggtttcc
>77952b068a90ee75d64be1873c8bdfcd\_12
gctcctaccgattggatgagttggtgagggaattggagtaacaggtatctttctgaaagattgttgtattttttaaaatttgcaaactagattatctagaggaaggagaagtcgtaacaaggtttcc
>b0e4969cd406766096e70096ed4df7f2\_12
gctcctaccgattggatgagttggtgagtgaattggagtaacagctatcttcctgaaagattgttgttattttttaaaatttgcaaactagattatctagaggaaggagaagtcgtaacaaggtttcc
>e24903cc99655d1816857d72bd98542a\_12
gctactaccgattggatgagttggtgagtgaattggagtaacaggtatctttctgaaagattgttgcattttttaaaatttgcaaactagattatctagaggaaggagaagtcgtaacaaggtttcc
>c254554094967bb389e5d74f1bd4f38f\_11
gctcctaccgattggatgagttggtgagtgaattggagtaacagctatctttctgaaagattgttgtattttttaaaatttgcaaactagattatctagaggaaggagaagtcgtaacaagatttcc
>d19c4869c5667565a4e6f5eb39cee399\_11
gctcctaccgattggatgagttggtgagtgaattggagtaacaggtatctttctgaaagattgttgcattttttaaaatttgcaaactagattatctagaggaagtaaaagtcgtaacaaggtttcc
>04a9a609fd0c82d2dfb422d4087bd9d0\_10
gctcctaccgattggatgagttggtgagtgaattggagtaacagctatctttctgaaagattgttgtattttttaaaatttgacaaactagattatctagaggaaggagaagtcgtaacaaggtttcc
>57f8b65d0e08061c1fd62ffe243c9506\_10
gctactaccgattggatgagttggtgagtgaattggagtaacagctatctttctgaaagattgttgtattttttaaaatttgcaaactagattatctagaggaaggagaagtcgtaacaaggtttcc
>65ca15ccd08da92fbcd0c95ac6124434\_10
gctcctaccgattggatgagttggtgagtgaattggagtaacagctatctttctgaaagattgttgtattttttaaaaatttgcaaactagattatctagaggaaggagaagtcgtaacaaggtttcc
>718c82b8a5f1f864707e755b766eb34b\_10
gctcctaccgattggatgagttggtgagtgaattggagtaacagctatctttctgaaagattgttgtattttaaaaatttgcaaactagattatctagaggaaggagaagtcgtaacaaggtttcc
>a346596ddbfa4e45f2ea94c418d1b948\_10
gctcctaccgattggatgagttggtgagtgaattggagtaacaggtatctttctgaaagattgttgcattttttaaaatttgacaaactagattatctagaggaaggagaagtcgtaacaaggtttcc
>a47ff0754b82727ad6f7d6d67d4ffada\_10
gctcctaccgattggatgagttggtgagtgaattggagtaacagctatctttctgaaagattgttgtaatttttaaaaatttgcaaactagattatctagaggaaggagaagtcgtaacaaggtttcc
>c3d1b798e12e1fb1dd8196c66e753f88\_10
gctcctaccgattggatgagttggtgagtgaattggagtaacaggtatctttctgaaagattgttgcatttttttaaaaatttgcaaaactagattatctagaggaaggagaagtcgtaacaaggttttcc
>da886773d6621a28431d025df8d01d1f\_10
gctcctaccgattggatgagttggtgagtgaattggagtaacaggtatctttctgaaagattgttgcatttttttaaaatttgcaaaactagattatctagaggaaggagaagtcgtaacaaggtttcc
>4ecae6b0af37c50fd7e6dbadb64a94bf\_9
gctcctaccgattggatgagttggtgagtgaattggagtaacagctatctttctgaaagattgttgtattttttaaatttgcaaactagattatctagaggaaggagaagtcgtaacaaggtttcc
>528e5b55e98eb1121c2c7bc94c643734\_9
gctcctaccgattggatgagttggtgagtgaattggagtaacaggtatctttctgaaagattgttgtattttaaaatttgcaaactagattatctagaggaaggagaagtcgtaacaaggtttcc
>6877e30bca620285af7c5266c91f041a\_9
gctcctaccgattggatgagttggtgagtgaattggagtaacaggtatctttctgaaagattgttgcattttttaaaatttgcaaactagattatctagaggaaggagaagttgtaacaaggtttcc
>fc30628e1c0f5efe5dedd7b9c8fc251c\_9
gctcctaccgattggatgagttggtgagtgaattggagtaacaggtatctttctgaaagattgttgcattttttaaaattttgcaaactagattatctagaggaaggagaagtcgtaacaaggtttcc
>fec75dbab9e0a238b625a23e8ab4f8e3\_9
gctcctaccgattggatgagttggtgagtgaattggagtaacaggtatctttctgaaagattgttgcattttttaaaatttgcaaactagattatctagagaaaggagaagtcgtaacaaggtttcc
>01c1c460c16633b7731515b144c604ac\_8
gctcctaccgattggatgagttggtgagtgaattggagtaacaggtatctttctgaaagattgttgctattttttaaaatttgcaaactagattatctagaggaaggagaagtcgtaacaaggttttcc
>31a62a8e53c8c23f290769ee4faa1d79\_8
gctcctaccgattggatgagtttgtgagtgaattggagtaacagctatctttctgaaagattgttgtattttttaaaatttgcaaactagattatctagaggaaggagaagtcgtaacaaggtttcc
>67835940bee89910d51658014a453f6e\_8
gctcctaccgattggatgagttggtgagtgaattggagtaacagctatctttctgaaagattgttgtattttttaaaatttgcaaattagattatctagaggaaggagaagtcgtaacaaggtttcc
>98f9ab277750daa918b33b45fbc8ecac\_8
gctcctaccgattggatgagttggtgagtgaattggagtaacagctatctttctgaaagattgttgtattttttaaaatttgcaaactagattatctagaggaaggagaagtcgtaacaaggtttct
>a4c75ac3480bf8f45a8911cc98665688\_8
gctcctaccgattggatgagttggtgagtgaattggagtaacagctatctttctgaaagattgttgtatttttaaaattgcaaactagattatctagaggaaggagaagtcgtaacaaggtttcc
>d25586bcdaabdc325685369be67628a9\_8
gctcctaccgattggatgagttggtgagtaaattggagtaacagctatctttctgaaagattgttgtattttttaaaatttgcaaactagattatctagaggaaggagaagtcgtaacaaggtttcc
>04d7f4df0136b04cab56686c86fec4a2\_7
gctcctaccgattggatgagttggtgagtgaattggagtaacatgtatctttctgaaagattgttgcattttttaaaatttgcaaactagattatctagaggaaggagaagtcgtaacaaggtttcc
>21608e7afcfef3ba45aea1ffc24a2229\_7
gctcctaccgattggatgagttggtgagtgaattggagtaacagctatctttctgaaagattgttgtattttttaaaatttgcaaactagattatctagaggaaggagaagtcgtaataaggtttcc
>2fcb031bc0e74e0742868f2773181361\_7
gctcctaccgattggatgagttggtgagtgaattggagtaacagctatctttctgaaagattgttgtattttttaaaatttgcaaaactagattatctagaggaaggagaagtcgtaacaaggtttcc
>32e381f83a962048b8a474785874d81e\_7
gctcctaccgattggatgagttggtgagtgaattggagtaacagctatctttctgaaagattgttgtattttttaaaatttgcaaactagattatctagaggaaggagaagtcgtaacaaagtttcc
>342c00c6233818a6c9e6a33207febbd6\_7
gctcctaccgattggatgagttggtgagtgaattggagtaacagctatctttctgaaagattgttgttatttttaaaaatttgcaaactagattatctagaggaaggagaagtcgtaacaaggtttcc
>3cba81f97233a9742a8785838ac78c82\_7
gctcctaccgattggatgagttggtgagtgaattggagtaacagctatctttctgaaagattgttgtattttttaaaatttacaaactagattatctagaggaaggagaagtcgtaacaaggtttcc
>6c8d45ae540745a63fc9ae0ff71576b2\_7
gctcctactgattggatgagttggtgagtgaattggagtaacagctatctttctgaaagattgttgtattttttaaaatttgcaaactagattatctagaggaaggagaagtcgtaacaaggtttcc
>7750fa8eb1dfaf84d1feb814d6f63e7a\_7
gctcctaccgattggatgagttggtgagtgaattggagtaacagctatctttctgaaagattgttgtattttttaatatttgcaaactagattatctagaggaaggagaagtcgtaacaaggtttcc
>8f5274e07ce0dfd6770ef8241acd0a7f\_7
gctcctaccgattggatgatttggtgagtgaattggagtaacagctatctttctgaaagattgttgtattttttaaaatttgcaaactagattatctagaggaaggagaagtcgtaacaaggtttcc
>9757230ff9c2d53940789f05c9581d2c\_7
gctcctaccgattggatgagttggtgagtgaattggagtaacagctatctttctgaaagattgttgtattttttaaaatttgcaaactagactatctagaggaaggagaagtcgtaacaaggtttcc
>a01cdc0eb3042407ae0007fed7a4835a\_7
gctcctaccgattggatgagttggtgagtgaattggagtaacagctatctttctgaaagattgttattattttttaaaatttgcaaactagattatctagaggaaggagaagtcgtaacaaggtttcc
>b01e0688807307c3a3c2ed49f1a30b61\_7
gctcctaccgattggatgagttggtgagtgaattggagtaacagctatctttctgaaagattgttgtatttttttaaaatttgcaaactagattatctagaggaaggagaagtcgtaacaaggtttcc
>b23b4ccf95af5b4e615605f587bae6f4\_7
gctcctaccgattggatgagttggtgagtgaattggagtaacaggtatctttctgaaagattgttgcattttttaaaaattttgcaaaactagattatctagaggaaggagaagtcgtaacaaggttttcc
>0d625bfff25014bbaf81905981450ee5\_6
gctcctaccgattggatgagttggtgagtgaattggagtaacaggtatctctctgaaagattgttgcattttttaaaatttgcaaactagattatctagaggaaggagaagtcgtaacaaggtttcc
>144418816d850c322e8264b493628234\_6
gctcctaccgattggatgagttggtgagtgaattggagtaacaggtatctttctgaaagattgttgtattttttaaaatttgcaaactagattatctagaggaaggagaagtcgtaacaaggttttcc
>1d0318448f1cf3fc6087fab150238c9a\_6
gctcctaccgattggatgagttggtgagtgaattggagtaacagctatctttcttaaagattgttgtattttttaaaatttgcaaactagattatctagaggaaggagaagtcgtaacaaggtttcc
>26874b7fa788d1b426d3a448632800ce\_6
gctcctaccgattggatgagttggtgagtgaattggagtaacagctatctttctgaaagattgttgttaatttttaaaaatttgcaaactagattatctagaggaaggagaagtcgtaacaaggtttcc
>3a7f87fd84da525bbf850ce13978b324\_6
gctcctaccgattggatgaattggtgagtgaattggagtaacagctatctttctgaaagattgttgtattttttaaaatttgcaaactagattatctagaggaaggagaagtcgtaacaaggtttcc
>3cfc3c09563044d8e06d328ccb80d2a3\_6
gctcctaccgattggatgagttggtgagtgaattggagtaacaggtatctttctgaaagattgttgctaattttttaaaatttgcaaactagattatctagaggaaggagaagtcgtaacaaggtttcc
>405251d8ad50e815267d5f2f1b50f704\_6
gctcctaccgattggatgagttggtgagtgagttggagtaacagctatctttctgaaagattgttgtattttttaaaatttgcaaactagattatctagaggaaggagaagtcgtaacaaggtttcc
>4775b6dd52c00134e8edea5f7e8b4c74\_6
gctcctaccgattggatgagttggtgagtgaattggagtaacagctatctttctgaaagattgttgtattttttaaaatttgcaaactagattatctagaggaagtaaaagtcgtaacaaggtttcc
>4b642afc002a5ad529fc939f19383785\_6
gctcctatcgattggatgagttggtgagtgaattggagtaacagctatctttctgaaagattgttgtattttttaaaatttgcaaactagattatctagaggaaggagaagtcgtaacaaggtttcc
>61e8191d3496d804a9a802e51e7d8d14\_6
gctcctaccgattggatgagttggtgagtgaattggagtaacagctatctttctgaaagattgttgtattttttaaaattttgcaaactagattatctagaggaaggagaagtcgtaacaaggtttcc
>6b4f39091e1044b61d45a6134e3f0bd2\_6
gctcctaccgattggatgagttggtgagtgaattggagtaacagctatctttctgaaagattgttgattttttaaaatttgcaaactagattatctagaggaaggagaagtcgtaacaaggtttcc
>74862c43f2ae22bdccd59790ee35bde4\_6
gctcctaccgattggatgagttggtgagtgaattggagtaacaggtatctttctgaaagattgttgcattttttaaaattgcaaactagattatctagaggaaggagaagtcgtaacaaggtttcc
>814d7cc5df90b9ee12ee582dd01881e1\_6
gctcctaccgattggatgagttggtgagtgaattggagtaacagctatctttctgaaagattgttgtatttttaaaatttgcaaactagattatctagaggaaggagaagtcgtaacaaggttttcc
>862d4b638462a38ceb07893a906ae43e\_6
gctcctaccgattggatgagttggtgagttaattggagtaacagctatctttctgaaagattgttgtattttttaaaatttgcaaactagattatctagaggaaggagaagtcgtaacaaggtttcc
>8707d4bac498c70ca7963ff2c4d979c6\_6
gctcctaccgattggatgagttggtgagtgaattggagtaacagctatctttctagaaagattgttgtattttttaaaatttgcaaactagattatctagaggaaggagaagtcgtaacaaggtttcc
>8e5eae52b8fdca97b8f95766f72ac189\_6
gctcctaccgattggatgagttggtgagtgaattggaataacagctatctttctgaaagattgttgtattttttaaaatttgcaaactagattatctagaggaaggagaagtcgtaacaaggtttcc
>977c1bfbee5073110dfc4f9e9301f862\_6
gctcctaccgattggatgagttggtgagtgaattggagtaacagctatctttctgaaagattgttgtattttttaaaatttgcaaactagattatctagaggaaggagaagtcgtaacaaggattcc
>a27e33f09da8bf0234fb0a6ff52ec50e\_6
gctcctaccgattggatgagttggtgagtgaattggagtaacagctatctttctgaaagattgttgtattttttaaaatttgcaaactagattatctagaggaaggagaagtcgtaacaatgtttcc
>cd7945e547da6363ddea02eee7f9134f\_6
gctcctaccgattggatgagttggtgagtgaattggagtaacaggtatctttctgaaagattgttgcatttttaaaatttgcaaactagattatctagatgaaggagaagtcgtaacaaggtttcc
>de7e8886e3c797aca36df84b07f64c86\_6
gctcctaccgattggatgagttggtgagtgaattggagtaacagctatctttctgaaagattgttgtattttttaaaatttgcaaactagattatctagaggaaggagaagtacgtaacaaggtttcc
>eadcfd7b2518d5062168792d2845d5d5\_6
gctcctaccgattggatgagttggtgagtgaattggagtaacaggtatctttctgaaagattgttgcatttttttaaaaatttgcaaaactagattatctagaggaaggagaagtcgtaacaaggtttcc
>ec64e9c50fcdeb53efe195743ecf44f9\_6
gctcctaccgattggatgagttggtgagtggattggagtaacagctatcttcctgtaagattgttgttatttttttaaaatttgcaaactagattatctagaggaaggagaagtcgtaacaaggtttcc
>001b27dcf40d7fc64082be6f569698dd\_5
gctcctaccgattggatgagttggtgagtgaattggagtaacagctatctttctgaaagattgttgtattttttaaaatttgcaaactaaattatctagaggaaggagaagtcgtaacaaggtttcc
>02c15add55e241401c32a2ceb5d3ef14\_5
gctcttaccgattggatgagttggtgagtgaattggagtaacaggtatctttctgaaagattgttgcattttttaaaatttgcaaactagattatctagaggaaggagaagtcgtaacaaggtttcc
>04ebaf250634b96da5f404a4bec81336\_5
gctcctaccgattggatgagttggtgagtgaattggagtaacaggtatctttctgaaagattgttgctaatttttttaaaatttgcaaactagattatctagaggaaggagaagtcgtaacaaggtttcc
>05173b4c6e4c5afdb1c435ceab8a622a\_5
gctcctaccgattggatgagttggtgagtgaattggagtaacagctatcttcctgaaagattgttgtattttttaaaattgcaaactagattatctagaggaaggagaagtcgtaacaaggtttcc
>13dcd365ae5c581c7f8feacc05797db9\_5
gctcctaccgattggatgagttggtgagtgaattggagtaacagctatctttctgaaagattgttgtattttttaaaatttgcaaactagattatgtagaggaaggagaagtcgtaacaaggtttcc
>23340d61030186f6951f3fa9dbeca840\_5
gctcctaccgattggatgagttggtgagtggattggagtagcagctatcttcctgtaagattgttgtattttttaaaatttgcaaactagattatctagaggaaggagaagtcgtaacaaggtttcc
>384d071fd8964eddee17738106bc2ae8\_5
gctcctaccgattggatgagttggtgagtgaattggagtaacaggtatctttctgaaagattgttgtattttttaaaatttgcaaactaaattatctagaggaaggagaagtcgtaacaaggtttcc
>3e29d499c69736708c2a404889ef84c0\_5
gctcctaccgattggatgagttggtgagtgaattggagtaacagatatctttctgaaagattgttgtattttttaaaatttgcaaactagattatctagaggaaggagaagtcgtaacaaggtttcc
>45f3aff9102b08aec9bfaa400372feeb\_5
gctcctaccgattggatgagttggtgagtgaattggagtaacagctatctttctgaaagattgttgtactttttaaaatttgcaaactagattatctagaggaaggagaagtcgtaacaaggtttcc
>63b2622da460b7351450a25fddc188e7\_5
gctcctaccgattggatgagttggtgagtgaattggagtaacagctatcttctgaaagattgttgtattttttaaaatttgcaaactagattatctagaggaaggagaagtcgtaacaaggtttcc
>66e30223f74a8664471648d0d5e56501\_5
gctcctaccgattggatgagttggtgagtgaattggagtaacagctatctttctgaaagattgttgtattttataaaatttgcaaactagattatctagaggaaggagaagtcgtaacaaggtttcc
>7756f201a06161f57d08147424da6150\_5
gctcctaccgattggatgagttggtgagtggattggagtaacagctatcttcctgtaagattgttgtatttttttaaaattgcaaactagattatctagaggaaggagaagtcgtaacaaggtttcc
>8975e9dbda6149c018560390148c5858\_5
gctcctaccgattggatgagttggtgagtgaattggagtaacaggtatctttctgaaagattgttgcatttttttaaaatttgcaaaactagattatctagaggaaggagaagtcgtaacaaggttttcc
>90874be90210ced23bed3f1be9d7d65f\_5
gctcctaccgattggatgagttggtgagtgaatttgagtaacagctatctttctgaaagattgttgtattttttaaaatttgcaaactagattatctagaggaaggagaagtcgtaacaaggtttcc
>9138c1ff8594ca6099299f3296b1586c\_5
gctcctaccgattggatgagttggtgaatgaattggagtaacagctatctttctgaaagattgttgtattttttaaaatttgcaaactagattatctagaggaaggagaagtcgtaacaaggtttcc
>9a328f969ac7d62726ee850b170e7652\_5
gctcctaccgattggatgagttggtgagtgaattggagtaacaggtatctttctgaaagattgttgcattttttaaaattttcaaactagattatctagaggaaggagaagtcgtaacaaggtttcc
>9fb8d0c0fa02d697cabd6aa9b0dcf0e7\_5
gctcctaccgattggatgagttggtgagtgaattggagtaacagctatctttctgaaagattgttgtattttttaaaatttgcaaactagattatttagaggaaggagaagtcgtaacaaggtttcc
>aaf3c3eb2d95dded731f5cafd5368fb1\_5
gctcctaccgattggatgagttggtgagtgaattggagtaacagctatctttctgaaagattgttgtattttttaaaatttgcaaactagattttctagaggaaggagaagtcgtaacaaggtttcc
>b4b61831f71ce1a97f4803a390bd35cd\_5
gctcctaccgattggatgagttggtgagtgaattggagtaacagctatctttctgaaagattgttgtattttttaaaatttgcaaactagattatctaaaggaaggagaagtcgtaacaaggtttcc
>ba2ab12a7b8f072fad4839b1b62e160c\_5
gctcctaccgattggatgagttggtgagttaattggagtaacaggtatctttctgaaagattgttgcattttttaaaatttgcaaactagattatctagaggaaggagaagtcgtaacaaggtttcc
>ca00e26a7df3f23f369dac79b21d6b9a\_5
gctcctaccgattggatgagttggtgagtgaattggagtaacaggtatctttctgtaaagattgttgcattttttaaaatttgcaaactagattatctagaggaaggagaagtcgtaacaaggtttcc
>ca7543f91d70e7fefe6212602a1a0cb9\_5
gctcctaccgattggatgagttggtgagtgaattggagtaacaggtatctttctgaaagattgttgtattttttaaaatttgcaaactaggttatctagaggaaggagaagtcgtaacaaggtttcc
>d6b2935c8c9a6f6d7ddb928e873956b8\_5
gctcctaccgattggatgagttggtgagtgaattggagtaacagctatcttcctgaaagattgttgttatttttttaaaatttgcaaactagattatctagaggaaggagaagtcgtaacaaggtttcc
>db03715bc3885a6e16b5088d8c7123b2\_5
gctcctaccgattggatgagttggtgagtgaattggagtaacaggtatctttctgaaagattgttgcattttaaaatttgcaaactagattatctagaggaaggagaagtcgtaacaaggtttcc
>ea61046867c4770cbc4724ec496ce56b\_5
gctcctaccgattggatgagttggtgagtgaattggagtaacagctatctttctgaaagattgttgttattttttaaaatttgcaaactagattatctagaggaaggagaagtcgtaacaaggttttcc
>eed7036e72099ef6f3299445baf28f78\_5
gctcctaccgattggatgagttggtgagtgaattggagtaacaggtatctttctgaaagattgttgcattttttaaaatttgcaaactagattatctagaggaaggagaagtcgtaacaagatttcc
>f4d36237d8484b449b9c141daed45cc8\_5
gctcctaccgattggatgagttggtgagtgaattggagtaacaggtatctttctgaaagattgttgcaatttttttaaaatttgcaaactagattatctagaggaaggagaagtcgtaacaaggtttcc
>f982dc37350128649a314701cdcee9d6\_5
gctcctaccgattggatgagttggtgagtgaattggagtaacaggtatctttctgaaagattgttgcattttttaaaatttgcaaactagattatctagaggaaggagaagtcgtaataaggtttcc
>fcf8fb6cac3ba44aade9c744a4692070\_5
gctcctaccgattggatgagttggtgagtgaattggagtaacaggtatctttctgaaagattgttgcattttttaaaatttgcaaattagattatctagaggaaggagaagtcgtaacaaggtttcc
>ff0132ba89e129e5a7d602f6293790d9\_5
gctcctaccgattggatgagttggtgagtgaattggagtaacagctatctttctgaaagattgttgtattttttaaaatttgcaaagtagattatctagaggaaggagaagtcgtaacaaggtttcc
>093483588b941f4b2c88c2add977def4\_4
gctcctaccgattggatgagttggtgagtgaattggagtaacagctatcttcctgaaagattgttgtatttttaaaatttgcaaactagattatctagaggaaggagaagtcgtaacaaggtttcc
>1169855322afa6c30a4bb6b6000495a0\_4
gctcctaccgattggatgagttggtgagtggattggagtaacagctatctttctgaaagattgttgtattttttaaaatttgcaaactagattatctagaggaaggagaagtcgtaacaaggtttcc
>169656251baa934f5b2e92645cfc23aa\_4
gctcctaccgattggatgagttggtgagtgaattggagtaacagctatctttctgaaagattgttgttattttttaaaatttgcaaactagattatctagaggaaggagaagtcgtaacaaggttttc
>19750de59700f26754a03c7eeb1abebf\_4
gctcctaccgattggatgagttggtgagtgaattggagtaacagctatctttctgaaaaattgttgtattttttaaaatttgcaaactagattatctagaggaaggagaagtcgtaacaaggtttcc
>1ae02a13b1b03e99d677b3d254439a47\_4
gctcctatcgattggatgagttggtgagtgaattggagtaacaggtatctttctgaaagattgttgtgttttttaaaatttgcaaactagattatctagaggaaggagaagtcgtaacaaggtttcc
>208c74f54742221ccc216f1ebdb118b6\_4
gctcctaccgattggatgagttggtgagtgaattggagtaacaggtatctttctgaaagattgatgcattttttaaaatttgcaaactagattatctagaggaaggagaagtcgtaacaaggtttcc
>2d18b47cc5caf9a1f0acc3c12b37d87e\_4
gctcctaccgattggatgagttggtgagtgaattggagtaacaggtatctttctgaaagattgttgtattttttaaaatttgcaaactagattatctagaggaaggagaagtcgtaacaaggttcc
>376648d37bb2a89ff17bb2819865c7e6\_4
gctcctaccgattggatgagttggtgagtgaattggagtaacagctatctttctgaaagattgttgtattttttaaaatttgcaaactatattatctagaggaaggagaagtcgtaacaaggtttcc
>3a10b6eb8d470359ac980374d47b736c\_4
gctcctaccgattggatgagttggtgagtgaattggagtaataggtatctttctgaaagattgttgcattttttaaaatttgcaaactagattatctagaggaaggagaagtcgtaacaaggtttcc
>4d137a49cb8a691949f2d45339cc8771\_4
gctcctaccgattggatgagttggtgagtgaattggagtaacagctatctttctgaaagattgttgtaattttttaaaatttgcaaactagattatctagaggaaggagaagtcgtaacaaggtttcc
>4eab0770d4ee01801e719d36500b0e4f\_4
gctcctaccgattggatgagttggtgagtgaattggagtaacagctatctttctgaaagattgttgttattttttaaaatttgcaaattagattatctagaggaaggagaagtcgtaacaaggtttcc
>519bec4287e2c68ab9d31efdaef8180e\_4
gctcctaccgattggatgagttggtgagtgaattggagtaacagctatctttctgaaatattgttgtattttttaaaatttgcaaactagattatctagaggaaggagaagtcgtaacaaggtttcc
>55a55256bc57ff17f1d95f1ea9ac59c1\_4
gctcctaccgattggatgagttggtgagtgaattggagtaacagctatctttctgaaagattattgtattttttaaaatttgcaaactagattatctagaggaaggagaagtcgtaacaaggtttcc
>6ba4681a5eb3db2e1b20861bd9a980d7\_4
gctcctaccgattggatgagttggtgagtgaattggagtaacaggtatctttctgaaagattgttgcatttttaaaatttacaaactagattatctagaggaaggagaagtcgtaacaaggtttcc
>74a35a70c965575545fff15008e2ee83\_4
gctcctaccgattggatgagttggtgagtgaattggagtaacagctattttctgaaagattgttgtatttttaaaatttgcaaactagattatctagaggaaggagaagtcgtaacaaggtttcc
>81a94c447942193d46d7083a8dc8e679\_4
gctcctaccgattggatgagttggtgagtgaattggagtaacaggtatctttctgaaagattgttgcattttttaaaatttgcaaactagattatctagaggaaggagaagtcgttaacaaggtttcc
>8624191c5ef7938e49d440e6cc377c11\_4
gctcctaccgattggatgagttggtgagtgaattggagtaacagctatctttctgaaagattgttgtattttttaaaatttgcaaactagattatctagaggaaggagaagttgtaacaaggtttcc
>950d2d82ef8ede959734e6e4716ecd88\_4
gctcctaccgattggatgagttggtgagtgaattggagtaacagctatctttctgaaagattgttgtattttttaaaatttgcaaactagattacctagaggaaggagaagtcgtaacaaggtttcc
>a7cb9e3926e0656f9e93fa7f9657ef20\_4
gctcctaccgattggatgagttggtgagtgaattggagtaacagctatctttctgaaagattgtttgtattttttaaaatttgcaaactagattatctagaggaaggagaagtcgtaacaaggtttcc
>aa07e7b906a52599dc021f09de49a3da\_4
gctcctaccgattggatgagttggtgagtgaattggagtaacagctatctttctgaaagattgttgtattttttaaaatttgcaaactacattatctagaggaaggagaagtcgtaacaaggtttcc
>acf6ab4a933bb409a67731914781caa5\_4
gctcctaccgattggatgagttggtgagtgaattggagtaacagctgtctttctgaaagattgttgtattttttaaaatttgcaaactagattatctagaggaaggagaagtcgtaacaaggtttcc
>b00fbb606c9c510a59d2b7c06a177c0c\_4
gctcctaccgattggatgagttagtgagtgaattggagtaacagctatctttctgaaagattgttgtattttttaaaatttgcaaactagattatctagaggaaggagaagtcgtaacaaggtttcc
>c44f54f440bb97a037db3fc960c77bec\_4
gctcctaccgattggatgagttggtgagtgaattggagtaacagctatctttctgaaagattgttgttattttttaaaatttgcaaactagattatctagaggaagtagaagtcgtaacaaggtttcc
>da4720a9d204e385b06e613aeec72ce4\_4
gctcctaccgattggatgagttggtgagtgaattggagtaacaggtatctttctgaaagattgttgcattttttaaaaatttgcaaaactagattatctagaggaaggagaagtcgtaacaaggttttcc
>e19c6c6c50ad151e2efc19b987767a98\_4
gctcctaccgattggatgagttggtgagtgaattggagtaacaggtatctttctgaaagattgttgcattttttaaaatttgcaaactagattatctagaggaaggagaagtcgtaacaaagtttcc
>e6bc58d9bedcb0c3f12b9ea60a6ef9fa\_4
actcctaccgattggatgagttggtgagtgaattggagtaacaggtatctttctgaaagattgttgcattttttaaaatttgcaaactagattatctagaggaaggagaagtcgtaacaaggtttcc
>e896cdfe7ebd57e3849d6721b0534df0\_4
gctcctaccgattggatgagttggtgagtgaattggagtaacaggtatatttctgaaagattgttgcattttttaaaatttgcaaactagattatctagaggaaggagaagtcgtaacaaggtttcc
>f2829895422c22d28264d9b960ebd6e9\_4
gctcctaccgattggatgagttggtgagtgaattggagtaacaggtatctttctgaaagattgttgtattttttaaaatttgcaaattagattatctagaggaaggagaagtcgtaacaaggtttcc
>f98d13e2771d85e4cc2bd57385a9d309\_4
gctcctaccgattggatgagttggtgagtgaattggagtaacagctatctttctggaagattgttgtattttttaaaatttgcaaactagattatctagaggaaggagaagtcgtaacaaggtttcc
>fc6a7e4453ef04eaebe217b2ced7faaf\_4
gctcctaccgattggatgagttggtgagtgaattggagttaacagctatctttctgaaagattgttgtattttttaaaatttgcaaactagattatctagaggaaggagaagtcgtaacaaggtttcc
>00cfa031d8bf8d1f66235d5eed073d98\_3
gctcctaccgattggatgagttggtgagtgaattggagtaacagctatctttctgaaagattgttgtattttttaaaatttgcaaactagattatctagaggaaggagaagtcgtgacaaggtttcc
>04a58ea3165a8a59a37add2135e51c55\_3
gctcctaccgattggatgagttggtgagtggattggagtaacagctatcttcctgtaagattgttgtaatttttaaaatttgcaaactagattatctagaggaaggagaagtcgtaacaaggtttcc
>065cb05d0c0fcaa72a78328eb34edfd1\_3
gctcctaccgattggatgagttggtgagtgaattggagtaacagctatctttgtgaaagattgttgtattttttaaaatttgcaaactagattatctagaggaaggagaagtcgtaacaaggtttcc
>0c9bb1710d28d60cf76954bc747da535\_3
gctcctaccgattggatgagttggtgagtgaattggagtaacaggtatctttctgaaagattgttgcattttttaaaatttgcaaactagattatctagaggaaggagaagtcgtaacaaggttttc
>17b0b1ecbdf75dd8631db8e908dd3fed\_3
gctcctaccgattggatgagttggtgagtgaattggagtaacagctatctttctgaaagattgttgtattttttaaaatttgcaaactagattatctagaagaaggagaagtcgtaacaaggtttcc
>1c9ea07edba105121574a4e7c131a2b7\_3
gctcctaccgattggatgagttggtgagtgaattggtgtaacagctatctttctgaaagattgttgttattttttaaaatttgcaaactagattatctagaggaaggagaagtcgtaacaaggtttcc
>2303814cf1ad8ec5cc97685c63ba557c\_3
gctcctaccgattggatgagttggtgagtggattggagtaacagctatcttcctgtaagattgttgtaattttttaaaatttgcaaactagattatctagaggaaggagaagtcgtaacaaggtttcc
>25fd4b5a5e6316e1eb267a5ed75dc846\_3
gctcctaccgattcgatgagttggtgagtgaattggagtaacaggtatctttctgaaagattgttgcatttttttaaatttgcaaactagattatctagaggaaggagaagtcgtaacaaggtttcc
>2a0f8130feca89438bd1c8585afed0ee\_3
gctcctaccgattggatgagttggtgagtgaattggagtaacagctatctttctaaaagattgttgtattttttaaaatttgcaaactagattatctagaggaaggagaagtcgtaacaaggtttcc
>2c59413ef626cc5407167255a356a64b\_3
gctactaccgattggatgagttggtgagtgaattggagtaacaggtatctttctgaaagattgttgtattttttaaaatttgcaaactagattatctagaggaaggagaagtcgtaacaaggtttcc
>2e587518ac1d611f9b513750a36d728a\_3
gctcctaccgattggatgagttggtgagtgaattggagcaacagctatctttctgaaagattgttgtattttttaaaatttgcaaactagattatctagaggaaggagaagtcgtaacaaggtttcc
>32f3f9da4ecf84080cecde4ed168ab56\_3
gctcctaccgattggatgagttgttgagtgaattggagtaacaggtatctttctgaaagattgttgcattttttaaaatttgcaaactagattatctagaggaaggagaagtcgtaacaaggtttcc
>341d6a1c95cfea3aad31ba18460c90a4\_3
gctcctaccgattggatgagttggtgagtgaattggagtaacatgtatctttctgaaagattgttgcatttttaaaatttgcaaactagattatctagaggaaggagaagtcgtaacaaggtttcc
>345aa8f744a18c4a25357007b1a823b0\_3
gctcctaccgattggatgagttggtgagtgaattggagtaacaggtatctttctgaaagattgttgcattttttaaaatttgcaaactagattatctagaggaaggagaagtacgtaacaaggtttcc
>3c42e2d40349741617dd9a37c22db599\_3
gctcctaccgattggatgagttggtgagtgaattggagtaacaggtatctttctgaaagattgttgcatttttaaaatttgcaaaactagattatctagaggaaggagaagtcgtaacaaggtttcc
>47ef89de2d2599258c6d873ec1cdb510\_3
gctcctaccgattggatgagttggtgagtgaattggagtaacaggtatctttctgaaagattgttttattttttaaaatttgcaaactagattatctagaggaaggagaagtcgtaacaaggtttcc
>539656648785c1623228ad878d18aff7\_3
gctcctaccgattgaatgagttggtgagtgaattggagtaacagctatctttctgaaagattgttgtattttttaaaatttgcaaactagattatctagaggaaggagaagtcgtaacaaggtttcc
>561590af47ba51248291e93b71b6ae3e\_3
gctcctaccgattggatgagttggtgagtgaattggagtaacaggtatctttctgaaagattgttgcattttttaaaatttgcaaactagattatctagaggaaggagaagtcgtaacaaggtttac
>57d6cbfe41b6d6d00363202bc86aa52f\_3
gctcctaccgattggatgagttggtgaatgaattggagtaacaggtatctttctgaaagattgttgcattttttaaaatttgcaaactagattatctagaggaaggagaagtcgtaacaaggtttcc
>5c0295bfb9d06d78ccce099b989cc91b\_3
actcctaccgattggatgagttggtgagtgaattggagtaacaggtatctttctgaaagattgttgtattttttaaaatttgcaaactagattatctagaggaaggagaagtcgtaacaaggtttcc
>5d595ef6fc47a5c4a67f658b6853a08f\_3
gctcctaccgattggatgagttggtgagtgaattggagtaacaggtatctttctgaaagattgttgcatttttaaaaatttgcaaaactagattatctagaggaaggagaagtcgtaacaaggtttcc
>5daa08ed590a129aa3dd106be651220a\_3
gcttctaccgattggatgagttggtgagtgaattggagtaacaggtatctttctgaaagattgttgtattttttaaaatttgcaaactagattatctagaggaaggagaagtcgtaacaagatttcc
>5df70be6593ec5976ff2c7e084daee4d\_3
gctcctaccgattggatgagttggtgagtgaattggagtaacaggtatctttctgaaagattgttgcattttttaaaattgcaaactagattatctagaggaaggagaagtcgtaacaaggttttcc
>5e39202345564e7573409187544fdeb7\_3
gctcctaccgattcgatgagttggtgagtgaattggagtaacagctatctttctgaaagattgttgtattttttaaaatttgcaaactagattatctagaggaaggagaagtcgtaacaaggtttcc
>5f4d6c7b93c939bfcf26474d255ea951\_3
gctcctaccgattggatgagttggtgagtgaattggagtaacaggtatctttctgaaagattgttgtattttttaaaatttgcaaactagattatctagaggaagtaaaagtcgtaacaaggtttcc
>60a9a7e9a53c7b7a5f7fa0984d1f8247\_3
gctcctaccgattggatgagttggtgagtgaattggagtaacaggtatcttctgaaagattgttgcattttttaaaatttgcaaactagattatctagaggaaggagaagtcgtaacaaggttttcc
>62de69eb34c3772716db94267cc44c86\_3
gctcctaccgattggatgagttggtgagtgtaattggagtaacagctatctttctgaaagattgttgtattttttaaaatttgcaaactagattatctagaggaaggagaagtcgtaacaaggtttcc
>6457a5ba79a256172cf22bb6c1b22c7f\_3
gctcttaccgattggatgagttggtgagtgaattggagtaacagctatctttctgaaagattgttgttattttttaaaatttgcaaactagattatctagaggaaggagaagtcgtaacaaggtttcc
>64bb0ab52ce6903e5c69e3b43f4500e0\_3
gctcctaccgattggatgagttggtgagtgaattggagtaacagctatctttctgaaagattgttgtattttttaaaatttgcaaactagattatctagaggaaggagaaatcgtaacaaggtttcc
>65fbb3e2fc32f08daa7962346d4d4b6d\_3
gctcctaccgattggatgagttggtgagtgaattggagtaacagctatctttctgaaagattgttttattttttaaaatttgcaaactagattatctagaggaaggagaagtcgtaacaaggtttcc
>67c930e54a7a08b2366ce8cc8d5ea2e4\_3
gctcctaccgattggatgagttggtgagtgaattggagtaacagctttctttctgaaagattgttgtattttttaaaatttgcaaactagattatctagaggaaggagaagtcgtaacaaggtttcc
>688461445d92e2ffc9d2d48f1e8f1861\_3
gctcctaccgattggatgagttggtgagtgaattggagtaacagctatctttctgaaagattgttgttattttaaaatttgcaaactagattatctagaggaaggagaagtcgtaacaaggtttcc
>68ba2a9a8e400e78388aa75dfd58b1ae\_3
gctcctaccgattggatgagttggtgagtgaattggagtaacagctatctttctgaaagattgttgtagttttttaaaatttgcaaactagattatctagaggaaggagaagtcgtaacaaggtttcc
>6949df7834a9eef58353d2cc3d518332\_3
gctcctaccgaatggatgagttggtgagtgaattggagtaacagctatctttctgaaagattgttgtattttttaaaatttgcaaactagattatctagaggaaggagaagtcgtaacaaggtttcc
>6ed9b1ccd49f09978f404734fa07858a\_3
gttcctaccgattggatgagttggtgagtgaattggagtaacaggtatctttctgaaagattgttgcattttttaaaatttgcaaactagattatctagaggaaggagaagtcgtaacaaggtttcc
>7869268d4882da1e2ded305521aaf85d\_3
gctcctaccgattggatgagttggtgagtgaattggagtaacagctatctttctgaaagattgttgtattttttaaaatttgcaaactagattatctagaggaaggagaagtcgtaacaaggtctcc
>7b97a442c033962b814a77bb5c3b468c\_3
gctcctaccgattggatgagttggtgagtgaattggactaacagctatctttctgaaagattgttgtattttttaaaatttgcaaactagattatctagaggaaggagaagtcgtaacaaggtttcc
>812c5b79d3f813709544dbf30a71d97d\_3
gttcctaccgattggatgagttggtgagtgaattggagtaacagctatctttctgaaagattgttgtattttttaaaatttgcaaactagattatctagaggaaggagaagtcgtaacaaggtttcc
>8183b8a208455a3c2bce5ba94e80612c\_3
gctcctaccgattggatgagttggtgagtgaattggagtaacaggtatctttctgaaagattgttacattttttaaaatttgcaaactagattatctagaggaaggagaagtcgtaacaaggtttcc
>83bbfb1f409ab01fdd3cb237147ab002\_3
gctcctactgattggatgagttggtgagtgaattggagtaacaggtatctttctgaaagattgttgcattttttaaaatttgcaaactagattatctagaggaaggagaagtcgtaacaaggtttcc
>84640eb324a033d79cfbc3bbddba1547\_3
gctcctaccgattggatgagttggtgagtgaattggagtatcagctatctttctgaaagattgttgtattttttaaaatttgcaaactagattatctagaggaaggagaagtcgtaacaaggtttcc
>85acf094fd40803efface79d0064e5c9\_3
gctcctaccgattggatgagttggtgagtgaattggagtaacagctatctttctgaaagattgttgtattttttaaaatttgcaaactagattatctagaggaagaagaagtcgtaacaaggtttcc
>8a7149af370a67d445dde82485a191df\_3
gctcctaccgattggatgagttggtgagtgaattggagtaacagctatctttctgaaagattgttgtattttttaaaatttgcaaactagattatctagaggaaggaaaagtcgtaacaaggtttcc
>8f602b1b1a0cbf9da651ec0a5cf6575d\_3
gctcctaccgattggatgagttggtgagtgaattggagtaacagctatctttctgaaagattgttgtatttttaaaaatttgcaaactagattatctagaggaaggagaagtcgtaacaaggttttcc
>909e04925b0b342dd525bb197995dc94\_3
gctcctaccgattggatgagttggtgagtgaattggagtaacagctatctttctgaaagattgttgttattttttaaaatttgcaaactagattatctagaggaaggagaaatcgtaacaaggtttcc
>92b520703bea4f92823bc5316b95032f\_3
gctcctaccgattggatgagttggtgagtgaattggagtaacaggtatctttctgaaagattgttgtattttttaaaattgcaaactagattatctagaggaaggagaagtcgtaacaaggtttcc
>93a23cd1e37746aca2aa560eec89934a\_3
gctcctaccaattggatgagttggtgagtgaattggagtaacaggtatctttctgaaagattgttgcattttttaaaatttgcaaactagattatctagaggaaggagaagtcgtaacaaggtttcc
>95e8f690b4780583ae9cc3148a034a16\_3
gctcctaccgattggatgagttggtgagtgaattggagtaacagctatctttctgaaagattgttgtattttttaaaatttgcaaactagtttatctagaggaaggagaagtcgtaacaaggtttcc
>9d251e0e787399e41ab5ab7c0a2ae310\_3
gctcctaccgattggatgagttggtgagtgaattggagtaacagctatctttctgaaagattgttgtattttttaaattttgcaaactagattatctagaggaaggagaagtcgtaacaaggtttcc
>a00468c5e911f75eb90934afb6230926\_3
gctcctaccgattggatgagttggtgagtgaattggagtaacagctatctttctgaaagattgttgtattttttaaaatttgcaaactagattatctagaggaaggagaggtcgtaacaaggtttcc
>a261ae044eeb26c1c746effdc12e19f0\_3
gctcctaccgattggatgagttggtgagtgaattggagtaacaggtatctttctgaaagattgttgtattttttaaaatttgcaaactaagattatctagaggaaggagaagtcgtaacaaggtttcc
>a2a445b8b4c7792b1bfe73fe0e54da25\_3
gctcctaccgattggatgagttggtgagtgaattggagtaacaggtatctttctgaaagattgttgcattttttaaaatttgcaaactagattttctagaggaaggagaagtcgtaacaaggtttcc
>b0b9ac538a3f964a84e3e1a6047d5637\_3
gctcctaccgattggatgagctggtgagtgaattggagtaacagctatctttctgaaagattgttgtattttttaaaatttgcaaactagattatctagaggaaggagaagtcgtaacaaggtttcc
>b101f4d2a7053cb98eac271a55699f9e\_3
gctcctaccgattggatgagttggtgagtgaattggagtaacagctatctttctgaaagattgttgtattttttaaaatttgcaaactagattatctagaggaaggagaagtcgtaacaaggttttc
>b219afb6990a6e610129e2ea4b910ead\_3
gctcctaccgattggatgagttggtgagtgaattggattaacagctatctttctgaaagattgttgtattttttaaaatttgcaaactagattatctagaggaaggagaagtcgtaacaaggtttcc
>b3bb37bebcaa286c15a3445fb8c5a6f9\_3
cgctcctaccgattggatgagttggtgagtgaattggagtaacagctatctttctgaaagattgttgtattttttaaaatttgcaaactagattatctagaggaaggagaagtcgtaacaaggtttcc
>b5dfc337ee35a389e6a931ee09296371\_3
gctcctaccgattggatgagttggtgagtgaattggagtaacaggtatctttctgaaagattgttgtattttttaaaatttgcaaagtagattatctagaggaaggagaagtcgtaacaaggtttcc
>b89b4e54b4fb23ac6fbbaf6fc698f644\_3
gctcctaccgattggatgagttggtgagtgaattggagtaacagctatctttctgaaagattgttgtattttttaaaatttgcaaacaagattatctagaggaaggagaagtcgtaacaaggtttcc
>bc0e6a7b3cdedd71e56c297338883127\_3
gctcctaccgattggatgagttggtgagtgaattgaagtaacagctatctttctgaaagattgttgtattttttaaaatttgcaaactagattatctagaggaaggagaagtcgtaacaaggtttcc
>bca2140137e4c597ec0cbf5420f0a1d7\_3
gctcctaccgattggatgagttggtgagtgaattggagtaacagctatctttctgaaagattgttgtattttttaaaatttgcaagctagattatctagaggaaggagaagtcgtaacaaggtttcc
>c11ba9d436c771b5e6cf14d64e36d4b3\_3
gctcctaccgattggataagttggtgagtgaattggagtaacagctatctttctgaaagattgttgtattttttaaaatttgcaaactagattatctagaggaaggagaagtcgtaacaaggtttcc
>c1e4da308ce8b9692583e7cdf3c134fe\_3
gctcctaccgattggatgagttggtgagtgaattggagtaacagctatctttctgaaagattgttgttatttttttaaaatttgcaaactagattatctagaggaaggagaagtcgtaacaaggtttcc
>c2090354a9009a6a803e86948e83fed1\_3
gctctaccgattggatgagttggtgagtgaattggagtaacagctatctttctgaaagattgttgtattttttaaaatttgcaaactagattatctagaggaaggagaagtcgtaacaaggtttcc
>c4f4e7700830610d1ebe9073be24d3a9\_3
gctcctaccgattggatgagttggtgagtgaattggagtaacagctatctttctgaaagattgttatatttttaaaatttgcaaactagattatctagaggaaggagaagtcgtaacaaggtttcc
>c74aff6779fea767b3b86cee57503fdc\_3
gctcctaccgattggatgagttggtgagtgaattggagtaacagctatctttctgaaagattgttgtatttttaaaatttgcaaactagattatctagaggaaggagaagtcgtaactaaggtttcc
>c8d7e79ec08881898e3744ae12bdaee0\_3
gctcctaccgattggatgagttggtgagtgaattggagtaacagctatctttctgaaagattgttgtattttttaaaatctgcaaactagattatctagaggaaggagaagtcgtaacaaggtttcc
>c96abf4a6879a3e8569a1997a651beeb\_3
gctcctaccgattggatgagttggtgagtgaattggagtaacagctatctttctgaaagattgttgtatttcttaaaatttgcaaactagattatctagaggaaggagaagtcgtaacaaggtttcc
>ca01cbdea5e0b398d3ff5e71c2d64057\_3
gctcctaccgattggatgagttggtgagtgaattggagtaacagctatcttttgaaagattgttgtattttttaaaatttgcaaactagattatctagaggaaggagaagtcgtaacaaggtttcc
>cde8ddde750c629804e26e6e29e47d50\_3
gctcctaccgattggatgagttggtgagtgaattggagtaacagttatctttctgaaagattgttgtattttttaaaatttgcaaactagattatctagaggaaggagaagtcgtaacaaggtttcc
>d05e503a9e000d1d5fecbd3eee1ec3e8\_3
gctcctaccgattggatgagttggtgagtggattggagtaacagctatcttcctgtaagattgttgtaatttttttaaaatttgcaaactagattatctagaggaaggagaagtcgtaacaaggtttcc
>d1ac4fddc908d35553e614a2bc3f5566\_3
gctcctaccgattggatgagttggtgagtgaattggagtaacagctattttcctgaaagattgttatattttttaaaatttgcaaactagattatctagaggaaggagaagtcgtaacaaggtttcc
>d4a5eb808a77413a95f0ec46f3058004\_3
gctcctaccgattggatgagttggtgagtgaattggagtaacagcaatctttctgaaagattgttgtattttttaaaatttgcaaactagattatctagaggaaggagaagtcgtaacaaggtttcc
>d8e93191b8d485af554857d08175fe2f\_3
gctcctaccgattggatgagttggtgagtgaattggagtaacagctatctttctgaaagattgttgtattttttaaaatttgtaaactagattatctagaggaaggagaagtcgtaacaaggtttcc
>dc84d8e731bb5d979d03515d381f88b9\_3
gctcctaccgattggatgagttggtgagtgaattggagtaacaggtatgtttctgaaagattgttgtattttttaaaatttgcaaactagattatctagaggaaggagaagtcgtaacaaggtttcc
>dfbb0a4342c1cc80ef8f4e57ca0ac0b0\_3
gctcctaccgattggatgagttggtgagtgaattggagtaacagctatctttctgaaagattgttgtattttttcaaatttgcaaactagattatctagaggaaggagaagtcgtaacaaggtttcc
>e1a3b59b6ae65fa194012dd395b3347d\_3
gctcctaccgattggatgagttggtgagtgaattggagtaacaggtatctttctgaaagattgttgcattttttaaaatttgcaaaccagattatctagaggaaggagaagtcgtaacaaggtttcc
>e8df833b57ee38c8e064e08f414d507a\_3
gctcctaccgattggatgagttggtgagtgaattggagtaacagctatctttctgaaagattgttgtattttttaagatttgcaaactagattatctagaggaaggagaagtcgtaacaaggtttcc
>ed5233a827b07d098c08385f2827676c\_3
gctcctaccgattggatgagttggtgagtgaattggagtaacagctatctttctgaaagattgttgtatttttaaaatttgcaaactagattatctagaggaagtaaaagtcgtaacaaggtttcc
>fc4bbe0e5bc71dc0fec0653e516afed1\_3
gctcctaccgattggatgagttggtgagtgaattggagtaacagctatctttctgaaagattgttgtattttttaaaatttgcaaactggattatctagaggaaggagaagtcgtaacaaggtttcc
>0115caadd737e3b2ba0b9f2b835acaf7\_2
gctcctaccgattagatgagttggtgagtgaattggagtaacagctatctttctgaaagattgttgtattttttaaaatttgcaaactagattatctagaggaaggagaagtcgtaacaaggtttcc
>014a43ac12c18b77d120035bc97704f0\_2
gctcctaccgattggatgagttggtgagtgaattggagtaacaggtatctttctgaaagattgttgcattttttaaaatttgcaaaactagattatctagaggaaggagaagtcgtaacaaggttttcc
>03596c09f065813e09e8e0cfdcfcaea8\_2
gctcctaccgattggatgagttgttgagtgaattggagtaacagctatctttctgaaagattgttgtatttttaaaatttgcaaactagattatctagaggaaggagaagtcgtaacaaggtttcc
>03fa799baec3c8a58c7a2ad761850314\_2
gctcctaccgattggatgagttggtgagtgaattggagtaacagctacctttctgaaagattgttgtattttttaaaatttgcaaactagattatctagaggaaggagaagtcgtaacaaggtttcc
>055244ababdeecdd2d54f202de6252ad\_2
actcctaccgattggatgagttggtgagtgaattggagtaacagctatctttctgaaagattgttgttattttttaaaatttgcaaactagattatctagaggaaggagaagtcgtaacaaggtttcc
>06e6a0fbb6b603f2302d6a3d456b94d3\_2
gctcctaccgattggatgagttggtgagtgaattggagtaacaggtatctttctgaaagattgttgtattttttacaatttgcaaactagattatctagaggaaggagaagtcgtaacaaggtttcc
>07a33425c98d1a2bc4146c19a2be4f4d\_2
gctcctaccgattggatgagttggtgagtgaattggagtaacagctatctttctgaaagattgtagtattttttaaaatttgcaaactagattatctagaggaaggagaagtcgtaacaaggtttcc
>0808cea7ea4496d51a5d6e58d8185a95\_2
gctcctaccgattggatgagttggtgagtgaattggagtaacagctatctttctgaaagattgttgtattttttaaaattgcaaactagattatctagaggaaggagaagtcgtaacaaggttttcc
>09888961384d1ad17f7e153fcb1459d4\_2
gctcctaccgatcggatgagttggtgagtgaattggagtaacagctatctttctgaaagattgttgtattttttaaaatttgcaaactagattatctagaggaaggagaagtcgtaacaaggtttcc
>0c51112aeec05aeb3986b83a23f5bebc\_2
gctcctaccgattggatgagttggtgagtgaattggagtaacagctatctttctgaaagattgttgtatttttaaaaatttgcaaactagagtatctagaggaaggagaagtcgtaacaaggtttcc
>0c71dd4a91879421edd46d04b141548c\_2
gctcctaccgattggatgagttggtgagtgaattggagtaacagctatatttctgaaagattgttgtatttttaaaatttgcaaactagattatctagaggaaggagaagtcgtaacaaggtttcc
>0d8ccc533f0974588696f82d06e55954\_2
gcttctaccgattggatgagttggtgagtgaattggagtaacagctatctttctgaaagattgttgttattttttaaaatttgcaaactagattatctagaggaaggagaagtcgtaacaaggtttcc
>0e265b7a266454be8f03fa26e51cdcd3\_2
gctcctaccgattggatgagttggtgagtgaattggagtaacaggtatctttctgaaagattgttgcattttttaaaattttgcaaactagattatctagaggaaggagaagtcgtaacaaggttttcc
>0f1316ff5a7b2a44eae7cb11444c408f\_2
gcttctaccgattggatgagttggtgagtgaattggagtaacaggtatctttctgaaagattgttgtatttttaaaatttgcaaactagattatctagaggaaggagaagtcgtaacaaggtttcc
>104124d093ca036ccb2740d728aff69e\_2
gctcctaccgattggatgagttggtgagtgaattggagtaacagctatcttcctgaaagattgttatatttttttaaaatttgcaaactagattatctagaggaaggagaagtcgtaacaaggtttcc
>10c3314693266e41f3042bfe84505df0\_2
gctcctaccgattggatgagttggtgagtgaattggagtaacaggtatctttctgaaagattgttgtattttttaaaatttgcaaactagattatctagaggaaggagaagtcgtaagaaggtttcc
>11bc8d34c669dbf9932a64d446636cee\_2
gctcctaccgattggatgagttggtgagtgaattggagtaacagctatctttctgaaagattgttgtattttttaaaatttgcaaactagattatctagagggaggagaagtcgtaacaaggtttcc
>11f13e33b72a9f5c93440707befa390b\_2
actcctaccgattggatgagttggtgagtgaattggagtaacagctatctttctgaaaggattgttgtattttttaaaatttgcaaactagattatctagaggaaggagaagtcgtaacaaggtttcc
>11fbedd5821b7cc45fbfbc181c20bb9d\_2
gctcctaccgattggatgagttggtgagtgaattggagtaacagctatctttttgaaagattgttgtattttttaaaatttgcaaactagattatctagaggaaggagaagtcgtaacaaggtttcc
>128c0f36d9ae0c22b098fd56ceeda0c9\_2
gctcctaccgattggatgagttggtgagtgaattggagtaacagctatctttctgaaaagattgttgtattttttaaaaatttgcaaactagattatctagaggaaggagaagtcgtaacaaggtttcc
>13949f6f40d661ce9faf593817568ae3\_2
gctcctaccgattggatgagttggtgagtgaattggagtaacagctatctttctgaaagattgttgtattttttaaaatttgcaaactagattatctacaggaaggagaagtcgtaacaaggtttcc
>1433dfb032c96f7a1e322a471d4f3928\_2
gctcctaccaattggatgagttggtgagtgaattggagtaacagctatctttctgaaagattgttgtattttttaaaatttgcaaactagattatctagaggaaggagaagtcgtaacaaggtttcc
>16819416e2e8d227d712f5877cf022a2\_2
gctcctaccgattggataagttggtgagtgaattggagtaacaggtatctttctgaaagattgttgcattttttaaaatttgcaaactagattatctagaggaaggagaagtcgtaacaaggtttcc
>174da876d3826cadb5b33794676056d1\_2
gctcctaccgattggatgagttggtgagtggattggagtaacagctatcttcctgtaagattgttgtaattttaaaatttgcaaactagattatctagaggaaggagaagtcgtaacaaggtttcc
>18f05c66ea2da748d201337833e07eec\_2
gctcctaccgattggatgatttggtgagtgaattggagtaacagctatctttctgaaagattgttgttattttttaaaatttgcaaactagattatctagaggaaggagaagtcgtaacaaggtttcc
>199d66f18b7db7b5a1de7a0b1b865ce7\_2
gctcctaccgattggatgagttggtgagtggattggagtagcagctatcttcctgtaagattgttgtatttttaaaatttgcaaactagattatctagaggaaggagaagtcgtaacaaggtttcc
>1b5518d0a3c283cbc227b0d709e20dd2\_2
gctcctaccgattggatgagttggtgagtgaattggagtaacaggtatatttctgaaagattgttgtatttttaaaatttgcaaactagattatctagaggaaggagaagtcgtaacaaggtttcc
>1bc138fb5ef906a1057bf7b1824d1bfd\_2
gctcctaccgattggatgagttggtgagtgaattggagtaacagctatctttctgaaagattgttgtattttttaaaatttgcaaactagattgtctagaggaaggagaagtcgtaacaaggtttcc
>1c1b62d2a27301be698b532f919e2cfb\_2
gctcctaccgattggatgagttggtgagtgaattggagtaacaggtatctttctgaaagattgttgcattttttaaaatttagcaaactagattatctagaggaaggagaagtcgtaacaaggtttcc
>1c21c535260710e456690461623072e5\_2
gctcctaccgattggatgagttggtgagtgaattggagtaacagctatctttctgaaagattgttgtattttttaaaatttgcaaactagattatctagaggaaggagaagtcgtaacaaggcttcc
>1d3cf2374eb2610a98c80be36a395bf8\_2
gctcctaccgattggatgagttggtgagtgaattggagtaacagctatctctctgaaagattgttgtattttttaaaatttgcaaactagattatctagaggaaggagaagtcgtaacaaggtttcc
>1e0a7bd99e2912dcc87183f06387bcf3\_2
gctcctaccgtttggatgagttggtgagtgaattggagtaacagctatctttctgaaagattgttgtattttttaaaatttgcaaactagattatctagaggaaggagaagtcgtaacaaggtttcc
>1fcf089612b5cfd5802b4a5de5315ce4\_2
gctcctaccgatttgatgagttggtgagtgaattggagtaacagctatctttctgaaagattgttgtattttttaaaatttgcaaactagattatctagaggaaggagaagtcgtaacaaggtttcc
>2033a78dc4b173bd5c3571fcb27b5f98\_2
gctcctaccgattggatgagttggtgagtgaattggagtaacagctatctttctgaaagattgttgtattttttaaaatttgcaaactagattatctagaggaaggagaagtcgtaacaaggtttca
>20da99884dd7c271a8a82087629620c2\_2
gctcctaccgattggatgagttggtgagtgaattggagtaacagctatctttctgaaagattgttgtattttttaaaatttgcaaactagattatctggaggaaggagaagtcgtaacaaggtttcc
>226552340026cad6abc50967c1e6ce0c\_2
gctcctaccgacttggatgagttggtgagtgaattggagtaacagctatctttctgaaagattgttgtattttttaaaatttgcaaactagattatctagaggaaggagaagtcgtaacaaggtttcc
>233938a6bbe97931d48edf6d47665029\_2
gctcctaccgattggatgagttggtgagtggattggagtaacagctatcttcctgtaagattgttgtatttttaaaatttgcaaactagattatctagaggaaggagaagtcgtaacaaggtttcc
>23b0c0dafe31520d9e204548f1fc28b4\_2
gctcctaccgattggatgagttggtgagtgaattggagtaacagctatctttctgaaagattgttgtattttttaaaatttgcaaactagattatctagaggaaggggaagtcgtaacaaggtttcc
>2454f270eaca19b4505016c7b9dea389\_2
gctcctaccgattggatgagttggtgagtgaattggagtaacaggtatctttctgaaagattgttgcattttttaaaatttgacaaactagattatctagaggaaggaagaagtcgtaacaaggtttcc
>260c732490588a9d7f77556b6a23ec6b\_2
gctcctaccgattggatgagttggtgagtgaattggagtaacagctatctttctgaaagattgttgtattttttaaaatttgcaaactagattatctagaggagggagaagtcgtaacaaggtttcc
>27352e921c3e5206ab7e516505f8b55c\_2
gctcctaccgattggatgagttggtgagtgaattggagtaacagctatctttctgaaagattgttgtgtttttaaaaatttgcaaactagattatctagaggaaggagaagtcgtaacaaggtttcc
>275c407abd5dc6d3f020bd21c8e93dab\_2
gctcctaccgattggatgagttggtgagtgaattggagtaacagctatctttctgaaagattgttgttattttttaaaatttgcaaactagattatctagaagaaggagaagtcgtaacaaggtttcc
>2cb97c263f261e95478808ac1b8fc9a7\_2
gctcctaccgattggatgagttggtgagtgaattggagtaacaggtatctttctagaaagattgttgcattttttaaaatttgcaaactagattatctagaggaaggagaagtcgtaacaaggtttcc
>2f1def98db510f03dfea9f7ec6c54559\_2
gctcctaccgattggatgaggtggtgagtgaattggagtaacagctatctttctgaaagattgttgtattttttaaaatttgcaaactagattatctagaggaaggagaagtcgtaacaaggtttcc
>323d93b2cd43598aef63324aecb74777\_2
gctcctgccgattggatgagttggtgagtgaattggagtaacagctatctttctgaaagattgttgtattttttaaaatttgcaaactagattatctagaggaaggagaagtcgtaacaaggtttcc
>344791b747d049fff17f201b6e21cd8f\_2
gctcctaccgattggatgagttggtgagtgaattggagtaacaggtatctttctgaaagattgttgcattttttaaaatttgcaaacaagattatctagaggaaggagaagtcgtaacaaggtttcc
>345321f25eadd97221b75d4c2ac2dbc5\_2
gctcctaccgattggatgagttggtgagtgaattggagtaacagctatctttctgatagattgttgtattttttaaaatttgcaaactagattatctagaggaaggagaagtcgtaacaaggtttcc
>3497ac6f84da174e3127ee02b2be0e36\_2
gctcctaccgattggatgagttggtgagtgaattggagtaacaggtatctttctgaaagattgttgctatttttttaaaatttgcaaactagattatctagaggaaggagaagtcgtaacaaggttttcc
>34e3996db7008c3998f6ba4b124d6089\_2
gctcctaccgattggatgagttggtgagtgaattggagtaacaggtatctttctgaaagattgttgcatttttaaaaattttgcaaaactagattatctagaggaaggagaagtcgtaacaaggtttcc
>35d209dd6455a4af6884a2ce882b5f38\_2
gctcctaccgattggatgagttggtgagtgaattggagtaacaggtatctttctgaaagattgttgcatttttaaaatttgcaaactagattatctagaggaaggaaaagtcgtaacaaggtttcc
>365aea924457b7adb1f15fae6269a868\_2
gctcctaccgattggatgagttggtgagtgaattggagtaacagctatctttctgagagattgttgtattttttaaaatttgcaaactagattatctagaggaaggagaagtcgtaacaaggtttcc
>39ae1f68d7e6d3de806066ed0e82c39c\_2
gctcctaccgattggatgagttggtgagtgaattggagtaacagctatctttctgaaagattgttgtattttttaaaatttgcaaactagattatctagaggaaggagaagtcgtagcaaggtttcc
>3c219e552b7749c7b00863643be5d530\_2
gctcctaccgattggatgagttggtgagtgaattggagtaacagctatctttctgaaagattgttgtattttttaaagtttgcaaactagattatctagaggaaggagaagtcgtaacaaggtttcc
>3c6747029fa7093fac0173e249d11a5f\_2
gctcctaccgattggatgagttggtgagtggattggagtaacagctatcttcctgaaagattgttgtattttttaaaatttgcaaactagattatctagaggaaggagaagtcgtaacaaggtttcc
>3d0a0accfcc68987ae0902404419df50\_2
gctcctaccgattggatgagttggtgagtgaattggagtaacaggtatctttctgaaagattgttgtattttttaaaatttgcaaactagattatctagaggaaggagaagtcataacaaggtttcc
>3d1d9a35f3252e6c863f024318721c2b\_2
gctcctaccgattggatgagttggtgagtgaattggagtaacagctattttcctgaaagattgttatatttttttaaaatttgcaaactagattatctagaggaaggagaagtcgtaacaaggtttcc
>4098627cd4e60beac8d0e8a14db259ef\_2
gctcctaccgattggatgagttggtgagtgaattggagtaacagctatctttctgaaagattgttgtattttttaaaatttgcaaactagattatctagaggaaggagaaggcgtaacaaggtttcc
>416e053c58e31d0c9758088139c772c7\_2
gctcctaccgattggatgaattggtgagtgaattggagtaacaggtatctttctgaaagattgttgcattttttaaaatttgcaaactagattatctagaggaaggagaagtcgtaacaaggtttcc
>41f09e9b44ed6f0c2311682043fa8fb3\_2
gctcctaccgattggatgagttggtgagtgaattggagtaacagctatctttctgaaagattgttgtattttttaaaatttgcaaactagattatctagaggaaggagaagtcataacaaggtttcc
>443a4f18baf15f9291e2d9c82a889197\_2
cctcctaccgattggatgagttggtgagtgaattggagtaacagctatctttctgaaagattgttgtattttttaaaatttgcaaactagattatctagaggaaggagaagtcgtaacaaggtttcc
>44fefaf71cb5b55a5fea605ab94c309f\_2
gctctaccgattggatgagttggtgagtgaattggagtaacaggtatctttctgaaagattgttgcattttttaaaatttgcaaactagattatctagaggaaggagaagtcgtaacaaggtttcc
>4d6cc15b7efaf488bc2bf3e68da12f69\_2
gctcctaccgattggatgagttggtgagtgaattggagtaacgggtatctttctgaaagattgttgcattttttaaaatttgcaaactagattatctagaggaaggagaagtcgtaacaaggtttcc
>4ede74e4d5cab1227c77001dfae3b70b\_2
gctcctaccgattggatgagttggtgaatgaattggagtaacaggtatctttctgaaagattgttgtattttttaaaatttgcaaactagattatctagaggaaggagaagtcgtaacaaggtttcc
>4f6dcb2622d3dc17a6b757f0c441aeb8\_2
gctcctaccgattggatgagttggtgagtgaattggagtaatagctatctttctgaaagattgttgttattttttaaaatttgcaaactagattatctagaggaaggagaagtcgtaacaaggtttcc
>5158c7a814c68eeb92413507e90ba477\_2
gctcctaccgattggatgagttggtgagtgaattggagtaacagctatctttctgaaagattgttgtatttttaaaaatttgcaaactagattatctagaggaaggagaagtcgtaacaaagtttcc
>518452b1a3d4a3d106112aa56a832150\_2
gctcctaccgattggatgagttggtgagtgaattggagtaacaggtatctttctgaaaagattgttgtattttaaaatttgcaaactagattatctagaggaaggagaagtcgtaacaaggtttcc
>525493b2ab4c0a43194cc9afe5b75245\_2
gctcctaccgattggatgagttggtgagtgaattggagtaacaggtatctttctgaaagattgttgcattttttaaaattttagcaaactagattatctagaggaaggagaagtcgtaacaaggtttcc
>5260cf3d8e7c849c472415b6d353adee\_2
gctcctaccgattggatgagttggtgagtgaattggagtaacatgtatctttctgaaagattgttgtattttttaaaatttgcaaactagattatctagaggaaggagaagtcgtaacaaggtttcc
>540bdb9b36682184fc5ac38efc3e579a\_2
gctcctaccgattggatgagttggtgagtgtaattggagtaacaggtatctttctgaaagattgttgcattttttaaaatttgcaaactagattatctagaggaaggagaagtcgtaacaaggtttcc
>543eaeea24999226e55cb9a59ff1ce6d\_2
gctcctaccgattggatgagttggtgagtgaattggagtaacagctacctttctgaaagattgttgtatttttaaaatttgcaaactagattatctagaggaaggagaagtcgtaacaaggtttcc
>55cd99f938e949220700598d7ba753e4\_2
gctcctaccgattggatgagttggtgagtgaattggagtaacaggtatctttctgaaagattgttgtattttttaaaatttagcaaactagattatctagaggaaggagaagtcgtaacaaggtttcc
>55eebb938fe5453da998e7760753454d\_2
gctcctaccgattggatgagttggtgagtgaattggagtaacagctatctttctgaaagattgttgtattttttaaaatttgcaaactagattatatagaggaaggagaagtcgtaacaaggtttcc
>5785b800f20c2e2fa233f92a581d538a\_2
gctcctaccgattggatgagttggtgagtgaattggagtaacaggtatcgtttctgaaagattgttgtattttttaaaatttgcaaactagattatctagaggaaggagaagtcgtaacaaggtttcc
>579d51c75338d751b6826adcadb85be4\_2
gctcctaccgattggatgagttggtgagtgaattggagtaacaggtatctttctgaaaaattgttgcatttttaaaatttgcaaactagattatctagaggaaggagaagtcgtaacaaggtttcc
>5c81db6eff176887934a737a6e19839f\_2
gctcctaccgattggatgagttggtgagtgaattggagtaacaggtatctttctgaaagattgttgcattttttaaaatttgcaaactagattatctagaggaaggaaaagtcgtaacaaggtttcc
>5fa8fc8f965a9ef13ddde254f63e3dc3\_2
gctcctaccgattggatgagttggttgagtgaattggagtaacagctatctttctgaaagattgttgtatttttaaaatttgcaaactagattatctagaggaaggagaagtcgtaacaaggtttcc
>6072db4ceeddb085140a8a5a6fd7be92\_2
gctcctaccgattggatgagttggtgactgaattggagtaacagctatctttctgaaagattgttgtattttttaaaatttgcaaactagattatctagaggaaggagaagtcgtaacaaggtttcc
>61c995ca21f3339e77250a092d2adf56\_2
gctcctaccgattggatgagttggtgagagaattggagtaacagctatctttctgaaagattgttgtattttttaaaatttgcaaactagattatctagaggaaggagaagtcgtaacaaggtttcc
>62bc95fce9a5dbd0ddfa14cc7a6a7d92\_2
gctcctaccgattggatgagttggttgagtgaattggagtaacagctatctttctgaaagattgttgtattttttaaaatttgcaaactagattatctagaggaaggagaagtcgtaacaaggtttcc
>63491fd0c774f3d0d2264d968a42bc10\_2
gctcctaccgattggatgagttggtgagtgaattggagtaacagctatctttctgaaagattgttgtattttttaaaatttgcaaaccagattatctagaggaaggagaagtcgtaacaaggtttcc
>6722606637d3147de898702dc9c84d9d\_2
gctcctaccgattggatgagttggtgagttaattggagtaacaggtatctttctgaaagattgttgtattttttaaaatttgcaaactagattatctagaggaaggagaagtcgtaacaaggtttcc
>68e15604e4c55d34d79f6ffe6dc828cb\_2
gctcctaccgattggatgagttggtgagtgaattggagtaacaggtatctttctgaaagattgttgcattttttaaaatttgcaaactagattatctagaggaaggagaagtcataacaaggtttcc
>6a138ed626a29cb601bb0d3d017158f7\_2
gctcctaccgattggatgagttggtgagtgaattggagtaacagctatctttctgaaagattgttgtattttttaaaatttgcaaactagattatctagaggaatgagaagtcgtaacaaggtttcc
>6ad137dee14be05402009f9b030b00f2\_2
gctcctaccgattggatgagttggtgagtgaattggagtaacagctatctttctgaaagattgttgtattttttaaaatttgcaaactagattatctagaggaaggagaagtcgtaacaaggttcc
>6bd558c9f199578c5df35c415dff7cc1\_2
gctcctaccgattggatgagttggtgagtgaattggagtaacaggtatctttctgaaagattgttgtattttttaaaatttgcaaactatattatctagaggaaggagaagtcgtaacaaggtttcc
>6e8d3f322c09ed8d961a0c1c1f8d2fe7\_2
gctcctaccgattggatgagttggtgagtgaattggagtaacagctatctttctgaaagattgttgttattttttaaaatttgcaaactagattatctagaggaaggagaagtcgtaactaaggtttcc
>6f6103dcfb099e523593af2ff43812b6\_2
gctcctaccgattggatgagttggtgagtgaattggagtaacaggtatctttctgaaagattgttgcatttttttaaaaattgcaaactagattatctagaggaaggagaagtcgtaacaaggtttcc
>704ee9535189d4714d1bcf3ad0e75fe2\_2
gctcctaccgattggatgagtaggtgagtgaattggagtaacaggtatctttctgaaagattgttgcattttttaaaatttgcaaactagattatctagaggaaggagaagtcgtaacaaggtttcc
>711b3f94290123b04f8426ab69fa6f2c\_2
gctcctaccgattggatgagttggtgagtgaattggagtaacaggtatctttctgaaagattgttgcattttttaaaatttgcaaactagattatctagaggaaggagaagtcgtaacaaggattcc
>71775b199fbdc91bd3064f59eff68366\_2
gctcctaccgattggatgagttggtgagtgaattggagtaacagctatctttctgaaagattgttgtattttttaaaatttgcaaactagattatctagaggaaggagaattcgtaacaaggtttcc
>718bc0f5f8862beb17db6963d7e70d31\_2
gctcctaccgattggatgagttgctgagtgaattggagtaacagctatctttctgaaagattgttgtattttttaaaatttgcaaactagattatctagaggaaggagaagtcgtaacaaggtttcc
>718d857faef54703e777b996f5512f44\_2
gctcctaccgattggatgagttggtgagtgaattggagtaacagctatctttctgaaagattgttgtattttttaaaaatttgcaaaactagattatctagaggaaggagaagtcgtaacaaggtttcc
>748cdd810b99c06ed9df2e79d574ac47\_2
gctcctaccgattggatgagttggtgagtgaattggagtaacagctatctttctgaaagattgttgtattttttaaaatttgcaaactagattatctagaggaaggaaaaagtcgtaacaaggtttcc
>783740154a3f943f231ba5b9c63d881b\_2
gctcctaccgattggatgagttggtgagtgaattggagtaacagctatctttctgaaagattgttgaatttttaaaatttgcaaactagattatctagaggaaggagaagtcgtaacaaggtttcc
>79ba3d9e531d459aab07b7990b434b51\_2
gctcctaacgattggatgagttggtgagtgaattggagtaacagctatctttctgaaagattgttgtattttttaaaatttgcaaactagattatctagaggaaggagaagtcgtaacaaggtttcc
>7a4b7352f50d8c766401e81c1ee5dd3c\_2
gctcctaccgattggatgagttggtaagtgaattggagtaacagctatctttctgaaagattgttgtattttttaaaatttgcaaactagattatctagaggaaggagaagtcgtaacaaggtttcc
>7a9fffb4c00e5990cf9871dcd43c409e\_2
gctcctaccgattggatgagttggtgagtgaattggagtaacaggtatctttctgaaagattgttgcatttttaaaatttgcaaactagattatctaaaggaaggagaagtcgtaacaaggtttcc
>7d2f23d051b10edba3c1e28a83fb4ea4\_2
gctcctaccgattggatgagttggtgagtgaattggagtaacagctatctttctgaaagattgttgtattttttaaaatttgcaaactagattatctagagaaaggagaagtcgtaacaaggtttcc
>7eff17fd17a71b469b1c86d5e4847d31\_2
gctcctaccgattggatgagttggtgagtgaattggagtaacaggtatctttctgaaagattgttgcattttttaaaatttgcaaactagattatctagaggaaggagaagtcgtaacatggtttcc
>7f96baa19c5f013220f497494652c976\_2
gctcctaccgattggatgagttggtgagtgaattggagtaacaggtatctttctgaaagattgttgcattttttaaaatttgcaagctagattatctagaggaaggagaagtcgtaacaaggtttcc
>7fdd0fcbbc8da80cc674f854a692fe2d\_2
gctcctaccgattggatgagttggtgagtgaattggagtaacagctatctttctgaaagattgttgttaatttttaaaaatttgacaaactagattatctagaggaaggagaagtcgtaacaaggtttcc
>82a12153bd0b0e28c5443f16d181fe33\_2
gctcctaccgattggatgagttggtgagtgaattggagtaacaggtatctttctgaaagattgttgtattttttaaaatttgcaaactagactatctagaggaaggagaagtcgtaacaaggtttcc
>869e764e22f63d91d1e19967c71136ba\_2
gctcctaccgattggatgagttggagagtgaattggagtaacaggtatctttctgaaagattgttgcattttttaaaatttgcaaactagattatctagaggaaggagaagtcgtaacaaggtttcc
>87249192ea1e15e2a8d61fd2ca7d494a\_2
gctcctaccgattggatgagttggtgagtgaattggagtaacaggtatgtttctgaaagattgttgcattttttaaaatttgcaaactagattatctagaggaaggagaagtcgtaacaaggtttcc
>881836e7ec6bd155064d7b54be327c23\_2
gctcctaccgattggatgagttggtgagtgaattggagtaacaggtatctttctgaaagattgctgcattttttaaaatttgcaaactagattatctagaggaaggagaagtcgtaacaaggtttcc
>8abce827b69353c7a800dc42ccf6337a\_2
gctcctaccgattggatgagttggtgagtgaattggagtaacagctatctttctgaaagattattgttattttttaaaatttgcaaactagattatctagaggaaggagaagtcgtaacaaggtttcc
>8e913e1897cec7f6aab4553f2fbb506d\_2
gctcctaccgattggatgagttggttagtgaattggagtaacagctatctttctgaaagattgttgtattttttaaaatttgcaaactagattatctagaggaaggagaagtcgtaacaaggtttcc
>8fa902788bba22f72b188474ce4cc872\_2
gctcctaccgattggatgagttggtgagtgaattggagtaacaggtatctttctgaaagattgttgcttttttaaatttgcaaactagattatctagaggaaggagaagtcgtaacaaggtttcc
>8fc2245355721a446ab882970f5b687a\_2
gctcctaccgattggatgagttggtgagtgaattggagtaactgctatctttctgaaagattgttgttattttttaaaatttgcaaactagattatctagaggaaggagaagtcgtaacaaggtttcc
>906cfd0c4a70e17c76bc6b65cbf5b7ea\_2
gctcctaccgattggatgagttggtgagtgaattggagtaacagctatctttctgaaagattgttgtaatttttaaaatttgcaaactagattatctagaggaaggagaagtcgtaacaaggtttcc
>91d101b9bbac22f982d7ad1a3acc2be3\_2
gctcctaccgattggatgagtcggtgagtgaattggagtaacaggtatctttctgaaagattgttgtattttttaaaatttgcaaactagattatctagaggaaggagaagtcgtaacaaggtttcc
>91fdb8fcb518c20f34337cd0162edd2c\_2
gctcctaccgattggatgagttggtgagtgaattggagtaacaggtatctttctgaaagattgttgcatttattaaaatttgcaaactagattatctagaggaaggagaagtcgtaacaaggtttcc
>939ab71e7956e5854fac475e139d8c32\_2
gctcctaccgattggatgagttggtgagtgaattggagtaacagctatctttctgaaagattgttgtattttttaaaatttgcagactagattatctagaggaaggagaagtcgtaacaaggtttcc
>97acaf44b26eccef916af5a1412f9596\_2
gctcctaccgattggatgagttggtgagtgaattggagtaacagctatctttctggaaagattgttgtattttttaaaatttgcaaactagattatctagaggaaggagaagtcgtaacaaggtttcc
>986f5e4505dfc71ba9dddde3b67f51d4\_2
gctcctaccgattggatgagttggtgagtgaattggagtaacaggtttctttctgaaagattgttgcattttttaaaatttgcaaactagattatctagaggaaggagaagtcgtaacaaggtttcc
>9968a007bae1f6250f43da7f6e9b1760\_2
gctcctaccgattggatgagttggtgagtgaattggagtaacaggtatctttctgaaagagttgttgcattttttaaaatttgcaaactagattatctagaggaaggagaagtcgtaacaaggtttcc
>99d0f339df904d19e0afc5c70d4edca3\_2
gctcctaccgattggatgagttggtgagtgaattggagtaacaggtatctttctgaaagattgttgcattttttaaaatttgcaaactagattatctagaggaaggagaagtcgtaacaaggtttca
>99f1d290e594dae9c18d5794f809724d\_2
gctcctaccgattggatgagttggtgagtgaattggagtaacagctatctttctgaaagactgttgtattttttaaaatttgcaaactagattatctagaggaaggagaagtcgtaacaaggtttcc
>9ac2f367a3eeb70099d864269b72ff16\_2
gctcctaccgattggatgagttggtgagtgaattggagtaacagctatctttctgaaagattgttgttattttttaaaatttgcaaactagattatctagaggaaggagaagtcgtaccaaggtttcc
>9bf96c2e5eaf89c87ab2c8243928a9eb\_2
gctcctaccgattggatgagttggtgagtgaattggagtaacagctatctttctgaaagattgttgttattttttaaaatttgcaaactagattatctagaggaaggagaagtcttaacaaggtttcc
>9c89f3020e8ea35c08fe55635d8e0af6\_2
gctcctaccgattggatgagttggtgagtgaattggagtaacagctatctttctgaaagattgttgtattttttaaaatttgcaaactagattatctaagggaaggagaagtcgtaacaaggtttcc
>9d63bbf1ea18f0f133c7d32f462228e5\_2
gctcctaccgattggatgagttggtgagtgaattggagtaacagctatctttctgaaagattgttgtattttttaaaatttgcaaactagattatctagaggaaggagaaagtcgtaacaaggtttcc
>a1319adebe0beca3b5da08d4638a08a9\_2
gctcctaccgattggatgagttggtgagtgaattggagtaacaggtatctttctgaaagattgttgcattttttaaaatttgcaaactagattatctagaggaagaagaagtcgtaacaaggtttcc
>a15e8a1468f6702d4a352c714ce94c52\_2
gctcctgccgattggatgaattggtgagtgaattggagtaacagctatctttctgaaagattgttgtattttttaaaatttgcaaactagattatctagaggaaggagaagtcgtaacaaggtttcc
>a252b2c56a721a509e85198dd63fcf6b\_2
gctcctaccgattggatgagttggtgagtgaattggagtaacagctatctttctgaaagatttttgtattttttaaaatttgcaaactagattatctagaggaaggagaagtcgtaacaaggtttcc
>a362661cf470c2e667d51926e1aae02d\_2
gctcctaccgattggatgagttggtgagtgaattggagtaacagctatctttctgaaagattgttgtttttttaaaatttgcaaactagattatctagaggaaggagaagtcgtaacaaggtttcc
>a5bc8bac5227f4f50ca421cffd4bfd8d\_2
gctcctaccgattggatgagttggtgagtgatttggagtaacagctatctttctgaaagattgttgtattttttaaaatttgcaaactagattatctagaggaaggagaagtcgtaacaaggtttcc
>a718d6e3e6489c38da713f8e39854d56\_2
gctcctaccgattggatgagttggtgagtgaattggagtaacaggtatctttctgaaagattgttgtatttttaaaattgcaaactagattatctagaggaaggagaagtcgtaacaaggtttcc
>ac2dd3f129685edbebde2350b6631904\_2
gctcctaccgattggatgagttggtgagtgaattggagtaacagctatctttctgaaagattgttgtatttgttaaaatttgcaaactagattatctagaggaaggagaagtcgtaacaaggtttcc
>ac594f35cb01356962df91897b2fd831\_2
gctcctaccgattggatgagttggtgagtgaattggagtaacagctatctttctgaaagattgttgtattttttaaaatttgcaaactagattatctagaggaaagagaagtcgtaacaaggtttcc
>ac667ddf2b446f25cb10708fde93c8b4\_2
gctcctaccgattggatgagttggtgagtgaattggagtaacagctatctttctgaaagattgttgtatttttcaaaatttgcaaactagattatctagaggaaggagaagtcgtaacaaggtttcc
>b1d513ad8407098a45a8e9aa9f62b103\_2
gctcctaccgattggatgagatggtgagtgaattggagtaacaggtatctttctgaaagattgttgcattttttaaaatttgcaaactagattatctagaggaaggagaagtcgtaacaaggtttcc
>b3a3915859182599d8e1777112eec22f\_2
gctcctaccgattggatgagttggtgagtgaattggagtaacaggtatctttctgaaagattgttgcatttttttaaaatttgcaaactagattatctagaggaaggagaagtcgtaacaaggtttca
>b3fbb2f54e4588a27bbc89dd9f5476a5\_2
gctcctaccgattggatgagttggtgagtgaattggagtaacagctatctttctgaaagattgttgtatttttaaaatttagcaaactagattatctagaggaaggagaagtcgtaacaaggtttcc
>b629e343da4d734a1916a94687b56a3a\_2
gctcctaccgattggatgagttggtgagtgaattggagtaacaggtatctttctgaaagattgttgtattttttaaaatttgcaaactagattatctagaggatggagaagtcgtaacaaggtttcc
>b8afe3ab8468404f6b7e5ea8440b225c\_2
actcctaccgattggatgagttggtgagtgaattggagtaacagctatctttctgaaagattgttgtatttttaaaatttgcaaactagattatctagaggaaggagaagtcgtaacaaggtttcc
>b99e450ae828384efc49493573f2f17c\_2
gctcctaccgattggatgagttggtgagtgaattggagtaacagctatctttctgaaagattgttgtattttttaaaatttgcaaactagattatctagaggaaggagaagtcgtatcaaggtttcc
>b9da0c715b3f635d5776479e9bddb406\_2
gctcctaccgattggatgagttggtgagtgaattggagtaacaggtatctttctgaaagattgttgcattttttaaaatttgcaaactagattatctagacgaaggagaagtcgtaacaaggtttcc
>ba06cbf4c1b07835be259eed9f773fd0\_2
gctcctaccgattggatgagttggtgagtgaattggagtaacaggtatctttctgaaagattgttgcattttttaaaatttgcaaactagattatctagaggaaggaggagtcgtaacaaggtttcc
>bb8ee8cb24b6acca1e5c41d0ceb8a4a9\_2
cctcctaccgattggatgagttggtgagtgaattggagtaacaggtatctttctgaaagattgttgcattttttaaaatttgcaaactagattatctagaggaaggagaagtcgtaacaaggtttcc
>bd905fecba5f37de521f5aa63885e2df\_2
gctcctaccgattggatgagttggtgagtgaattggagtaacagctatctttctgaaagattgttgtatttttaaaatttgcaaaactagattatctagaggaaggagaagtcgtaacaaggtttcc
>bf58a4414f81ea5bbf13b15d3db443a1\_2
gctcctacagattggatgagttggtgagtgaattggagtaacaggtatctttctgaaagattgttgcattttttaaaatttgcaaactagattatctagaggaaggagaagtcgtaacaaggtttcc
>c00b17bcf5752eb3e0c47b208c388bf1\_2
gctcctaccgattggatgagttggtgagtgaattggagtaacaggtatctttctgaaagattgttgtattttttaaaatttgcaaactagattatctaggggaaggagaagtcgtaacaaggtttcc
>c0836e8a154dc64847802f6ee2973ec8\_2
gctcctaccgattggatgagttggtgagtggattggagtaacagctatcttcctgtaagattgttgtattttttaaaattgcaaactagattatctagaggaaggagaagtcgtaacaaggtttcc
>c0a3224a5465e56b2162eee2c90a5802\_2
gctcctaccgattggatgagttggtgagtgaattggagtaacagctatctttctgaaagattgttgtattttttaaaatttgcatactagattatctagaggaaggagaagtcgtaacaaggtttcc
>c3efe06ba987f7fba3d6fb06bc1d4693\_2
gctcctaccgattggatgagttggtgagtgaattggagtaacagctattttctgaaagattgttgtattttttaaaatttgcaaactagattatctagaggaaggagaagtcgtaacaaggtttcc
>c45204660fbeb196a45a2745a3c229d1\_2
gctcctaccgattggatgagttggtgagtgaataggagtaacagctatctttctgaaagattgttgtattttttaaaatttgcaaactagattatctagaggaaggagaagtcgtaacaaggtttcc
>c5e8d7c42f17d42316822c6b94d65a46\_2
gctcctaccgattggatgagttggtgagtgaattggagtaacagctatctttctgaaagattgttgtattatttaaaatttgcaaactagattatctagaggaaggagaagtcgtaacaaggtttcc
>c7f7a4e137f961849aeb3c899b0e5f1b\_2
gctcctacctattggatgagttggtgagtgaattggagtaacaggtatctttctgaaagattgttgcattttttaaaatttgcaaactagattatctagaggaaggagaagtcgtaacaaggtttcc
>c9629e28204b7e4739ce5af57735c1e6\_2
gctcctaccgattggatgagttggtgagtgaattggagtaacaggtatctttctgaaagattgttgatttttaaaatttgcaaactagattatctagaggaaggagaagtcgtaacaaggtttcc
>ca747066c6eb1cdaeaa9544489290cf9\_2
gctcctaccgattggttgagttggtgagtgaattggagtaacaggtatctttctgaaagattgttgcattttttaaaatttgcaaactagattatctagaggaaggagaagtcgtaacaaggtttcc
>ce09ae476a551aa01ef923d6a28d5743\_2
gctcctaccgattggatgagttggtgagtgaattggagtaacaggtatctttctgaaagattgttgcatttttttaaaatttgcaaattagattatctagaggaaggagaagtcgtaacaaggtttcc
>ce8cc4aa9d2a1ef946f6e10845446411\_2
gctcctaccgattggatgagttggtgagtgaattggagtaacagctatctttctgaaagattgttgatttttaaaatttgcaaactagattatctagaggaaggagaagtcgtaacaaggtttcc
>cfb006cc597ff29d95f02bdbd514886a\_2
gctcctaccgattggatgagttggtgagtgaattggagtaacagctatctttctgaaagattgttgtattttttaaaatttgcaaactagattatctagaggaaggagaagtcgtaagaaggtttcc
>cffe0bad4c2aa3a01356c693a25951e2\_2
gctcctaccgattggatgagatggtgagtgaattggagtaacagctatctttctgaaagattgttgtattttttaaaatttgcaaactagattatctagaggaaggagaagtcgtaacaaggtttcc
>d31792978f25adad88da8efc1509ba9a\_2
gctcctaccgattggatgagttggtgagtgaattggagtaacaggtatctttctgaaagattgttgattttttaaaatttgcaaactagattatctagaggaaggagaagtcgtaacaaggtttcc
>d33c8bde077c649777237cc3659e35ce\_2
gctcctaccgattggatgagttggtgagtgaactggagtaacagctatctttctgaaagattgttgtattttttaaaatttgcaaactagattatctagaggaaggagaagtcgtaacaaggtttcc
>d3822dc194ce8b9f022746d158ba29bb\_2
gctcctaccgattggatgagttggtgagtgaattggagtaacagctatctttctgaaatattgttgttattttttaaaatttgcaaactagattatctagaggaaggagaagtcgtaacaaggtttcc
>d3a218e42e2b69b46b9b3967dc8484ab\_2
gctcctaccgattggatgagttggtgagtgaattggagtaacagctatctttctgaaagattgttttatttttaaaatttgcaaactagattatctagaggaaggagaagtcgtaacaaggtttcc
>d4f7f40e9225a8c682ca3a5b5ff9acab\_2
gctcctaccgattggatgagttggtgagtgaattggagtaacagctatctttctgaaagattgttgtattttttaaaatttgcaaactagattatctagaggaaggagaagtagtaacaaggtttcc
>d51e908b84d29db7fe76e87e4bfbe342\_2
gctccctaccgattggatgagttggtgagtgaattggagtaacagctatctttctgaaagattgttgtattttttaaaatttgcaaactagattatctagaggaaggagaagtcgtaacaaggtttcc
>d5a55edac4fa67ad2d0b7bf3b7af0982\_2
gctcctaccgattggatgagttggtgagtgaattggagtaacaggtatctttctgaaagattgttgcattttttaaatttgcaaactagattatctagaggaaggagaagtcgtaacaaggtttcc
>d6db9e800c8372874bd26c30fecd9ee5\_2
gctcctaccgattggatgagttgatgagtgaattggagtaacagctatctttctgaaagattgttgtattttttaaaatttgcaaactagattatctagaggaaggagaagtcgtaacaaggtttcc
>d6ef534963f26448cf352c7c24c09271\_2
gctcctaccgattggatgagttggtgagtgaattggagtaacagctatctttctgaaagattgttgtattttttaaaatttagcaaactagattatctagaggaaggagaagtcgtaacaaggtttcc
>d72db43e17a8ed630f208b0c8bf0633c\_2
gctcctaccgattggatgagttggtgagtgaattggagtaacaggtatctttctgaaagattgttgcattttttaaaatttgcaaactagattatctagaggaaggaagaagtcgtaacaaggttttcc
>d737477ad550bfe647a0b9d5b1b4d111\_2
gctcctaccgattggatgagttggtgagtgaattggagtaacaggtatctttctgaaagattgttgtattttttaaaatttgcaatctagattatctagaggaaggagaagtcgtaacaaggtttcc
>d7878a7be185abdd2de741f121bdf9d7\_2
gctcctaccgattggatgagttggtgagtgaattggagtaacagctatctttctgaaagattgttgtattttgtaaaatttgcaaactagattatctagaggaaggagaagtcgtaacaaggtttcc
>d9dcc68ec25f63608c6408586dacf219\_2
gctcctaccgattggatgagttggtgagtgaattggagtaactagctatctttctgaaagattgttgtattttttaaaatttgcaaactagattatctagaggaaggagaagtcgtaacaaggtttcc
>da8643ea8984423939cd0a2f4a738cd0\_2
gctcctaccgattggatgagttggtgagtgaattggagtaacaggtatctttctgaaagattgttgcatttttttaaatttgcaaactagattatctagaggaaggagaagtcgtaacaaggtttcc
>daac959611465df7565b32ec4700b49f\_2
gctcctaccgattggatgagttggtgagtgaattggagtaacagctatctttctgaaagattgttgtatttttaaaaatttagcaaactagattatctagaggaaggagaagtcgtaacaaggtttcc
>dbb266c6038b4a4843060895f27b0889\_2
gctcctaccgattggatgagttggtgagtgaattggagtaacagctatctttctgaaagattgttgtattttttaaaatttgaaaactagattatctagaggaaggagaagtcgtaacaaggtttcc
>ddff760a7ce7c3ca29d4e1426cae6eea\_2
gctcctaccgattggatgagttggtgagtgaattggagttaacaggtatctttctgaaagattgttgtattttttaaaatttgcaaactagattatctagaggaaggagaagtcgtaacaaggtttcc
>de9d06a70824b068d681094a98beeaaf\_2
gctcctaccgattggatgagttggtgagtgaattggagtaacaggtatatttctgaaagattgttgtattttttaaaatttgcaaactagattatctagaggaaggagaagtcgtaacaaggtttcc
>df0ba369545e4561c15aee88236f93b5\_2
gctcctacagattggatgagttggtgagtgaattggagtaacagctatctttctgaaagattgttgtattttttaaaatttgcaaactagattatctagaggaaggagaagtcgtaacaaggtttcc
>e1647235947d9c96091bf8b905e44e51\_2
gctcctaccgattggatgagttggtgagtgaattggagtaacagctatctttctgaaagattgttgtattttttaaaatttgcaaactaaattatgtagaggaaggagaagtcgtaacaaggtttcc
>e2d9b82d74de050f15662c3bfe993bd3\_2
gctcctaccgattggatgagttggtgagtgaattggagtaacagctatctttctgaaagattgttgtattttttagaatttgcaaactagattatctagaggaaggagaagtcgtaacaaggtttcc
>e49eccfe6df3d1d16db3f69040868ca9\_2
gctcctaccgattggatgagttggtgagtgaattggagtaacagctatctttctgaaaggttgttgtattttttaaaatttgcaaactagattatctagaggaaggagaagtcgtaacaaggtttcc
>e4fa05c8ff57ff7953584b23fe180575\_2
gctcctaccgattcgatgagttggtgagtgaattggagtaacaggtatctttctgaaagattgttgtattttttaaaatttgcaaactagattatctagaggaaggagaagtcgtaacaaggtttcc
>e552bc986a1e0122b2d1e9f62b2e590a\_2
gctcctaccgattggatgagttggtgagtgaattggagtaacaggtatctttctgaaagattgttgtattttttaaaatttgcaaactagattatctagaggaaggagaagtcgtaacaagatttcc
>e6b108c38f4992473d4c914f98616ef1\_2
gctcctaccgattggatgagttggtgagtgaattggagtaacaggtatctttctgaaagattgttgtattttttaaaatttgcaaactagattatctagaggaaggagaggtcgtaacaaggtttcc
>ec074d3a2f80c07dbf69deacd9abf2b1\_2
gctcctaccgattggatgagttggtgagtgaatggagtaacaggtatctttctgaaagattgttgcattttttaaaatttgcaaactagattatctagaggaaggagaagtcgtaacaaggtttcc
>ee3d718fb1825374141c19e9f57db34d\_2
actcctaccgattggatgagttggtgagtgaattggagtaacaggtatctttctgaaagattgttgcattttttaaaatttgcaaactagattatctagaggaaggagaagtcgtaacaaggttttcc
>ee818dd00c5aeeee524581a83670299a\_2
gcacctaccgattggatgagttggtgagtgaattggagtaacagctatctttctgaaagattgttgtattttttaaaatttgcaaactagattatctagaggaaggagaagtcgtaacaaggtttcc
>ef3b839b072bf594f76a8056c2bdb69f\_2
gctcctaccgattggatgagttggtgagtgaattggagtaacaggtatctttctgaaagattgttgcattttttaaaatttgcaaactagattatctagaggaaggagatgtcgtaacaaggtttcc
>f0bbef7508256681cbc955492d714c2c\_2
gctcctaccgattggatgagttggtgagtgaattggagtaacagctatctttctgaaagattgatgtattttttaaaatttgcaaactagattatctagaggaaggagaagtcgtaacaaggtttcc
>f4481f1e97c3e9ab5741edab0e77a633\_2
gctcctaccgattggatgagttggtgagtgaattggagtaacagctatctttctgaaagattgttgtatttttaaaattttgcaaactagattatctagaggaaggagaagtcgtaacaaggtttcc
>f517602926051e69428f37b2fb04b767\_2
gctcctaccgattggatgagttggtgagtgaattggagtaacaggtatctttctgaaagattgttgcattttttaaaatttacaaactagattatctagaggaaggagaagtcgtaacaaggtttcc
>f68f29e0142639502266b880f1f44db9\_2
gctcttaccgattggatgagttggtgagtgaattggagtaacagctatctttctgaaagattgttgtattttttaaaatttgcaaactagattatctagaggaaggagaagtcgtaacaaggtttcc
>f82a7e42f37e52e2d3f95ec6e47bccd4\_2
gctcctaccgattggatgagttggtgagtgaattagagtaacaggtatctttctgaaagattgttgcattttttaaaatttgcaaactagattatctagaggaaggagaagtcgtaacaaggtttcc
>f853d810ea7527478de125ca884adeca\_2
gctcctaccgattggatgagttggtgagtggattggagtaacagctatcttcctgtaagattgttgtattttttaaaatttgcaaactagattatctagaggaaggagaagttgtaacaaggtttcc
>f89a2c228a34daba300ae2806c85f157\_2
gctcctaccgattggatgagttggtgagtgaattggagtaacagctatctttctgaaagattgttgtattttttaaaatttgcaaactagaatatctagaggaaggagaagtcgtaacaaggtttcc
>f9437dac851e8ac7b02345553689bdc6\_2
gctcctaccgattggatgagttggtgagtgaattggagtaacagctatctttctgaaagattgttgtattttttaaaatttgcaaactagagtatctagaggaaggagaagtcgtaacaaggtttcc
>fca558d8d7ef6d95806c5b755563592b\_2
gctcctaccgattggatgagttggtgagtgaattggagtaacagatatctttctgaaagattgttgcattttttaaaatttgcaaactagattatctagaggaaggagaagtcgtaacaaggtttcc
>fcc01c8fdba15bf388c1deaa99a32973\_2
gctactaccgattggatgagttggtgagtgaattggagtaacagctatctttctgaaagattgttgttattttttaaaatttgcaaactagattatctagaggaaggagaagtcgtaacaaggtttcc
>fd93f8fd0b8c4b11d29cd51177522e92\_2
gctcctaccgattggatgagttggtgagtgaattggagtaacaggtatctttctgaaagattgttgcattttttaaaatttgcaaactagattatttagaggaaggagaagtcgtaacaaggtttcc
>fe326a5d97deff74b2b4d1bc53d28660\_2
gctcctaccgattagatgagttggtgagtgaattggagtaacaggtatctttctgaaagattgttgtattttttaaaatttgcaaactagattatctagaggaaggagaagtcgtaacaaggtttcc
>fefdbc3a79f07afe3590339186904ee1\_2
gctcctaccgattggatgagttggtgagtgaattggagtaacaggtatctttctgaaagattgttgcatttttaaaaatttgcaaactagattatctagaggaaggagaagtcgtaacaaggtttcc
>0002fd9c3239775adc9a35f915021e8e\_1
gctcctaccgattggatgagttggtgtagtgaattggagtaactagctatctttctgaaagattgttgtattttttaaaatttgcaaactagattatctagaggaaggagaagtcgtaacaaggtttcc
>006639f982681bc50e57a012ccf1d1d7\_1
gctcctaccgattggatgagttggtgagtgaattggagtaaaagctatctttctgaaagattgttgtattttttaaaatttgcaaactagattatctagaggaaggagaagtcgtaacaaggtttcc
>015d983565287e3eff88cfde03a0934d\_1
gctcctaccgattggatgagttggtgagtgaattggagtaacaagtatctttctgaaagattgttgtattttttaaaatttgcaaactagattatctagaggaaggagaagtcgtaacaaggtttcc
>01683ef11c6564b1fbe85397fd91b54d\_1
gctcctaccgattgtatgagttggtgagtggattggagtagcagctatcttcctgtaagattgttgtattttttaaaatttgcaaactagattatctagaggaaggagaagtcgtaacaaggtttcc
>021688579e1b2107e3d71640ae5351c2\_1
gctcctaccgattggatgagttggtgagtgaattggagtaacaggtatctttctgaaagattgttgcattttttcaaatttgcaaactagattatctagaggaaggagaagtcgtaacaaggtttcc
>02d510b19f824140afe7a306f0b1df5d\_1
gctcctaccgattggatgagttggtgagtgaattggagtaaccaggtatctttctgaaagattgttgcattttttaaaatttgcaaactagattatctagaggaaggagaagtcgtaacaaggtttcc
>035c34f16818cf73d61efeb8130dc755\_1
gctcctaccgattgggtgagttggtgagtgaattggagtaacagctatctttctgaaagattgttgtattttaaaatttgcaaactagattatctagaggaaggagaagtcgtaacaaggtttcc
>04d3d194f2117be64823c8c4b3dd9031\_1
gctcctaccgattggatgagttggtgagtgaattggagtaacagctatctttctgaaagattgttgttattttttaaaatttgcaaactagattatctagaggaaggagaagtcctaacaaggtttcc
>04f15d32cf633f25169b5ed938134130\_1
gctcctaccgattggatgagttggtgagtgaattagagtaacagctatctttctgaaagattgttgtatttttaaaatttgcaaactagattatctagaggaaggagaagtcgtaacaagatttcc
>05ca95b4939e9d724901798f8a701ce0\_1
gctcctaccgattggatgagttggtgagtgaattggagtaacagctatctttctggaagattgttgttattttttaaaatttgcaaactagattatctagaggaaggagaagtcgtaacaaggtttcc
>05f11426d945287ac1a3cc5f00c043bd\_1
gctcctaccgattggatgagttggtgagtgaattggagtaacaggtatctttctgaaagattgttgtattttttaaaatttgcaaactagattatctagaggaaggacaagtcgtaacaaggtttcc
>0685228f015bd3c775d3c723e1234ae0\_1
gctcctaccgattggatgagttggtgagtgaattggagtaacaggtatctttctgaaagattgttgcattttttacaatttgcaaactagattatctagaggaaggagaagtcgtaacaaggtttcc
>068e3ee3a8e17c6d6c16139a8675e1b0\_1
gctcctaccgattggatgagttggtgagtgaattggagtaactgctatctttctgaaagattgttgtattttttaaaatttgcaaactagattatctagaggaaggagaagtcgtaacaaggtttcc
>06d08eb513069add73d95c4bb3759114\_1
gctcctaccgattggatgagttggtgagtgaattgaagtaacaggtatctttctgaaagattgttgtatttttaaaatttgcaaactagattatctagaggaaggagaagtcgtaacaaggtttcc
>073fa78c4805b184dccde6360958eb82\_1
gctcctaccgattggatgagttggtgagtgaattggagtaacagctatctttctgaaagattgttgttattttttaaaatttgcaaactggattatctagaggaaggagaagtcgtaacaaggtttcc
>07882f881333c91e2c5c2d11885907db\_1
gctcctaccgattggatgagttggtgagtgaattggagtaacagctatctttctgaaagattgttgtattttttaaaatttgcaaactagattatcaagaggaaggagaagtcgtaacaaggtttcc
>08b8c7fb0ca8c4e0c260e57572f7848b\_1
gctcctaccgattggatgagttggtgagtgaattggagtaacatctatctttctgaaagattgttgtatttttaaaatttgcaaactagattatctagaggaaggagaagtcgtaacaaggtttcc
>08ee9922f2cf30b5acb6a67030a11f68\_1
gctcctaccgattggatgagttggtgagtgaattggagtaacaggtatctttctgaaagattgttgcatttttaaaatttgcaaactagattatctaggggaaggagaagtcgtaacaaggtttcc
>0a53a28a54e9e7bc28e17fbd59082be1\_1
gctcctaccaattggatgagttggtgagtgaattggagtaacagctatctttctgaaagattgttgttattttttaaaatttgcaaactagattatctagaggaaggagaagtcgtaacaaggtttcc
>0a5ac2f68c6f4aa601ed9f99a8545880\_1
gctcctaccgattggatgagttggtgagtgaattggagtaacagctatctttctgaaagattgttgtattttttaaaattggcaaactagattatctagaggaaggagaagtcgtaacaaggtttcc
>0ac81830a16f8ce0d221a7b623d8e673\_1
gctcctaccgattggatgagttggtgagtggattggagtaacagctatcttcctgtaagattgttgtatttttttaaaatttgcaaactagattatctagaggaaggagaagtcgtaacaatgtttcc
>0affeb5c212a0b14c7a102d453c52635\_1
gctcctaccgattggatgagttggtgagtgaattggagtaacggctatctttctgaaagattgttgtattttttaaaatttgcaaactagattatctagaggaaggagaagtcgtaacaaggtttcc
>0b6f55fe2f22e639bfebb2ed9ecfecbe\_1
gctcctaccgattggatgagttggtgagtgaattggagtaacaggtatctttttgaaagattgttgcattttttaaaatttgcaaactagattatctagaggaaggagaagtcgtaacaaggtttcc
>0bec96b6339b7888a9c37fcc5677e513\_1
gctcctaccgattggattagttggtgagtgaattggagtaacaggtatctttctgaaagattgttgcattttttaaaatttgcaaactagattatctagaggaaggagaagtcgtaacaaggtttcc
>0c19810b3d54e082495bca2992c4c91b\_1
gctcctaccgattggatgagttggtgagtgaattggagtaacaggtatctttctgaaagattgttgtattttataaatttgcaaactagattatctagaggaaggagaagtcgtaacaaggtttcc
>0c32133903ed9daa50a098f6440ed8cc\_1
gctcctaccgattggatgagttggtgagtggattggagtaacaggtatctttctgaaagattgttgtattttttaaaatttgcaaactagattatctagaggaaggagaagtcgtaacaaggtttcc
>0c9fe09e5a3c928852e36019a563d3c1\_1
gctcctaccgattggttgagttggtgagtgaattggagtaacagctatctttctgaaagattgttgtattttttaaaatttgcaaactagattatctagaggaaggagaagtcgtaacaaggtttcc
>0d4580311ff3261be715d2ef4f3b053a\_1
gctcctaccgattggatgagttggtgagtgaattggagtaacaggtttctttctgaaagattgttgcatttttaaaatttgcaaactagattatctagaggaaggagaagtcgtaacaaggtttcc
>0e1c7d4b23a0792877e601c6bfcaf2b3\_1
gctcctaccgattggatgagttggtgagtgaattggagtaacaggtatctttctgaaagattgttgcattttttaaaatttgtaaactagattatctagaggaaggagaagtcgtaacaaggtttcc
>0ec0b751e0b0530082bdc8bf97f73129\_1
gctcctaccgattggatgagttggtgagcgaattggagtaacagctatctttctgaaagattgttgttattttttaaaatttgcaaactagattatctagaggaaggagaagtcgtaacaaggtttcc
>0f82fecba2ff7138bbaafa67fcb7ff84\_1
gctcctatcgattggatgagttggtgagtgaattggagtaacagctatctttctgaaagattgttgtatttttaaaatttgcaaactagattatctagaggaaggagaagtcgtaacaaggtttcc
>10100500cba9e78ab2ebe09fec49dbb4\_1
gctcctaccgattggatgagttggtgagtgaattggagtaacagctatctttctgaaagattgttgttattttttaaaatttgcaaactagattatctagaggaaggaggagtcgtaacaaggtttcc
>10b63399e9a459c2ec91edb00d5515f5\_1
gctcctaccgattggatgagttggtgagtggattggagtaacagctatcttcatgtaagattgttgtatttttaaaatttgcaaactagattatctagaggaaggagaagtcgtaacaaggtttcc
>10d69dc58d79f42ec809ee12fad7aaee\_1
gctcctaccgattggatgagttggtgagtgaattggagtaacaggtatctttctgaaagattgttgcattttttaatatttgcaaactagattatctagaggaaggagaagtcgtaacaaggtttcc
>10db07f119d1607361db3e5f5e20c58c\_1
gttcctaccgattggatgagttggtgagtgaattggagtaacaactatctttctgaaagattgttgtattttttaaaatttgcaaactagattatctagaggaaggagaagtcgtaacaaggtttcc
>1159ea96ae25cc22307d0361bc13076c\_1
gctcctaccgattggatgagttggtgagtgaattggagtaacaggtatctttatgaaagattgttgcattttttaaaatttgcaaactagattatctagaggaaggagaagtcgtaacaaggtttcc
>122474c2572a767d3d332794cf32b75c\_1
gctcctaccgattggatgagttggtgagtgaattggagtaacagctatctttctgaaagattgttgtattttttaaaatttgcaaactagattatctagaggaaggagaagtcgttacaaggtttcc
>1239cd0562f9b11b996cba6bfac721aa\_1
gctcctaccgattggatgagttggtgagtgaataggagtaacagctatctttctgaaagattgttgtatttttaaaatttgcaaactagattatctagaggaaggagaagtcgtaacaaggtttcc
>124b7cc703d6f5a16a15b9d5ed3fb47f\_1
gctcctaccgattggatgagttggtgagtgaattggagtaacagctatctttctgaaagattgttgtattttttaaaatttgcaaactagattatctagaggaaggagaagtcgtaacaagctttcc
>1276dfc79477c9799bc801ad066412a8\_1
gctcctaccgattggatgagttggtgagtgaattggagtagcaggtatctttctgaaagattgttgtatttttaaaatttgcaaactagattatctagaggaaggagaagtcgtaacaaggtttcc
>12d60d94371c7bc405ac9aa4440e48db\_1
gctcctaccgattggatgagttggtgattgaattggagtaacagctatctttctgaaagattgttgtattttttaaaatttgcaaactagattatctagaggaaggagaagtcgtaacaaggtttcc
>1356a65d4c72c7710bbe122b498d249f\_1
gctcctaccgattagatgagttggtgagtgaattggagtaacagctatctttctgaaagattgttatattttttaaaatttgcaaactagattatctagaggaaggagaagtcgtaacaaggtttcc
>14347667c99f3e962e2c967411c16cc1\_1
gctcctaccgattggatgagttggtgagtgaattggagtaacagctatcttactgaaagattgttgtattttttaaaatttgcaaactagattatctagaggaaggagaagtcgtaacaaggtttcc
>14c578195b9e2311fc02ffec5a08d7e9\_1
gctcctaccgattggatgagttggtgagtgaattggagttacagctatctttctgaaagattgttgtattttttaaaatttgcaaactagattatctagaggaaggagaagtcgtaacaaggtttcc
>1569caf86e44b65492fa21b4522a92ab\_1
gctcctaccgattggatgagttggtgagtgaattggagtaacagctatctttctgaaagattgttgtatttttaaaaatttgcaaactagattatgtagaggaaggagaagtcgtaacaaggtttcc
>15a378022908cf182226124c5e0270c5\_1
gctcctaccgattggatgagttagtgagtgaattggagtaacaggtatctttctgaaagattgttgcatttttttaaaatttgcaaactagattatctagaggaaggagaagtcgtaacaaggtttcc
>161744527965c93ae62e064bebbab226\_1
gctcctaccgattggatgagttggtgagtgaattggagtaacaggtatctttctgaaagattgttgcattttttaaaatttgcaaactagattatgtagaggaaggagaagtcgtaacaaggttttcc
>16207d9ce55035c7e5e4ef2ce6f46815\_1
gctcctaccgattggatgagttggtgagtgaattggagtaacagctatctttctgaaagattgttgtatttttataatttgcaaactagattatctagaggaaggagaagtcgtaacaaggtttcc
>16629e2caf7aef891db6209b3c2ac6f7\_1
gctcctaccgattggatgagttggtgagtgaattggagtaacagctatctttctgaaagattgttgtattttttaacatttgcaaactagattatatagaggaaggagaagtcgtaacaaggtttcc
>16d1e952c4820510d06223f2f2017ef9\_1
gctcctaccgattggatgagttggtgagtgtattggagtaacaggtatctttctgaaagattgttgcattttttaaaatttgcaaactagattatctagaggaaggagaagtcgtaacaaggtttcc
>18127f6f3d0602e15c409b6d7f2786f2\_1
gctcctaccgattggatgagttggtgagtgaattggagtaacaggtatctttctgaaagattgttgcattttaaaatttggaaactagattatctagaggaaggagaagtcgtaacaaggtttcc
>185a215b2018f0903a425c0eb11339a4\_1
gctcctaccgattggatgagttggtgagtgaattgcagtaacagctatctttctgaaagattgttgtattttttaaaatttgcaaactagattatctagaggaaggagaagtcgtaacaaggtttcc
>186e0da9e9fb04c80492a4ba362a80db\_1
gctcctaccgattggatgagttggtgagtgaattggagtaacagctatctttctgaaagattgttgtatttttaaaatttgcaaactagattgtctagaggaaggagaagtcgtaacaaggtttcc
>18888260644dc631956a126f665e6947\_1
gctcctaccgattggatgagttggtgagtgaattggagtaacaggtatctttctgaaaaattgttgcattttttaaaatttgcaaactagattatctagaggaaggagaagtcgtaacaaggtttcc
>19b6583419f1032a7f7558b203ce6b3a\_1
gctcctaccgattggatgagttggtgagtgaattggagtaacagctatctttctgaaagattgttgtattttttaaaatttgcaaactagattatctagaggaaggataagtcgtaacaaggtttcc
>1a85b4cfcdb4729d143557608778b17f\_1
gctcctaccgattggatgagttggtgagtgggttggagtaacagctatcttcctgtaagattgttgtatttttttaaaatttgcaaactagattatctagaggaaggagaagtcgtaacaaggtttcc
>1b0b89baeddf95ac16cff9460c4318b0\_1
gctcctaccgattggatgagttggtgagtgaattggagtaacagctatctttctgaaagattgttgtattttttaaaatttgcaaactaaattatctagaggaaggagaagtcgtaacaagatttcc
>1b1287fc5778d6020e75fe26fb90a9b6\_1
gctcctaccgattggatgagctggtgagtgaattggagtaacagctatctttctgaaagattgttgtattttaaaatttgcaaactagattatctagaggaaggagaagtcgtaacaaggtttcc
>1c1478ff2f8fef7e24ffef1ca118ce6d\_1
gctcctaccgattggatgagttggtgagtgaattggagtaacagctaactttctgaaagattgttgtatttttaaaatttgcaaactagattatctagaggaaggagaagtcgtaacaaggtttcc
>1c66c0e96afffebe442e25a11564d4e3\_1
gctcataccgattggatgagttggtgagtgaattggagtaacaggtatctttctgaaagattgttgcattttttaaaatttgcaaactagattatctagaggaaggagaagtcgtaacaaggtttcc
>1c72c4bb31d986610374991c3766482d\_1
gctcctaccgattggatgagttggtgagtgaattggagtaacagctatctttctgaaagatagttgtattttttaaaatttgcaaactagattatctagaggaaggagaagtcgtaacaaggtttcc
>1cbd4ad44cfd5b5224480271bea4750c\_1
gctcctaccgattggatgagttggtgagtgaatcggagtaacagctatctttctgaaagattgttgtattttttaaaatttgcaaactagattatctagaggaaggagaagtcgtaacaaggtttcc
>1cf6d390988f07c7e5bbd8716fc85702\_1
gctcctaccgattggatgagttggtgagtgaattggagtaacagctatctttctgaaagattgttgtatttttaaaaattagcaaactagattatctagaggaaggagaagtcgtaacaaggtttcc
>1d82cb5f4ec73620fcbd2dfec949a3d0\_1
gctcctaccgattggatgagttggtgagtgaattggagtaacagctatctttctgaaatattgttgtattttttaaaatttgcaaactagattatctagaggaaggagaagtcgtaactaaggtttcc
>1e8041d9c5e4f1b6764113e893c512ca\_1
gctcctaccgattggatgagttggtgagtgaattggagtaacaggtatctttctgaaagattgttgtatttttaaaatttgcaaactagattatctagaggaaggagaagtcgtaacaatgtttcc
>1f9fbe3cd4260785424b548563ee2222\_1
gctcctaccgattggatgagttggtgagtgaattggagtaacaggtatctttctgaaagattgttgcattttttaaaatttgcaaactagaattatctagaggaaggagaagtcgtaacaaggtttcc
>2127dc898b356932181dc929795f5f67\_1
gctcctaccgattggatgagttggtgagtgaatttgagtaacaggtatctttctgaaagattgttgtattttttaaaatttgcaaactagattatctagaggaaggagaagtcgtaacaaggtttcc
>21e23cc91171f8f19ab26171a5ae116a\_1
gctcctaccgattggatgagttggtgagtgaattggagtaacagctatctttctgaaagattgttgcattttttaaaatttacaaactagattatctagaggaaggagaagtcgtaacaaggtttcc
>21f775f0421c3ad10fafb1b4f4ed5062\_1
gctcctaccgattggatgagttggtgagtgaattggagtaacagctatctttctgaaagattgttgttattttttaaaatttgcaaactatattatctagaggaaggagaagtcgtaacaaggtttcc
>221c7b4ba09df1d9a4f845b057222520\_1
gctcctaccgattggatgagttggtgagtgaattggagtaacaggcatctttctgaaagattgttgcattttttaaaatttgcaaactagattatctagaggaaggagaagtcgtaacaaggtttcc
>22b47e664afdad7bdf22f7233203b660\_1
gctcctaccgattggatgagttggtgagtgaattggagtaacaggtatctttctgaaagattgttgtatttttttaaaaatttgcaaaactagattatctagaggaaggagaagtcgtaacaaggttttcc
>2336c8ce92c3c85ad63fbe4bcf49aed1\_1
gctcctaccgattggatgagatggtgagtgaattggagtaacaggtatctttctgaaagattgttgcattttaaaatttgcaaactagattatctagaggaaggagaagtcgtaacaaggtttcc
>2390354f8487a8e0325030a9988bae77\_1
gctcctaccgattggatgagttagtgagtgaattggagtaacaggtatctttctgaaagattgttgcattttttaaaatttgcaaactaaattatctagaggaaggagaagtcgtaacaaggtttcc
>239ab0f422556292bed40a44cb49ac09\_1
gctcctaccgattggatgaattggtgagtgaattggagtaacagctatctttctgaaagattgttgtatttttaaaaatttgcaaactagattatctagaggaaggagaagtcgtaacaaggtttcc
>23e8195b2de726a6da584879d573fe7a\_1
gcttctaccgattggatgagttggtgagtgaattggagtaacaggtatctttctgaaagattgttgtatttttaacatttgcaaactagattatctagaggaaggagaagtcgtaacaaggtttcc
>245cc859df2f506d264c61d4c08ec77f\_1
gctcctaccgattggatgagttggtgagtgaattggagtaacaggtaactttctgaaagattgttgcatttttaaaatttgcaaactagattatctagaggaaggagaagtcgtaacaaggtttcc
>248fb559e8e976c11c862d3c8e799e0e\_1
gctcctaccgattggatgagttggtgagtgtattggagtaacagctatctttctgaaagattgttgtattttttaaaatttgcaaactagattatctagaggaaggagaagtcgtaacaaggtttcc
>24ff370503356554cc3ca33aed570c6c\_1
gctccaaccgattggatgagttggtgagtgaattggagtaacagctatctttctgaaagattgttgttattttttaaaatttgcaaactagattatctagaggaaggagaagtcgtaacaaggtttcc
>2653cd11d51211f582a7d34204c54198\_1
gctcctaccgattggatgagttggtgagtgaattggagtaacagctatctttctgatagattgttgtatttttaaaaatttgcaaactagattatctagaggaaggagaagtcgtaacaaggtttcc
>26aa8d803462ad3d5a8ca02259034c9b\_1
gctcctaccgattggatgagttggtgagtgaattggagtaacaggtatctttctgaaagattgttgtattttttaaaatttgcaaactagattatcttagaggaaggagaagtcgtaacaaggtttcc
>26be46be335d25dd0ad6f9010e97a739\_1
gctcctaccgattggatgagttggtgagtgaattggagtaacagctatctttctgaaagattgttgtattttttaaaatttgcaaactagattatctagagcaaggagaagtcgtaacaaggtttcc
>26c7594067d9a244dcfc62f28d5623f7\_1
gctcctaccgattggatgagttggtgagtgaaatggagtaacagctatctttctggaagattgttgtattttttaaaatttgcaaactagattatctagaggaaggagaagtcgtaacaaggtttcc
>278f7a6ced5be02048093e144cd2bb25\_1
gctcctaccgattggatgagttggtgagtgaattggagtaacaggtatctttctgaaagattgttgcattttttaagatttgcaaactagattatctagaggaaggagaagtcgtaacaaggtttcc
>284f8de3bce9331fe60e78f03643dc72\_1
gctcctaccgattggatgagttggtgagtgaattggagttaacagctatctttctgaaagattgttgtattttttaaatttgcaaactagattatctagaggaaggagaagtcgtaacaaggtttcc
>285a6ad0a7c020dc54749ae257d51eaf\_1
gctcctaccgattggatgagttggtgagtgaattggagtaacagctatctttctgaaagattgttgtattttttaaaattttcaaactagattatctagaggaaggagaagtcgtaacaaggtttcc
>28882de9cd548c83a58ec49263c6c117\_1
gctcctaccgattggatgagttggtgagtgaattggagtaacagctatctttctgaaagattgttgtattttttaaaatttgcaaactagattatcaagaggaaggagaagtcgtaacaaggttttcc
>28d2ae9dc1d3763706329d7acb5f2098\_1
gctcctaccgattggatgagttggtgagtgaattggagtaacagctatctttctgtaagattgttgtattttttaaaatttgcaaactagattatctagaggaaggagaagtcgtaacaaggtttcc
>29355c087750488dce2bcc2dab235fbd\_1
gctcctaccgattggatgagttggtgagtgaattggagtaacagctatctttctgaaagattgttgtattttttaaaatttgcaaactagattatctagaggaaggagaagtcgtaacaggtttcc
>2935ec6fda3fe0492e0756e5e0a6c81b\_1
gctactaccgattggatgagttggtgagtgaattggagtaacagctatctttctgaaagattgttgtattttttaaaatttgcaaactagattatctagaggaaggaaaagtcgtaacaaggtttcc
>29e64caece9ac125bf66846650e9f14e\_1
gctcctaccgattggatgagttggtgagtgaattggagtaacagctatctttctgaaagattgttgtattttttaaaatttgcaacctagattatctagaggaaggagaagtcgtaacaaggtttcc
>2a50ccca1727198eb08e0185545aaadd\_1
gctcctaccgattggatgagttggtgagtgaattggagtaacaggtatctttctgaaagattgttgcaattttttaaaatttgcaaactagattatctagaggaaggagaagtcgtaacaagatttcc
>2a59b6f88dff11bb1c4de77ebf5f1c2e\_1
gctcctaccgattggatgagttggtgagtgaattggagtaaccggtatctttctgaaagattgttgcattttttaaaatttgcaaactagattatctagaggaaggagaagtcgtaacaaggtttcc
>2ba6ad860bdf09c066effac8a2e0c3e8\_1
gctcctaccgattggatgagttggtgagtgaattggtgtaacagctatctttctgaaagattgttgtattttttaaaatttgcaaactagattatctagaggaaggagaagtcgtaacaaggtttcc
>2bdaf1bc94b78366e96cfee5a258869b\_1
gctcctaccgattggatgagttggtgagtgaattggagtaacagctatctttctgaaagattgttgtatttttaaaaattgcaaactagattatctagaggaaggagaagtcgtaacaaggtttcc
>2c774c7c4f6a7aba397ec20773fdf90e\_1
gctactaccgattggatgagttggtgagtgaattggagtaacagctatctttctgaaagattgttgcattttttaaaatttgcaaactagattatctagaggaaggagaagtcgtaacaaggtttcc
>2cb5b9d1070a932ab78dbeaed22f1c94\_1
gctcctaccgattggatgagttggtgagtgaattggagtaacagctatctttctgaaagattgttgtattttttaaaatatgcaaactagattatctagaggaaggagaagtcgtaacaaggtttcc
>2cc32750f7486e4282246cfd86800104\_1
gctcctaccgattggatgagttggtgagtgaattggagtaacagctatctttctgaaagattgttgtatttttaaaaatttgcaaactagattacctagaggaaggagaagtcgtaacaaggtttcc
>2ce8331f9dae37344189cf3739325845\_1
gctcctaccgattggatgagttggtgagtgaattggagtaaaagctatctttctgaaagattgttgtattttataaaatttgcaaactagattatctagaggaaggagaagtcgtaacaaggtttcc
>2ceb6d607e72edb62b8dd5c45d2d438d\_1
gctcctaccgattggatgagttggtgagggaattggagtaacaggtatctttctgaaagattgttgttattttttaaaatttgcaaactagattatctagaggaaggagaagtcgtaacaaggtttcc
>2e9bd4a6f66cf01d4885f697a2918255\_1
gctcctaccgattggatgagttggtgagtgaattggagtaacagctatctttctgaaagattgttgtatttttaaaaatttgcaaactagattatctagaggaaggagagtcgtaacaaggtttcc
>2ee7deb8b30b3d3863b1afa43f754b86\_1
gctcctaccgattggatgagttggtgagtgaattggagtaacagctatctttctgaaagattgttgtatttttaaaaatttgcaaactagattatctagaggaaggaaaagtcgtaacaaggtttcc
>30308bf6f24478503516def55d3ae429\_1
gctcctaccgattggatgagttggtgagtgaattggagtaacaggtatctttctgaaagattgttgtatttttaaaatttgcaaactagattatctagaggaaggagaagtcgtaacaaggttttcc
>308132f1932453c7bd39eab695c6cb76\_1
gctcctaccgattggatgagttggtgagtgaattggagtaacagctatctttctgaaagattgttgtattttttaacatttgcaaactagattatctagaggaaggagaagtcgtaacaaggtttcc
>30847224f80f0aad42ac8ea0a4728750\_1
gctcctaccgattggatgagttggtgagtgatttggagtaacagctatctttctgaaagattgttgtatttttaaaaatttgcaaactagattatctagaggaaggagaagtcgtaacaaggtttcc
>30fb857ce86cf70dd255bf7c408d02bc\_1
gctcctaccgattggatgagttgattagtgaattggagtaacagctatctttctgaaagattgttgtattttttaaaatttgcaaactagattatctagaggaaggagaagtcgtaacaaggtttcc
>310722861d7944fd354a66e77b7424c6\_1
gctcctaccgattggatgagttggtgagtgaattggagtaacaggtatctttctgaaagattgttgcatttttaaaatttgcaaactagattatctagaggaaggagaagtcgtaacacggtttcc
>317b54cbf450b9ade2fa18c87ac9aef0\_1
gctcctacgattggatgagttggtgagtgaattggagtaacaggtatctttctgaaagattgttgcattttttaaaatttgcaaactagattatctagaggaaggagaagtcgtaacaaggtttcc
>31c19ddeb87e242a98bce68f0ddc28c3\_1
gctcctaccgattggatgagttggtgagtgaattagagtaacagctatctttctgaaagattgttgtattttttaaaatttgcaaactagattatctagaggaaggagaagtcgtaacaaggtttcc
>33236c354a3032241d84a00fc94b64cb\_1
gctcctaccgattggatgagttggtgagtgaattggagtaacagctatctttctgaaggattgttgtattttttaaaatttgcaaactagattatctagaggaaggagaagtcgtaacaaggtttcc
>3328e3d82f752fde0a8b0712591bcd44\_1
gctcctaccgattggatgagttgttgagtgaattggagtaacagctatctttctgaaagattgttgttaattttttaaaatttgcaaactagattatctagaggaaggagaagtcgtaacaaggtttcc
>340c5015f7eb4be6156e8466bd41b85b\_1
gctcctaccgattggatgagttggtgagtgaattggagtaacagctatctttctgaaagattgttgcatttttaaaatttgcaaactagattatctagaggaaggagaagtcgtaacaaggtttcc
>3416002e666fb642ff374eb8657524a2\_1
gctcctaccgattggatgagttggtgagtgaatttgagtaacagctatctttctgaaagattgttatattttttaaaatttgcaaactagattatctagaggaaggagaagtcgtaacaaggtttcc
>3417140762aac4bd9deac72e50d93dc7\_1
gctcctaccgattggatgagttggtgagtgaattggagtaacagctatctttctgaaagattgttgtattttttaaaatttgcaaactagattaactagaggaaggagaagtcgtaacaaggtttcc
>34fbd494d2d10a5e8fffafb122366486\_1
gctcctaccgattggatgagttggtgagtgaattggagtaacagctatctttctgaaagattgttgttattttttaaaatttgcaaactagattatctagaggaaggagaagttgtaacaaggtttcc
>355ac2fc69eaa1f6c1796b4ab0c97104\_1
gctcctaccgattggatgagttggtgagtgaattggagtaacagctatatttctgaaagattgttgtattttttaaaatttgcaaactagattatctagaggaaggagaagtcgtaacaaggttttcc
>357a672a000e948ad97309a89a0fc67c\_1
gctcctaccgattggatgagttggtgagtgaattggagtaacagctatctttctgaaagattgttgtattttttaaaatttgcaaactagattatctagacgaaggagaagtcgtaacaaggtttcc
>35f80bf06f786cef9ea2f4460038bbda\_1
gctcctaccgattggatgagttggtgagtgaattggagtaacagctatctttctgaaagattgttgtattttttaaaatttgcaaactagatcatctagaggaaggagaagtcgtaacaaggtttcc
>36b82e169ab42437b82b03bc9430a616\_1
gctcctaccgattggatgagttggtgagtgaattggattaacagctatctttctgaaagattgttgttattttttaaaatttgcaaactagattatctagaggaaggagaagtcgtaacaaggtttcc
>37575237339e926b5997f2ccb90ec62d\_1
gctcctaccgattggatgagttggtgagtgaattggagtaacagctatctttctgaaagattgttgtatttttttaaaatttgcaaactagattatctagaggaagtaaaagtcgtaacaaggtttcc
>37b63bedfcda8902714b6639eba4bf9b\_1
gctcctaccgattgtatgagttggtgagtgaattggagtaacagctatctttctgaaagattgttgtatttttaaaatttgcaaactagattatctagaggaaggagaagtcgtaacaaggtttcc
>3829197c0059092799d0a4522d3fc496\_1
gctcctaccgattggatgagttggtgagtgaattggagtaatagctatctttctgaaagattgttgtattttttaaaatttgcaaactagattatctagaggaaggagaagtcgtaacaaggttttcc
>3841ca89eb1862314b577c6f64e7639b\_1
gctcctaccgattggatgagttggtgagtggattggagtaacagctatcttcctgtaagattgttgttatttttttaaatttgcaaactagattatctagaggaaggagaagtcgtaacaaggtttcc
>38a8ab637183c70ebefab99615124497\_1
gctcctaccgattggatgagttggtgagtgaattggagtaacaggtatctttctgaaagattgttgtattttttaaaacttgcaaactagattatctagaggaaggagaagtcgtaacaaggtttcc
>3959c575de51dedb2f2ca1d801af8d98\_1
gctcctaccgattggatgagttggtgagtggattggagtaacagctatcttcctgaaagattgttgtatttttaaaatttgcaaactagattatctagaggaaggagaagtcgtaacaaggtttcc
>3a148100e5adebf188230f88ff0d80b1\_1
gctcctaccgattggatgagttggtgagtgaattggagtaacaggtatctttctgaaagattgttgcattttttaaaatttgcaaactagattacctagaggaaggagaagtcgtaacaaggtttcc
>3b1ea88c522d0f228a21832e23f65a77\_1
gctcctaccgattggatgagttggtgagtgaattggagtaacagctatctttctgaaagcttgttgtattttttaaaatttgcaaactagattatctagaggaaggagaagtcgtaacaaggtttcc
>3b3a35e7712675aa407a995e5fbe3305\_1
gctcctaccgattggatgagttagtgagtgaattggagtaacaggtatctttctgaaagattgttgtattttttaaaatttgcaaactagattatctagaggaaggagaagtcgtaacaaggtttcc
>3becb8ebc455f2264e60c3c24c0f123f\_1
gctcctaccgattggatgagttggtgagtgaattggagtaacagctatctttctgaaaggttgttattattttttaaaatttgcaaactagattatctagaggaaggagaagtcgtaacaaggtttcc
>3ca539cac3cdd7a4f85dccfb5a27bea5\_1
gctcctaccgattggatgagttggtgagtgaattgaagtaacagctatctttctgaaagattgttgtatttttaaaatttgcaaactagattatctagaggaaggagaagtcgtaacaaggtttcc
>3d6884f374e36bb01a7b263241043e2a\_1
gctcctaccgattggatgagttggtaagtgaattggagtaacagctatctttctgaaagattgttgtattttaaaatttgcaaactagattatctagaggaaggagaagtcgtaacaaggtttcc
>3e2ba2ebdcf6ac51022bb35e0ce0d0dd\_1
gctcctaccgattggatgagttggtgagtgtattggagtaacagctatctttctgaaagattgttgtattttttaaaatttacaaactagattatctagaggaaggagaagtcgtaacaaggtttcc
>3e31e46b366ed11828936cddee1ba78b\_1
gctcctaccgattggatgagttgggagtgaattggagtaacagctatctttctgaaagattgttgtattttttaaaatttgcaaactagattatctagaggaaggagaagtcgtaacaaggtttcc
>3e578931a6a7339fb5e8d90186feee2e\_1
gctcctaccgattggatgagttggtgagtgaaatggagtaacaggtatctttctaaaagattgttgtattttttaaaatttgcaaactagattatctagaggaaggagaagtcgtaacaaggtttcc
>3f191dc0f59e9271d7c2dd5681af220d\_1
gctcctaccgattggatgagttggtgagtgaattggagtaacagctatctttctgaaagattgttgtatttattaaaatttgcaaactagattatctagaggaaggagaagtcgtaacaaggtttcc
>3f3eeb85d736134c0f3d428ae2fdcbd8\_1
gctcctaccgattggatgagttggtgagtgaattggagtaacagctatctttctgaaagagtgttgtattttttaaaatttgcaaactagattatctagaggaaggagaagtcgtaacaaggtttcc
>3f68db95c32b4cf8b707b9367dd54141\_1
gctcttaccgattggatgagttggtgagtgaattggagtaacaggtatctttctgaaagattgttgtattttttaaaatttgcaaactagattatctagaggaaggagaagtcgtaacaaggtttcc
>40387753af0e108a005e39af2ead585c\_1
gctcctaccgattggatgagttggtgagtgcattggagtaacaggtatctttctgaaagattgttgcattttttaaaatttgcaaactagattatctagaggaaggagaagtcgtaacaaggttttcc
>41907293f1b098d2ca56325deef216f4\_1
gctcctaccgattggatgagttggtgagtgaattggagtaacaggtatctttcggaaagattgttgcattttttaaaatttgcaaactagattatctagaggaaggagaagtcgtaacaaggtttcc
>42eb890f1899f11f70e9a353184af50f\_1
gctcctaccgattggatgagttggtgagtgaattggagtaacaggtatctttctaaaagattgttgtattttttaaaatttgcaaactagattatctagaggaaggagaagtcgtaacaaggtttcc
>433524c25f97a38c0e4f0d549872ce50\_1
gcccctaccgattggatgagttggtgagtgaattggagtaacagctatctttctgaaagattgttgtattttttaaaatttgcaaactagattatctagaggaaggagaagtcgtaacaaggtttcc
>44b991b88d3ea215ed4b72cd4b404b6c\_1
gctcctaccgattggatgagttggttgagtggattggagtaacaggtatctttctgaaagattgttgtattttttaaaatttgcaaactagattatctagaggaaggagaagtcgtaacaaggtttcc
>44c2f909ee7e153332faa5d0eb455d07\_1
gttcctaccgattggatgagttggtgagtgaattggagtaacagctatctttctgaaagattgttgtatttttaaaatttgctaactagattatctagaggaaggagaagtcgtaacaaggtttcc
>45a9abcd2033141adf698e7b592bfc46\_1
cctcctaccgattggatgagttggtgagtgaattggagtaacaggtatctttctgaaagattgttgcatttttaaaatttgcaaactagattatctagaggaaggagaagtcgtaacaaggtttcc
>4710298f9316c4760395d21e5893b3fa\_1
gctcctaccgattgtatgagttggtgagtgaattggagtaacagctatctttctgaaagattgttgtattttttaaaatttgcaaactagattatctagaggaaggagaagtcgtaacaaggtttcc
>4778a7c56ea9e34d0c4f8c6aacd05ec5\_1
gctcctaccgattggatgagttggtgagtgaattggagtaacagctatctttctgaaagattgtgtatttttaaaatttgcaaactagattatctagaggaaggagaagtcgtaacaaggtttcc
>47a1d4e377e25c6a9f7059739308333b\_1
gctcctaccgaatggatgagttggtgagtgaattggagtaacagctatctttctgaaagattgttgtattttttaaaatttgtaaactagattatctagaggaaggagaagtcgtaacaaggtttcc
>47e1ea4704873bbbc88d5cda56b4895f\_1
gctcctaccgattggatgagttggtgagtgaattggagtaacagctatctttctgaaagattgttgtattttttaaaatttgcaaactagattatctagaggaaggagaagtcgtaacatggtttcc
>47f5656ae18538352dae1d477019793d\_1
gctcctaccgattggatgagttggtgagtgaattggagtaacagctatctttctgaaagattgttgtatttttaaaatttgcaaactagattatctagaggaaggagaagtcgtaacaaggtgtcc
>48147a214380138b642f43770124860c\_1
gctcctaccgattggatgagttggtgagtgaattggagtaacagctatctttctgaaagattgttgtattttaaaatttgtaaactagattatctagaggaaggagaagtcgtaacaaggtttcc
>4a6a4ab99477897e9e56ea28e0947e4f\_1
gctcctaccgattggatgagttggtgagtgaattggagtaacaggaatctttctgaaagattgttgtatttttaaaatttgcaaactagattatctagaggaaggagaagtcgtaacaaggtttcc
>4b98ded714ec1308ab623d02e8ba4bcb\_1
gctcctaccgattggatgagttggtgagtgaattggagtaacagctatctttctagaaagattgttgtattttttaaaatttgacaaactagattatctagaggaaggagaagtcgtaacaaggtttcc
>4e6735d661aedb51067a3d633154973e\_1
gctcctaccgattggatgagttggtgagtgaattggagtaacaggtatctttctgaaagattgttgtacttttaaaatttgcaaactagattatctagaggaaggagaagtcgtaacaaggtttcc
>4f45ad011f01be2b69c309cd48e2d9c3\_1
gctcctaccgattggatgagttggtgagtgaattgaagtaacagctatctttctgaaagattgttgtattttttataatttgcaaactagattatctagaggaaggagaagtcgtaacaaggtttcc
>4f4eda6fb082b33d733baad2850170d3\_1
gctactaccgattggatgagttggtgagtgaattggagtaacagctatcattctgaaagattgttgtattttttaaaatttgcaaactagattatctagaggaaggagaagtcgtaacaaggtttcc
>4fa9c3309f942c83364c60799aa9c1af\_1
gctcctaccgattggatgagttggtgagtgaattggagtaacagctatctttctgaaagattgttgtattttttaaaatttgcaaactagattatctagaggaaggagaagtggtaacaaggtttcc
>4fb4879bddf74152b0fe6d9c26b6e01b\_1
gctcctaccgattggatgagttggtgagtgaattggagtaacagctatctttctgaaagattgttgttaattttttaaaatttgcaaactagattatctagagaaaggagaagtcgtaacaaggtttcc
>507a01ae8bd0c7eda5a7b28051556e3a\_1
gctcctaccgattggatgagttggtgagtgaattggagtaacagttatctttctgaaagattgttgtattttttaaaatttgccaactagattatctagaggaaggagaagtcgtaacaaggtttcc
>50fb30dcd21c4d62cb07686e7c6000ca\_1
gctcctaccgattggatgagttggtgagtgcattggagtaacaggtatctttctgaaagattgttgcatttttaaaatttgcaaactagattatctagaggaaggagaagtcgtaacaaggtttcc
>51060b416842d18d1b25664fff5c3510\_1
gctcctaccgattggatgagttggcgagtgaattggagtaacagctatctttctgaaagattgttgttattttttaaaatttgcaaactagattatctagaggaaggagaagtcgtaacaaggtttcc
>512a3b031382b9b8e28aaa6c65c73187\_1
gctcctaccgattggatgagttggtgagtgaattggagtaacagctatctttctgaaagattgttgtatttttaaacatttgcaaactagattatctagaggaaggagaagtcgtaacaaggtttcc
>524b3a26594924d28c187af946c33382\_1
gctcctaccgattggatgagttggtgagtgaattggagtaacagctatctttttgaaagattgttgtattttttaaaatttgcaaactagattatctagaggaaggagaagtcgtaacaagatttcc
>564935d33c0e1b8c63b0091bdb13a022\_1
gctactaccgattggatgagttggtgagtgaattggagtaacagctatctttctgaaagattgttgtattttttaaaatttgcaaactagattatctagtaggaaggagaagtcgtaacaaggtttcc
>569709b411f08665957496d1f76d0762\_1
gctcctaccgattggatgagttggtgagtgaattggagtaacaggtatctgtctgaaagattgttgtattttttaaaatttgcaaactagattatctagaggaaggagaagtcgtaacaaggtttcc
>56c3efdec54b07ef71b2810596176c48\_1
gctcctaccgattggatgagttggtgagtgaattggagtaacagctatctttctgaaagattgttgtatattttaaaatttgcaaactagattatctagaggaaggagaagtcgtaacaaggtttcc
>595e90c22566930f825d38d13ae1e780\_1
gctcctaccgattggatgagttggtgagtgaattggagtaacaactatctttctgaaagattgttgtattttttaaaatttgcaaactagattatctagaggaaggagaagtcgtaacaaggtttcc
>599943fad4762ea0fc8995c2081b161e\_1
gctcctaccgattggatgagttggtgagtgaattggagtaacagctatctttctgaaagattgttgtattttttgaaatttgcaaactagattatctagaggaaggagaagtcgtaacaaggtttcc
>59ab0adf451baf3a8e3074c8d1feb9c8\_1
gctcctaccgattggatgagttggtgagtgaattggagtaacagctatctttctgaaagattgttgtattttttaaaacttgcaaactagattatctagaggaaggagaagtcgtaacaaggtttcc
>59eb1a665c2e6cb43058e7223d9ec146\_1
gctcctaccgattggatgagttggtgagtgaattggagtaacaggtatctttctgaaagattgtagcattttttaaaatttgcaaattagattatctagaggaaggagaagtcgtaacaaggtttcc
>5a2e75dfcb022650ae8a25766daecc65\_1
gctactaccgattggatgagttggtgagtgaattggagtaacagctatctttctgaaagattgttgtattttttaaaatttggaaactagattatctagaggaaggagaagtcgtaacaaggtttcc
>5e164f0d2c02999a5078eeb4ee4f0dbb\_1
gctcctaccgattggatgagttggtgagtgaattggagtaacagctatctttctgaaagattgttgtattttttaaaatttgcaaactagattatctagaggaaggaaagtcgtaacaaggtttcc
>5ea92bef1b053595ef1338c8e4338f0c\_1
gctcctaccgattggatgagttggtgagtgaattggagtaacagctatctttctgaatgattgttgtattttaaaatttgcaaactagattatctagaggaaggagaagtcgtaacaaggtttcc
>5f3a6b241e72db8d430fd42a8c69919f\_1
gctcctaccgattggatgagttggtgagtgaattggagtaatagctatctttctgaaagattgttgtattttttaaaatttgcacactagattatctagaggaaggagaagtcgtaacaaggtttcc
>5f5f76f564f5767f2b52a93c870e8fe4\_1
gctcctgccgattggatgagttggtgagtgaattggagtaacaggtatctttctgaaagattgttgcatttttaaaatttgcaaactagattatctagaggaaggagaagtcgtaacaaggtttcc
>5f90b7b4dc8dd211815e7648008867a1\_1
gctcctaccgattggatgagttggtgagtgaattggagtaacagctatctttctgaaagattgttgtattttttaaaatttgctaaactagattatctagaggaaggagaagtcgtaacaaggtttcc
>5fbdc680d2f4109b4d3396b9cf9eb2a0\_1
gctcctaccgattggatgagttggtgagtgaattggagtaacagctatctttctgaaagattgttgtattttttaaaatttgcaaactagattatctagaggaaggagaagtcgtaaccaggtttcc
>5fcb7e67429de0c12db57c509dc58ab7\_1
gctcctaccgattggatgagttggtgagtgaattggagtaacagctatctttctgaaagattgttgtattttttaaaatttgcaaactagattatctataggaaggagaagtcgtaacaaggtttcc
>5fe8580ba8e8485c860c2da3122582f4\_1
gctcctaccgattggatgagttgatgagtgaattggagtaacagctatctttctgaaagattgttttattttttaaaatttgcaaactagattatctagaggaaggagaagtcgtaacaaggtttcc
>6046ae27ccafc79fe3bfb2cefbe7f601\_1
gctcctaccgattggatgagttggtgagtggattggagtaacagctatcttcctgtaatattgttgtattttttaaaatttgcaaactagattatctagaggaaggagaagtcgtaacaaggtttcc
>607334f40b1d218cc31c74991a821867\_1
gctcctaccgattggatgagttggtgagtgaattggagtaacaggtatctttctgaaagattgttgtattttttaaaatttgcaaactagattatctagaggaaggagaagtcgtaacaaggtttca
>6107f6b8892e137dd579778c7bebe5aa\_1
gctcctaccgattggatgagttggtgggtgaattggagtaacaggtatctttctgaaagattgttgcattttttaaaatttgcaaactagattatctagaggaaggagaagtcgtaacaaggtttcc
>629a2c9514137ffdce865057bd9d4b88\_1
gctcctaccgattggatgagttggtgagtgaattggagtaacaggtatctttctgaatgattgttgcattttttaaaatttgcaaactagattatctagaggaaggagaagtcgtaacaaggtttcc
>62c25350a9bcf43bcf893a913f02b422\_1
gctcctaccgattggatgagttggtgagtgaattggagtaacaggtatctttctgaaagattgttgctattttttaaaaattgcaaactagattatctagaggaaggagaagtcgtaacaaggttttcc
>62e9ae0fef137769eb50b40b6e5643be\_1
gctcctaccgattggatgagttggtgagtgaattggagtaacagctatctttctgaaagattgttgttattttttaaaatttgcaaactagattatctagaggaagaagaagtcgtaacaaggtttcc
>640ecebe3a12c7cb28d155c3421f80a2\_1
gctcctaccgattggatgagttggtgagtgaattggagtaacagctatctttctgaaagattgttgtattttttataaaatttgcaaactagattatctagaggaaggagaagtcgtaacaaggtttcc
>65ef4e849e99cb6d096702396abdcd17\_1
gctcctaccgattggatgagttggtgagtgaattggagtagcagctatctttctgaaagattgttgtattttttaaaatttgcaaactagattatctagaggaaggagaagtcgtaacaaggtttcc
>671109f9765dfec4eb2a025f8b20a885\_1
gctcctaccgattggatgagttggtgagtgaattggagtaacagctatctttctgaaagattgttgtattttttaaaatttgcaaactagattatcttagaggaaggagaagtcgtaacaaggtttcc
>671bf5522aa40f85ca7ac31e3f115198\_1
gctcctaccgattggatgagttggtgagtgaattggagtaacaggtatctttctgaaagattgttgtattttaaaatttgtaaactagattatctagaggaaggagaagtcgtaacaaggtttcc
>68c7e5cac0269189d29fdbd23af18653\_1
gctcctaccgattggatgagttggtgagtgaattggagtgacaggtatctttctgaaagattgttgcatttttaaaatttgcaaactagattatctagaggaaggagaagtcgtaacaaggtttcc
>6a0630500bb0509c80aa255ec5ec6a71\_1
gctcctaccgattggatgagttggtgagtgaattggagtaacaggtatctttctgaaagattgttgcattttttaaaatttgcaaactagattatctagagtaaggagaagtcgtaacaaggtttcc
>6a083e1bd6b421696565962b6034d0b4\_1
gctgctaccaattggatgagttggtgagtgaattggagtaacagctatctttctgaaagattgttgtattttttaaaatttgcaaactagattatctagaggaaggagaagtcgtaacaaggtttcc
>6a7a5c040a285dd76e799c72544a54fb\_1
actcctaccgattggatgagttagtgagtgaattggagtaacagctatctttctgaaagattgttgtattttttaaaatttgcaaactagattatctagaggaaggagaagtcgtaacaaggtttcc
>6ab5efae5eead4d07a97a4320d01d35b\_1
gctcctaccgattggatgagttggtgagtgaattggagtaacagctatctttcagaaagattgttgtattttttaaaatttgcaaactagattatctagaggaaggagaagtcgtaacaaggtttcc
>6acaf506b570860f338e5cf5f0fe0549\_1
gctcctaccgatgggatgagttggtgagtgaattggagtaacagctatctttctgaaagattgttgtattttttaaaatttgcaaactagattatctagaggaaggagaagtcgtaacaaggtttcc
>6b14e119610a47759f7fedcfc70d4cd0\_1
gctcctaccgattggatgagttggtgagtgaattggagtaacagctatctttctgaaagattgttgttattttttaaattttgcaaactagattatctagaggaaggagaagtcgtaacaaggtttcc
>6b681c62a62a45ee3c908227e23703d1\_1
gctcctaccgattggatgagttggtgagtgaattggagtaacaggtatctttctgaaagattgttgtattttttaaaatttgccaactagattatctagaggaaggagaagtcgtaacaaggtttcc
>6c4cac6bc8a1975de4f86d245a8e3edf\_1
gctcctaccgattggatgagttggtgagtgaattggagtaacagctatatttctgaaagattgttgtattttttaaaatttgcaaactagattatctagaggaaggagaagtcgtaacaaggtttcc
>6c76ef2dc906d829207de03eed8dbeb4\_1
gttcctaccgattggatgagttggtgagtgaattggagtaacagctatctttctgaaagattgttgtattttaaaatttgcaaactagattatctagaggaaggagaagtcgtaacaaggtttcc
>6d51275c7d456d8477fdcc4e59dbfd04\_1
gctcctaccgattggatgagttggtgagtgaattggagtaacagctatctttctgaaagattgttgtattttaaaatttgcaagctagattatctagaggaaggagaagtcgtaacaaggtttcc
>6dda8ea8b38a26336e93873c32d1bce4\_1
gctcctactgattggatgagttggtgagtgaattggagtaacagctatctttctgaaagattgttgtattttaaaatttgcaaactagattatctagaggaaggagaagtcgtaacaaggtttcc
>6e0a6d294b39df2673426ba60a6a7eb6\_1
gctcctacctattggatgagttggtgagtgaattggagtaacagctatctttctgaaagattgttgtattttttaaaatttgcaaactagattatctagaggaaggagaagtcgtaacaaggtttcc
>6e93928a240c148192a6053acecd027d\_1
gctcctaccgattggatgagttggtgagtgaattggagtaacagctatctttctgaaagattgttgttgttttttaaaatttgcaaactagattatctagaggaaggagaagtcgtaacaaggtttcc
>6fa22962d8d4acaaf625f1d186e3b0f9\_1
gctcctaccgattggatgagttggtgagtgaattggagtaacaggtatttttctgaaagattgttgtattttttaaaatttgcaaactagattatctagaggaaggagaagtcgtaacaaggtttcc
>6fcd8f7c48d6acd3446291ad5d542d69\_1
gctcctaccgattggatgagttggggagtgaattggagtaacaggtatctttctgaaagattgttgcatttttaaaatttgcaaactagattatctagaggaaggagaagtcgtaacaaggtttcc
>7092bc94489f7fd6a15953f49595ef7d\_1
gctcctaccgattggatgatttggtgagtgaattggagtaacagctatctttctgaaagattgttgttattttttaaaatttgcaaactagattatctagaggaagaagaagtcgtaacaaggtttcc
>70af834a6dd13916ea2f621adf46cfa5\_1
gctcctaccgattggatgagttggtgagtgaattggagtaacaggtatctttctgaaagattgttgtattttttaaaatttgcaaactagattttctagaggaaggagaagtcgtaacaaggtttcc
>71342f4adbcf84da2f4104d289ee7c2c\_1
gctcctaccgattgggtgagttggtgagtgaattggagtaacagctatctttctgaaagattgttgtattttttaaaatttgcaaactagattatctagaggaaggagaagtcgtaacaaggtttcc
>71a8f6331fab07cbcef073a1db0edbee\_1
gctcctaccgattggatgagttggtgagtgaattggagtaacagctatctttctgaatgattgttgtatttttaaaatttgcaaactagattatctagaggaaggagaagtcgtaacaaggtttcc
>72777b0267ff5a629a5c5d5acee88fdf\_1
gctcctaccgattggatgagttggtgagtgaattggagtaacagctatctttctgaaagattgttgttaattttttaaaatttgcaaactagattatctagaggagggagaagtcgtaacaaggtttcc
>7295be47c49d6bff8569c4c43d590ecb\_1
gctcctaccgattggatgagttggtgagtgagttggagtaacagctatctttctgaaagattgttgtattttttaaaatttgcaaactagattgtctagaggaaggagaagtcgtaacaaggtttcc
>735d39163c2a5c5e39e7a6a07ab82949\_1
gctcctaccgattggatgagttggtgattgaattggagtaacagctatctttctgaaagattgttgttattttttaaaatttgcaaactagattatctagaggaaggagaagtcgtaacaaggtttcc
>73683c8a66f3d1f61bba40fb1d1f3d56\_1
gctcctaccgattggatgagttactgagtgaattggagtaacagctatctttctgaaagattgttgttattttttaaaatttgcaaactagattatctagaggaaggagaagtcgtaacaaggtttcc
>74b7f0b6fe817a365be4414c576a9b28\_1
gctcctaccgattggatgagttggtgagtgaattggactaacagctatctttctgaaagattgttgtatttttaaaatttgcaaactagattatctagaggaaggagaagtcgtaacaaggtttcc
>758aab8f7de3e37e7d133e5968d29bba\_1
gctcctaccgattggatgagttggtgagtgcattggagtaacagctatctttctgaaagattgttgtattttttaaaatttgcaaactagattatctagaggaaggagaagtcgtaacaaggtttcc
>76f68b4a198aa491e1efe47a63700be6\_1
gcttctaccgattggatgagttggtgagtgaatcggagtaacaggtatctttctgaaagattgttgtattttttaaaatttgcaaactagattatctagaggaaggagaagtcgtaacaaggtttcc
>77206b16b734317734062c8efbfb103c\_1
gctcctaccgattggatgagttggtgagtgaattggagtaacaggtatctttctgaaagattgttgtattttttcaaaatttgcaaactagattatctagaggaaggagaagtcgtaacaaggtttcc
>775d08a5e8196bc1e4a4d19aed5e922a\_1
gctcccaccgattggatgagttggtgagtgaattggagtaacagctatctttctgaaagattgttgtattttttaaaatttgcaaactagattatctagaggaaggagaagtcgtaacaaggtttcc
>77a0e6787102e044da783670136e09ef\_1
gctcctaccgattggatgagttggtgagtgaattggagtaacagctatctttctgaaagattgttgtgttttttaaaatttgcaaactagattatctagaggaaggagaagtcgtaacaaggtttcc
>785094821a7ecc76539804fdf84fa686\_1
gctcctaccgattggatgagttggtgagtgaattggagtaacaggtatctttctgaaagattgttgcatttttaaaatttgcaaactagattatctagaggaaggagaagtcgtaaaaaggtttcc
>78ea93dab19bdb80e4cc8d66b799c561\_1
gctcctaccgattggatgagttggtgagtgaattggagtaacagctatctttctaaaagattgttgtattttttaaaattgcaaactagattatctagaggaaggagaagtcgtaacaaggtttcc
>792820452d73818880ac21cbbb5e9845\_1
gctcctaccgattggatgagttggtgagtgaattggagtaacagctatctttctgaaagattcttgtattttttaaaatttgcaaactagattatctagaggaaggagaagtcgtaacaaggtttcc
>7981ac32409f3f1015469f23453caa9a\_1
gctcctaccgattggatgagttggtgagtgaattggagtaacagctatctttctgaaagattgttgtattttttaaaatttgcaaactagattatcaaaggaaggagaagtcgtaacaaggtttcc
>79c87febfa2b4f877c5532078a0dd8d4\_1
gctcctaccgattggatgagttggtgagtgaattggagtaacaggtatctttctgaaagattgttgcatttaaaatttgcaaactagattatctagaggaaggagaagtcgtaacaaggtttcc
>7a8af99f1090f9da1a9ccd614dca605a\_1
gctcctaccgattggatgagttggtgagtgaattggagtaacaggtatctttctgaaagattgttgtattttttaaaatttgcaaactaaattatcaagaggaaggagaagtcgtaacaaggtttcc
>7af58ae4f6d4dba4a0f7150b13292800\_1
gctcctaccgattggatgagttggtgagtgaattggagtaacaggtatctttctgaaagattgttgtattttttaaatttgcaaactagattatctagaggaaggagaagtcgtaacaaggtttcc
>7b4aff8eb336d5e097121705bed2b82b\_1
gctcctaccgattggatgagttggtgagtgagttggagtaacagctatctttctgaaagattgttgttattttttaaaatttgcaaactagattatctagaggaaggagaagtcgtaacaaggtttcc
>7c25d6f6b82b5fd114ef1f1def0dbfc1\_1
gctcctaccgattggatgagttggtgagtgaattggagtaacagctatctttctgaaagattgttgtattttctaaaatttgcaaactagattatctagaggaaggagaagtcgtaactaaggtttcc
>7c3ccf67d728b93e2b3361f405e6156b\_1
gctcctaccgattggatgagttggtgagtgaattggagtaacaggtatctttctgaaagattgttgcattttaaaatttgcaaactagattatctagatgaaggagaagtcgtaacaaggtttcc
>7c80db7cf66249100de04669da0506bb\_1
gctcctaccgattggatgagttggtgagtgaattggagtaacaggtatctttctgaaagattggtgcattttttaaaatttgcaaactagattatctagaggaaggagaagtcgtaacaaggtttcc
>7d68e7eed9ae77b3dd1e37d652ba2061\_1
gctcctaccaattggatgagttggtgagtgaattggagtaacaggtatctttctgaaagattgttgcatttcttaaaatttgcaaactagattatctagaggaaggagaagtcgtaacaaggtttcc
>7dae405f64d58b797cd45650572383f8\_1
gctcctaccgattggatgagtaggtgagtgaattggagtaacagctatctttctgaaagattgttgtattttttaaaatttgcaaactagattatctagaggaaggagaagtcgtaacaaggtttcc
>7e6037968ffe9b268b759f7337ca0f54\_1
gctcctaccgattggatgagttggtgagtgaattggagtaacaggtatctttctgaaagattgttgcattttttaaaatttgcaaactagttatctagaggaaggagaagtcgtaacaaggtttcc
>7f4cc31fcc1d1fbe01299504bf22d82a\_1
gctcctaccgattggatgagttggtgagtgaattggagtaacaggtatctttctgaaagattgttgtattttgtaaaatttgcaaactagattatctagaggaaggagaagtcgtaacaaggtttcc
>7fd4d1e6045efefd363521e0d64f01e8\_1
gctcctaccgattggatgagttggtgagtgaattggagtaacagctaccttcctgaaagattgttgtatttttttaaaatttgcaaactagattatctagaggaaggagaagtcgtaacaaggtttcc
>7fdb6365887ba283c78ffb9d20a79791\_1
gctcctaccgattggatgagttggtgagtggattggagtaacagctatcttcctgtaagattgttgtatttttttaaaatttgcaaactagattatctagaggaaggagaagtcgtaacaagatttcc
>809881305e0131b71db82e82dc43db0a\_1
gctcctaccgattggatgagttggtgagtgaattggagtaacaggtatctttctgaaagattgttgcattttttaaaatttgcaaactagattatctaaaggaaggagaagtcgtaacaaggtttcc
>80a45fa12d511b4d4a722c1ad721702f\_1
gctcctaccgattggatgagttggtgagtgaattggagtaacaggtagctttctgaaagattgttgtattttttaaaatttgcaaactagattatctagaggaaggagaagtcgtaacaaggtttcc
>81194f58941b1d289c16ed1a918dc4c0\_1
gctcctaccgattggatgagttggtgagtggattggagtaacagctatcttcctgtaagattgttgtattttttaaaatttgcaaactagattatctagaggaaggagaagtcgtaacgaggtttcc
>8123761d37b60838a704c4e17b824d68\_1
gctcctaccgattggatgagttggtgagtgaattggagtaacagctatctttctgaaagattgttgtattttctaaaatttgcaaactagattatctagaggaaggagaagtcataacaaggtttcc
>814a37445ebff3ca193389ffe6d10e8e\_1
gctcctaccgattggatgagttggtgagtgaattgaagtagcagctatctttctgaaagattgttgtattttttaaaatttgcaaactagattatctagaggaaggagaagtcgtaacaaggtttcc
>81be5433b5e5c9932c890dd607eee6d1\_1
gctcctaccgattggatgagttagtgagtgaattggagtaacagctatctttctgaaagattgttgttattttttaaaatttgcaaactagattatctagaggaaggagaagtcgtaacaaggtttcc
>83142816263f2bcd61ee55711195c5b8\_1
gctcctaccgattggatgacttggtgagtgaattggagtaacaggtatctttctgaaagattgttgtattttttaaaatttgcaaactaaattatctagaggaaggagaagtcgtaacaaggtttcc
>83548679e1db68dbe5147bcbb716d5d7\_1
gctcctaccgattggatgagttggtgagtgaattggagtaacaggtatctttctgaaagattgttacattttttaaaatctgcaaactagattatctagaggaaggagaagtcgtaacaaggtttcc
>83694c79408b193863b13720e0b4cd24\_1
gctcctaccgattggatgagttggtgagtgaattggagtaacaggtatctttctgaaagattgttgtattttttaaaatttgcaaactagattatctagaggaaggagaagtcgtaacaaagtttcc
>8444c9a6a891dc51b5be724bf7cfe79e\_1
gctcctactgattggatgagttggtgagtgaattggagtaacagctatctttctgaaagatttttgtattttttaaaatttgcaaactagattatctagaggaaggagaagtcgtaacaaggtttcc
>84bf42d6ccefb041c7e4b945f6c4c594\_1
gctcctaccgattggatgagttggtgagtgaattggagtaacagctatctttctgaaagattgttgtatttttaaaatttgcaaactagattttctagaggaaggagaagtcgtaacaaggtttcc
>84d4297d4ea394e42264b4b692e2b745\_1
gctcctaccgattggatgagttggtgagtgaattggagtaacagctatctttctgaaagattgttgtactttttaaaatttgcaaactagattatctagaggaaggagaagtcataacaaggtttcc
>856d9c810f9c3709a83dbd2cc439bf62\_1
gctcctaccgattggatgagttggtgagtgaattggagtaacaggtatctttctgaaagattgttgcattttataaaatttgcaaactagattatctagaggaaggagaagtcgtaacaaggtttcc
>8790e548fbeefa21ad0d8d37c57ee4dc\_1
gctcctaccgattggatgagttggtgagtgaattggagtaacagctatctttctgaaagattattatattttttaaaatttgcaaactagattatctagaggaaggagaagtcgtaacaaggtttcc
>87ac6214cdcd72544600d91483ef1a84\_1
gctcctaccgattggatgagttggtgagtgaatgggagtaacagctatctttctgaaagattgttgtatttttaaaatttgcaaactagattatctagaggaaggagaagtcgtaacaaggtttcc
>88353332382080f36b1de01b32b95aa6\_1
gctcctaccgattggatgagttggtgagtgaattggagtaacagctatctttctgaaagattgttgtattttttaaaatttgcaaactagattatctagaggaaggagaagtcgtaaaaaggtttcc
>89fc97ce913ed3bdc1bc3c33329dbe0e\_1
gctcctactgattggatgagttggtgagtgaattggagtaacaggtatctttctgaaagattgttgtattttttaaaatttgcaaactagattatctagaggaaggagaagtcgtaacaaggtttcc
>8a42938273608f0e60378e8e79df6f0b\_1
gctcctaccgattggatgagttggtgagtgaattggagtaacaggtatctttctgaaagattgttgcatttataaaatttgcaaactagattatctagaggaaggagaagtcgtaacaaggtttcc
>8a46e4b2020e02a92b032e09f62ffda1\_1
gctcctaccgattggatgagttggtgagtgaattggagtaacagctatctttctgaaagattgttgtattttttaaaatttgcaaactagattatctaggaggaaggagaagtcgtaacaaggtttcc
>8a5d576534631abe06f6996e1d9d990c\_1
gctcctaccgattggatgagttggtgagtgaattggagtaacaggtatctttctgaaagattgttgtattttttaaaatttgcaaactagattatctagaggaaggagtagtcgtaacaaggtttcc
>8b905f9ad9d7b36dd537e97685060479\_1
gctcctaccgattggatgagttggtgagtgcattggagtaacaggtatctttctgaaagattgttgtattttttaaaatttgcaaactagattatctagaggaaggagaagtcgtaacaaggtttcc
>8cf2e593fdd7546340809ab6cc5decc5\_1
gctcctaccgattggatgagttggtgagtgaattggagtaacagccatctttctgaaagattgttgtattttttaaaatttgcaaactagattatctagaggaaggagaagtcgtaacaaggtttcc
>8db277ab17317e5b93808dc39b153609\_1
gctcctaccgattggatgagttggtaagtgaattggagtaacaggtatctttctgaaagattgttgtatttttaaaatttgcaaactagattatctagaggaaggagaagtcgtaacaaggtttcc
>8dd1f3ffb63aa07bcc5027798552c4c4\_1
gctcctaccgattggacgagttggtgagtgaattggagtaacagctatctttctgaaagattgttgtattttttaaaatttgcaaactagattatctagaggaagtaaaagtcgtaacaaggtttcc
>8e33a40e462f3eaae5fc9bcd0da5b820\_1
gctcctaccgattggatgagttggtgagtgaattggagtaacagctatctttctgaaagattgttgtattttctaaaatttgcaaactagattatctagaggaaggagaagtcgtaacaaggtttcc
>8f3358b42499dbf239b64cbce0e46cf1\_1
gctcctaccgattggatgagttggtgagtggattggagtaacagctatcttcctgtaagattgttgtaattttttaaaatgtgcaaactagattatctagaggaaggagaagtcgtaacaaggtttcc
>8f7487f24818cd73c7d1af28f3af6b2d\_1
cgctcctaccgattggatgagttggtgagtgaattggagtaacaggtatctttctgaaagattgttgcattttttaaaatttgcaaactagattatctagaggaaggagaagtcgtaacaaggtttcc
>8f9d6629ff304f512610a0881d945534\_1
gctcctaccgattggatgagttgttgagtgaattggagtaacagctatctttctgaaagattgttgttattttttaaaatttgcaaactagattatctagaggaaggagaagtcgtaacaaggtttcc
>8fd09bc712af320639d4c8304773c3e7\_1
gctcctaccgattggatgagctggtgagtgaattggagtaacaggtatctttctgaaagattgttgtatttttaaaatttgcaaactagattatctagaggaaggagaagtcgtaacaaggtttcc
>8fdb69f822ae1ead0604f757fa24a9dc\_1
gctcctaccgattggatgagttggtgagtgaattggagtaacagctatctttctgaaagattgttgtattttttaaaatttgcaaactagattatctagaggaaggagaagtcgtaacaaggtttgc
>8fde9f0a2f6a43c16af467ee9262efd6\_1
gctcctatcgattggatgagttggtgagtgaattggagtaacaggtatctttctgaaagattgttgcattttttaaaatttgcaaactagattatctagaggaaggagaagtcgtaacaaggtttac
>8fe600ffe16533deee02333c6001d749\_1
gctcctaccgattggatgagttggtgagtgaattggagtaacaggtatctttctgaaagattgttgcattttttaaaatttgcaaactagattatctagaagaaggagaagtcgtaacaaggtttcc
>90629344bf6efcfd4c8ea52577f5a8f4\_1
gctactaccgattggatgaattggtgagtgaattggagtaacagctatctttctgaaagattgttgtattttttaaaatttgcaaactagattatctagaggaaggagaagtcgtaacaaggtttcc
>9083a47f03557320c2c531409d0cd2a0\_1
gctcctaccgattggatgagttggtgagtgaattggagtaacaggtatctttctgaaagattgttgtatttttaaaatttgcaaactagattatctagaggaaggagaagtcgtaacaaggtttcg
>90a60e63566f3937b8295d8d7a4269bd\_1
gctcctaccgattggatgagttggtgagtgaattggagtaacaggtatctttctgaaagattgttgtattttttaaaatttgcaaactagtttatctagaggaaggagaagtcgtaacaaggtttcc
>90cbac54cb13282646624ed2dfc2eb94\_1
gctcctaccgattggatgagttggtgagtgaattggagtaacaggtatctttctgaaagattgttgtattttttaaaatttgcaaactagattatctagagaaaggagaagtcgtaacaaggtttcc
>91c67591af719baff41ee22927a5bb9f\_1
gctcctaccgattggatgagttggtgagtggattggagtaacagctatcttcctgtaagattgttgttaatttttttaaaatttgcaaactagattatctagaggaaggagaagtcgtaacaaggtttcc
>9219e415c451a80f1e1b50b0a4a6ee44\_1
gctcctaccgattggatgagttggtgagtgaattggagtaacaggtatctttctgaaagattgttgcatttttaaaatttggaaactagattatctagaggaaggagaagtcgtaacaaggtttcc
>92c02c73b71b27f82c3adf15ac179631\_1
gctcctaccgattggatgagttggtgagtgaattggagaaacagctatctttctgaaagattgttgtatttttaaaatttgcaaactagattatctagaggaaggagaagtcgtaacaaggtttcc
>93beebd03caac09b6d2613e9aa9e64e5\_1
gctcctaccgattgcatgagttggtgagtgaattggagtaacagctatctttctgaaagattgttatatttttaaaatttgcaaactagattatctagaggaaggagaagtcgtaacaaggtttcc
>93ec1dcbf04b6bb8797241f999741e4a\_1
gctcctaccgattggatgagttggtgagtgaattagagtaacagctatctttctgaaagattgttgtattttttaaaatttgcaaactagattatctagaggaaggagaagtcgtaacaagatttcc
>943731b874be56d95bb33ce3a02af3db\_1
gctcctaccgattggatgagttggtgagtgaattggagtaacagctatctttctgaaagattgttgtatttttaaaatttgcaaactagattatctagaggaatgagaagtcgtaacaaggtttcc
>9465a72cea5cf7dbed7a11811580aa3a\_1
gctcctaccgattggatgagttggtgagtgaattggagtaacaggtatatttctgaaagattgttgtaatttttaaaatttgcaaactagattatctagaggaaggagaagtcgtaacaaggtttcc
>9467bbc60a5c7d6b6e44fa430d411d59\_1
gctcctaccgattggatgagttggtgagtgaattggagtaacagctatctttctgaaagattgttgttattttttaaaatttgcaaactagatatctagaggaaggagaagtcgtaacaaggtttcc
>957ed47a3a711fffa902fd2d398223de\_1
gctcctaccgattggttgagttggtgagtgaattggagtaacaggtatctttctgaaagattgttgcatttttaaaatttgcaaactagattatctagaggaaggagaagtcgtaacaaggtttcc
>972043e881bca74c8d71e52babb5bc2d\_1
gctcctaccgattggatgagttggtgagtgaattggagtaacagctatctttctgaaagattgttgtattttttaaaatttgcaaactagattatctagaggaaggagaattcttaacaaggtttcc
>97f4de2958855641830ed2b8b9778a52\_1
gctcctaccgattggatgagttggtgagtggattggagtaacatctatcttcctgtaagattgttgtattttttaaaatttgcaaactagattatctagaggaaggagaagtcgtaacaaggtttcc
>98956d77dafdd74d7845ac5303cac262\_1
gctcctaccgattggatgagttggtgagtgaattggagtaacaggtatctttctaaaagattgttgtattttttagaatttgcaaactagattatctagaggaaggagaagtcgtaacaaggtttcc
>9b4594811b0a927e8d65e74daafcf412\_1
gttcctaccgattggatgagttggtgagtgaattggagtaacagctatctttctgaaagattgttgtatttttaaaatttgcaaactagattatctagaggaaggagaagtcgtaacaaggtttcc
>9ca0b082bd6fcfca819894e14ff62dd8\_1
gctcctaccgattggatgagttggtgagtaaattggagtaacaggtatctttctgaaagattgttgtattttttaaaatttgcaaactagattatctagaggaaggagaagtcgtaacaaggtttcc
>9d474f4cecab8c965beef7d43c7de89a\_1
gctactaccgattggatgagttggtgaatgaattggagtaacaggtatctttctgaaagattgttgcattttttaaaatttgcaaactagattatctagaggaaggagaagtcgtaacaaggtttcc
>9d90c1d24070c6ed4452812a1d060a4d\_1
gcttctaccgattggatgagttggtgagtgaattggagtaacaggtatctttctgaaagattgttgtattttttaaaatttgcaaactagattatctagaggaaggagaagtcgtaacaagattcc
>9f7ad6eaabec13e2ade69969c27d1412\_1
gcttctgccgattggatgagttggtgagtgaattggagtaacagctatctttctgaaagattgttgtattttttaaaatttgcaaactagattatctagaggaaggagaagtcgtaacaaggtttcc
>9f8fe397f953a97f1259040916c48973\_1
gctcctaccgattggatgagttggtgagtggattggagtaacagctatcttcctgtaacattgttgtatttttaaaatttgcaaactagattatctagaggaaggagaagtcgtaacaaggtttcc
>a0262358804bf3921f7abff97cc3e7fd\_1
gctcctaccgattggatgagttggtgagtgaattggagtaacaggtatctttctgaaagattgttgtattttaaaattttcaaactagattatctagaggaaggagaagtcgtaacaaggtttcc
>a077b4b3f2b44da157c82504b5ba744a\_1
gctactaccgattggatgagttggtgagtgaattggagtaacagctatcttcctgaaagattgttgtattttttaaaatttgcaaactagattatctagaggaaggagaagtcgtaacaaggtttcc
>a0a14cd3ef4b15090010bc9738eb2235\_1
gctcctaccgattggatgagttggtgagtgaattggaagtaacagctatctttctgaaagattgttgtattttttaaaatttgcaaactagattatctagaggaaggagaagtcgtatcaaggtttcc
>a0fc71495ee99f3058f8f4c483aa8fc4\_1
gctcctaccgattggatgagttggtgagtgaattggagtaacaggtatctttctgaaagattgttgcattttttaaaatttgcaaactagattatctacaggaaggagaagtcgtaacaaggttttcc
>a10864adaa553e9210d518761fef252f\_1
gctcctaccgattggatgagttggtgagtgaattggagtaacaggtatctttctgaaagattgttgcattttttaaaatttgcaaactagattatctagaggaaggagaagtcgtaacaacgtttcc
>a1d176cf6d49cab9381889c717a344fa\_1
gctcctaccgattggatgtgttggtgagtgaattggagtaacaggtatctttctgaaagattgttgtattttttaaaatttgcaaactagattatctagaggaaggagaagtcgtaacaaggtttcc
>a37eaf7cd8cf3cd74186197a061131ca\_1
gctcctaccgattggatgagttggtgagtgaattggagtaacaggtatctttctgaaagattgttgcactttttaaaatttgcaaactagattatctagaggaaggagaagtcgtaacaaggtttcc
>a3cdb7131a59c2644c4dfa580809c872\_1
gctcctaccgattggatgagttggtgagtgaattggagtaacaggtatctttctgaaagattgttatattttttaaaatttgcaaactagattatctagaggaaggagaagtcgtaacaaggtttcc
>a3edf062436a3bcbd709971f1beecc70\_1
gctcctaccgattggatgagttggtgagtgaattggagtaacagctatctttctgaaagattgttgtattttttaaaatttgcaaactagattatctagaggaaggagaagtcgtaacaaggtgtcc
>a42ef4722fe4f33d1e02c242baba3a88\_1
ggtcctaccgattggatgagttggtgagtgaattggagtaacaggtatctttctgaaagattgttgcattttttaaaatttgcaaactagattatctagaggaaggagaagtcgtaacaaggtttcc
>a4fb3c542fcdd53f38603cb46c8570b5\_1
gctcctaccgattggatgagttggtgagtgaattggagtaacaggtatctttctgaaagattgttgtagttttttaaaatttgcaaactagattatctagaggaaggagaagtcgtaacaaggtttcc
>a5e7596cc65ef7547ba1731b1348c870\_1
gctcctaccgattggatgagttggtgagtggattggagtaacagctatcttcctgtaagattgttgtatttttttaaaatttgcaaacttgattatctagaggaaggagaagtcgtaacaaggtttcc
>a88621df4ccb1386905aac0896617db1\_1
gctcctaccgattggatgagttggtgagtgaattggagtaacaggtatctttctgaaagattgttgcattttttaaaatttgcaaataagattatctagaggaaggagaagtcgtaacaaggtttcc
>a8c9d1c515cd879e7a1bb234129e8a85\_1
gctcctaccgattggatgagttggtgagtgaattggagtaacagctatctttctgaaagattgtttgtatttttaaaaatttgcaaactagattatctagaggaaggagaagtcgtaacaaggtttcc
>aa32ef6bf5a982cb315b05713bfeb5dd\_1
gctcctaccgattggatgagttggtgagtgaattggagtaacagctatctttctgaaagattgttgtattttaaaatttacaaactagattatctagaggaaggagaagtcgtaacaaggtttcc
>aac895de5946739a4e57947eafdb89e3\_1
gctcctaccgattgggtgagttggtgagtgaattggagtaacaggtatctttctgaaagattgttgcattttttaaaatttgcaaactagattatctagaggaaggagaagtcgtaacaaggtttcc
>aafbd32b4726656011c93034174584ca\_1
gctcctaccgattggatgagttggtgagtgaattggagtaacagctatctttctgaaagattgttgtatttttgaaaatttgcaaactagattatctagaggaaggagaagtcgtaacaaggtttcc
>abb020958b43a23250a92ff2dc3d4410\_1
gctcctaccgattggatgagttggtgggtgaattggagtaacaggtatctttctgaaagattgttgcattttttaaaatttgcaaactaggttatctagaggaaggagaagtcgtaacaaggtttcc
>ac41f5f44d7e37edffde9d2ab46483d5\_1
gctcctaccgattggatgagttggtgagtgaactggagtaacaggtatctttctgaaagattgttgcattttttaaaatttgcaaactagattatctagaggaaggagaagtcgtaacaaggtttcc
>acd03e0ef9562ff9c4b44c3eb51fe964\_1
gctcctaccgattggatgagttggtgagtgaattggagtaacaggtatctttctgaaagattgttgcattttttagaatttgcaaactagattatctagaggaaggagaagtcgtaacaaggtttcc
>ade4063129ebb8c05ad9aacd47d417ae\_1
gcgcctaccgattggatgagttggtgagtgaattggagtaacagctatctttctgaaagattgttgtattttttaaaatttgcaaactagattatctagaggaaggagaagtcgtaacaaggtttcc
>aed61f0889d52f90f1b033a87af71c82\_1
gctcctaccgattggatgagttggtgtagtgaattggagtaacagctatctttctgaaagattgttgtattttttaaaatttgcaaactagattatctagaggaaggagaagtcgtaacaaggtttcc
>aef8f8ce96bd91f673dc1d80b3c71d4b\_1
tctcctaccgattggatgagttggtgagtgaattggagtaacaggtatctttctgaaagattgttgcattttttaaaatttgcaaactagattatctagaggaaggagaagtcgtaacaaggtttcc
>aeffe893ee24d6f6890b2dc629c0a7e8\_1
gctcctaccgattggatgagttggtgagtgaattggagaaacagctatctttctgaaagattgttgtattttttaaaatttgcaaactagattatctagaggaaggagaagtcgtaacaaagtttcc
>afa307d4c288f55d941be2f3cbe9dcf4\_1
gctcctaccgattggatgagttggtgagtgaattggagtaacagctatctttctgaaagattgttgtattttttaaaatttgcaaactagattatttagaggaaggagaaatcgtaacaaggtttcc
>afb840266281473448f37491fac5d2eb\_1
gctcctaccgattggatgagttggtgagtgaattggagtaacagttatctttctgaaagattgttatattttttaaaatttgcaaactagattatctagaggaaggagaagtcgtaacaaggtttcc
>affa68422803cf2c9a219a350c04c027\_1
gctcctaccgattggatgagttggtgagtgaattggagtaacaggtatctttctgaaagattgtttcattttttaaaatttgcaaactagattatctagaggaaggagaagtcgtaacaaggtttcc
>b179419df4d488bb37b23dc20ae29389\_1
gctcctaccgattggatgagttggtgagtgaattggagtaacagatatctttctgaaagattgttgcattttaaaatttgcaaactagattatctagaggaaggagaagtcgtaacaaggtttcc
>b25e3def5c7d3ee6e2eebccd1f9ac463\_1
gctcctaccgattggaagagttggtgagtgaattggagtaacagctatctttctgaaagattgttgtattttttaaaatttgcaaactagattatctagaggaaggagaagtcgtaacaaggtttcc
>b32304347a3c5cb54f88af5e52ac8fca\_1
gctcctaccggttggatgagttggtgagtgaattggagtaacagctatctttctgaaagattgttgtattttttaaaatttgcaaactagattatctagaggaaggagaagtcgtaacaaggtttcc
>b32306e015ed44ab0ab3f76e17d4d8f1\_1
gctcctaccgattggatgagttggtgagggaattggagtaacaggtatctttctgaaagattgttgtatttttaaaatttgcaaactagattatctagaggaaggagaagtcgtaacaaggtttcc
>b35a9ce98e13446ee2294eea6ebcd340\_1
gcttctaccgattggatgagttggtgagtgaattggagtaacaggtatctttctgaaagattgttgtatttttgaaaatttgcaaactagattatctagaggaaggagaagtcgtaacaaggtttcc
>b41d48266c4963a8f56e549d65bdeee8\_1
gctcctaccgattggatgagttggtgagtgaattggagtaacagctatctttctgaatgattgttgtattttttaaaaatttgcaaactagattatctagaggaaggagaagtcgtaacaaggtttcc
>b5092aa0f0b36e7fb08157e6fa7100b8\_1
gctcctaccgattggatgagttggtgagtgaattggagtagcaggtatctttctgaaagattgttgtattttttaaaatttgcaaactagattatctagaggaaggagaagtcgtaacaaggtttcc
>b6a92831b408d2b79a4617cf9900793b\_1
gctcctaccgattggatgagttggtgagtgaattggagtaacaggtatctttctgaaagattgttgtgttttttaaaatttgcaaactagattatctagaggaaggagaagtcgtaacaaggtttcc
>b75927a74ff08e77955531a41bc4f116\_1
gctcctaccgattggatgagttggtgagtgaattggagtaacaggtatctttctgaaagattgttgctttttttaaaatttgcaaactagattatctagaggaaggagaagtcgtaacaaggtttcc
>b762e781ddc40e4715576d86f7a5de22\_1
gctcctaccgattggatgggttggtgagtgaattggagtaacatgtatctttctgaaagattgttgcattttttaaaatttgcaaactagattatctagaggaaggagaagtcgtaacaaggtttcc
>b7a0e5e2a99ed01ad5d9c7ef1cb7c29f\_1
gctcctaccgattggatgagatggtgagtgaattggagtaacagctatctttctgaaagattgttgtatttttaaaatttgcaaactagattatctagaggaaggagaagtcgtaacaaggtttcc
>b7f82da6050d2d81e563f965ab693de2\_1
gctcctaccgattggttgagttggtgagtgaattggagtaacagctatctttctgaaagattgttgattttttaaaatttgcaaactagattatctagaggaaggagaagtcgtaacaaggtttcc
>b84452896ab64c394a07ba880833d798\_1
gctcctaccgattgtatgagttggtgagtgaattggagtaacaggtatctttctgaaagattgttgcattttttaaaatttgcaaactagattatctagaggaaggagaagtcgtaacaaggtttcc
>b8975eeb6b24c1e6e924f1c4bf5124d8\_1
gctcctaccgattggatgagttggtgagtgaattggagtaacagctatctttctgaaagattgttgtattttttaaaatttggaaactagattatctagaggaaggagaagtcgtaacaaggtttcc
>b8e0e98b0dccba2ab301c58ebac85192\_1
gctgctaccgattggatgagttggtgagtgaattggagtaacaggtatctttctgaaagattgttgcattttttaaaatttgcaaactagattatctagaggaaggagaagtcgtaacaaggtttcc
>b936574535f02cf6186fdae680eed06e\_1
gctcctaccgattggatgagttggtgagtgaattggagtaacagctatctttctgaaagattgttgtattttttaaaatatgcaaattagattatctagaggaaggagaagtcgtaacaaggtttcc
>b983cd9014b3f1482a5c981248f882a1\_1
gctcctaccgattggatgagttggtgagtgaattggagtaataggtatctttctgaaagattgttgtattttttaaaatttgcaaactagattatctagaggaaggagaagtcgtaacaaggtttcc
>bbcae68ccb73c8c2632ccaa04112fbe1\_1
gctcctatcgattggatgagttggtgagtgaattggagtaacagctatctttctgaaagattgttgtattttaaaatttgcaaactagattatctagaggaaggagaagtcgtaacaaggtttcc
>bc0350ccb6eebd536d7a17c4f8d9fd8b\_1
gctcctaccgattggatgagttggtgagtgaattggagtaacaggtatctttctgaaagattgttgtattttttaaaatttgcaaactagattatctagaggaaggagaagttgtaacaaggtttcc
>bcbf497f50333ab775072e00ce6abed5\_1
gctcctaccgattggatgagttggtgagtgaattggagtaacagctatctttctgaaagattgttgtatttaataaaatttgcaaactagattatctagaggaaggagaagtcgtaacaaggtttcc
>bcf1828b967a658b7545becd2f674583\_1
gctcctaccgattggatgagttggtgagtgaattggagtaacaggtatctttctgaaagattgttgcattttctaaaatttagcaaactagattatctagaggaaggagaagtcgtaacaaggtttcc
>bd5a8beb60ed2af0fd193f8b3426ee2a\_1
gctcctcccgattggatgagttggtgagtgaattggagtaacagctatctttctgaaagattgttgtatttttaaaatttgcaaactagattatctagaggaaggagaagtcgtaacaaggtttcc
>be0746071d89dd700c4afc02dcea4973\_1
gctcctaccgattggatgagttgttgagtgaattggagtaacagctatctttctgaaagattgttgtattttttaaaatttgcaaactagattatctagaggaaggagaagtcgtaacaaggtttcc
>be41c5ca5c01e9742a9232cf6df0b521\_1
actcctaccgattggatgagttggtgagtgaattggagtaacagctatctttctgaaagattgttatattttttaaaatttgcaaactagattatctagaggaaggagaagtcgtaacaaggtttcc
>bf877aa283fd5ce79a7cd9305be3de03\_1
gctcctaccgattggatgagttggtgagtgaattggagtaacaggtatctttcttaaagattgttgtattttttaaaatttgcaaactagattatctagaggaaggagaagtcgtaacaaggtttcc
>c15c3b0db80a9f74505652342fa97b40\_1
gctcctaccgattggatgagttggtgagtgaattggagtaacaggtatctttctgaaagattgttgtattttttaaaattttagcaaactagattatctagaggaaggagaagtcgtaacaaggtttcc
>c17357a11d1c53699133ae03e5ed1c4d\_1
gctcctaccgattggatgagttggtgagtgaattggagtaacaggtatctttctgaaagattgttgcattttttaaaatttgcaactagattatctagaggaaggagaagtcgtaacaaggtttcc
>c17581f71c0ba01940d1f76211495224\_1
gctcctaccgattggatgagttggtgagtgaattggagtaacagctatctttctgaaagattgttgtatatttaaaaatttgcaaactagattatctagaggaaggagaagtcgtaacaaggtttcc
>c1cda3dcebbc06821e8e5630ca0b6d65\_1
gctcctaccgattggatgagttggtgagtgaattggagtaacagctatctttccgaaagattgttgtattttttaaaatttgcaaactagattatctagaggaaggagaagtcgtaacaaggtttcc
>c3405a6a4e7269ab7468c2d776e9d2b2\_1
gctcctaccgattggatgagttggtgagtgatttggagtaacaggtatctttctgaaagattgttgtattttttaaaatttgcaaactagattatctagaggaaggagaagtcgtaacaaggtttcc
>c3fcfd24d0f5992aa2743f555752bf7e\_1
ggtcctaccgattggatgagttggtgagtgaattggagtaacagctatctttctgaaagattgttgtattttttaaaatttgcaaactaaattatctagaggaaggagaagtcgtaacaaggtttcc
>c462a932afd2f27e0c4f71049e8b9d59\_1
gctcctaccgattggatgagttggtgagtgaattggagtaacaggtatctttctgaaagattgttgtattttttaaaatttgcaaactagatcatctagaggaaggagaagtcgtaacaaggtttcc
>c4730556f962ec2d2f92e839d304e9e2\_1
gctcctaccgattggatgagttggtgagtgaattggagtaacagctatctttctgaaagatggttgtattttttaaaatttgcaaactagattatctagaggaaggagaagtcgtaacaaggtttcc
>c5c217d6ed7a4ecdd520966d16cd42ae\_1
gctcctaccgattggatgagttggtgagtgaattggagtaacaggtatctttctgaaagattgttgcattttttaaaatttgcaaactagattatctagatgaaggagaagtcgtaacaagatttcc
>c82652d82506be4abef5c565217f0637\_1
gctcctaccgattggatgagttggtgagtgaattggagtaacaggtatctttctgaaagaatgttgtattttttaaaatttgcaaactagattatctagaggaaggagaagtcgtaacaaggtttcc
>c89d93f955c8d0626841a70612622b34\_1
gctcctaccgattggatgagttggtgagtgaattggagtaacaggtatctttctgaaagatggttgcattttttaaaatttgcaaattagattatctagaggaaggagaagtcgtaacaaggtttcc
>c8ffb869a5b4ac5c86fcd71a1c202875\_1
gctcctaccgattggatgagttagtgagtgaattggagtaacaggtatctttctgaaagattgttgcatttttaaaatttgcaaactaaattatctagaggaaggagaagtcgtaacaaggtttcc
>c9518ceb9e746ce313161c279af345b9\_1
gctcctaccgattggatgagttggtgagtgaattggagtaacagctatctttctgaaagattgttgtattttttaaaatttgcaaactagattatctagaggaaggaggaagtcgtaacaaggtttcc
>c960806950aadde88e650eb62d29f2f3\_1
gttcctaccgattggatgagttggtgagtgaattggagtaacaggtatctttctgaaagattgttgtattttttaaaatttgcaaactagattatctagaggaaggagaagtcgtaacaaggtttcc
>ca0c47bf82fcf769f04d94954a336574\_1
gctcctaccgattggatgagttggtgagtgaattggagtaacagctatctttctgaaggattgttgtatttttaaaaatttgcaaactagattatctagaggaaggagaagtcgtaacaaggtttcc
>cc09d63e8b57ff9839b4c2fb04339e75\_1
gctcctaccgattggatgagttggtgagtgaattggagtaacaggtatctttctgaaagattgttgtattttctaaaatttgcaaactagattatctagaggaaggagaagtcgtaacaaggtttcc
>cc89a9f3193ba39e922b567cb64e6772\_1
gctcctaccgattggatgagttggtgggtgaattggagtaacaggtatctttctgaaagattgttgtattttttaaaatttgcaaactagattatctagaggaaggagaagtcgtaacaaggtttcc
>cd349015c95670073d3675e3e1118ed8\_1
gctcctaccgattggatgagttggtgagtgaattggagtaacaactatctttctgaaagattgttgtatttttaaaatttgcaaactagattatctagaggaaggagaagtcgtaacaaggtttcc
>cd8067e9599efb94ecee536c50255619\_1
gctcctaccgattggatgagttggtgagtgaattggagtaacagctatctttctgaaagattgttgaatttttttaaaatttgcaaactagattatctagaggaaggagaagtcgtaacaaggtttcc
>cfe6a62b9c3ccf7c67e116d6b27e01eb\_1
gcttctaccgattggatgagttggtgagtgaattggagtaacaggtatctttctgaaagattgttgtagttttttaaaatttgcaaactagattatctagaggaaggagaagtcgtaacaagatttcc
>d02122e715db90505154699b12352f4a\_1
gctcctaccggttggatgagttggtgagtgaattggagtaacaggtatctttctgaaagattgttgtattttttaaaatttgcaaactagattatctagaggaaggagaagtcgtaacaaggtttcc
>d05748a7b25275e00074613c557527c1\_1
gctcctaccgattggatgagttggtgagtgaattggagtaacaggtatctctctgaaagattgttgcattttctaaaatttgcaaactagattatctagaggaaggagaagtcgtaacaaggtttcc
>d064b0b9fd0f4dc29a8a144dc38b9a3e\_1
gctcctaccgattggatgagttggtgagtgaattggagtaacagctatctttctgaaagattgttgtattttttaaaattttcaaactagattatctagaggaaggagaagttgtaacaaggtttcc
>d06f977f2b473a8773f105d302fe0437\_1
gctccttccgattggatgagttggtgagtgaattggagtaacagctatctttctgaaagattgttgttattttttaaaatttgcaaactagattatctagaggaaggagaagtcgtaacaaggtttcc
>d0efca6252d52fffe403dec80912e381\_1
gctcctaccgattggatgagttggtgagtgaattggaataacagctatctttctgaaggattgttgtattttttaaaatttgcaaactagattatctagaggaaggagaagtcgtaacaaggtttcc
>d11794fccb7c1efa32ab859e4159db07\_1
gctcctaccgattggatgagttggtgagtggattggagtagcagctatcttcctgtaagattgttgtatttttaaaatttgcaaactagattatctagaggaaggagaagtcgtaagaaggtttcc
>d11fa4baa3b8ecd0dd86100f151667bc\_1
gctcctaccgattggatgagttggtgagtgaattggagtaacaggtatctttctgaaagattgttgtatttttaaaatttgcaaactagatcatctagaggaaggagaagtcgtaacaaggtttcc
>d18f640e10078091fb915d70b296b560\_1
gctcctaccgattggatgagttggtgagtgaattggagtaacagctatctttctgaaagattgttgtattttttaaaatttacaaactagattatctagaggaaagagaagtcgtaacaaggtttcc
>d3ff938ee4e7b01a8c8a31aad984d6d1\_1
gcttctaccgattggatgagttggtgagtgaattggagtaacagctatctttctgaaagattgttgttattttttaaaatttgcaaactagattatctagcggaaggagaagtcgtaacaaggtttcc
>d437f944cddc2054100ed83e72195576\_1
gctcctaccgattggatgagttggtgagtgaattggagtaacaggtatctttctgaaagattgttgtattttttagaatttgcaaactagattatctagaggaaggagaagtcgtaacaaggtttcc
>d4b99e9024505bc90196ff809bb5722a\_1
gctcctaccgattggatgagttggtgagtgaattggagtaacagctatctttctgaaagattgttgttattttttaaaatttgctaactagattatctagaggaaggagaagtcgtaacaaggtttcc
>d53f5e39178ff8414a1f3cecdf6882c6\_1
gctcctaccgattggatgagttggtgaatgaattggagtaacagctatctttctgaaagattgttgttattttttaaaatttgcaaactagattatctagaggaaggagaagtcgtaacaaggtttcc
>d59959c544d7c25e93086694c9ee2a07\_1
gctcctatcgattggatgagttggtgagtgaattggagtaacagctatctttctgaaagattgttgtatttttaaaatttgcaaactagattatctagaggaaggagaattcgtaacaaggtttcc
>d67f3ca8eabf173b62fcd353fec13b73\_1
gctcctaccgattggatgagttggtgagtgaattggagtaacaggtatctttctgaaagattgttgtatttttttaaaatttgcaaactagattatctagaggaaggagaagtcgtaacaaggttttcc
>d7685a7cdc34a07082f7ca12615d9d13\_1
gctcctaccgattggatgagttggcgagtgaattggagtaacagctatctttctgaaagattgttgtattttttaaaatttgcaaactagattatctagaggaaggagaaggcgtaacaaggtttcc
>d784dac88729e501c9283c144b160d15\_1
gctcctaccgattggatgagttggtgagtgaatttgagtaacaggtatctttctgaaagattgttgcattttttaaaatttgcaaactagattatctagatgaaggagaagtcgtaacaaggtttcc
>d806ead454bc78e9fef8d702e0004a0e\_1
gctcctaccgattggatgagttggtgagtgaattggagtaacagctatctttctgaaagattgttgtattttttaaaatttgcaaactagattatctagaggaaggagaagtcgttaacaaggtttcc
>d95b2a7fbb766573a2a37afba33c7696\_1
gctcctaccgattggatgagttggtgagtgaattggagtaacaggtatctttctgaaagattgttgtattttttaaaatgtgcaaactagattatctagaggaaggagaagtcgtaacaaggtttcc
>d991c7997c83711b85984ea9ebf55b0e\_1
gctcctaccgattggatgagttggtgagtgaattggagtaacaggtatctttctgaaagattgttgcattttttaaaatttgcaaactagattatctagaggaaggagaagtcgtaacaaggttacc
>d9a9622701156aa1436385f0277e02ce\_1
gctcctaccgattggatgagttggtgagtgaattggagtaacagctatctttctgaaagattgttgtatttttaaaatttgcaaactagattatctagaggaaggagaagtcgtaacaagttttcc
>d9ca662d13475418e6749299305270bd\_1
gctcctaccgattggatgagttggtgagtggattggagtaacagctatcttcctgtaagattgttgtattttttaaaatttgcaaactagattatctagaggaaggagaagtcgtaacaagatttcc
>d9dcaea9427546bb889e9a4b53c2e5ad\_1
gctcctaccgattggatgagttggtgagtgaattggagtaacagctatctttctgaaagtttgttgttattttttaaaatttgcaaactagattatctagaggaaggagaagtcgtaacaaggtttcc
>d9ed10ab258e7db684896f4671bc72ec\_1
gctactaccgattggatgagttggtgagtgaattggagtaacagctatctttctgaaagattgttgtatttttaaaatttgcaaactagattatctagaggaaggagaagtcgtaacaaggtttcc
>da45859ba47594d9c40c43a7ce8a7bc5\_1
gctcctaccgattggatgagttggtgagtgaattggagtaacagctatctttctgaaagattgttgtatttttaaaaatttgcaaactagattatctagaggaaggagaagtcgtaacaaggtttct
>daa83adb47b7daef11038439e34b041b\_1
gctcctaccgataggatgagttggtgagtgaattggagtaacagctatctttctgaaagattgttgtattttttaaaatttgcaaactagattatctagaggaaggagaagtcgtaataaggtttcc
>db0871bc916559d8259d44bb3008a574\_1
gctcctaccgattggatgagttggtgagtgaattggagtaacaggtatctttctgaaagattgttgtattttttaaaatttgcaaactagagtatctagaggaaggagaagtcgtaacaaggtttcc
>dc10938338d6f3e481833341a848256a\_1
gctcctaccgattggatgagttggtgagtgaattggagtaacagctatctttctgaaagattgttgtattttttaaaatttgcaaactagattatctagaggaaggagtagtcgtaacatggtttcc
>dcc850660fb5260a66ec1f46d02d456b\_1
gctcctaccgataggatgagttggtgagtgaattggagtaacagctatctttctgaaagattgttgtattttttaaaatttgcaaactagattatctagaggaaggagaagtcgtaacaaggtttcc
>de55032fcddca7aaa360daf0d06740c8\_1
gctcctaccgattggatgagttggtgagtgaattggagtaacaggtatctttctgaaagattgttgtattttttaaaatttgcaaactagattatctagtggaaggagaagtcgtaacaaggtttcc
>de7588808bb94f2695e7472580a98c0e\_1
gctcctaccgattggatgagttggtgagtgaattggagtaacaggtatctttctgaaagattgttgctattttaaaatttgcaaactagattatctagaggaaggagaagtcgtaacaaggtttcc
>de911e6b97291e408a2a9d3310a8ae54\_1
gctcctaccgattggatgagttggtgagtgaattggagtatcaggtatctttctgaaagattgttgcattttttaaaatttgcaaactagattatctagaggaaggagaagtcgtaacaaggtttcc
>df6df02061389f8d3e05ed6ad6dccaa5\_1
gctcctaccgattggatgagttggtgagtggattggagtaacagctatctttctgaaagattgttgttattttttaaaatttgcaaactagattatctagaggaaggagaagtcgtaacaaggtttcc
>df7d47e37027af52003a320387d34f42\_1
gttcctaccgattggatgagttggtgagtgaagtggagtaacaggtatctttctgaaagattgttgtattttttaaaatttgcaaactagattatctagaggaaggagaagtcgtaacaaggtttcc
>df8520978c4be4e68b2498870cb02924\_1
gctcctaccgattggatgagttggtgagtgaattggagtaacaggtatctttctgaaagattgttgcattttttaaaatttgcaaactagattatctagaggaaggagaagtcgtaagaaggtttcc
>dfda5b24c2d29956165d65b5fb5fd71f\_1
gctcctaccgattggatgagttggtgagtgaatttgagtaacaggtatctttctgaaagattgttgcattttttaaaatttgcaaactagattatctagaggaaggagaagtcgtaacaaggtttcc
>e115d3d985e1ec1e342f7596fa59dea2\_1
gctcctaccgattggatgagttggtgagtgaattggagtaacagctatctttctgaaagattgttgtatttttaaaatttgcaaactagattatctagaggaaggagaagtcataacaaggtttcc
>e16501880ff98be04b0350bcf09c7b42\_1
gctcctaccgattggatgagttggtgagtgaattggagtaacagctatctttctgaaagattgttgtattttttaaaatttgcaaactagattatctagaggatggagaagtcgtaacaaggtttcc
>e178abf1d66899e82d3e7d3622ac07bd\_1
gctactaccgattggatgagttggtgagtgcattggagtaacagctatctttctgaaagattgttgtattttttaaaatttgcaaactagattatctagaggaaggagaagtcgtaacaaggtttcc
>e1da51a5b7274848177bf17fdc13353f\_1
gctcataccgattggatgagttggtgagtgaattggagtaacagctatctttctgaaagattgttgtatttttaaaatttgcaaactagattatctagaggaaggagaagtcgtaacaaggtttcc
>e292f120d44fa5cebf0f73d2bb857d8e\_1
gctcctaccgattggatgagttggtgagtgaattggagtaacagctatctttctgaaagattgttgttattttttaaaatttgcaaactagattatctagaggaaggagaagtcgtaacaaggttcc
>e35537a5ce91dcf8bbd830beb702ce3d\_1
gctcctaccgattggatgagttgatgagtgaattggagtaacaggtatctttctgaaagattgttgtatttttaaaatttgcaaactagattatctagaggaaggagaagtcgtaacaaggtttcc
>e3814ba025ac70ba47fd6490d70a61f5\_1
gctcctaccgattggatgagttggtgagtgaattggagtaaaaggtatctttctgaaagattgttgcattttttaaaatttgcaaactagattatctagaggaaggagaagtcgtaacaaggtttcc
>e3a4b8fed0cf2d0094b61d1ca30cf3f5\_1
gctcctaccgattggatgagttggtgagtgaattggagtaacagctatctttctgaaagattgttgtattttttaaaatttgcaaactagataatctagaggaaggagaagtcgtaacaaggtttcc
>e423892b26dcfcd5db77b92c307997b7\_1
gctcctaccgattggatgagttggtcagtgaattggagtaacagctatctttctgaaagattgttgtattttttaaaatttgcaaactagattatctagaggaaggagaagtcgtaacaaggtttcc
>e45467ecdce270e99ed5732976c6f76f\_1
gctcctaccgattggatgagttggtgagtgaattggagtaacagctatatttctgaaagattgttgttattttttaaaatttgcaaactagattatctagaggaaggagaagtcgtaacaaggtttcc
>e83f25f4bf2dbcd07540426e3a724153\_1
gctcctaccgattggatgagttggtgagtgaattggagtaacaggtatctttctgaaagattgttgcatttttttaaaatttgcaaactagattatctagagaaaggagaagtcgtaacaaggttttcc
>e85b411eabeb11653fe182bc4ebb0d83\_1
gctcctaccgattggatgagttggtgagtgaattggagtaacagctatctttctgaaagattgtggttattttttaaaatttgcaaactagattatctagaggaaggagaagtcgtaacaaggtttcc
>e8c0e48cf5351ef8d0d6bc4e3ea03ef7\_1
gctcctaccaattggatgagttggtgagtgaattggagtaacaggtatctttctgaaagattgttgcattttttaaaatttgcaaactagattatctagaggaaggagaagtcataacaaggtttcc
>e9760fb8de6683d3750601bb13374f50\_1
gctcctaccgattggatgagttggtgagtgaattggagtaacaggtatctttctgaaagattgttgtattttttaaaatttgcaaactagattatctagaggaaggagaagtcgtaacaaggtgtcc
>eb10d6c7ed2d9325b6ec592bb6ae54a9\_1
gctcctaccgattggatcagttggtgagtgaattggagtaacaggtatctttctgaaagattgttgtattttttaaaatttgcaaactagattatctagaggaaggagaagtcgtaacaaggtttcc
>ebaa204dd972342a59e0f6b492613835\_1
gctcctaccgattggatgagttggtgagtgaattggagtaacagctatctttctgaaagattgttgtatttttaaaatttgcaaacaagattatctagaggaaggagaagtcgtaacaaggtttcc
>ee11cd4cde09d66e681c13a8082772eb\_1
gctcctaccgattggatgagttggtgagtgaattggagtaacagctatctttctgaaagattgttgtattttttaaaatttgcacactagattatctagaggaaggagaagtcgtaacaaggtttcc
>ee855a58de948b503b6d0f8d64b20570\_1
gctcctaccgattggatgagttggtgagtgaattggagtaacaggtatctttctgaaagattgttgtattttttaaaatttgcaaactagattatctagaggaaggagaattcgtaacaaggtttcc
>eeea1f305ec60fcb0e02d40c8df0b9c7\_1
gctcctaccgattggatgagttggtgagtgaattggagtaacagctattttcctgaaagattgttatatttttttaaaattgcaaactagattatctagaggaaggagaagtcgtaacaaggtttcc
>ef8cd1f1debd522e47866b86a4d35d26\_1
gctcctaccgattggatgagttggtgagtgaattggagtaacagctatctttctgaaagattgttgttatttttaaaatttgcaaactagattatctagaggaaggagaagtcgtaacaaggttttcc
>efe5dd3189eb2f595d92a250a8060194\_1
actcctaccgattggatgagttggtgagtgaattggagtaacagctatctttctgaagattgttgtattttttaaaatttgcaaactagattatctagaggaaggagaagtcgtaacaaggtttcc
>f003e5fd5fbd1c54e5901d34f5a554f4\_1
gctcctaccgattggatgagttggtgagtgaattggagtaacagctatctttctgaaaaattgttgtatttttaaaaatttgcaaactagattatctagaggaaggagaagtcgtaacaaggtttcc
>f02d78d3ddaa4c3db27e9b57b310bdf5\_1
gctcctaccgattggatgagttggtgagtgaattggagtaacagctatctttctgaaagattgttgtattttttaaaatttgcgaactagattatctagaggaaggagaagtcgtaacaaggtttcc
>f10189d8c8dbf7fd34fb22f786bb4c35\_1
gctcctaccgattggatgagttggtgagtgaattggagtaacagctatctttctgaaagattgttgtattttttaaaatctgcaaactagattatctagaggaaggagaaatcgtaacaaggtttcc
>f1558b34cfac08db6ef998b891960fba\_1
gctcctaccgattggatgagttggtgagtgaattggaataacagctatctttctgaaagattgttgtattttttaaaatttgcaaactagattatctagcggaaggagaagtcgtaacaaggtttcc
>f17bc175184cf279ba8e8b99baa111af\_1
gctcctaccgattggatgagttggtgagtgaattggagtaacaggtatctttgtgaaagattgttgcattttttaaaatttgcaaactagattatctagaggaaggagaagtcgtaacaaggtttcc
>f33e44a0ad4f28ef741317e3b8ac51fa\_1
gctcctaccgattggatgagttggtgagtgaattggagtaacaggtatctttctgaaagattgttgtattttttaaaattttgcaaactagattatctagaggaaggagaagtcgtaacaaggtttcc
>f3848aa34bc1957625ba4cf17e2b2b01\_1
gctccttccgattggatgagttggtgagtgaattggagtaacaggtatctttctgaaagattgttgtattttttaaaatttgcaaactagattatctagaggaaggagaagtcgtaacaaggtttcc
>f41847a4306073c59b8e5efe6c78d86b\_1
gctcctaccgattggatgagttggtgagtgaattggagtaacagctatgtttctgaaagattgttgtattttttaaaatttgcaaactagattatctagaggaaggagaagtcgtaacaaggtttcc
>f4372858a28b94b9bb3780cf30958585\_1
gctcctaccgattggatgagttggtgagtgaattggagtaacagctatctttctgaaagattgttgtatttttttaaaatttgcaaactagattatctaaaggaaggagaagtcgtaacaaggtttcc
>f44768315bf3f4adbbd1f15a3d78fee8\_1
gctcctaccgattggatgagttggtgagtgaattggagtaacagctatctttctgaaagattgttgtatttttaaaaatttgcaaactagattatctagaggaagtaaaagtcgtaacaaggtttcc
>f5fbde6085582c02465cd2d989a0a8ec\_1
gctcctaccgattggatgagttggtgagtgaattggagtaacagctatctttctcaaagattgttgtattttttaaaatttgcaaactagattatctagaggaaggagaagtcgtaacaaggtttcc
>f6270905b05cac614b8e8180c3acc5cb\_1
gctcctaccgattggatgagttggtgagttaattggagtaacaggtatctttctgaaagattgttgcattttaaaatttgcaaactagattatctagaggaaggagaagtcgtaacaaggtttcc
>f657f2e55f462af0748338c4376fc67a\_1
gctcctaccgattggatgagttggtgagtgaattggagtaacaggtatctttctgaaagattgttgcattttttaaaatttgcaaactaaattatctagaggaaggagaagtcgtaacaaggtttcc
>f6a85848cb585c9cd9a273e02d4e167e\_1
gctcctaccgattggatgagttggtgagtggattggagtaacagctatcttcctgtaagattgttgtatttttttaaaatttggaaactagattatctagaggaaggagaagtcgtaacaaggtttcc
>f88573f2e2b3b8a3dbbf0a225e23eb34\_1
gctcctaccgattggatgagttggtgagtgaattggagtaacagctatctttctgaaagattgttgtatttttttaaaatttgcaaactagattatctagaggaaggagaagtcataacaaggtttcc
>f99d7fc343a6c6f2d6a021363a4269f4\_1
gctcctaccgattggatgagttggtgagtaaattggagtaacagctatctttctgaaagattgttgtattttttaaaattagcaaactagattatctagaggaaggagaagtcgtaacaaggtttcc
>fb338115941152ea9fbd5d52009420b8\_1
gctcctaccgattggatgagttggtgagtgaattggagtaacagctatctttctgaaagattgttgtattttttaaaatttgcaaactagattatctagaggaaggagaagtcgaaacaaggtttcc
>fb40b13d94e96ee39d5415afdda8b78f\_1
gctcctaccgattggatgagttggtgagtgaattggaataacaggtatctttctgaaagattgttgcattttttaaaatttgcaaactagattatctagaggaaggagaagtcgtaacatggtttcc
>fb6051d70d6c254c6e91a447e8910638\_1
gctcctaccgattggatgagtcggtgagtgaattggagtaacaggtatctttctgaaagattgttgtattttttaaaattttgcaaactagattatctagaggaaggagaagtcgtaacaaggtttcc
>fb83020e05a7950cc0b450b442617fc3\_1
gctcctaccgattggatgagttggtgagtgcattggagtaacagctatctttctgaaagattgttgtattttttaaagtttgcaaactagattatctagaggaaggagaagtcgtaacaaggtttcc
>fbbdbad16e878693bda28f60bfd21e72\_1
gctcctaccgattggatgagttggtgagtgaattggagtaacagctaacttcctgaaagattgttgtatttttaaaatttgcaaactagattatctagaggaaggagaagtcgtaacaaggtttcc
>fbe0f8d5918a85df995921f6a7ec02e0\_1
gctcctaccgattggatgagttggtgagtgaattggagtaacagctatctttctgaaagattgttctattttttaaaatttgcaaactagattatctagaggaaggagaagtcgtaacaaggtttcc
>fc1668c1fc8d0cce23b19d731db092c4\_1
gctcctaccgattggatgagttggtgagtgaattggagtaacaggtatctttctgaaagattgttgcattttttaaaatttgcaaactagatcatctagaggaaggagaagtcgtaacaaggtttcc
>fda7e86f3488df92c09e62e395917654\_1
gctcctactgattggatgagttggtgagtgaattggagtaacagctatctttctgaaagattgttgtattttttaaaatttgcaaactagattatctagaggaaagagaagtcgtaacaaggtttcc
>fe0d7bac7eb95a07fed3c1cc9a444779\_1
gctcctaccgattggatgagttggtgagtgaattggagtaacagctatctttctgaaagattgttgtattttttaaaactttgcaaactagattatctagaggaaggagaagtcgtaacaaggtttcc

#### 3.3.2 BioMarKs 18S rRNA V4 OTU 54 fasta file

To simplify replication of our results, we provide the OTU 54 in fasta
format:

>cf300107400178f2895f501833126e35\_1153
agctccaatagcgtatattaaagttgttgcagttaaaaagctcgtagttggatttcggagtgggccagttggtccgccgcaaggtgtgttactgactggtttgctcttcttcgcaaagactgcgtgtgctctttattgagtgtgcgtaggatttacgacgtttactttgaaaaaattagagtgttcaaagcaggctattgcttgaatacatgagcatggaataatggaataggattttggtcccattttgttggtttctaggaccgaagtaatgattaagagggacaattgggggcatccgtatttcgttgtcagaggtgaaattcttggatttacgaaagacgaacaactgcgaaagcacttgccaagagtgttttca
>834a8eff393e23d3b887ee35b0556568\_1112
agctccaatagcgtatattaaagttgttgcagttaaaaagctcgtagttggatttcggagtgggcccgttggtccgccgcaaggtgtgttactgactggtctgctcttcttcgcaaagactgcgtgtgctctttgttgagtgtgcgtaggatttacgacgtttactttgaaaaaattagagtgttcaaagcaggctattgcttgaatacatgagcatggaataatggaataggactttggtcccattttgttggtttctaggaccgaagtaatgattaagagggacaattgggggcatccgtatttcgttgtcagaggtgaaattcttggatttacgaaagacgaacaactgcgaaagcacttgccaagagtgttttca
>7d8fab98aa0ff020eefb0cceabb38964\_815
agctccaatagcgtatattaaagttgttgcagttaaaaagctcgtagttggatttcggagtgggccagttggtccgccgcaaggtgtgttactgactggtctgctcttcttcgcaaagactgcgtgtgctctttgttgagtgtgcgtaggatttacgacgtttactttgaaaaaattagagtgttcaaagcaggctattgcttgaatacatgagcatggaataatggaataggactttggtcccattttgttggtttctaggaccgaagtaatgattaagagggacaattgggggcatccgtatttcgttgtcagaggtgaaattcttggatttacgaaagacgaacaactgcgaaagcatttgccaagagtgttttca
>64ed1bf0bddf9cc2c2e24a7351da1de8\_273
agctccaatagcgtatattaaagttgttgcagttaaaaaagctcgtagttggatttcggagtgggccagttggtccgccgcaaggtgtgttactgactggtttgctcttcttcgcaaagactgcgtgtgctctttattgagtgtgcgtaggatttacgacgtttactttgaaaaaattagagtgttcaaagcaggctattgcttgaatacatgagcatggaataatggaataggattttggtcccattttgttggtttctaggaccgaagtaatgattaagagggacaattgggggcatccgtatttcgttgtcagaggtgaaattcttggatttacgaaagacgaacaactgcgaaagcacttgccaagagtgttttca
>04bce1ff6c66a14329d6cc0a3d404d7a\_192
agctccaatagcgtatattaaagttgttgcagttaaaaagctcgtagttggatttcggagtgggcccgttggtccgccgcaaggtgtgttactgactggtctgctcttcttcgcaaagactgcgtgtgctctttgttgagtgtgcgtaggatttacgacgtttactttgaaaaaattagagtgttcaaagcaggctattgcttgaatacatgagcatggaataatggaataggactttggtcccattttgttggtttctaggaccgaagtaatgattaagagggacaattgggggcatccgtatttcgttgtcagaggtgaaatttcttggatttacgaaagacgaacaactgcgaaagcacttgccaagagtgttttca
>2436f9e3d5a43bed39d95a7e8f26bc20\_159
agctccaatagcgtatattaaagttgttgcagttaaaaagctcgtagttggatttcggagtgggccagttggtccgccgcaaggtgtgttactgactggtctgctcttcttcgcaaagactgcgtgtgctctttgttgagtgtgcgtaggatttacgacgtttactttgaaaaaattagagtgttcaaagcaggctattgcttgaatacatgagcatggaataatggaataggactttggtcccattttgttggtttctaggaccgaagtaatgattaagagggacaattgggggcatccgtatttcgttgtcagaggtgaaattcttggatttacgaaagacgaacaactgcgaaaagcatttgccaagagtgttttca
>6e1917a02de5e3c255b5ed110800e4f7\_123
agctccaatagcgtatattaaagttgttgcagttaaaaagctcgtagttggatttcggagtgggcccgttggtccgccgcaaggtgtgttactgactggtctgctcttcttcgcaaagactgcgtgtgctctttgttgagtgtgcgtaggatttacgacgtttactttgaaaaaattagagtgttcaaagcaggctattgcttgaatacatgagcatggaataatggaataggactttggtcccattttgttggtttctaggaccgaagtaatgattaagagggacaattgggggcatccgtatttcgttgtcagaggtgaaattcttggatttacgaaagacgaacaactgcgaaaagcacttgccaagagtgttttca
>140f5cab49eca4ffbd1d7fd9623e78fd\_112
agctccaatagcgtatattaaagttgttgcagttaaaaagctcgtagttggatttcggagtgggcccgttggtccgccgcaaggtgtgttactgactggtctgctcttcttcgcaaagactgcgtgtgctctttgttgagtgtgcgtaggatttacgacgtttactttgaaaaaaattagagtgttcaaagcaggctattgcttgaatacatgagcatggaataatggaataggactttggtcccattttgttggtttctaggaccgaagtaatgattaagagggacaattgggggcatccgtatttcgttgtcagaggtgaaattcttggatttacgaaagacgaacaactgcgaaagcacttgccaagagtgttttca
>e83fa06fe7a9b83c11922ee64672d2b0\_111
agctccaatagcgtatattaaagttgttgcagttaaaaagctcgtagttggatttcggagtgggccagttggtccgccgcaaggtgtgttactgactggtctgctcttcttcgcaaagactgcgtgtgctctttgttgagtgtgcgtaggatttacgacgtttactttgaaaaaattagagtgttcaaagcaggctattgcttgaatacatgagcatggaataatggaataggactttggtcccattttgttggtttctaggaccgaagtaatgattaagagggacaattgggggcatccgtatttcgttgtcagaggtgaaattcttggatttacgaaagacgaacaactgcgaaagcacttgccaagagtgttttca
>28b950f7ffba88afdb88b91096929256\_108
agctccaatagcgtatattaaagttgttgcagttaaaaagctcgtagttggatttcggagtgggccagttggtccgccgcaaggtgtgttactgactggtctgctcttcttcgcaaagactgcgtgtgctctttgttgagtgtgcgtaggatttacgacgtttactttgaaaaaaattagagtgttcaaagcaggctattgcttgaatacatgagcatggaataatggaataggactttggtcccattttgttggtttctaggaccgaagtaatgattaagagggacaattgggggcatccgtatttcgttgtcagaggtgaaattcttggatttacgaaagacgaacaactgcgaaagcatttgccaagagtgttttca
>ce58d381f8b8e9b8aae1ce1748f140e2\_102
agctccaatagcgtatattaaagttgttgcagttaaaaaagctcgtagttggatttcggagtgggcccgttggtccgccgcaaggtgtgttactgactggtctgctcttcttcgcaaagactgcgtgtgctctttgttgagtgtgcgtaggatttacgacgtttactttgaaaaaattagagtgttcaaagcaggctattgcttgaatacatgagcatggaataatggaataggactttggtcccattttgttggtttctaggaccgaagtaatgattaagagggacaattgggggcatccgtatttcgttgtcagaggtgaaattcttggatttacgaaagacgaacaactgcgaaagcacttgccaagagtgttttca
>5fda227c7fc6357a12af4ae237cd38e7\_89
agctccaatagcgtatattaaagttgttgcagttaaaaagctcgtagttggatttcggagtgggccagttggtccgccgcaaggtgtgttactgactggtttgctcttcttcgcaaagactgcgtgtgctctttattgagtgtgcgtaggatttacgacgtttactttgaaaaaattagagtgttcaaagcaggctattgcttgaatacatgagcatggaataatggaataggattttggtcccattttgttggtttctaggaccgaagtaatgattaagagggacaattggggcatccgtatttcgttgtcagaggtgaaattcttggatttacgaaagacgaacaactgcgaaagcacttgccaagagtgttttca
>9e364663220a8acf3e4c7dd6e34a69a3\_87
agctccaatagcgtatattaaagttgttgcagttaaaaagctcgtagttggatttcggagtgggccagttggtccgccgcaaggtgtgttactgactggtctgctcttcttcgcaaagactgcgtgtgctctttgttgagtgtgcgtaggatttacgacgtttactttgaaaaaattagagtgttcaaagcaggctattgcttgaatacatgagcatggaataatggaataggactttggtcccattttgttggtttctaggaccgaagtaatgattaagagggacaattggggcatccgtatttcgttgtcagaggtgaaattcttggatttacgaaagacgaacaactgcgaaagcatttgccaagagtgttttca
>63826e7bcd6cb46c8efd672915c6e923\_84
agctccaatagcgtatattaaagttgttgcagttaaaaagctcgtagttggatttcggagtgggccagttggtccgccgcaaggtgtgttactgactggtttgctcttcttcgcaaagactgcgtgtgctctttattgagtgtgcgtaggatttacgacgtttactttgaaaaaattagagtgttcaaagcaggctattgcttgaatacatgagcatggaataatggaataggattttggtcccattttgttggtttctaggaccgaagtaatgattaagagggacaattgggggcatccgtatttcgttgtcagaggtgaaattcttggatttacgaaagacgaacaactgcgaaagcacttgccaagagtgtttca
>29a829050299e6325bed164768757e0e\_74
agctccaatagcgtatattaaagttgttgcagttaaaaagctcgtagttggatttcggagtgggccagttggtccgccgcaaggtgtgttactgactggtttgctcttcttcgcaaagactgcgtgtgctctttattgagtgtgcgtaggatttacgacgtttactttgaaaaaattgagtgttcaaagcaggctattgcttgaatacatgagcatggaataatggaataggattttggtcccattttgttggtttctaggaccgaagtaatgattaagagggacaattgggggcatccgtatttcgttgtcagaggtgaaattcttggatttacgaaagacgaacaactgcgaaagcacttgccaagagtgttttca
>d55cf2e2b1afe7b42a07955659a84761\_72
agctccaatagcgtatattaaagttgttgcagttaaaaagctcgtagttggatttcggagtgggccagttggtccgccgcaaggtgtgttactgactggtttgctcttcttcgcaaagactgcgtgtgctctttattgagtgtgcgtaggatttacgacgtttactttgaaaaaaattagagtgttcaaagcaggctattgcttgaatacatgagcatggaataatggaataggattttggtcccattttgttggtttctaggaccgaagtaatgattaagagggacaattgggggcatccgtatttcgttgtcagaggtgaaattcttggatttacgaaagacgaacaactgcgaaagcacttgccaagagtgttttca
>17d2490fe855503e2e2b474c46a285a1\_65
agctccaatagcgtatattaaagttgttgcagttaaaaagctcgtagttggatttcggagtgggccagttggtccgccgcaaggtgtgttactgactggtttgctcttcttcgcaaagactgcgtgtgctctttgttgagtgtgcgtaggatttacgacgtttactttgaaaaaattagagtgttcaaagcaggctattgcttgaatacatgagcatggaataatggaataggactttggtcccattttgttggtttctaggaccgaagtaatgattaagagggacaattgggggcatccgtatttcgttgtcagaggtgaaattcttggatttacgaaagacgaacaactgcgaaagcacttgccaagagtgttttca
>187d5fc94047cbe9083f65d21377e658\_62
agctccaatagcgtatattaaagttgttgcagttaaaaaagctcgtagttggatttcggagtgggccagttggtccgccgcaaggtgtgttactgactggtctgctcttcttcgcaaagactgcgtgtgctctttgttgagtgtgcgtaggatttacgacgtttactttgaaaaaattagagtgttcaaagcaggctattgcttgaatacatgagcatggaataatggaataggactttggtcccattttgttggtttctaggaccgaagtaatgattaagagggacaattgggggcatccgtatttcgttgtcagaggtgaaattcttggatttacgaaagacgaacaactgcgaaagcatttgccaagagtgttttca
>b51750e153b4fff69febb9043933a10f\_60
agctccaatagcgtatattaaagttgttgcagttaaaaagctcgtagttggatttcggagtgggccagttggtccgccgcaaggtgtgttactgactggtctgctcttcttcgcaaagactgcgtgtgctctttgttgagtgtgcgtaggatttacgacgtttactttgaaaaaattagagtgttcaaagcaggctattgcttgaatacatgagcatggaataatggaataggactttggtcccattttgttggtttctaggaccgaagtaatgattaagagggacaattgggggcatccgtatttcgttgtcagaggtgaaatttcttggatttacgaaagacgaacaactgcgaaagcatttgccaagagtgttttca
>b176c7e34ea6c5bdbcfa7e658c85e09a\_54
agctccaatagcgtatattaaagttgttgcagttaaaaaagctcgtagttggatttcggagtgggccagttggtccgccgcaaggtgtgttactgactggtttgctcttcttcgcaaagactgcgtgtgctctttattgagtgtgcgtaggatttacgacgtttactttgaaaaaattagagtgttcaaagcaggctattgcttgaatacatgagcatggaataatggaataggattttggtcccattttgttggtttctaggaccgaagtaatgattaagagggacaattgggggcatccgtatttcgttgtcagaggtgaaattcttggatttacgaaagacgaacaactgcgaaagcacttgccaagagtgtttca
>8049530f2aefcc1961562f77609045b0\_48
agctccaatagcgtatattaaagttgttgcagttaaaaagctcgtagttggatttcggagtgggcccgttggtccgccgcaaggtgtgttactgactggtctgctcttcttcgcaaagactgcgtgtgctctttgttgagtgtgcgtaggatttacgacgtttactttgaaaaaattagagtgttcaaagcaggctattgcttgaatacatgagcatggaataatggaataggactttggtcccattttgttggtttctaggaccgaagtaatgattaagagggacaattggggcatccgtatttcgttgtcagaggtgaaattcttggatttacgaaagacgaacaactgcgaaagcacttgccaagagtgttttca
>0bb359d3a919fa70f63aad18f2edd3e0\_41
agctccaatagcgtatattaaagttgttgcagttaaaaaagctcgtagttggatttcggagtgggccagttggtccgccgcaaggtgtgttactgactggtttgctcttcttcgcaaagactgcgtgtgctctttattgagtgtgcgtaggatttacgacgtttactttgaaaaaattagagtgttcaaagcaggctattgcttgaatacatgagcatggaataatggaataggattttggtcccattttgttggtttctaggaccgaagtaatgattaagagggacaattggggcatccgtatttcgttgtcagaggtgaaattcttggatttacgaaagacgaacaactgcgaaagcacttgccaagagtgttttca
>30296ee28cb13dbb521612906ab5d8b4\_41
agctccaatagcgtatattaaagttgttgcagttaaaaagctcgtagttggatttcggagtgggcccgttggtccgccgcaaggtgtgttactgactggtctgctcttcttcgcaaagactgcgtgtgctctttgttgagtgtgcgtaggatttacgacgtttactttgaaaaaattgagtgttcaaagcaggctattgcttgaatacatgagcatggaataatggaataggactttggtcccattttgttggtttctaggaccgaagtaatgattaagagggacaattgggggcatccgtatttcgttgtcagaggtgaaattcttggatttacgaaagacgaacaactgcgaaagcacttgccaagagtgttttca
>f5f2cf21eedb11f7579994ca98ff7842\_35
agctccaatagcgtatattaaagttgttgcagttaaaaagctcgtagttggatttcggagtgggccagttggtccgccgcaaggtgtgttactgactggtttgctcttcttcgcaaagactgcgtgtgctctttattgagtgtgcgtaggatttacgacgtttactttgaaaaaattagagtgttcaaagcaggctattgcttgaatacatgagcatggaataatggaataggattttggtcccatttttgttggtttctaggaccgaagtaatgattaagagggacaattgggggcatccgtatttcgttgtcagaggtgaaattcttggatttacgaaagacgaacaactgcgaaagcacttgccaagagtgttttca
>f68ef4bd0897ab53bd01239762af0c0f\_34
agctccaatagcgtatattaaagttgttgcagttaaaaagctcgtagttggatttcggagtgggccagttggtccgccgcaaggtgtgttactgactggtttgctcttcttcgcaaagactgcgtgtgctctttattgagtgtgcgtaggatttacgacgtttactttgaaaaaattagagtgttcaaagcaggctattgcttgaatacatgagcatggaataatggaataggattttggtcccattttgtggtttctaggaccgaagtaatgattaagagggacaattgggggcatccgtatttcgttgtcagaggtgaaattcttggatttacgaaagacgaacaactgcgaaagcacttgccaagagtgttttca
>f4d66a7868fcf2d65e0fa6312bb32d2e\_32
agctccaatagcgtatattaaagttgttgcagttaaaaagctcgtagttggatttcggagtgggcccgttggtccgccgcaaggtgtgttactgactggtctgctcttcttcgcaaagactgcgtgtgctctttgttgagtgtgcgtaggatttacgacgtttactttgaaaaaattagagtgttcaaagcaggctattgcttgaatacatgagcatggaataatggaataggactttggtcccattttgttggtttctaggaccgaagtaatgattaagagggacaattgggggcatccgtatttcgttgtcagaggtgaaattcttggatttacgaaagacgaacaactgcgaaagcatttgccaagagtgttttca
>b5fe7a3c4c951655a0608303c933b26c\_31
agctccaatagcgtatattaaagttgttgcagttaaaaagctcgtagttggatttcggagtgggcccgttggtccgccgcaaggtgtgttactgactggtctgctcttcttcgcaaagactgcgtgtgctctttgttgagtgtgcgtaggatttacgacgtttactttgaaaaaattagagtgttcaaagcaggctattgcttgaatacatgagcatggaataatggaataggactttggtcccattttgttggtttctaggaccgaagtaatgattaagagggacaattgggggcatccgtatttcgttgtcagaggtgaaattctttggatttacgaaagacgaacaactgcgaaagcacttgccaagagtgttttca
>0e438643ee22805ea7e466f6ba2482c4\_29
agctccaatagcgtatattaaagttgttgcagttaaaaaagctcgtagttggatttcggagtgggccagttggtccgccgcaaggtgtgttactgactggtttgctcttcttcgcaaagactgcgtgtgctctttattgagtgtgcgtaggatttacgacgtttactttgaaaaaaattagagtgttcaaagcaggctattgcttgaatacatgagcatggaataatggaataggattttggtcccattttgttggtttctaggaccgaagtaatgattaagagggacaattgggggcatccgtatttcgttgtcagaggtgaaattcttggatttacgaaagacgaacaactgcgaaagcacttgccaagagtgttttca
>dc1b194f0bcfc3929959f245e6dc9b07\_29
agctccaatagcgtatattaaagttgttgcagttaaaaagctcgtagttggatttcggagtgggccagttggtccgccgcaaggtgtgttactgactggtttgctcttcttcgcaaagactgcgtgtgctctttattgagtgtgcgtaggatttacgacgtttactttgaaaaaattagagtgttcaaagcaggctattgcttgaatacatgagcatggaataatggaataggatttggtcccattttgttggtttctaggaccgaagtaatgattaagagggacaattgggggcatccgtatttcgttgtcagaggtgaaattcttggatttacgaaagacgaacaactgcgaaagcacttgccaagagtgttttca
>436f836d26c7eaa86b04a529e5f05e3b\_27
agctccaatagcgtatattaaagttgttgcagttaaaaagctcgtagttggatttcggagtgggccagttggtccgccgcaaggtgtgttactgactggtctgctcttcttcgcaaagactgcgtgtgctctttgttgagtgtgcgtaggatttacgacgtttactttgaaaaaattagagtgttcaaagcaggctattgcttgaatacatgagcatggaataatggaataggactttggtcccattttgttggtttctaggaccgaagtaatgattaagagggacaattgggggcatccgtatttcgttgtcagaggtgaaattcttggatttacgaaagacgaacaactgcgaaagcatttgccaagagtgtttca
>89b3c193d5eb6b7fe9ddc26ff8dbd0df\_26
agctccaatagcgtatattaaagttgttgcagttaaaaagctcgtagttggatttcggagtgggcccgttggtccgccgcaaggtgtgttactgactggtctgctcttcttcgcaaagactgcgtgtgctctttgttgagtgtgcgtaggatttacgacgtttactttgaaaaaattagagtgttcaaagcaggctattgcttgaatacatgagcatggaataatggaataggactttggtcccattttgttggtttctaggaccgaagtaatgattaagagggacaattgggggcatccgtatttcgttgtcagaggtgaaattcttggatttacgaaagacgaacaactgcgaaagcacttgccaagagtgtttca
>70fcd72c5a99608ee32826ea0a6cd715\_23
agctccaatagcgtatattaaagttgttgcagttaaaaagctcgtagttggatttcggagtgggccagttggtccgccgcaaggtgtgttactgactggtttgctcttcttcgcaaagactgcgtgtgctctttattgagtgtgcgtaggatttacgacgtttactttgaaaaaattagagtgttcaaagcaggctattgcttgaatacatgagcatggaataatggaataggattttggtcccattttgttggtttctaggaccgaagtaatgattaagagggacaattggggcatccgtatttcgttgtcagaggtgaaattcttggatttacgaaagacgaacaactgcgaaagcacttgccaagagtgtttca
>cee1a8adedc4577641a3cd71b76688e7\_22
agctccaatagcgtatattaaagttgttgcagttaaaaagctcgtagttggatttcggagtgggccagttggtccgccgcaaggtgtgttactgactggtctgctcttcttcgcaaagactgcgtgtgctctttgttgagtgtgcgtaggatttacgacgtttactttgaaaaaattgagtgttcaaagcaggctattgcttgaatacatgagcatggaataatggaataggactttggtcccattttgttggtttctaggaccgaagtaatgattaagagggacaattgggggcatccgtatttcgttgtcagaggtgaaattcttggatttacgaaagacgaacaactgcgaaagcatttgccaagagtgttttca
>a6d4e63a3667e4447414469deb62910e\_21
agctccaatagcgtatattaaagttgttgcagttaaaaagctcgtagttggatttcggagtgggccagttggtccgccgcaaggtgtgttactgactggtctgctcttcttcgcaaagactgcgtgtgctctttgttgagtgtgcgtaggatttacgacgtttactttgaaaaaattagagtgttcaaagcaggctattgcttgaatacatgagcatggaataatggaataggactttggtcccattttgttggtttctaggaccgaagtaatgattaagagggacaattgggggcatccgtatttcgtgtcagaggtgaaattcttggatttacgaaagacgaacaactgcgaaagcatttgccaagagtgttttca
>5b483703e8e9c4e55d1b2cb74e9b36d2\_20
agctccaatagcgtatattaaagttgttgcagttaaaaaagctcgtagttggatttcggagtgggccagttggtccgccgcaaggtgtgttactgactggtctgctcttcttcgcaaagactgcgtgtgctctttgttgagtgtgcgtaggatttacgacgtttactttgaaaaaaattagagtgttcaaagcaggctattgcttgaatacatgagcatggaataatggaataggactttggtcccattttgttggtttctaggaccgaagtaatgattaagagggacaattgggggcatccgtatttcgttgtcagaggtgaaattcttggatttacgaaagacgaacaactgcgaaagcatttgccaagagtgttttca
>06f1c02d3ca92112bb0041a148241350\_19
agctccaatagcgtatattaaagttgttgcagttaaaaagctcgtagttggatttcggagtgggcccgttggtccgccgcaaggtgtgttactgactggtctgctcttcttcgcaaagactgcgtgtgctctttgttgagtgtgcgtaggatttacgacgtttactttgaaaaaattagagtgttcaaagcaggctattgcttgaatacatgagcatggaataatggaataggactttggtcccattttgttggtttctaggaccgaagtaatgattaagagggacaattgggggcatccgtatttcgttgtcagaggtgaaattcttggatttacgaaagacgaacaactgcgaaagcacttgccaagagtgtttttca
>5bbd96b7fa3e7b8f57e347f56c1d8ddc\_19
agctccaatagcgtatattaaagttgttgcagttaaaaaagctcgtagttggatttcggagtgggccagttggtccgccgcaaggtgtgttactgactggtttgctcttcttcgcaaagactgcgtgtgctctttattgagtgtgcgtaggatttacgacgtttactttgaaaaaattgagtgttcaaagcaggctattgcttgaatacatgagcatggaataatggaataggattttggtcccattttgttggtttctaggaccgaagtaatgattaagagggacaattgggggcatccgtatttcgttgtcagaggtgaaattcttggatttacgaaagacgaacaactgcgaaagcacttgccaagagtgttttca
>b547df2ae0b32b96a2279c0f74e3e8d1\_18
agctccaatagcgtatattaaagttgttgtggttaaaaagctcgtagttggatttcggagtgggcccgttggtccgccgcaaggtgtgttactgactggtctgctcttcttcgcaaagactgcgtgtgctctttgttgagtgtgcgtaggatttacgacgtttactttgaaaaaattagagtgttcaaagcaggctattgcttgaatacatgagcatggaataatggaataggactttggtcccattttgttggtttctaggaccgaagtaatgattaagagggacaattgggggcatccgtatttcgttgtcagaggtgaaattcttggatttacgaaagacgaacaactgcgaaagcacttgccaagagtgttttca
>a89f44babe0e5f2ef570feffb4b85c8f\_17
agctccaatagcgtatattaaagttgttgcagttaaaaagctcgtagttggatttcggagtgggccagttggtccgccgcaaggtgtgttactgactggtttgctcttcttcgcaaagactgcgtgtgctctttattgagtgtgcgtaggatttacgacgtttactttgaaaaaattagagtgttcaaagcaggctattgcttgaatacatgagcatggaataatggaataggattttggtcccattttgttggtttctaggaccgaagtaatgattaagagggacaattgggggcatccgtatttcgttgtcagaggtgaaattcttggatttacgaaagacgaacaactgcgaaagcacttgccaagagtgtttttca
>dd7196ea8b760395477f4d1b5025874c\_17
agctccaatagcgtatattaaagttgttgcagttaaaaagctcgtagttggatttcggagtgggccagttggtccgccgcaaggtgtgttactgactggtctgctcttcttcgcaaagactgcgtgtgctctttgttgagtgtgcgtaggatttacgacgtttactttgaaaaaaattgagtgttcaaagcaggctattgcttgaatacatgagcatggaataatggaataggactttggtcccattttgttggtttctaggaccgaagtaatgattaagagggacaattgggggcatccgtatttcgttgtcagaggtgaaattcttggatttacgaaagacgaacaactgcgaaagcatttgccaagagtgttttca
>04e006c6a32b904f8f558c8d8a0dd897\_16
agctccaatagcgtatattaaagttgttgcagttaaaaagctcgtagttggatttcggagtgggccagttggtccgccgcaaggtgtgttactgactggtctgctcttcttcgcaaagactgcgtgtgctctttgttgagtgtgcgtaggatttacgacgtttactttgaaaaaaattagagtgttcaaagcaggctattgcttgaatacatgagcatggaataatggaataggactttggtcccattttgttggtttctaggaccgaagtaatgattaagagggacaattggggcatccgtatttcgttgtcagaggtgaaattcttggatttacgaaagacgaacaactgcgaaagcatttgccaagagtgttttca
>08d7307064849f1229f1f476a976dfd7\_16
agctccaatagcgtatattaaagttgttgcagttaaaaagctcgtagttggatttcggagtgggcccgttggtccgccgcaaggtgtgttactgactggtctgctcttcttcgcaaagactgcgtgtgctctttgttgagtgtgcgtaggatttacgacgtttactttgaaaaaaattgagtgttcaaagcaggctattgcttgaatacatgagcatggaataatggaataggactttggtcccattttgttggtttctaggaccgaagtaatgattaagagggacaattgggggcatccgtatttcgttgtcagaggtgaaattcttggatttacgaaagacgaacaactgcgaaagcacttgccaagagtgttttca
>134150abdba3bdf77c49d7d6706b2ac4\_16
agctccaatagcgtatattaaagttgttgcagttaaaaagctcgtagttggatttcggagtgggccagttggtccgccgcaaggtgtgttactgactggtttgctcttcttcgcaaagactgcgtgtgctctttattgagtgtgcgtaggatttacgacgtttactttgaaaaaattagagtgttcaaagcaggctattgcttgaatacatgagcatggaataatggaataggatttttggtcccattttgttggtttctaggaccgaagtaatgattaagagggacaattgggggcatccgtatttcgttgtcagaggtgaaattcttggatttacgaaagacgaacaactgcgaaagcacttgccaagagtgttttca
>a462ecf13fe377ca5b54f5bba8d61014\_16
agctccaatagcgtatattaaagttgttgcagttaaaaaagctcgtagttggatttcggagtgggcccgttggtccgccgcaaggtgtgttactgactggtctgctcttcttcgcaaagactgcgtgtgctctttgttgagtgtgcgtaggatttacgacgtttactttgaaaaaaattagagtgttcaaagcaggctattgcttgaatacatgagcatggaataatggaataggactttggtcccattttgttggtttctaggaccgaagtaatgattaagagggacaattgggggcatccgtatttcgttgtcagaggtgaaattcttggatttacgaaagacgaacaactgcgaaagcacttgccaagagtgttttca
>00515a05e5b05f33d5e3356cc83a7c5d\_15
agctccaatagcgtatattaaagttgttgcagttaaaaagctcgtagttggatttcggagtgggccagttggtccgccgcaaggtgtgttactgactggtttgctcttcttcgcaaagactgcgtgtgctctttattgagtgtgcgtaggatttacgacgtttactttgaaaaaattagagtgttcaaagcaggctattgcttgaatacatgagcatggaataatggaataggattttggtcccattttgttggtttctaggaccgaagtaatgattaagagggacaattgggggcatccgtatttcgttgtcagaggtgaattcttggatttacgaaagacgaacaactgcgaaagcacttgccaagagtgttttca
>0c50218d78989898fed11d2d8202e048\_15
agctccaatagcgtatattaaagttgttgcagttaaaaagctcgtagttggatttcggagtgggcccgttggtccgccgcaaggtgtgttactgactggtctgctcttcttcgcaaagactgcgtgtgctctttgttgagtgtgcgtaggatttacgacgtttactttgaaaaaattgagtgttcaaagcaggctattgcttgaatacatgagcatggaataatggaataggactttggtcccattttgttggtttctaggaccgaagtaatgattaagagggacaattgggggcatccgtatttcgttgtcagaggtgaaatttcttggatttacgaaagacgaacaactgcgaaagcacttgccaagagtgttttca
>4e1af44b3be15770273000de63ecd166\_15
agctccaatagcgtatattaaagttgttgcagttaaaaagctcgtagttggatttcggagtgggcccgttggtccgccgcaaggtgtgttactgactggtctgctcttcttcgcaaagactgcgtgtgctctttgttgagtgtgcgtaggatttacgacgtttactttgaaaaaattagagtgttcaaagcaggctattgcttgaatacatgagcatggaataatggaataggactttggtcccattttgttggtttctaggaccgaagtaatgattaagagggacaattgggggcatccgtatttcgttgtcagaggtgaaatttcttggatttacgaaaagacgaacaactgcgaaagcacttgccaagagtgttttca
>ec6240f0dcf80b134d7136b0c13131ad\_15
agctccaatagcgtatattaaagttgttgcagttaaaagctcgtagttggatttcggagtgggcccgttggtccgccgcaaggtgtgttactgactggtctgctcttcttcgcaaagactgcgtgtgctctttgttgagtgtgcgtaggatttacgacgtttactttgaaaaaattagagtgttcaaagcaggctattgcttgaatacatgagcatggaataatggaataggactttggtcccattttgttggtttctaggaccgaagtaatgattaagagggacaattgggggcatccgtatttcgttgtcagaggtgaaattcttggatttacgaaagacgaacaactgcgaaagcacttgccaagagtgttttca
>276f1cd872338bea058f85a8452d393f\_14
agctccaatagcgtatattaaagttgttgcggttaaaaagctcgtagttggatttcggagtgggcccgttggtccgccgcaaggtgtgttactgactggtctgctcttcttcgcaaagactgcgtgtgctctttgttgagtgtgcgtaggatttacgacgtttactttgaaaaaattagagtgttcaaagcaggctattgcttgaatacatgagcatggaataatggaataggactttggtcccattttgttggtttctaggaccgaagtaatgattaagagggacaattgggggcatccgtatttcgttgtcagaggtgaaattcttggatttacgaaagacgaacaactgcgaaagcacttgccaagagtgttttca
>51dbca29f9d71d6b02fd4124f99db412\_14
agctccaatagcgtatattaaagttgttgcagttaaaaagctcgtagttggatttcggagtgggccagttggtccgccgcaaggtgtgttactgactggtttgctcttcttcgcaaagactgcgtgtgctctttgttgagtgtgcgtaggatttacgacgtttactttgaaaaaaattagagtgttcaaagcaggctattgcttgaatacatgagcatggaataatggaataggactttggtcccattttgttggtttctaggaccgaagtaatgattaagagggacaattgggggcatccgtatttcgttgtcagaggtgaaattcttggatttacgaaagacgaacaactgcgaaagcacttgccaagagtgttttca
>71250a4b73bfa7612c11b6538d551032\_14
agctccaatagcgtatattaaagttgttgcagttaaaaaagctcgtagttggatttcggagtgggccagttggtccgccgcaaggtgtgttactgactggtttgctcttcttcgcaaagactgcgtgtgctctttattgagtgtgcgtaggatttacgacgtttactttgaaaaaattagagtgttcaaagcaggctattgcttgaatacatgagcatggaataatggaataggattttggtcccattttgttggtttctaggaccgaagtaatgattaagagggacaattggggcatccgtatttcgttgtcagaggtgaaattcttggatttacgaaagacgaacaactgcgaaagcacttgccaagagtgtttca
>f9235aeef63c7d2ebc7eb9ad1932d0de\_14
agctccaatagcgtatattaaagttgttgcagttaaaaagctcgtagttggatttcggagtgggccagttggtccgccgcaaggtgtgttactgactggtttgctcttcttcgcaaagactgcgtgtgctctttattgagtgtgcgtaggatttacgacgtttactttgaaaaaattagagtgttcaaagcaggctattgcttgaatacatgagcatggaataatggaataggattttggtcccattttgttggtttctaggaccgaagtaatgattaagagggacaattgggggcatccgtatttcgttgtcagaggtgaaattcttggatttacgaaagacgaacaactgcgaagcacttgccaagagtgttttca
>18b0b758eeb7d8360ae560140f3458f2\_13
agctccaatagcgtatattaaagttgttgcagttaaaaagctcgtagttggattttcggagtgggcccgttggtccgccgcaaggtgtgttactgactggtctgctcttcttcgcaaagactgcgtgtgctctttgttgagtgtgcgtaggatttacgacgtttactttgaaaaaattagagtgttcaaagcaggctattgcttgaatacatgagcatggaataatggaataggactttggtcccattttgttggtttctaggaccgaagtaatgattaagagggacaattgggggcatccgtatttcgttgtcagaggtgaaattcttggatttacgaaagacgaacaactgcgaaagcacttgccaagagtgttttca
>89f95a3d0e6af42762ebf12edd8894f9\_13
agctccaatagcgtatattaaagttgttgcagttaaaaagctcgtagttggatttcggagtgggcccgttggtccgccgcaaggtgtgttactgactggtctgctcttcttcgcaaagactgcgtgtgctctttgttgagtgtgcgtaggatttacgacgtttactttgaaaaaaattagagtgttcaaagcaggctattgcttgaatacatgagcatggaataatggaataggactttggtcccattttgttggtttctaggaccgaagtaatgattaagagggacaattgggggcatccgtatttcgttgtcagaggtgaaattctttggatttacgaaagacgaacaactgcgaaagcacttgccaagagtgttttca
>a9ed5fc350760133c4f12bee018414b2\_13
agctccaatagcgtatattaaagttgttgcggttaaaaagctcgtagttggatttcggagtgggccagttggtccgccgcaaggtgtgttactgactggtctgctcttcttcgcaaagactgcgtgtgctctttgttgagtgtgcgtaggatttacgacgtttactttgaaaaaattagagtgttcaaagcaggctattgcttgaatacatgagcatggaataatggaataggactttggtcccattttgttggtttctaggaccgaagtaatgattaagagggacaattgggggcatccgtatttcgttgtcagaggtgaaattcttggatttacgaaagacgaacaactgcgaaagcatttgccaagagtgttttca
>fcbe43d71573c8b87ad3b2b4031d3b07\_13
agctccaatagcgtatattaaagttgttgcagttaaaaagctcgtagttggatttcggagtgggccagttggtccgccgcaaggtgtgttactgactggtctgctcttcttcgcaaagactgcgtgtgctctttgttgagtgtgcgtaggatttacgacgtttactttgaaaaaattagagtgttcaaagcaggctattgcttgaatacatgagcatggaataatggaataggactttggtcccattttgttggtttctaggaccgaagtaatgattaagagggacaattgggggcatccgtatttcgttgtcagaggtgaaattcttggatttacgaaagacgaacaactgcgaaagcatttgccaagagtgtttttca
>53a9117f7bf7d159ecce86aa81ba3d13\_12
agctccaatagcgtatattaaagttgttgcagttaaaaagctcgtagttggatttcggagtgggccagttggtccgccgcaaggtgtgttactgactggtttgctcttcttcgcaaagactgcgtgtgctctttattgagtgtgcgtaggatttacgacgtttactttgaaaaaaattgagtgttcaaagcaggctattgcttgaatacatgagcatggaataatggaataggattttggtcccattttgttggtttctaggaccgaagtaatgattaagagggacaattgggggcatccgtatttcgttgtcagaggtgaaattcttggatttacgaaagacgaacaactgcgaaagcacttgccaagagtgttttca
>ec4c22b2bd35e69c9218559c594bdcf9\_12
agctccaatagcgtatattaaagttgttgcagttaaaaagctcgtagttggatttcggagtgggcccgttggtccgccgcaaggtgtgttactgactggtctgctcttcttcgcaaagactgcgtgtgctctttgttgagtgtgcgtaggatttacgacgtttactttgaaaaaattagagtgttcaaagcaggctattgcttgaatacatgagcatggaataatggaataggactttggtcccattttgttggtttctaggaccgaagtaatgattaagagggacaattgggggcatccgtattcgttgtcagaggtgaaattcttggatttacgaaagacgaacaactgcgaaagcacttgccaagagtgttttca
>297a26ad91753b49f84fcfa9f8f6f93a\_11
agctccaatagcgtatattaaagttgttgcagttaaaaagctcgtagttggatttcggagtgggccagttggtccgccgcaaggtgtgttactgactggtttgctcttcttcgcaaagactgcgtgtgctctttattgagtgtgcgtaggatttacgacgtttactttgaaaaaattagagtgttcaaagcaggctattgcttgaatacatgagcatggaataatggaataggattttggtcccattttgttggtttctaggaccgaagtaatgattaagagggacaattgggggcatccgtatttcgtgtcagaggtgaaattcttggatttacgaaagacgaacaactgcgaaagcacttgccaagagtgttttca
>a0b24b8b366850889c0fa08241998a92\_11
agctccaatagcgtatattaaagttgttgcagttaaaaagctcgtagttggatttcggagtgggccagttggtccgccgcaaggtgtgttactgactggtttgctcttcttcgcaaagactgcgtgtgctctttattgagtgtgcgtaggatttacgacgtttactttgaaaaaattgagtgttcaaagcaggctattgcttgaatacatgagcatggaataatggaataggattttggtcccattttgttggtttctaggaccgaagtaatgattaagagggacaattgggggcatccgtatttcgttgtcagaggtgaaattcttggatttacgaaagacgaacaactgcgaaagcacttgccaagagtgtttca
>a195bb9df8bf3ba444796edaec889df3\_11
agctccaatagcgtatattaaagttgttgcagttaaaaagctcgtagttggatttcggagtgggccagttggtccgccgcaaggtgtgttactgactggtttgctcttcttcgcaaagactgcgtgtgctctttattgagtgtgcgtaggatttacgacgtttactttgaaaaaattagagtgttcaaagcaggctattgcttgaatacatgagcatggaataatggaataggattttggtcccattttgttggtttctaggaccgaagtaatgattaagagggacaattgggggcatccgtatttcgttgtcagaggtgaaattcttggattttacgaaagacgaacaactgcgaaagcacttgccaagagtgttttca
>c5a3569a6fc3ee0d712c15ca2086a9ca\_11
agctccaatagcgtatattaaagttgttgcagttaaaaaagctcgtagttggatttcggagtgggcccgttggtccgccgcaaggtgtgttactgactggtctgctcttcttcgcaaagactgcgtgtgctctttgttgagtgtgcgtaggatttacgacgtttactttgaaaaaattagagtgttcaaagcaggctattgcttgaatacatgagcatggaataatggaataggactttggtcccattttgttggtttctaggaccgaagtaatgattaagagggacaattggggcatccgtatttcgttgtcagaggtgaaattcttggatttacgaaagacgaacaactgcgaaagcacttgccaagagtgttttca
>dd4af1791ab65ce23a8cf85a2adb24b7\_11
agctccaatagcgtatattaaagttgttgcagttaaaaagctcgtagttggatttcggagtgggcccgttggtccgccgcaaggtgtgttactgactgggtctgctcttcttcgcaaagactgcgtgtgctctttgttgagtgtgcgtaggatttacgacgtttactttgaaaaaattagagtgttcaaagcaggctattgcttgaatacatgagcatggaataatggaataggactttggtcccattttgttggtttctaggaccgaagtaatgattaagagggacaattgggggcatccgtatttcgttgtcagaggtgaaattcttggatttacgaaagacgaacaactgcgaaagcacttgccaagagtgttttca
>5f504b4ca0a98f7ca9f0ce4d4eabf34b\_10
agctccaatagcgtatattaaagttgttgcagttaaaaagctcgtagttggatttcggagtgggcccgttggtccgccgcaaggtgtgttactgactggtctgctcttcttcgcaaagactgcgtgtgctctttgttgagtgtgcgtaggatttacgacgtttactttgaaaaaattagagtgttcaaagcaggctattgcttgaatacatgagcatggaataatggaataggactttggtcccatttttgttggtttctaggaccgaagtaatgattaagagggacaattgggggcatccgtatttcgttgtcagaggtgaaattcttggatttacgaaagacgaacaactgcgaaagcacttgccaagagtgttttca
>aad669bb2c622fbbbec15c81c182cea8\_10
agctccaatagcgtatattaaagttgttgcagttaaaaaagctcgtagttggatttcggagtgggccagttggtccgccgcaaggtgtgttactgactggtttgctcttcttcgcaaagactgcgtgtgctctttattgagtgtgcgtaggatttacgacgtttactttgaaaaaattagagtgttcaaagcaggctattgcttgaatacatgagcatggaataatggaataggatttggtcccattttgttggtttctaggaccgaagtaatgattaagagggacaattgggggcatccgtatttcgttgtcagaggtgaaattcttggatttacgaaagacgaacaactgcgaaagcacttgccaagagtgttttca
>e0d0137977d7c5c1de77cb399701cd27\_10
agctccaatagcgtatattaaagttgttgcagttaaaagctcgtagttggatttcggagtgggccagttggtccgccgcaaggtgtgttactgactggtctgctcttcttcgcaaagactgcgtgtgctctttgttgagtgtgcgtaggatttacgacgtttactttgaaaaaattagagtgttcaaagcaggctattgcttgaatacatgagcatggaataatggaataggactttggtcccattttgttggtttctaggaccgaagtaatgattaagagggacaattgggggcatccgtatttcgttgtcagaggtgaaattcttggatttacgaaagacgaacaactgcgaaagcatttgccaagagtgttttca
>23ac1673feb707cabcd5f61c5f7ba052\_9
agctccaatagcgtatattaaagttgttgcagttaaaaagctcgtagttggatttcggagtgggccagttggtccgccgcaaggtgtgttactgactggtttgctcttcttcgcaaagactgcgtgtgctctttattgagtgtgcgtaggatttacgacgtttactttgaaaaaattagagtgttcaaagcaggctattgcttgaatacatgagcatggaataatggaataggatttggtcccattttgttggtttctaggaccgaagtaatgattaagagggacaattgggggcatccgtatttcgttgtcagaggtgaaattcttggatttacgaaagacgaacaactgcgaaagcacttgccaagagtgtttca
>3c5186ea9ec5c168348a0718ff0ffe1f\_9
agctccaatagcgtatattaaagttgttgcagttaaaaaagctcgtagttggatttcggagtgggccagttggtccgccgcaaggtgtgttactgactggtctgctcttcttcgcaaagactgcgtgtgctctttgttgagtgtgcgtaggatttacgacgtttactttgaaaaaattagagtgttcaaagcaggctattgcttgaatacatgagcatggaataatggaataggactttggtcccattttgttggtttctaggaccgaagtaatgattaagagggacaattgggggcatccgtatttcgttgtcagaggtgaaattcttggatttacgaaagacgaacaactgcgaaagcatttgccaagagtgtttca
>93e3268f9fed0e45931a6d47de7ed321\_9
agctccaatagcgtatattaaagttgttgcagttaaaaagctcgtagttggatttcggagtgggccagttggtccgccgcaaggtgtgttactgactggtctgctcttcttcgcaaagactgcgtgtgctctttgttgagtgtgcgtaggatttacgacgtttactttgaaaaaaattagagtgttcaaagcaggctattgcttgaatacatgagcatggaataatggaataggactttggtcccattttgttggtttctaggaccgaagtaatgattaagagggacaattgggggcatccgtatttcgttgtcagaggtgaaattcttggatttacgaaagacgaacaactgcgaaagcacttgccaagagtgttttca
>ba28aaf6b82d2788352fd9891938f536\_9
agctccaatagcgtatattaaagttgttgcagttaaaaagctcgtagttggatttcggagtgggcccgttggtccgccgcaaggtgtgttactgactggtctgctcttcttcgcaaagactgcgtgtgctctttgttgagtgtgcgtaggatttacgacgtttactttgaaaaaattagagtgttcaaagcaggctattgcttgaatacatgagcatggaataatggaataggactttggtcccattttgttggtttctaggaccgaagtaatgattaagagggacaattgggggcatccgtatttcgttgtcagaggtgaaattcttggatttacgaaaagacgaacaactgcgaaagcacttgccaagagtgttttca
>be787512e9c3e4b2099ff9da5d400b7e\_9
agctccaatagcgtatattaaagttgttgcagttaaaaagctcgtagttggatttcggagtgggcccgttggtccgccgcaaggtgtgttactgactggtctgctcttcttcgcaaagactgcgtgtgctctttgttgagtgtgcgtaggatttacgacgtttactttgaaaaaattagagtgttcaaagcaggctattgcttgaatacatgagcatggaataatggaataggactttggtcccattttgttggtttctaggaccgaagtaatgattaagagggacaattgggggcatccgtatttcgttgtcagaggtgaaatttcttggatttacgaaagacgaacaactgcgaatgcacttgccaagagtgttttca
>0360f9a7c3e6c1203c3724fc1cfc196f\_8
agctccaatagcgtatattaaagttgttgcagttaaaaagctcgtagttggatttcggagtgggccagttggtccgccgcaaggtgtgttactgactggtttgctcttcttcgcaaagactgcgtgtgctctttattgagtgtgcgtaggatttacgacgtttactttgaaaaaattagagtgttcaaagcaggctattgcttgaatacatgagcatggaataatggaataggatttggtcccattttgttggtttctaggaccgaagtaatgattaagagggacaattggggcatccgtatttcgttgtcagaggtgaaattcttggatttacgaaagacgaacaactgcgaaagcacttgccaagagtgttttca
>3ada1a4933c6c1ba2c79135b5bf4ba43\_8
agctccaatagcgtatattaaagttgttgcagttaaaaagctcgtagttggatttcggagtgggccagttggtccgccgcaaggtgtgttactgactggtctgctcttcttcgcaaagactgcgtgtgctctttgttgagtgtgcgtaggatttacgacgtttactttgaaaaaattgagtgttcaaagcaggctattgcttgaatacatgagcatggaataatggaataggactttggtcccattttgttggtttctaggaccgaagtaatgattaagagggacaattggggcatccgtatttcgttgtcagaggtgaaattcttggatttacgaaagacgaacaactgcgaaagcatttgccaagagtgttttca
>423ca8ed4c8646ba274bbe57ce0353a9\_8
agctccaatagcgtatattaaagttgttgcagttaaaaagctcgtagttggatttcggagtgggccagttggtccgccgcaaggtgtgttactgactggtttgctcttcttcgcaaagactgcgtgtgctctttattgagtgtgcgtaggatttacgacgtttactttgaaaaaattagagtgttcaaagcaggctattgcttgaatacatgagcatggaataatggataggattttggtcccattttgttggtttctaggaccgaagtaatgattaagagggacaattgggggcatccgtatttcgttgtcagaggtgaaattcttggatttacgaaagacgaacaactgcgaaagcacttgccaagagtgttttca
>425b5706668e4e43b55a41d145a5df57\_8
agctccaatagcgtatattaaagttgttgcagttaaaaagctcgtagttggatttcggagtgggccagttggtccgccgcaaggtgtgttactgactggtttgctcttcttcgcaaagactgcgtgtgctctttattgagtgtgcgtaggatttacgacgtttactttgaaaaaattagagtgttcaaagcaggctattgcttgaatacatgagcatggaataatggaataggattttggtcccattttgttggtttctaggaccgaagtaatgattaagagggacaattgggggcatccgtatttcgttgtcagaggtgaaattcttggatttacgaaagacgaacaactgcgaaagcaacttgccaagagtgttttca
>43494c584c4067954eb2968cc2fd7dd3\_8
agctccaatagcgtatattaaagttgttgcagttaaaaagctcgtagttggatttcggagtgggccagttggtccgccgcaaggtgtgttactgactggtctgctcttcttcgcaaagactgcgtgtgctctttgttgagtgtgcgtaggatttacgacgtttactttgaaaaaattagagtgttcaaagcaggctattgcttgaatacatgagcatggaataatggaataggactttggtcccatttttgttggtttctaggaccgaagtaatgattaagagggacaattgggggcatccgtatttcgttgtcagaggtgaaattcttggatttacgaaagacgaacaactgcgaaagcatttgccaagagtgttttca
>58000d532425329d7bc84c006ef38457\_8
agctccaatagcgtatattaaagttgttgcagttaaaaagactcgtagttggatttcggagtgggcccgttggtccgccgcaaggtgtgttactgactggtctgctcttcttcgcaaagactgcgtgtgctctttgttgagtgtgcgtaggatttacgacgtttactttgaaaaaattagagtgttcaaagcaggctattgcttgaatacatgagcatggaataatggaataggactttggtcccattttgttggtttctaggaccgaagtaatgattaagagggacaattgggggcatccgtatttcgttgtcagaggtgaaattcttggatttacgaaagacgaacaactgcgaaagcacttgccaagagtgttttca
>76ea528d5fdb23f1718202f8e3da7593\_8
agctccaatagcgtatattaaagttgttgcagttaaaaagctcgtagttggatttcggagtgggccagttggtccgccgcaaggtgtgttactgactggtttgctcttcttcgcaaagactgcgtgtgctctttattgagtgtgcgtaggatttacgacgtttactttgaaaaaattagagtgttcaaagcaggctattgcttgaatacatgagcatggaataatggaataggatttttggtcccatttttgttggtttctaggaccgaagtaatgattaagagggacaattgggggcatccgtatttcgttgtcagaggtgaaattcttggatttacgaaagacgaacaactgcgaaagcacttgccaagagtgttttca
>7cd5f05271f397056b097a238604a62f\_8
agctccaatagcgtatattaaagttgttgcagttaaaaagctcgtagttggatttcggagtgggccagttggtccgccgcaaggtgtgttactgactggtctgctcttcttcgcaaagactgcgtgtgctctttgttgagtgtgcgtaggatttacgacgtttactttgaaaaaattagagtgttcaaagcaggctattgcttgaatacatgagcatggaataatggaataggactttggtcccattttgttggtttctaggaccgaagtaatgattaagagggacaattggggcatccgtatttcgttgtcagaggtgaaattcttggatttacgaaagacgaacaactgcgaaagcatttgccaagagtgtttca
>83952410a3f6b916e7a1e6c9b0205952\_8
agctccaatagcgtatattaaagttgttgcagttaaaagctcgtagttggatttcggagtgggccagttggtccgccgcaaggtgtgttactgactggtttgctcttcttcgcaaagactgcgtgtgctctttattgagtgtgcgtaggatttacgacgtttactttgaaaaaattagagtgttcaaagcaggctattgcttgaatacatgagcatggaataatggaataggattttggtcccattttgttggtttctaggaccgaagtaatgattaagagggacaattgggggcatccgtatttcgttgtcagaggtgaaattcttggatttacgaaagacgaacaactgcgaaagcacttgccaagagtgttttca
>ab7d67ed75002104f6437251556525ff\_8
agctccaatagcgtatattaaagttgttgcagttaaaaaagctcgtagttggatttcggagtgggccagttggtccgccgcaaggtgtgttactgactggtctgctcttcttcgcaaagactgcgtgtgctctttgttgagtgtgcgtaggatttacgacgtttactttgaaaaaattagagtgttcaaagcaggctattgcttgaatacatgagcatggaataatggaataggactttggtcccattttgttggtttctaggaccgaagtaatgattaagagggacaattggggcatccgtatttcgttgtcagaggtgaaattcttggatttacgaaagacgaacaactgcgaaagcatttgccaagagtgttttca
>af420072f2ff83c99a02c3f09541217d\_8
agctccaatagcgtatattaaagttgttgcagttaaaaagctcgtagttggatttcggagtgggcccgttggtccgccgcaaggtgtgttactgactggtctgctcttcttcgcaaagactgcgtgtgctctttgttgagtgtgcgtaggatttacgacgtttactttgaaaaattagagtgttcaaagcaggctattgcttgaatacatgagcatggaataatggaataggactttggtcccattttgttggtttctaggaccgaagtaatgattaagagggacaattgggggcatccgtatttcgttgtcagaggtgaaattcttggatttacgaaagacgaacaactgcgaaagcacttgccaagagtgttttca
>db78b9f78721da9c17ad3633db34086c\_8
agctccaatagcgtatattaaagttgttgcagttaaaaagctcgtagttggatttcggagtgggcccgttggtccgccgcaaggtgtgttactgactggtctgctcttcttcgcaaagactgcgtgtgctctttgttgagtgtgcgtaggattttacgacgtttactttgaaaaaattagagtgttcaaagcaggctattgcttgaatacatgagcatggaataatggaataggactttggtcccattttgttggtttctaggaccgaagtaatgattaagagggacaattgggggcatccgtatttcgttgtcagaggtgaaattcttggatttacgaaagacgaacaactgcgaaagcacttgccaagagtgttttca
>ddabf02e03700431b39ce836aeead44c\_8
agctccaatagcgtatactaaagttgttgcagttaaaaagctcgtagttggatttcggagtgggcccgttggtccgccgcaaggtgtgttactgactggtctgctcttcttcgcaaagactgcgtgtgctctttgttgagtgtgcgtaggatttacgacgtttactttgaaaaaattagagtgttcaaagcaggctattgcttgaatacatgagcatggaataatggaataggactttggtcccattttgttggtttctaggaccgaagtaatgattaagagggacaattgggggcatccgtatttcgttgtcagaggtgaaattcttggatttacgaaagacgaacaactgcgaaagcacttgccaagagtgttttca
>f79dd9a9cd44033cb0ffdc8491e1e9f9\_8
agctccaatagcgtatattaaagttgttgcggttaaaaagctcgtagttggatttcggagtgggcccgttggtccgccgcaaggtgtgttactgactggtctgctcttcttcgcaaagactgcgtgtgctctttgttgagtgtgcgtaggatttacgacgtttactttgaaaaaattagagtgttcaaagcaggctattgcttgaatacatgagcatggaataatggaataggactttggtcccattttgttggtttctaggaccgaagtaatgattaagagggacaattgggggcatccgtatttcgttgtcagaggtgaaatttcttggatttacgaaagacgaacaactgcgaaagcacttgccaagagtgttttca
>f81f0b15c83e4d4e0c8512878ab4cdc1\_8
agctccaatagcgtatattaaagttgttgcagttaaaaagctcgtagttggatttcggagtgggccagttggtccgccgcaaggtgtgttactgactggtttgctcttcttcgcaaagactgcgtgtgctctttattgagtgtgcgtaggatttacgacgtttactttgaaaaaattagagtgttcaaagcaggctattgcttgaatacatgagcatggaataatggaataggattttggtcccattttgttggtttctaggaccgaagtaatgattaagagggacaattgggggcatccgtatttcgttgtcagaggtgaaattcttggatttacgaaagacgaacaactgcgaaagcacttgccaagagtagttttca
>f957bd8a40785b57896e3852e923dfb4\_8
agctccaatagcgtatattaaagttgttgcagttaaaaagctcgtagttggatttcggagtgggccagttggtccgccgcaaggtgtgttactgactggtttgctcttcttcgcaaagactgcgtgtgctctttattgagtgtgcgtaggatttacgacgtttactttgaaaaaattagagtgttcaaagcaggctattgcttgaatacatgagcatggaataatggaataggattttggtcccattttgttggtttctaggaccgaagtaatgattaagagggacaattgggggcatccgtatttcgttgtcagaggtgaaatttcttggatttacgaaagacgaacaactgcgaaagcacttgccaagagtgttttca
>0ed61fdc2b85d8787b4501dc3ead3b13\_7
agctccaatagcgtatattaaagttgttgcagttaaaaaagctcgtagttggatttcggagtgggccagttggtccgccgcaaggtgtgttactgactggtctgctcttcttcgcaaagactgcgtgtgctctttgttgagtgtgcgtaggatttacgacgtttactttgaaaaaattagagtgttcaaagcaggctattgcttgaatacatgagcatggaataatggaataggactttggtcccattttgttggtttctaggaccgaagtaatgattaagagggacaattgggggcatccgtatttcgttgtcagaggtgaaattcttggatttacgaaagacgaacaactgcgaaagcacttgccaagagtgttttca
>1032399087542344b202ab6bc6b9b2f8\_7
agctccaatagcgtatattaaagttgttgcagttaaaaagctcgtagttggatttcggagtgggccccgttggtccgccgcaaggtgtgttactgactggtctgctcttcttcgcaaagactgcgtgtgctctttgttgagtgtgcgtaggatttacgacgtttactttgaaaaaattagagtgttcaaagcaggctattgcttgaatacatgagcatggaataatggaataggactttggtcccattttgttggtttctaggaccgaagtaatgattaagagggacaattgggggcatccgtatttcgttgtcagaggtgaaattcttggatttacgaaagacgaacaactgcgaaagcacttgccaagagtgttttca
>324080bcd342516fdadc60707f6068c8\_7
agctccaatagcgtatattaaagttgttgcagttaaaaagctcgtagttggatttcggagtgggccagttggtccgccgcaaggtgtgttactgactggtttgctcttcttcgcaaagactgcgtgtgctctttattgagtgtgcgtaggatttacgacgtttactttgaaaaaattagagtgttcaaagcaggctattgcttgaatacatgagcatggaataatggaataggattttggtcccattttgttggtttctaggaccgaagtaatgattaagagggacaattgggggcatccgtattcgttgtcagaggtgaaattcttggatttacgaaagacgaacaactgcgaaagcacttgccaagagtgttttca
>54a2d77d6b75501dab4fbdb0d6be0e7e\_7
agctccaatagcgtatattaaagttgttgcagttaaaaagctcgtagttggatttcggagtgggcccgttggtccgccgcaaggtgtgttactgactggtctgctcttcttcgcaaagactgcgtgtgctctttgttgagtgtgcgtaggatttacgacgtttactttgaaaaaattagagtgttcaaagcaggctattgcttgaatacatgagcatggaataatggaataggactttggtcccattttgttggtttctaggaccgaagtaatgattaagagggacaattggggggcatccgtatttcgttgtcagaggtgaaattcttggatttacgaaagacgaacaactgcgaaagcacttgccaagagtgttttca
>57b451d8caaeb21e668c3afb6a239688\_7
agctccaatagcgtatattaaagttgttgcagttaaaaagctcgtagttggatttcggagtgggcccgttggtccgccgcaaggtgtgttactgactggtctgctcttcttcgcaaagactgcgtgtgctctttgttgagtgtgcgtaggatttacgacgttttactttgaaaaaattagagtgttcaaagcaggctattgcttgaatacatgagcatggaataatggaataggactttggtcccattttgttggtttctaggaccgaagtaatgattaagagggacaattgggggcatccgtatttcgttgtcagaggtgaaattcttggatttacgaaagacgaacaactgcgaaagcacttgccaagagtgttttca
>6bb930024f40b872ffde75c770c35951\_7
agctccaatagcgtatattaaagttgttgcagttaaaaagctcgtagttggatttcggagtgggcccgttggtccgccgcaaggtgtgttactgactggtctgctcttcttcgcaaagactgcgtgtgctctttgttgagtgtgcgtaggatttacgacgtttactttgaaaaaaattagagtgttcaaagcaggctattgcttgaatacatgagcatggaataatggaataggactttggtcccattttgttggtttctaggaccgaagtaatgattaagagggacaattgggggcatccgtatttcgttgtcagaggtgaaatttcttggatttacgaaagacgaacaactgcgaaagcacttgccaagagtgttttca
>6f4df362be7a41e5ee7c252e24b37b4c\_7
agctccaatagcgtatattaaagttgttgcagttaaaaagctcgtagttggatttcggagtgggtcagttggtccgccgcaaggtgtgttactgactggtttgctcttcttcgcaaagactgcgtgtgctctttattgagtgtgcgtaggatttacgacgtttactttgaaaaaattagagtgttcaaagcaggctattgcttgaatacatgagcatggaataatggaataggattttggtcccattttgttggtttctaggaccgaagtaatgattaagagggacaattgggggcatccgtatttcgttgtcagaggtgaaattcttggatttacgaaagacgaacaactgcgaaagcacttgccaagagtgttttca
>885395b7879ab4572e9fd5fc69256cf1\_7
agctccaatagcgtatattaaagttgttgcagttaaaaagactcgtagttggatttcggagtgggccagttggtccgccgcaaggtgtgttactgactggtttgctcttcttcgcaaagactgcgtgtgctctttattgagtgtgcgtaggatttacgacgtttactttgaaaaaattagagtgttcaaagcaggctattgcttgaatacatgagcatggaataatggaataggattttggtcccattttgttggtttctaggaccgaagtaatgattaagagggacaattgggggcatccgtatttcgttgtcagaggtgaaattcttggatttacgaaagacgaacaactgcgaaagcacttgccaagagtgttttca
>923779d23b1a97234385f08160b0794b\_7
agctccaatagcgtatattaaagttgttgcagttaaaaagctcgtagttggatttcggagtgggcccgttggtccgccgcaaggtgtgttactgactggtctgctcttcttcgcaaagactgcgtgtgctctttgttgagtgtgcgtaggatttacgacgtttactttgaaaaaattagagtgttcaaagcaggctattgcttgaatacatgagcatggaataatggaataggactttggtcccattttgttggtttctaggaccgaagtaatgattaagagggacaattgggggcatccgtatttcgttgtcagaggtgaaattcttggatttacgaaagacgaacaactgcgaaagcacttgccaagaatgttttca
>a0dec25eef263e3313f43a057f249c52\_7
agctccaatagcgtatattaaagttgttgcagttaaaaagctcgtagttggatttcggagtgggcccgttggtccgccgcaaggtgtgttactgactggtctgctcttcttcgcaaaagactgcgtgtgctctttgttgagtgtgcgtaggatttacgacgtttactttgaaaaaattagagtgttcaaagcaggctattgcttgaatacatgagcatggaataatggaataggactttggtcccattttgttggtttctaggaccgaagtaatgattaagagggacaattgggggcatccgtatttcgttgtcagaggtgaaattcttggatttacgaaagacgaacaactgcgaaagcacttgccaagagtgttttca
>b8d93a2691acc55aed83637d59430658\_7
agctccaatagcgtatattaaagttgttgcagttaaaaagctcgtagttggatttcggagtgggccagttggtccgccgcaaggtgtgttactgactggtctgctcttcttcgcaaagactgcgtgtgctctttgttgagtgtgcgtaggatttacgacgtttactttgaaaaaattagagtgttcaaagcaggctattgcttgaatacatgagcatggaataatggaataggactttggtcccattttgttggtttctaggaccgaagtaatgattaagagggacaagttgggggcatccgtatttcgttgtcagaggtgaaattcttggatttacgaaagacgaacaactgcgaaaagcatttgccaagagtgttttca
>e8897904cca30663aea40fde4450145c\_7
agctccaatagcgtatattaaagttgttgcagttaaaaagctcgtagttggatttcggagtgggcccgttggtccgccgcaaggtgtgttactgactggtctgctcttcttcgcaaagactgcgtgtgctctttgttgagtgtgcgtaggatttacgacgtttactttgaaaaaattagagtgttcaaagcaggctattgcttgaatacatgagcatggaataatggaataggactttggtcccattttgttggtttctaggaccgaagtaatgattaagagggacaattgggggcatccgtatttcgttgtcagaggtgaaattcttggatttacgaaagacgaacaactgcgaaaagcacttgccaagagtgtttttca
>01fd5675c0fb200a61a70ab7272cc9e8\_6
agctccaatagcgtatattaaagttgttgcagttaaaaagctcgtagttggatttcggagtgggccagttggtccgccgcaaggtgtgttactgactggtctgctcttcttcgcaaagactgcgtgtgctctttgttgagtgtgcgtaggatttacgacgtttactttgaaaaaaattagagtgttcaaagcaggctattgcttgaatacatgagcatggaataatggaataggactttggtcccattttgttggtttctaggaccgaagtaatgattaagagggacaattgggggcatccgtatttcgttgtcagaggtgaaattcttggatttacgaaagacgaacaactgcgaaagcatttgccaagagtgtttca
>0deaebf62f3033c2be2b23fcedd2d611\_6
agctccaatagcgtatattaaagttgttgcggttaaaaagctcgtagttggatttcggagtgggccagttggtccgccgcaaggtgtgttactgactggtctgctcttcttcgcaaagactgcgtgtgctctttgttgagtgtgcgtaggatttacgacgtttactttgaaaaaaattagagtgttcaaagcaggctattgcttgaatacatgagcatggaataatggaataggactttggtcccattttgttggtttctaggaccgaagtaatgattaagagggacaattgggggcatccgtatttcgttgtcagaggtgaaattcttggatttacgaaagacgaacaactgcgaaagcatttgccaagagtgttttca
>20f33602b048d8109f6b4f470f4d5ff3\_6
agctccaatagcgtattattaaagttgttgcagttaaaaagctcgtagttggatttcggagtgggcccgttggtccgccgcaaggtgtgttactgactggtctgctcttcttcgcaaagactgcgtgtgctctttgttgagtgtgcgtaggatttacgacgtttactttgaaaaaattagagtgttcaaagcaggctattgcttgaatacatgagcatggaataatggaataggactttggtcccattttgttggtttctaggaccgaagtaatgattaagagggacaattgggggcatccgtatttcgttgtcagaggtgaaattcttggatttacgaaagacgaacaactgcgaaagcacttgccaagagtgttttca
>213a0b6e14531fe1e30be713e1922a1d\_6
agctccaatagcgtatattaaagttgttgcagttaaaaagctcgtagttggatttcggagtgggccagttggtccgccgcaaggtgtgttactgactggtttgctcttcttcgcaaagactgcgtgtgctctttattgagtgtgcgtaggatttacgacgtttactttgaaaaattagagtgttcaaagcaggctattgcttgaatacatgagcatggaataatggaataggattttggtcccattttgttggtttctaggaccgaagtaatgattaagagggacaattgggggcatccgtatttcgttgtcagaggtgaaattcttggatttacgaaagacgaacaactgcgaaagcacttgccaagagtgttttca
>2819ef22db72af20d45b519e8dcee870\_6
agctccaatagcgtatattaaagttgttgcagttaaaaagctcgtagttggatttcggagtgggcccgttggtccgccgcaaggtgtgttactgactggtctgctcttcttcgcaaagactgcgtgtgctctttgttgagtgtgcgtaggatttacgacgtttactttgaaaaaattagagtgttcaaagcaggctattgcttgaatacatgagcatggaataatggaataggactttggtcccattttgttggtttctaggaccgaagtaatgattaagagggacaattgggggcatccgtatttcgttgtcagaggtgaaattcttggatttacgaaagacgaaacaactgcgaaagcacttgccaagagtgttttca
>28b284b2cadd30b7cc19ac1b03b5cc29\_6
agctccaatagcgtatattaaagttgttgcagttaaaaagctcgtagttggatttcggagtgggccagttggtccgccgcaaggtgtgttactgactggtttgctcttcttcgcaaagactgcgtgtgctctttattgagtgtgcgtaggatttacgacgttttactttgaaaaaattagagtgttcaaagcaggctattgcttgaatacatgagcatggaataatggaataggattttggtcccattttgttggtttctaggaccgaagtaatgattaagagggacaattgggggcatccgtatttcgttgtcagaggtgaaattcttggatttacgaaagacgaacaactgcgaaagcacttgccaagagtgttttca
>29992e785119d7fa0dae2b0d39b6458a\_6
agctccaatagcgtatattaaagttgttgcagttaaaaagctcgtagttggatttcggagtgggcccgttggtccgccgcaaggtgtgttactgactggtctgctcttcttcgcaaagactgcgtgtgctctttgttgagtgtgcgtaggatttacgacgtttactttgaaaaaattagagtgttcaaagcaggctattgcttgaatacatgagcatggaataatggaataggactttggtcccattttgttggtttctaggaccgaagtaatgattaagagggacaattgggggcatccgtatttcgttgtcagaggtgaaatttcttggatttacgaaagacgaacaactgcgaaagcatttgccaagagtgttttca
>2c0a35e775612169155daa1684aea1ca\_6
agctccaatagcgtatattaaagttgttgcagttaaaaagctcgtagttggatttcggagtgggccagttggtccgccgcaaggtgtgttactgactggtttgctcttcttcgcaaagactgcgtgtgctctttattgagtgtgcgtaggatttacgacgtttactttgaaaaaattagagtgttcaaagcaggctattgcttgaatacatgagcatggaataatggaataggattttggtcccattttgttggtttctaggaccgaagtaatgattaagagggacaattgggggcatccgtatttcgttgtcagaggtgaaattcttggatttacgaaagacgaacaactgcgaaagcacttagccaagagtgttttca
>31c28270e663b4b1fe3efa9ed5e55456\_6
agctccaatagcgtatattaaagttgttgcagttaaaaagctcgtagttggatttcggagtgggcccgttggtccgccgcaaggtgtgttactgactggtctgctcttcttcgcaaagactgcgtgtgctctttgttgagtgtgcgtaggatttacgacgtttactttgaaaaaattagagtgttcaaagcaggctattgcttgaatacatgagcatggaataatggaataggactttggtcccattttgttggtttctaggaccgaagtaatgattaagagggacaattgggggcatccgtatttcgttgtcagagggtgaaattctttggatttacgaaagacgaacaactgcgaaagcacttgccaagagtgttttca
>335dfb88c987b660034a37836006780e\_6
agctccaatagcgtatattaaagttgttgcagttaaaaagctcgtagttggatttcggagtgggccagttggtccgccgcaaggtgtgttactgactggtctgctcttcttcgcaaagactgcgtgtgctctttgttgagtgtgcgtaggatttacgacgtttactttgaaaaaattagagtgttcaaagcaggctattgcttgaatacatgagcatggaataatggaataggactttggtcccattttgttggtttctaggaccgaagtaatgattaagagggacaattgggggcatccgtatttcgttgtcagaggtgaaattcttggatttacgaaaagacgaacaactgcgaaagcatttgccaagagtgttttca
>5dfe0cf2a18560148006f0aa0a1e5a71\_6
agctccaatagcgtatattaaagttgttgcagttaaaaagctcgtagttggattttcggagtgggccagttggtccgccgcaaggtgtgttactgactggtctgctcttcttcgcaaagactgcgtgtgctctttgttgagtgtgcgtaggatttacgacgtttactttgaaaaaattagagtgttcaaagcaggctattgcttgaatacatgagcatggaataatggaataggactttggtcccattttgttggtttctaggaccgaagtaatgattaagagggacaattgggggcatccgtatttcgttgtcagaggtgaaattcttggatttacgaaagacgaacaactgcgaaaagcatttgccaagagtgttttca
>6d52ea8663b7cd53d4d9f68932f8fde1\_6
agctccaatagcgtatattaaagttgttgcagttaaaaagctcgtagttggatttcggagtgggccagttggtccgccgcaaggtgtgttactgactggtctgctcttcttcgcaaagactgcgtgtgctctttgttgagtgtgcgtaggatttacgacgtttactttgaaaaaattagagtgttcaaagcaggctattgcttgaatacatgagcatggaataatggaataggactttggtcccattttgttggtttctagggccgaagtaatgattaagagggacaattgggggcatccgtatttcgttgtcagaggtgaaattcttggatttacgaaagacgaacaactgcgaaagcacttgccaagagtgttttca
>8f6c97c6e67d416849d4d3021c8ea99e\_6
agctccaatagcgtatattaaagttgttgcagttaaaaagctcgtagttggatttcggagtgggccagttggtccgccgcaaggtgtgttactgactggtttgctcttcttcgcaaagactgcgtgtgctctttattgagtgtgcgtaggatttacgacgtttactttgaaaaaattgagtgttcaaagcaggctattgcttgaatacatgagcatggaataatggaataggattttggtcccatttttgttggtttctaggaccgaagtaatgattaagagggacaattgggggcatccgtatttcgttgtcagaggtgaaattcttggatttacgaaagacgaacaactgcgaaagcacttgccaagagtgttttca
>a1ee7cb9667bd4598dc2d02ba492ac40\_6
agctccaatagcgtatattaaagttgttgcagttaaaaagctcgtagttggatttcggagtgggccagttggtccgccgcaaggtgtgttactgactggtctgctcttcttcgcaaagactgcgtgtgctctttgttgagtgtgcgtaggatttacgacgtttactttgaaaaaattagagtgttcaaagcaggctattgcttgaatacatgagcatggaataatggaataggactttggtcccattttgttggtttctaggaccgaagtaatgattaagagggacaattggggcatccgtatttcgttgtcagaggtgaaattcttggatttacgaaaagacgaacaactgcgaaagcatttgccaagagtgttttca
>a7d7e129638d1062635819cca259ae32\_6
agctccaatagcgtatattaaagttgttgcagttaaaaagctcgtagtttggatttcggagtgggcccgttggtccgccgcaaggtgtgttactgactggtctgctcttcttcgcaaagactgcgtgtgctctttgttgagtgtgcgtaggatttacgacgtttactttgaaaaaattagagtgttcaaagcaggctattgcttgaatacatgagcatggaataatggaataggactttggtcccattttgttggtttctaggaccgaagtaatgattaagagggacaattgggggcatccgtatttcgttgtcagaggtgaaattcttggatttacgaaagacgaacaactgcgaaagcacttgccaagagtgttttca
>ae14c57bd0a829bbf48ea21e0194bd3a\_6
agctccaatagcgtatattaaagttgttgcagttaaaaagctcgtagttggatttcggagtgggccagttggtccgccgcaaggtgtgttactgactggtctgctcttcttcgcaaagactgcgtgtgctctttgttgagtgtgcgtaggatttacgacgtttacttgaaaaaaattgagtgttcaaagcaggctattgcttgaatacatgagcatggaataatggaataggactttggtcccattttgttggtttctaggaccgaagtaatgattaagagggacaattgggggcatccgtatttcgttgtcagaggtgaaattcttggatttacgaaagacgaacaactgcgaaagcatttgccaagagtgttttca
>bd4469fd935c84dae4d0889bad8016f4\_6
agctccaatagcgtatattaaagttgttgcagttaaaaagctcgtagttggatttcggagtgggccagttggtccgccgcaaggtgtgttactgactggtctgctcttcttcgcaaagactgcgtgtgctctttgttgagtgtgcgtaggatttacgacgtttactttgaaaaaattagagtgttcaaagcaggctattgcttgaatacatgagcatggaataatggaataggactttggtcccattttgttggtttctaggaccgaagtaatgattaagagggacaattggggcatccgtatttcgttgtcagaggtgaaattcttggatttacgaaagacgaacaactgcgaaagcacttgccaagagtgttttca
>ce9c0977bbdbafdf3ecc5d103540b359\_6
agctccaatagcgtatattaaagttgttgcagttaaaaagctcgtagttggatttcggagtgggccagttggtccgccgcaaggtgtgttactgactggtctgctcttcttcgcaaagactgcgtgtgctctttgttgagtgtgcgtaggatttacgacgtttactttgaaaaaattagagtgttcaaagcaggctattgcttgaatacatgagcatggaataatggaataggactttggtcccattttgttggtttctaggaccgaagtaatgattaagagggacaattgggggcatccgtattcgttgtcagaggtgaaattcttggatttacgaaagacgaacaactgcgaaagcatttgccaagagtgttttca
>eee099766309bf0f4de66f6c427d1875\_6
agctccaatagcgtatattaaagttgttgcagttaaaaagctcgtagttggatttcggagtgggcccgttggtccgccgcaaggtgtgttactgactggtctgctcttcttcgcaaaagactgcgtgtgctctttgttgagtgtgcgtaggatttacgacgtttactttgaaaaaaattagagtgttcaaagcaggctattgcttgaatacatgagcatggaataatggaataggactttggtcccattttgttggtttctaggaccgaagtaatgattaagagggacaattgggggcatccgtatttcgttgtcagaggtgaaattcttggatttacgaaagacgaacaactgcgaaagcacttgccaagagtgttttca
>ef301d08ec9be3b8c4dc58cac3011847\_6
agctccaatagcgtatattaaagttgttgcagttaaaaaagctcgtagttggatttcggagtgggccagttggtccgccgcaaggtgtgttactgactggtttgctcttcttcgcaaagactgcgtgtgctctttgttgagtgtgcgtaggatttacgacgtttactttgaaaaaattagagtgttcaaagcaggctattgcttgaatacatgagcatggaataatggaataggactttggtcccattttgttggtttctaggaccgaagtaatgattaagagggacaattgggggcatccgtatttcgttgtcagaggtgaaattcttggatttacgaaagacgaacaactgcgaaagcacttgccaagagtgttttca
>fe69c2e7553724e4a9827c489442c8b3\_6
agctccaatagcgtatattaaagttgttgtggttaaaaagctcgtagttggatttcggagtgggccagttggtccgccgcaaggtgtgttactgactggtctgctcttcttcgcaaagactgcgtgtgctctttgttgagtgtgcgtaggatttacgacgtttactttgaaaaaattagagtgttcaaagcaggctattgcttgaatacatgagcatggaataatggaataggactttggtcccattttgttggtttctaggaccgaagtaatgattaagagggacaattgggggcatccgtatttcgttgtcagaggtgaaattcttggatttacgaaagacgaacaactgcgaaagcacttgccaagagtgttttca
>05e96d782828e5f00fc24ebb53701949\_5
agctccactagcgtatattaaagttgctgcagttaaaaagctcgtagttggatttcggagtgggcccgttggtccgccgcaaggtgtgttactgactggtctgctcttcttcgcaaagactgcgtgtgctctttgttgagtgtgcgtaggatttacgacgtttactttgaaaaaattagagtgttcaaagcaggctattgcttgaatacatgagcatggaataatggaataggactttggtcccattttgttggtttctaggaccgaagtaatgattaagagggacaattgggggcatccgtatttcgttgtcagaggtgaaattcttggatttacgaaagacgaacaactgcgaaagcacttgccaagagtgttttca
>07de66a78d24c6083cc20ac479c63184\_5
agctccaatagcgtatattaaagttgttgcagttaaaaagctcgtagttggatttcggagtgggccagttggtccgccgcaaggtgtgttactgactggtttgctcttcttcgcaaagactgcgtgtgctctttattgagtgtgcgtaggatttacgacgtttactttgaaaaaattagagtgttcaaagcaggctattgcttgaatacatgagcatggaataatggaataggattttggtcccattttgttggtttctaggaccgaagtaatgattaagagggacaattggggggcatccgtatttcgttgtcagaggtgaaattcttggatttacgaaagacgaacaactgcgaaagcacttgccaagagtgttttca
>08e5497c6c1ec7c1fe835071aeebeb45\_5
agctccaatagcgtatattaaagttgttgcagttaaaaagctcgtagttggatttcggagtgggccagttggtccgccgcaaggtgtgttactgactggtttgctcttcttcgcaaagactgcgtgtgctctttattgagtgtgcgtaggatttacgacgtttactttgaaaaaattagagtgttcaaagcaggctattgcttgaatacatgagcatggaataatggaataggattttggtcccattttgttggtttctaggaccgaagtaatgattaagagggacaattgggggcatccgtatttcgttgtcagaggtgaaattcttggatttacgaaaagacgaacaactgcgaaagcacttgccaagagtgttttca
>0a0816c4d3e21bf0c89e6f12a1a5c8f4\_5
agctccaatagcgtatattaaagttgttgcagttaaaaagctcgtagttggatttcggagtgggccagttggtccgccgcaaggtgtgttactgactggtctgctcttcttcgcaaagactgcgtgtgctctttgttgagtgtgcgtaggatttacgacgtttactttgaaaaattagagtgttcaaagcaggctattgcttgaatacatgagcatggaataatggaataggactttggtcccattttgttggtttctaggaccgaagtaatgattaagagggacaattgggggcatccgtatttcgttgtcagaggtgaaattcttggatttacgaaagacgaacaactgcgaaagcatttgccaagagtgttttca
>10d890a823d99642db14fc09ea8264f4\_5
agctccaatagcgtatattaaagttgttgcagttaaaaagctcgtagttggatttcggagtgggccagttggtccgccgcaaggtgtgttactgactggtctgctcttcttcgcaaagactgcgtgtgctctttgttgagtgtgcgtaggatttacgacgtttactttgaaaaaattagagtgttcaaagcaggctattgcttgaatacatgagcatggaataatggaataggactttggtcccattttgttggtttctaggaccgaagtaatgattaagagggacaattgggggcgatccgtatttcgttgtcagaggtgaaattcttggatttacgaaagacgaacaactgcgaaagcatttgccaagagtgttttca
>190bf82e86dee7ab6779e60d66920ba8\_5
agctccaatagcgtatattaaagttgttgtcagttaaaaagctcgtagttggatttcggagtgggccagttggtccgccgcaaggtgtgttactgactggtctgctcttcttcgcaaagactgcgtgtgctctttgttgagtgtgcgtaggatttacgacgtttactttgaaaaaattagagtgttcaaagcaggctattgcttgaatacatgagcatggaataatggaataggactttggtcccattttgttggtttctaggaccgaagtaatgattaagagggacaattgggggcatccgtatttcgttgtcagaggtgaaattcttggatttacgaaagacgaacaactgcgaaaagcatttgccaagagtgttttca
>1d3b33066904e8a86c224687c721dd77\_5
agctccaatagcgtatattaaagttgttgcagttaaaaaagctcgtagttggatttcggagtgggcccgttggtccgccgcaaggtgtgttactgactggtctgctcttcttcgcaaagactgcgtgtgctctttgttgagtgtgcgtaggatttacgacgtttactttgaaaaaattagagtgttcaaagcaggctattgcttgaatacatgagcatggaataatggaataggactttggtcccattttgttggtttctaggaccgaagtaatgattaagagggacaattgggggcatccgtatttcgttgtcagaggtgaaatttcttggatttacgaaagacgaacaactgcgaaagcacttgccaagagtgttttca
>234a6ee64af4ab02ececf56b1d3c0f10\_5
agctccaatagcgtatattaaagttgttgcagttaaaaagctcgtagttggatttcggagtgggccagttggtccgccgcaaggtgtgttactgactggtctgctcttcttcgcaaagactgcgtgtgctctttgttgagtgtgcgtaggatttacgacgtttactttgaaaaaattagagtgttcaaagcaggctattgcttgaatacatgagcatggaataatggaataggactttggtcccattttgttggtttctaggaccgaagtaatgattaagagggacaattgggggcatccgtatttcgttgtcagaggtgaaattcttggatttacgaaagacgaacaactgcgaaagcacttgccaagagtgtttca
>3208df695594ab86f7a48d029a5a18f6\_5
agctccaatagcgtatattaaagttgttgcagttaaaaagctcgtagttggatttcggagtgggccagttggtccgccgcaaggtgtgttactgactggtttgctcttcttcgcaaagactgcgtgtgctctttattgagtgtgcgtaggatttacgacgtttactttgaaaaaaattagagtgttcaaagcaggctattgcttgaatacatgagcatggaataatggaataggattttggtcccattttgttggtttctaggaccgaagtaatgattaagagggacaattgggggcatccgtatttcgttgtcagaggtgaaattcttggatttacgaaagacgaacaactgcgaaagcacttgccaagagtgtttca
>3f1b0620a05bd3fced90e70c68ee1c44\_5
agctccaatagcgtatattaaagttgttgcagttaaaaagctcgtagttggatttcggagtgggcccgttggtccgccgcaaggtgtgttactgactggtctgctcttcttcgcaaagactgcgtgtgctctttgttgagtgtgcgtaggatttacgacgtttactttgaaaaaattagagtgttcaaagcaggctattgcttgaatacatgagcatggaataatggaataggactttggtcccattttgttggtttctagggccgaagtaatgattaagagggacaattgggggcatccgtatttcgttgtcagaggtgaaattcttggatttacgaaagacgaacaactgcgaaagcacttgccaagagtgttttca
>4a59853724d5ac3c37087dcf0bc3eb55\_5
agctccaatagcgtatactaaagttgttgcggttaaaaagctcgtagttggatttcggagtgggccagttggtccgccgcaaggtgtgttactgactggtctgctcttcttcgcaaagactgcgtgtgctctttgttgagtgtgcgtaggatttacgacgtttactttgaaaaaattagagtgttcaaagcaggctattgcttgaatacatgagcatggaataatggaataggactttggtcccattttgttggtttctaggaccgaagtaatgattaagagggacaattgggggcatccgtatttcgttgtcagaggtgaaattcttggatttacgaaagacgaacaactgcgaaagcatttgccaagagtgttttca
>4da68f81458306ba2248172791cbd098\_5
agctccaatagcgttatattaaagttgttgcagttaaaaagctcgtagttggatttcggagtgggcccgttggtccgccgcaaggtgtgttactgactggtctgctcttcttcgcaaagactgcgtgtgctctttgttgagtgtgcgtaggatttacgacgtttactttgaaaaaattagagtgttcaaagcaggctattgcttgaatacatgagcatggaataatggaataggactttggtcccattttgttggtttctaggaccgaagtaatgattaagagggacaattgggggcatccgtatttcgttgtcagaggtgaaattcttggatttacgaaagacgaacaactgcgaaagcacttgccaagagtgttttca
>5c984acc73ab37b924eaf55e29c7fb0e\_5
agctccaatagcgtatattaaagttgttgcagttaaaaagctcgtagttggatttcggagtgggccagttggtccgccgcaaggtgtgttactgactggtttgctcttcttcgcaaagactgcgtgtgctctttattgagtgtgcgtaggatttacgacgtttactttgaaaaaaattagagtgttcaaagcaggctattgcttgaatacatgagcatggaataatggaataggattttggtcccattttgtggtttctaggaccgaagtaatgattaagagggacaattgggggcatccgtatttcgttgtcagaggtgaaattcttggatttacgaaagacgaacaactgcgaaagcacttgccaagagtgttttca
>61e812936c1544de08c633980ba7ab1f\_5
agctccaatagcgtatattaaagttgttgcagttaaaaagctcgtagttggatttcggagtgggccagttggtccgccgcaaggtgtgttactgactggtctgctcttcttcgcaaagactgcgtgtgctctttgttgagtgtgcgtaggatttacgacgtttactttgaaaaaaattagagtgttcaaagcaggctattgcttgaatacatgagcatggaataatggaataggactttggtcccattttgttggtttctaggaccgaagtaatgattaagagggacaattgggggcatccgtatttcgttgtcagaggtgaaattcttggatttacgaaagacgaacaactgcgaaaagcatttgccaagagtgttttca
>6998db3743c6c12a856b825c1579ed0a\_5
agctccaatagcgtatattaaagttgttgcagttaaaaagctcgtagttggatttcggagtgggccagttggtccgccgcaaggtgtgttactgactggtttgctcttcttcgcaaagactgcgtgtgctctttgttgagtgtgcgtaggatttacgacgtttactttgaaaaaattagagtgttcaaagcaggctattgcttgaatacatgagcatggaataatggaataggactttggtcccattttgttggtttctaggaccgaagtaatgattaagagggacaattggggcatccgtatttcgttgtcagaggtgaaattcttggatttacgaaagacgaacaactgcgaaagcacttgccaagagtgttttca
>6b49631bae69f8cca5b5271514191073\_5
agctccaatagcgtatattaaagttgttgcagttaaaaagctcgtagttggatttcggagtgggccagttggtccgccgcaaggtgtgttactgactggtttgctcttcttcgcaaagactgcgtgtgctctttattgagtgtgcgtaggatttacgacgtttactttgaaaaaattagagtgttcaaagcaggctattgcttgaatacatgagcatggaataatggaataggattttggtcccattttgttggtttctaggaccgaagtaatgattaagagggacaattgggggcgatccgtatttcgttgtcagaggtgaaattcttggatttacgaaagacgaacaactgcgaaagcacttgccaagagtgttttca
>71863311b17c4ebfd8694a7a34dc04a1\_5
agctccaatagcgtatattaaagttgttgcagttaaaaagctcgtagttggatttcggagtgggccagttggtccgccgcaaggtgtgttactgactggtctgctcttcttcgcaaagactgcgtgtgctctttgttgagtgtgcgtaggatttacgacgtttactttgaaaaaattagagtgttcaaagcaggctattgcttgaatacatgagcatggaataatggaataggactttggtcccattttgttggtttctaggaccgaagtaatgattaagagggacaattgggggcatccgtatttcgttgtcagaggtgaaatttcttggatttacgaaaagacgaacaactgcgaaagcatttgccaagagtgttttca
>723e086d11edd3363cd0c3adfbe8888b\_5
agctccaatagcgtatattaaagttgttgcagttaaaaagctcgtagttggatttcggagtgggccagttggtccgccgcaaggtgtgttactgactggtttgctcttcttcgcaaagactgcgtgtgctctttattgagtgtgcgtaggatttacgacgtttactttgaaaaaattagagtgttcaaagcaggctattgcttgaatacatgagcatggaataatggaataggattttggtcccattttgttggttctaggaccgaagtaatgattaagagggacaattgggggcatccgtatttcgttgtcagaggtgaaattcttggatttacgaaagacgaacaactgcgaaagcacttgccaagagtgttttca
>77f1fef11601f804ad83f4c50d913f88\_5
agctccaatagcgtatattaaagttgttgcagttaaaaaagctcgtagttggatttcggagtgggccagttggtccgccgcaaggtgtgttactgactggtttgctcttcttcgcaaagactgcgtgtgctctttattgagtgtgcgtaggatttacgacgtttactttgaaaaaattagagtgttcaaagcaggctattgcttgaatacatgagcatggaataatggaataggattttggtcccattttgttggtttctaggaccgaagtaatgattaagagggacaattgggggcatccgtatttcgttgtcagaggtgaaattcttggatttacgaaagacgaacaactgcgaagcacttgccaagagtgttttca
>79f5696b5edc3db508a88b4db92aa5f6\_5
agctccaatagcgtatattaaagtgttgcagttaaaaagctcgtagttggatttcggagtgggccagttggtccgccgcaaggtgtgttactgactggtctgctcttcttcgcaaagactgcgtgtgctctttgttgagtgtgcgtaggatttacgacgtttactttgaaaaaattagagtgttcaaagcaggctattgcttgaatacatgagcatggaataatggaataggactttggtcccattttgttggtttctaggaccgaagtaatgattaagagggacaattgggggcatccgtatttcgttgtcagaggtgaaattcttggatttacgaaagacgaacaactgcgaaagcatttgccaagagtgttttca
>81e07adb5a02935fd04f2dce534186f1\_5
agctccaatagcgtatattaaagttgttgcagttaaaaagctcgtagttggatttcggagtgggcccgttggtccgccgcaaggtgtgttactgactggtctgctcttcttcgcaaagactgcgtgtgctctttgttgagtgtgcgtaggatttacgacgtttactttgaaaaaattagagtgttcaaagcaggctattgcttgaatacatgagcatggaataatggaataggactttggtcccattttgttggtttctaggaccgaagtaatgattaagagggacaattgggggcatccgtatttcgtgtcagaggtgaaattcttggatttacgaaagacgaacaactgcgaaagcacttgccaagagtgttttca
>84ef11abecb58b6febae4a101720be29\_5
agctccaatagcgtatattaaagttgttgcagtttaaaaagctcgtagttggatttcggagtgggcccgttggtccgccgcaaggtgtgttactgactggtctgctcttcttcgcaaagactgcgtgtgctctttgttgagtgtgcgtaggatttacgacgtttactttgaaaaaattagagtgttcaaagcaggctattgcttgaatacatgagcatggaataatggaataggactttggtcccattttgttggtttctaggaccgaagtaatgattaagagggacaattgggggcatccgtatttcgttgtcagaggtgaaattcttggatttacgaaagacgaacaactgcgaaaagcacttgccaagagtgttttca
>95c42cb7a85bae481354bf09600e3bbd\_5
agctccaatagcgtatattaaagttgttgcagttaaaaagctcgtagttggatttcggagtgggcccgttggtccgccgcaaggtgtgttactgactggtctgctcttcttcgcaaagactgcgtgtgctctttgttgagtgtgcgtaggatttacgacgtttactttgaaaaaattagagtgttcaaagcaggctattgcttgaatacatgagcatggaataatggaataggactttggtcccattttgttggtttctaggaccgaagtaatgattaagagggacaattgggggcatccgtatttcgttgtcagaggtgaaattcttggatttacgaaagacgaacaactgcgaagcacttgccaagagtgttttca
>976ab15591bf4b3ad2fdf873d511282a\_5
agctccaatagcgtatactaaagttgttgcggttaaaaagctcgtagttggatttcggagtgggcccgttggtccgccgcaaggtgtgttactgactggtctgctcttcttcgcaaagactgcgtgtgctctttgttgagtgtgcgtaggatttacgacgtttactttgaaaaaattagagtgttcaaagcaggctattgcttgaatacatgagcatggaataatggaataggactttggtcccattttgttggtttctaggaccgaagtaatgattaagagggacaattgggggcatccgtatttcgttgtcagaggtgaaattcttggatttacgaaagacgaacaactgcgaaagcacttgccaagagtgttttca
>9a60c948b79c38d8ce33a80ba64140bf\_5
agctccaatagcgtatattaaagttgttgcagttaaaaagctcgtagttggatttcggagtgggccagttggtccgccgcaaggtgtgttactgactggtctgctcttcttcgcaaagactgcgtgtgctctttgttgagtgtgcgtaggatttacgacgtttactttgaaaaaattgagtgttcaaagcaggctattgcttgaatacatgagcatggaataatggaataggactttggtcccattttgttggtttctaggaccgaagtaatgattaagagggacaattgggggcatccgtatttcgttgtcagaggtgaaattcttggatttacgaaagacgaacaactgcgaaagcacttgccaagagtgttttca
>a6bd8da45a3d636da7c63c95069e65ab\_5
agctccaatagcgtatattaaagttgttgcagttaaaaagctcgtagttggatttcggagtgggccagttggtccgccgcaaggtgtgttactgactggtctgctcttcttcgcaaagactgcgtgtgctctttgttgagtgtgcgtaggatttacgacgtttactttgaaaaaattagagtgttcaaagcaggctattgcttgaatacatgagcatggaataatggaataggactttggtcccattttgttggtttctaggaccgaagtaatgattaagagggacaattgggggcatccgtatttcgttgtcagaggtgaaattcttggatttacgaaagacgaacaactgcgaaaagcacttgccaagagtgttttca
>b3e5044a167c5eb604179af12b74bc84\_5
agctccaatagcgtatattaaagttgttgcagttaaaaagctcgtagttggatttcggagtgggccagttggtccgccgcaaggtgtgttactgactggtttgctcttcttcgcaaagactgcgtgtgctctttattgagtgtgcgtaggatttacgacgtttactttgaaaaaattgagtgttcaaagcaggctattgcttgaatacatgagcatggaataatggaataggattttggtcccattttgtggtttctaggaccgaagtaatgattaagagggacaattgggggcatccgtatttcgttgtcagaggtgaaattcttggatttacgaaagacgaacaactgcgaaagcacttgccaagagtgttttca
>b90db9b1cf956c0fc763e9e91337ea35\_5
agctccaatagcgtatattaaagttgttgcagtttaaaaagctcgtagttggatttcggagtgggccagttggtccgccgcaaggtgtgttactgactggtctgctcttcttcgcaaagactgcgtgtgctctttgttgagtgtgcgtaggatttacgacgtttactttgaaaaaattagagtgttcaaagcaggctattgcttgaatacatgagcatggaataatggaataggactttggtcccattttgttggtttctaggaccgaagtaatgattaagagggacaattgggggcatccgtatttcgttgtcagaggtgaaattcttggatttacgaaagacgaacaactgcgaaagcatttgccaagagtgttttca
>c1288fe1fd6d18bd649e060b07dee13b\_5
agctccaatagcgtatattaaagttgttgcagttaaaagctcgtagttggatttcggagtgggcccgttggtccgccgcaaggtgtgttactgactggtctgctcttcttcgcaaagactgcgtgtgctctttgttgagtgtgcgtaggatttacgacgtttactttgaaaaaattagagtgttcaaagcaggctattgcttgaatacatgagcatggaataatggaataggactttggtcccattttgttggtttctaggaccgaagtaatgattaagagggacaattgggggcatccgtatttcgttgtcagaggtgaaatttcttggatttacgaaagacgaacaactgcgaaagcacttgccaagagtgttttca
>c1c3f20e46a290f1675304f252a6fd7d\_5
agctccaatagcgtatattaaagttgttgcagttaaaaagctcgtagttggatttcggagtgggccagttggtccgccgcaaggtgtgttactgactggtttgctcttcttcgcaaagactgcgtgtgctctttattgagtgtgcgtaggatttacgacgtttactttgaaaaaattagagtgttcaaagcaggctattgcttgaatacatgagcatggaataatggaataggattttggtcccattttgttggtttctaggaccgaagtaatgattaagagggacaattgggggcatccgtatttcgttgtcagaggtgaaattcttggatttacgaaagacgaacaactgcgaaagcacttgccaagagtgttttcat
>c2cf5a869c2aacb2b6675cc547de7695\_5
agctccaatagcgtatactaatgttgttgcagttaaaaagctcgtagttggatttcggagtgggcccgttggtccgccgcaaggtgtgttactgactggtctgctcttcttcgcaaagactgcgtgtgctctttgttgagtgtgcgtaggatttacgacgtttactttgaaaaaattagagtgttcaaagcaggctattgcttgaatacatgagcatggaataatggaataggactttggtcccattttgttggtttctaggaccgaagtaatgattaagagggacaattgggggcatccgtatttcgttgtcagaggtgaaattcttggatttacgaaagacgaacaactgcgaaagcacttgccaagagtgttttca
>c782b0f26a53142d3f013403973b0c0b\_5
agctccaatagcgtatattaaagttgttgcagttaaaaagctcgtagttggatttcggagtgggcccgttggtccgccgcaaggtgtgttactgactggtctgctcttcttcgcaaagactgcgtgtgctctttgttgagtgtgcgtaggatttacgacgtttactttgaaaaaattagagtgttcaaagcaggctattgcttgaatacatgagcatggaataatggaataggactttggtcccattttgttggtttctaggaccgaagtaatgattaagagggacaattgggggcatccgtatttcgttgtcaggaggtgaaatttcttggatttacgaaagacgaacaactgcgaaagcacttgccaagagtgttttca
>c8b189f82d87060e3922d29a07b05746\_5
agctccaatagcgtatattaaagttgttgcagttaaaaagctcgtagttggatttcggagtgggccagttggtccgccgcaaggtgtgttactgactggtctgctcttcttcgcaaagactgcgtgtgctctttgttgagtgtgcgtaggatttacgacgtttactttgaaaaaattagagtgttcaaagcaggctattgcttgaatacatgagcatggaataatggaataggactttggtcccattttgttggtttctaggaccgaagtaatgattaagagggacaattgggggcatccgtatttcgttgtcagaggtgaattcttggatttacgaaagacgaacaactgcgaaagcatttgccaagagtgttttca
>cf9877d9996118a97ce1213923c28625\_5
agctccaatagcgtatattaaagttgttgcagttaaaaagctcgtagttggatttcggagtgggccagttggtccgccacaaggtgtgttactgactggtttgctcttcttcgcaaagactgcgtgtgctctttattgagtgtgcgtaggatttacgacgtttactttgaaaaaattagagtgttcaaagcaggctattgcttgaatacatgagcatggaataatggaataggattttggtcccattttgttggtttctaggaccgaagtaatgattaagagggacaattgggggcatccgtatttcgttgtcagaggtgaaattcttggatttacgaaagacgaacaactgcgaaagcacttgccaagagtgttttca
>e15f8ad2721f7b81ccac15b2714246e9\_5
agctccaatagcgtatattaaagttgttgcagttaaaaaagctcgtagttggatttcggagtgggcccgttggtccgccgcaaggtgtgttactgactggtctgctcttcttcgcaaagactgcgtgtgctctttgttgagtgtgcgtaggatttacgacgtttactttgaaaaaattagagtgttcaaagcaggctattgcttgaatacatgagcatggaataatggaataggactttggtcccattttgttggtttctaggaccgaagtaatgattaagagggacaattgggggcatccgtatttcgttgtcagaggtgaaattcttggatttacgaaagacgaacaactgcgaaagcacttgccaagagtgtttca
>ed97a27a566c18b8aa1daa2158a73762\_5
agctccaatagcgtatattaaagttgttgcagttaaaaaagctcgtagttggatttcggagtgggccagttggtccgccgcaaggtgtgttactgactggtttgctcttcttcgcaaagactgcgtgtgctctttgttgagtgtgcgtaggatttacgacgtttactttgaaaaaaattagagtgttcaaagcaggctattgcttgaatacatgagcatggaataatggaataggactttggtcccattttgttggtttctaggaccgaagtaatgattaagagggacaattgggggcatccgtatttcgttgtcagaggtgaaattcttggatttacgaaagacgaacaactgcgaaagcacttgccaagagtgttttca
>fa84617e3301bf5dd3bcc1317225d12d\_5
agctccaatagcgtatattaaagttgttgcagttaaaaagctcgtagttggatttcggagtgggcccgttggtccgccgcaaggtgtgttactgactggtctgctcttcttcgcaaagactgcgtgtgctctttgttgagtgtgcgtaggatttacgacgtttactttgaaaaaaattagagtgttcaaagcaggctattgcttgaatacatgagcatggaataatggaataggactttggtcccattttgttggtttctaggaccgaagtaatgattaagagggacaattgggggcatccgtatttcgttgtcagaggtgaaattcttggatttacgaaagacgaacaactgcgaaagcatttgccaagagtgttttca
>fba748906c488d8433a7cc50a8dad700\_5
agctccaatagcgtatattaaagttgttgcagttaaaaagctcgtagttggatttcggagtgggccagttggtccgccgcaaggtgtgttactgactggtttgctcttcttcgcaaagactgcgtgtgctctttattgagtgtgcgtaggatttacgacgtttactttgaaaaaattagagtgttcaaagcaggctattgcttgaatacatgagcatggaataatggaataggattttggtcccattttgttggtttctaggaccgagtaatgattaagagggacaattgggggcatccgtatttcgttgtcagaggtgaaattcttggatttacgaaagacgaacaactgcgaaagcacttgccaagagtgttttca
>fbfa809a84e4f26585efd20177d9723e\_5
agctccaatagcgtatattaaagttgttgcagttaaaaagctcgtagttggatttcggagtgggcccgttggtccgccgcaaggtgtgttactgactggtctgctcttcttcgcaaagactgcgtgtgctctttgttgagtgtgcgtaggatttacgacgttttactttgaaaaaaattagagtgttcaaagcaggctattgcttgaatacatgagcatggaataatggaataggactttggtcccattttgttggtttctaggaccgaagtaatgattaagagggacaattgggggcatccgtatttcgttgtcagaggtgaaattcttggatttacgaaagacgaacaactgcgaaagcacttgccaagagtgttttca
>04ff243a09620fd0301f49faa16b38ba\_4
agctccaatagcgtatattaaagttgttgcagttaaaaagctcgtagttggatttcggagtgggcccgttggtccgccgcaaggtgtgttactgactggtctgctcttcttcgcaaagactgcgtgtgctctttgttgagtgtgcgtaggatttacgacgtttactttgaaaaaattagagtgttcaaagcaggctattgcttgaatacatgagcatggaataatggaataggactttggtcccatttttgttggtttctaggaccgaagtaatgattaagagggacaattgggggcatccgtatttcgttgtcagaggtgaaatttcttggatttacgaaagacgaacaactgcgaaagcacttgccaagagtgttttca
>06d5928b66e33311af554684a1c94a96\_4
agctccaatagcgtatattaaagttgttgcagttaaaaagctcgtagttggatttcggagtgggccagttggtccgccgcaaggtgtgttactgactggtctgctcttcttcgcaaaagactgcgtgtgctctttgttgagtgtgcgtaggatttacgacgtttactttgaaaaaattagagtgttcaaagcaggctattgcttgaatacatgagcatggaataatggaataggactttggtcccattttgttggtttctaggaccgaagtaatgattaagagggacaattgggggcatccgtatttcgttgtcagaggtgaaattcttggatttacgaaagacgaacaactgcgaaagcatttgccaagagtgttttca
>09a8807282e83d37db34d36a5bb55401\_4
agctccaatagcgtatattaaagttgttgcagttaaaaagctcgtagttggatttcggagtgggccagttggtccgccgcaaggtgtgttactgactggtttgctcttcttcgcaaagactgcgtgtgctctttattgagtgtgcgtaggatttacgacgtttacttgaaaaaattgagtgttcaaagcaggctattgcttgaatacatgagcatggaataatggaataggattttggtcccattttgttggtttctaggaccgaagtaatgattaagagggacaattgggggcatccgtatttcgttgtcagaggtgaaattcttggatttacgaaagacgaacaactgcgaaagcacttgccaagagtgttttca
>16d5ac9a35d5ea44b20a6026c56c3374\_4
agctccaatagcgtatactaaagttgttgcagttaaaaagctcgtagttggatttcggagtgggccagttggtccgccgcaaggtgtgttactgactggtctgctcttcttcgcaaagactgcgtgtgctctttgttgagtgtgcgtaggatttacgacgtttactttgaaaaaattagagtgttcaaagcaggctattgcttgaatacatgagcatggaataatggaataggactttggtcccattttgttggtttctaggaccgaagtaatgattaagagggacaattgggggcatccgtatttcgttgtcagaggtgaaattcttggatttacgaaagacgaacaactgcgaaagcatttgccaagagtgttttca
>16f2e2aafde7de32470ca8efb95bba29\_4
agctccaatagcgtatattaaagttgttgcagttaaaagctcgtagttggatttcggagtgggcccgttggtccgccgcaaggtgtgttactgactggtctgctcttcttcgcaaagactgcgtgtgctctttgttgagtgtgcgtaggatttacgacgtttactttgaaaaattagagtgttcaaagcaggctattgcttgaatacatgagcatggaataatggaataggactttggtcccattttgttggtttctaggaccgaagtaatgattaagagggacaattgggggcatccgtatttcgttgtcagaggtgaaatttcttggatttacgaaagacgaacaactgcgaaagcacttgccaagagtgttttca
>1f65c50a6840f5c949d9e3a95497d820\_4
agctccaatagcgtatattaaagttgttgcaagttaaaaagctcgtagttggatttcggagtgggcccgttggtccgccgcaaggtgtgttactgactggtctgctcttcttcgcaaagactgcgtgtgctctttgttgagtgtgcgtaggatttacgacgtttactttgaaaaaattagagtgttcaaagcaggctattgcttgaatacatgagcatggaataatggaataggactttggtcccattttgttggtttctaggaccgaagtaatgattaagagggacaattgggggcatccgtatttcgttgtcagaggtgaaattcttggatttacgaaagacgaacaactgcgaaagcacttgccaagagtgttttca
>22656fb1dd9cd3bc7895716c6a174efa\_4
agctccaatagcgtatattaaagttgttgcagttaaaaaagctcgtagttggatttcggagtgggcccgttggtccgccgcaaggtgtgttactgactggtctgctcttcttcgcaaagactgcgtgtgctctttgttgagtgtgcgtaggatttacgacgtttactttgaaaaaattgagtgttcaaagcaggctattgcttgaatacatgagcatggaataatggaataggactttggtcccattttgttggtttctaggaccgaagtaatgattaagagggacaattgggggcatccgtatttcgttgtcagaggtgaaattcttggatttacgaaagacgaacaactgcgaaagcacttgccaagagtgttttca
>2898cd54d77995f61e71036173512d7f\_4
agctccaatagcgtatattaaagttgttgcagttaaaaagctcgtagttggatttcggagtgggccagttggtccgccgcaaggtgtgttactgactggtctgctcttcttcgcaaagactgcgtgtgctctttgttgagtgtgcgtaggattttacgacgtttactttgaaaaaattagagtgttcaaagcaggctattgcttgaatacatgagcatggaataatggaataggactttggtcccattttgttggtttctaggaccgaagtaatgattaagagggacaattgggggcatccgtatttcgttgtcagaggtgaaattcttggatttacgaaagacgaacaactgcgaaagcatttgccaagagtgttttca
>360f4e7a031017f5734cd69b694141cf\_4
agctccaatagcgtatattaaagttgttgcagttaaaaagctcgtagttggatttcggagtgggccagttggtccgccgcaaggtgtgttactgactggtctgctcttcttcgcaaagactgcgtgtgctctttgttgagtgtgcgtaggatttacgacgtttactttgaaaaaattagagtgttcaaagcaggctattgcttgaatacatgagcatggaataatggaataggactttggtcccattttgttggtttctaggaccgaagtaatgattaagagggacaattgggggcatccgtatttcgttgtcagaggtgaaattcttggatttacgaaagacgaacaactgcgaaagcaatttgccaagagtgttttca
>3c6c3b66faaf681c266f2ba46ea64fba\_4
agctccaatagcgtatattaaagttgttgcagttaaaaagctcgtagttggatttcggagtgggccagttggtccgccgcaaggtgtgttactgactggtttgctcttcttcgcaaagactgcgtgtgctctttattgagtgtgcgtaggatttacgacgtttactttgaaaaaattagagtgttcaaagcaggctattgcttgaatacatgagcatggaataatggaataggattttggtcccattttgttggtttctaggaccgaagtaatgattaagagggacaattgggggcatccgtatttcgttgtcagaggtgaaattcttggatttacgaaagacgaacaactgcgaaagcacttgccaagagtgttttaca
>40a74abace31aef83bbd24b93047c3f4\_4
agctccaatagcgtatattaaagttgttgcagttaaaaagctcgtagttggatttcggagtgggcccgttggtccgccgcaaggtgtgttactgactggtctgctcttcttcgcaaagactgcgtgtgctctttgttgagtgtgcgtaggatttacgacgtttactttgaaaaaattagagtgttcaaagcaggctattgcttgaatacatgagcatggaataatggaataggactttggtcccattttgttggtttctaggaccgaagtaatgattaagagggacaattggggcatccgtatttcgtgtcagaggtgaaattcttggatttacgaaagacgaacaactgcgaaagcacttgccaagagtgttttca
>40d3cfc6328fb329edd4162d3e39c817\_4
agctccaatagcgtatattaaagttgttgcagttaaaaagctcgtagttggatttcggagtgggccagttggtccgccgcaaggtgtgttactgactggtctgctcttcttcgcaaagactgcgtgtgctctttgttgagtgtgcgtaggatttacgacgtttactttgaaaaaattagagtgttcaaagcaggctattgcttgaatacatgagcatggaataatggaataggactttggtcccattttgttggtttctaggaccgaagtaatgattaagagggacaattgggggcatccgtatttcgttgtcagaggtgaaattcttggatttacgaaagacgaacaactgcgaaagcattttgccaagagtgttttca
>471b37088ea0df83beeb5d4a8cd155d3\_4
agctccaatagcgtatattaaagttgttgcagttaaaaagctcgtagttggatttcggagtgggccagttggtccgccgcaaggtgtgttactgactggtttgctcttcttcgcaaagactgcgtgtgctctttattgagtgtgcgtaggatttacgacgtttaatttgaaaaaattagagtgttcaaagcaggctattgcttgaatacatgagcatggaataatggaataggattttggtcccattttgttggtttctaggaccgaagtaatgattaagagggacaattgggggcatccgtatttcgttgtcagaggtgaaattcttggatttacgaaagacgaacaactgcgaaagcacttgccaagagtgttttca
>51bb210f0fdc0f00aac6b49f2aca8cd6\_4
agctccaatagcgtatattaaagttgttgcagttaaaaaagctcgtagttggatttcggagtgggcccgttggtccgccgcaaggtgtgttactgactggtctgctcttcttcgcaaagactgcgtgtgctctttgttgagtgtgcgtaggatttacgacgtttactttgaaaaaattgagtgttcaaagcaggctattgcttgaatacatgagcatggaataatggaataggactttggtcccattttgttggtttctaggaccgaagtaatgattaagagggacaattgggggcatccgtatttcgttgtcagaggtgaaatttcttggatttacgaaagacgaacaactgcgaaagcacttgccaagagtgttttca
>5ea81e5bc9ba5578d839628f41fa296c\_4
agctccaatagcgtatattaaagttgttgcagttaaaaagctcgtagttggatttcggagtgggcccgttggtccgccgcaaggtgtgttactgactggtctgctcttcttcgcaaagactgcgtgtgctctttgttgagtgtgcgtaggatttacgacgtttactttgaaaaaattagagtgttcaaagcaggctattgcttgaatacatgagcatggaataatggaataggactttggtcccattttgttggtttctaggaccgaagtaatgattaagagggacaattgggggcatccgtatttcgttgtcagaggtgaaattcttggatttacgaaagacgaacaaactgcgaaagcacttgccaagagtgttttca
>6aa6cf50b32a0e46cc06f1b82947bd8d\_4
agctccaatagcgtatattaaagttgttgtggttaaaaagctcgtagttggatttcggagtgggccagttggtccgccgcaaggtgtgttactgactggtttgctcttcttcgcaaagactgcgtgtgctctttgttgagtgtgcgtaggatttacgacgtttactttgaaaaaattagagtgttcaaagcaggctattgcttgaatacatgagcatggaataatggaataggactttggtcccattttgttggtttctaggaccgaagtaatgattaagagggacaattgggggcatccgtatttcgttgtcagaggtgaaattcttggatttacgaaagacgaacaactgcgaaagcacttgccaagagtgttttca
>6c5ac7fb3f9196e7fff5691a64a1af35\_4
agctccaatagcgtatattaaagttgttgcagttaaaaaagctcgtagttggatttcggagtgggccagttggtccgccgcaaggtgtgttactgactggtttgctcttcttcgcaaagactgcgtgtgctctttattgagtgtgcgtaggatttacgacgtttactttgaaaaaaattagagtgttcaaagcaggctattgcttgaatacatgagcatggaataatggaataggattttggtcccattttgttggtttctaggaccgaagtaatgattaagagggacaattgggggcatccgtatttcgttgtcagaggtgaaattcttggatttacgaaagacgaacaactgcgaaagcacttgccaagagtgtttca
>7539a4560a258cf9cf1495152509debb\_4
agctccaatagcgtatattaaagttgttgcagttaaaaagctcgtagttggatttcggagtgggccagttggtccgccgcaaggtgtgttactgactggtctgctcttcttcgcaaaagactgcgtgtgctctttgttgagtgtgcgtaggatttacgacgtttactttgaaaaaattagagtgttcaaagcaggctattgcttgaatacatgagcatggaataatggaataggactttggtcccattttgttggtttctaggaccgaagtaatgattaagagggacaattgggggcatccgtatttcgttgtcagaggtgaaatttcttggatttacgaaagacgaacaactgcgaaagcatttgccaagagtgttttca
>7781701dbc3bc8b7267f86f3375e0e8f\_4
agctccaatagcgtatattaaagttgttgcagttaaaaaagctcgtagttggatttcggagtgggcccgttggtccgccgcaaggtgtgttactgactggtctgctcttcttcgcaaagactgcgtgtgctctttgttgagtgtgcgtaggatttacgacgtttactttgaaaaaattagagtgttcaaagcaggctattgcttgaatacatgagcatggaataatggaataggactttggtcccattttgttggtttctaggaccgaagtaatgattaagagggacaattgggggcatccgtatttcgttgtcagaggtgaaattcttggatttacgaaagacgaacaactgcgaaagcatttgccaagagtgttttca
>79f6e9bb71d1d18584225fbc24525c5a\_4
agctccaatagcgtatattaaagttgttgcagttaaaaagctcgtagttggatttcggagtgggccagttggtccgccgcaaggtgtgttactgactggtttgctcttcttcgcaaagactgcgtgtgctctttattgagtgtgcgtaggatttacgacgttttactttgaaaaaattgagtgttcaaagcaggctattgcttgaatacatgagcatggaataatggaataggattttggtcccattttgttggtttctaggaccgaagtaatgattaagagggacaattgggggcatccgtatttcgttgtcagaggtgaaattcttggatttacgaaagacgaacaactgcgaaagcacttgccaagagtgttttca
>7b8e3e2437e39e0bf679cd305466c0e6\_4
agctccaatagcgtatattaaagttgttgcagttaaaaagctcgtagttggatttcggagtgggccagttggtccgccgcaaggtgtgttactgactggtctgctcttcttcgcaaagactgcgtgtgctctttgttgagtgtgcgtaggatttacgacgtttactttgaaaaaattgagtgttcaaagcaggctattgcttgaatacatgagcatggaataatggaataggactttggtcccattttgttggtttctaggaccgaagtaatgattaagagggacaattggggcatccgtatttcgttgtcagaggtgaaattcttggatttacgaaagacgaacaactgcgaaagcatttgccaagagtgtttca
>7ed22fcb193553e6f02a2397a5bf05b4\_4
agctccaatagcgtatattaaagttgttgcagttaaaaagctcgtagttggatttcggagtgggccagttggtccgccgcaaggtgtgttactgactggtctgctcttcttcgcaaagactgcgtgtgctctttgttgagtgtgcgtaggatttacgacgtttactttgaaaaaattagagtgttcaaagcaggctattgcttgaatacatgagcatggaataatggaataggactttggtcccattttgttggtttctaggaccgaagtaatgattaagagggacaattgggggcatccgtatttcgtgtcagaggtgaaattctggatttacgaaagacgaacaactgcgaaagcatttgccaagagtgttttca
>8c25ee303b5b8985f93a02a6fce743ec\_4
agctccaatagcgtatattaaagttgttgcagttaaaaagctcgtagttggatttcggagtgggccagttggtccgccgcaaggtgtgttactgactggtctgctcttcttcgcaaagactgcgtgtgctctttgttgagtgtgcgtaggatttacgacgtttactttgaaaaaattagagtgttcaaagcaggctattgcttgaatacatgagcatggaataatggaataggactttggtcccattttgttggtttctaggaccgaagtaatgattaagagggacaattgggggcatccgtatttcgttgtcagaggtgaaatttcttggatttacgaaagacgaacaactgcgaaagcacttgccaagagtgttttca
>8c2996fe48de7b22e190f64ea4e897a8\_4
agctccaatagcgtatattaaagttgttgcagttaaaaagctcgtagttggatttcggagtgggcccgttggtccgccgcaaggtgtgttactgactggtctgctcttcttcgcaaagactgcgtgtgctctttgttgagtgtgcgtaggatttacgacgtttactttgaaaaaattagagtgttcaaagcaggctattgcttgaatacatgagcatggaataatggaataggactttggtcccattttgttggtttctaggaccgaagtaatgattaagagggacaattggggcatccgtatttcgttgtcagaggtgaaattcttggatttacgaaagacgaacaactgcgaaagcatttgccaagagtgttttca
>8ee70ab89883149c3324b5ffd64a5204\_4
agctccaatagcgtatattaaagttgttgcagttaaaaagctcgtagttggatttcggagtgggccagttggtccgccgcaaggtgtgttactgactggtttgctcttcttcgcaaagactgcgtgtgctctttattgagtgtgcgtaggatttacgacgtttactttgaaaaaattagagtgttcaaagcaggctattgcttgaatacatgagcatggaataatggaataggattttggtcccattttgttggtttctaggaccgaagtaatgattaagagggacaattggggcatccgtatttcgtgtcagaggtgaaattcttggatttacgaaagacgaacaactgcgaaagcacttgccaagagtgttttca
>9e99953d516e7cb06dd7d766e489febf\_4
agctccaatagcgtatattaaagttgttgcagttaaaaaagctcgtagttggatttcggagtgggccagttggtccgccgcaaggtgtgttactgactggtctgctcttcttcgcaaagactgcgtgtgctctttgttgagtgtgcgtaggatttacgacgtttactttgaaaaaattagagtgttcaaagcaggctattgcttgaatacatgagcatggaataatggaataggactttggtcccattttgttggtttctaggaccgaagtaatgattaagagggacaattgggggcatccgtatttcgttgtcagaggtgaaattcttggatttacgaaagacgaacaactgcgaaagcatttgaccaagagtgttttca
>a18a586e5cb91fba03c2858880a5141c\_4
agctccaatagcgtatattaaagttgttgcagttaaaaagctcgtagttggatttcggagtgggccagttggtccgccgcaaggtgtgttactggactggtttgctcttcttcgcaaagactgcgtgtgctctttattgagtgtgcgtaggatttacgacgtttactttgaaaaaattagagtgttcaaagcaggctattgcttgaatacatgagcatggaataatggaataggattttggtcccattttgttggtttctaggaccgaagtaatgattaagagggacaattgggggcatccgtatttcgttgtcagaggtgaaattcttggatttacgaaagacgaacaactgcgaaagcacttgccaagagtgttttca
>a2fa564b423ccbc0e20f8943f4eb0ecf\_4
agctccaatagcgtatattaaagttgttgcagttaaaaaagctcgtagttggatttcggagtgggccagttggtccgccgcaaggtgtgttactgactggtttgctcttcttcgcaaagactgcgtgtgctctttattgagtgtgcgtaggatttacgacgttttactttgaaaaaattagagtgttcaaagcaggctattgcttgaatacatgagcatggaataatggaataggattttggtcccattttgttggtttctaggaccgaagtaatgattaagagggacaattgggggcatccgtatttcgttgtcagaggtgaaattcttggatttacgaaagacgaacaactgcgaaagcacttgccaagagtgttttca
>a4995ff0499c128dae31877d9c230ab0\_4
agctccaatagcgtatattaaagttgttgcagttaaaaaagctcgtagttggatttcggagtgggccagttggtccgccgcaaggtgtgttactgactggtttgctcttcttcgcaaagactgcgtgtgctctttattgagtgtgcgtaggatttacgacgtttactttgaaaaaattagagtgttcaaagcaggctattgcttgaatacatgagcatggaataatggaataggatttggtcccattttgttggtttctaggaccgaagtaatgattaagagggacaattggggcatccgtatttcgttgtcagaggtgaaattcttggatttacgaaagacgaacaactgcgaaagcacttgccaagagtgtttca
>a7f28259efc27c0a956cef25a40f03a3\_4
agctccaatagcgtatattaaagttgttgcagttaaaaagctcgtagttggatttcggagtgggccagttggtccgccgcaaggtgtgttactgactggtctgctcttcttcgcaaagactgcgtgtgctctttgttgagtgtgcgtaggatttacgacgtttactttgaaaaaattagagtgttcaaagcaggctattgcttgaatacatgagcatggaataatggaataggactttggtcccattttgttggtttctaggaccgaagtaatgattaagagggacaattgggggcatccgtatttcattgtcagaggtgaaattcttggatttacgaaagacgaacaactgcgaaagcatttgccaagagtgttttca
>a9ead853195329595f66e4b0bb2d6225\_4
agctccaatagcgtatattaaagttgttgcagttaaaaagctcgtagttggatttcggagtgggccagttggtccgccgcaaggtgtgttactgactggtttgctcttcttcgcaaagactgcgtgtgctctttattgagtgtgcgtaggatttacgacgttttactttgaaaaaaattagagtgttcaaagcaggctattgcttgaatacatgagcatggaataatggaataggattttggtcccattttgttggtttctaggaccgaagtaatgattaagagggacaattgggggcatccgtatttcgttgtcagaggtgaaattcttggatttacgaaagacgaacaactgcgaaagcacttgccaagagtgttttca
>b0b57dad981cd7e1529ae5e8c421e7d7\_4
agctccaatagcgtatattaaagttgttgcagttaaaaagctcgtagttggatttcggagtgggcccgttggtccgccgcaaggtgtgttactgactggtctgctcttcttcgcaaagactgcgtgtgctctttgttgagtgtgcgtaggatttacgacgtttactttagaaaaaattagagtgttcaaagcaggctattgcttgaatacatgagcatggaataatggaataggactttggtcccattttgttggtttctaggaccgaagtaatgattaagagggacaattgggggcatccgtatttcgttgtcagaggtgaaattcttggatttacgaaagacgaacaactgcgaaagcacttgccaagagtgttttca
>b3b6e2dff15e5d208c6f8e3a87588cd1\_4
agctccaatagcgtatattaaagttgttgcagttaaaaagctcgtagttggatttcggagtgggccagttggtccgccgcaaggtgtgttactgactggtctgctcttcttcgcaaagactgcgtgtgctctttgttgagtgtgcgtaggatttacgacgtttactttgaaaaaattagagtgttcaaagcaggctattgcttgaatacatgagcatggaataatggaataggactttggtcccattttgttggtttctaggaccgaagtaatgattaagagggacaattggggcatccgtatttcgttgtcagaggtgaaattcttggatttacgaaagacgaacaactgcgaaaagcatttgccaagagtgttttca
>b68083758580b720e2901a14fef6937a\_4
agctccaatagcgtatattaaagttgttgcagttaaaaagctcgtagttggatttcggagtgggcccgttggtccgccgcaaggtgtgttactgactggtctgctcttcttcgcaaagactgcgtgtgctctttgttgagtgtgcgtaggatttacgacgtttactttgaaaaaattagagtgttcaaagcaggctattgcttgaatacatgagcatggaataatggaataggactttggtcccattttgttggtttctaggaccgaagtaatgattaagagggacaattgggggcatccgtatttcgttgtcagaggtgaaattcttggatttacgaaagacgaacaactgcgaaagcacttgccaagagtgttttcat
>b97e1dd67b60fc87434f37f259dd938f\_4
agctccaatagcgtatattaaagttgttgcagttaaaaagctcgtagttggatttcggagtgggccagttggtccgccgcaaggtgtgttactgactggtttgctcttcttcgcaaagactgcgtgtgctctttattgagtgtgcgtaggatttacgacgtttactttgaaaaaattagagtgttcaaagcaggctattgcttgaatacatgagcatggaataatggaataggattttggtcccattttgttggtttctaggaccgaagtaatgattaagagggacaattgggggcatccgtatttcgttgtcagaggtgaaattcttggatttacgaaagacgaacaactgacgaaagcacttgccaagagtgttttca
>c2eecc199d90c1f9e671c10901d5e0a5\_4
agctccaatagcgtatattaaagttgttgcagttaaaaagctcgtagttggatttcggagtgggccagttggtccgccgcaaggtgtgttactgactggtttgctcttcttcgcaaagactgcgtgtgctctttattgagtgtgcgtaggatttacgacgtttactttgaaaaaattagagtgttcaaagcaggctattgcttgaatacatgagcatggaataatggaataggattttggtcccattttgttggtttctaggtaccgaagtaatgattaagagggacaattgggggcatccgtatttcgttgtcagaggtgaaattcttggatttacgaaagacgaacaactgcgaaagcacttgccaagagtgttttca
>ca04bec5e7bb584e986fd6f3855554f8\_4
agctccaatagcgtatattaaagttgttgcagttaaaaagctcgtagttggatttcggagtgggccagttggtccgccgcaaggtgtgttactgactggtctgctcttcttcgcaaagactgcgtgtgctctttgttgagtgtgcgtaggatttacgacgtttactttgaaaaaattagagtgttcaaagcaggctattgcttgaatacatgagcatggaataatggaataggactttggtcccattttgttggtttctaggaccgaagtaatgattaagagggacaattgggggcatccgtatttcgttgtcagaggtgaaattcttggatttacgaaagacgaacaactgcgaagcatttgccaagagtgttttca
>cdd2e2abaaa1e6fd8d079d0c578a3f30\_4
agctccaatagcgtatattaaagttgttgcagttaaaaagctcgtagttggatttcggagtgggcccgttggtccgccgcaaggtgtgttactgactggtctgctcttcttcgcaaaagactgcgtgtgctctttgttgagtgtgcgtaggatttacgacgtttactttgaaaaaattagagtgttcaaagcaggctattgcttgaatacatgagcatggaataatggaataggactttggtcccattttgttggtttctaggaccgaagtaatgattaagagggacaattgggggcatccgtatttcgttgtcagaggtgaaatttcttggatttacgaaagacgaacaactgcgaaagcacttgccaagagtgttttca
>d40bd0063d892d9237f520e2ddbc744f\_4
agctccaatagcgtatattaaagttgttgcagttaaaaagctcgtagttggatttcggagtgggcccgttggtccgccgcaaggtgtgttactgactggtctgctcttcttcgcaaagactgcgtgtgctctttgttgagtgtgcgtaggatttacgacgtttactttgaaaaaaattagagtgttcaaagcaggctattgcttgaatacatgagcatggaataatggaataggactttggtcccattttgttggtttctaggaccgaagtaatgattaagagggacaattgggggcatccgtattcgttgtcagaggtgaaattcttggatttacgaaagacgaacaactgcgaaagcacttgccaagagtgttttca
>d6bcf924a4558706025fca9d03492db9\_4
agctccaatagcgtatattaaagttgttgcagttaaaaagctcgtagttggatttcggagtgggcccgttggtccgccgcaaggtgtgttactgactggtctgctcttcttcgcaaagactgcgtgtgctctttgttgagtgtgcgtaggatttacgacgtttactttgaaaaaattagagtgttcaaagcaggctattgcttgaatacatgagcatggaataatggaataggactttggtcccattttgttggtttctaggaccgaagtaatgattaagagggacaattgggggcatccgtatttcgttgtcagaggtgaattcttggatttacgaaagacgaacaactgcgaaagcacttgccaagagtgttttca
>da44cf8a5a330d2f4657f1c328860e95\_4
agctccaatagcgtatattaaagttgttgcagttaaaaagctcgtagttggattttcggagtgggccagttggtccgccgcaaggtgtgttactgactggtctgctcttcttcgcaaagactgcgtgtgctctttgttgagtgtgcgtaggatttacgacgtttactttgaaaaaattagagtgttcaaagcaggctattgcttgaatacatgagcatggaataatggaataggactttggtcccattttgttggtttctaggaccgaagtaatgattaagagggacaattgggggcatccgtatttcgttgtcagaggtgaaattcttggatttacgaaagacgaacaactgcgaaagcatttgccaagagtgttttca
>db853c5c5104f07829e9c673e14f7215\_4
agctccaatagcgtatattaaagttgttgcagttaaaaagctcgtagttggatttcggagtgggccagttggtccgccgcaaggtgtgttactgactggtttgctcttcttcgcaaagactgcgtgtgctctttattgagtgtgcgtaggatttacgacgtttactttgaaaaaaattagagtgttcaaagcaggctattgcttgaatacatgagcatggaataatggaataggattttggtcccatttttgttggtttctaggaccgaagtaatgattaagagggacaattgggggcatccgtatttcgttgtcagaggtgaaattcttggatttacgaaagacgaacaactgcgaaagcacttgccaagagtgttttca
>dc6f1f65b167521204c07e8dbf1dcc51\_4
agctccaatagcgtatattaaagttgttgtggttaaaaagctcgtagttggatttcggagtgggccagttggtccgccgcaaggtgtgttactgactggtttgctcttcttcgcaaagactgcgtgtgctctttattgagtgtgcgtaggatttacgacgtttactttgaaaaaattagagtgttcaaagcaggctattgcttgaatacatgagcatggaataatggaataggattttggtcccattttgttggtttctaggaccgaagtaatgattaagagggacaattgggggcatccgtatttcgttgtcagaggtgaaattcttggatttacgaaagacgaacaactgcgaaagcacttgccaagagtgttttca
>e3d26c69fee215ba252547317d48533a\_4
agctccaatagcgtatattaaagttgttgcagttaaaaagctcgtagttggatttcggagtgggccagttggtccgccgcaaggtgtgttactgactggtttgctcttcttcgcaaagactgcgtgtgctctttattgagtgtgcgtaggatttacgacgtttactttgaaaaaattagagtgttcaaagcaggctattgcttgaatacatgagcatggaataatggaataggattttggtcccattttgttggtttctaggaccgaagtaatgattaagagggacaattggggcatccgtatttcgttgtcagaggtgaattcttggatttacgaaagacgaacaactgcgaaagcacttgccaagagtgttttca
>f538f6f904afc074b1ca74a9ec94faa9\_4
agctccaatagcgtatattaaagttgttgcagttaaaaagctcgtagttggatttcggagtgggcccgttggtccgccgcaaggtgtgttactgactggtctgctcttcttcgcaaagactgcgtgtgctctttgttgagtgtgcgtaggatttacgacgtttactttgaaaaaaattagagtgttcaaagcaggctattgcttgaatacatgagcatggaataatggaataggactttggtcccattttgttggtttctaggaccgaagtaatgattaagagggacaattgggggcatccgtatttcgttgtcagaggtgaaattcttggatttacgaaagacgaacaactgcgaaaagcacttgccaagagtgttttca
>f741c544ff08693f8739b0a56f6f1f4d\_4
agctccaatagcgtatattaaagttgttgcagttaaaaaagctcgtagttggatttcggagtgggcccgttggtccgccgcaaggtgtgttactgactggtctgctcttcttcgcaaagactgcgtgtgctctttgttgagtgtgcgtaggatttacgacgtttactttgaaaaaattagagtgttcaaagcaggctattgcttgaatacatgagcatggaataatggaataggactttggtcccattttgttggtttctaggaccgaagtaatgattaagagggacaattgggggcatccgtatttcgttgtcagaggtgaaattcttggatttacgaaagacgaacaactgcgaaaagcacttgccaagagtgttttca
>ffbaa0e1d20c3f065f8c04a10368054e\_4
agctccaatagcgtatattaaagttgttgcagttaaaaagctcgtagttggatttcggagtgggccagttggtccgccgcaaggtgtgttactgactggtctgctcttcttcgcaaagactgcgtgtgctctttgttgagtgtgcgtaggatttacgacgtttactttgaaaaaattgagtgttcaaagcaggctattgcttgaatacatgagcatggaataatggaataggactttggtcccattttgttggtttctaggaccgaagtaatgattaagagggacaattgggggcatccgtatttcgttgtcagaggtgaaatttcttggatttacgaaagacgaacaactgcgaaagcatttgccaagagtgttttca
>01aefe031ec1869331ce5717f033ce47\_3
agctccaatagcgtatattaaagttgttgcagttaaaaagctcgtagttggatttcggagtgggccagttggtccgccgcaaggtgtgttactgactggtttgctcttcttcgcaaagactgcgtgtgctctttattgagtgtgcgtaggatttacgacgtttactttgaaaaaattagagtgttcaaagcaggctattgcttgaatacatgagcatggaataatggaataggattttggtcccattttgtggtttctaggaccgaagtaatgattaagagggacaattgggggcatccgtatttcgtgtcagaggtgaaattcttggatttacgaaagacgaacaactgcgaaagcacttgccaagagtgttttca
>0793ac8d4e2fb5e83fb03e5b5c112391\_3
agctccaatagcgtatattaaagttgttgcagttaaaagctcgtagttggatttcggagtgggcccgttggtccgccgcaaggtgtgttactgactggtctgctcttcttcgcaaagactgcgtgtgctctttgttgagtgtgcgtaggatttacgacgtttactttgaaaaattagagtgttcaaagcaggctattgcttgaatacatgagcatggaataatggaataggactttggtcccattttgttggtttctaggaccgaagtaatgattaagagggacaattgggggcatccgtatttcgttgtcagaggtgaaattcttggatttacgaaagacgaacaactgcgaaagcacttgccaagagtgttttca
>0f172c8f52e3f949397ab7293c31aac7\_3
agctccaatagcgtatattaaagttgttgcagttaaaaagctcgtagttggatttcggagtgggccagttggtccgccgcaaggtgtgttactgactggtttgctcttcttcgcaaagactgcgtgtgctctttattgagtgtgcgtaggatttacgacgtttactttgaaaaaattgagtgttcaaagcaggctattgcttgaatacatgagcatggaatatggaataggattttggtcccattttgttggtttctaggaccgaagtaatgattaagagggacaattgggggcatccgtatttcgttgtcagaggtgaaattcttggatttacgaaagacgaacaactgcgaaagcacttgccaagagtgttttca
>119318c0d9f8c71275e453a0fac43347\_3
agctccaatagcgtatattaaagttgttgcagttaaaaagctcgtagttggatttcggagtgggccagttggtccgccgcaaggtgtgttactgactggtctgctcttcttcgcaaagactgcgtgtgctctttgttgagtgtgcgtaggatttacgacgtttactttgaaaaaaattagagtgttcaaagcaggctattgcttgaatacatgagcatggaataatggaataggactttggtcccattttgttggtttctaggaccgaagtaatgattaagagggacaattgggggcatccgtatttcgttgtcagaggtgaaatttcttggatttacgaaagacgaacaactgcgaaagcatttgccaagagtgttttca
>1acf2f316eaae05d792aa1a544c43ebc\_3
agctccaatagcgtatattaaagttgttgcagttaaaaagctcgtagttggatttcggagtgggcccgttggtccgccgcaaggtgtgttactgactggtctgctcttcttcgcaaagactgcgtgtgctctttgttgagtgtgcgtaggatttacgacgtttacttgaaaaaattagagtgttcaaagcaggctattgcttgaatacatgagcatggaataatggaataggactttggtcccattttgttggtttctaggaccgaagtaatgattaagagggacaattgggggcatccgtatttcgttgtcagaggtgaaattcttggatttacgaaagacgaacaactgcgaaagcacttgccaagagtgttttca
>1b9bc4508f93608cb3056850310e0914\_3
agctccaatagcgtatattaaagttgttgcagttaaaaaagctcgtagttggatttcggagtgggccagttggtccgccgcaaggtgtgttactgactggtttgctcttcttcgcaaagactgcgtgtgctctttattgagtgtgcgtaggatttacgacgtttactttgaaaaaattagagtgttcaaagcaggctattgcttgaatacatgagcatggaataatggaataggattttggtcccattttgttggtttctaggaccgaagtaatgattaagagggacaattgggggcatccgtatttcgttgtcagaggtgaaattcttggatttacgaaagacgaacaactgcgaagcacttgccaagagtgtttca
>24ce4a77a97a6dca41902f380069cc8c\_3
agctccaatagcgtatattaaagttgttgtggttaaaaagctcgtagttggatttcggagtgggcccgttggtccgccgcaaggtgtgttactgactggtctgctcttcttcgcaaagactgcgtgtgctctttgttgagtgtgcgtaggatttacgacgtttactttgaaaaaaattagagtgttcaaagcaggctattgcttgaatacatgagcatggaataatggaataggactttggtcccattttgttggtttctaggaccgaagtaatgattaagagggacaattgggggcatccgtatttcgttgtcagaggtgaaattcttggatttacgaaagacgaacaactgcgaaagcacttgccaagagtgttttca
>2811cd91cacbae71511f2d6939a17acd\_3
agctccaatagcgtatattaaagttgttgtggttaaaaagctcgtagttggatttcggagtgggcccgttggtccgccgcaaggtgtgttactgactggtctgctcttcttcgcaaagactgcgtgtgctctttgttgagtgtgcgtaggatttacgacgtttactttgaaaaaattagagtgttcaaagcaggctattgcttgaatacatgagcatggaataatggaataggactttggtcccattttgttggtttctaggaccgaagtaatgattaagagggacaattgggggcatccgtatttcgttgtcagaggtgaaatttcttggatttacgaaagacgaacaactgcgaaagcacttgccaagagtgttttca
>2a01a1d499da76aa6a35fb7c6df44c7a\_3
agctccaatagcgtatattaaagttgttgcagttaaaaagctcgtagttggatttcggagtgggccagttggtccgccgcaaggtgtgttactgactggtctgctcttcttcgcaaagactgcgtgtgctctttgttgagtgtgcgtaggatttacgacgtttactttgaaaaaaattgagtgttcaaagcaggctattgcttgaatacatgagcatggaataatggaataggactttggtcccattttgttggtttctaggaccgaagtaatgattaagagggacaattgggggcatccgtatttcgttgtcagaggtgaaattcttggatttacgaaagacgaacaactgcgaaagcacttgccaagagtgttttca
>37cba0ad78e8702d40dce510bb54c850\_3
agctccaatagcgtatattaaagttgttgcagttaaaaagctcgtagttggatttcggagtgggccagttggtccgccgcaaggtgtgttactgactggtctgctcttcttcgcaaagactgcgtgtgctctttgttgagtgtgcgtaggatttacgacgtttactttgaaaaaattagagtgttcaaagcaggctattgcttgaatacatgagcatggaataatggaataggactttggtcccattttgttggtttctaggaccgaagtaatgattaagagggacaattgggggcatccgtatttcgtgtcagaggtgaaattcttggatttacgaaagacgaacaactgcgaaagcacttgccaagagtgttttca
>3f36ff54de62c3ada051e0518fa3ae1c\_3
agctccaatagcgtatattaaagttgttgcagttaaaaaagctcgtagttggatttcggagtgggcccgttggtccgccgcaaggtgtgttactgactggtctgctcttcttcgcaaagactgcgtgtgctctttgttgagtgtgcgtaggatttacgacgtttactttgaaaaaattagagtgttcaaagcaggctattgcttgaatacatgagcatggaataatggaataggactttggtcccattttgttggtttctaggaccgaagtaatgattaagagggacaattgggggcatccgtatttcgttgtcagaggtgaattcttggatttacgaaagacgaacaactgcgaaagcacttgccaagagtgttttca
>437df272b066ec2b7466c96579d70fe7\_3
agctccaatagcgtatattaaagttgttgcagttaaaaagctcgtagttggatttcggagtgggcccgttggtccgccgcaaggtgtgttactgactggtctgctcttcttcgcaaagactgcgtgtgctctttgttgagtgtgcgtaggatttacgacgtttactttgaaaaaattagagtgttcaaagcaggctattgcttgaatacatgagcatggaataatggaataggactttggtcccattttgttggtttctaggaccgaagtaatgattaagagggacaattggggcatccgtatttcgttgtcagaggtgaaattcttggatttacgaaagacgaacaactgcgaaagcacttgccaagagtgtttca
>48de28ca71a093e7e511168110bb3bd1\_3
agctccaatagcgtatattaaagttgttgcagttaaaaagctcgtagttggatttcggagtgggccagttggtccgccgcaaggtgtgttactgactggtttgctcttcttcgcaaagactgcgtgtgctctttattgagtgtgcgtaggatttacgacgtttactttgaaaaaattagagtgttcaaagcaggctattgcttgaatacatgagcatggaataatggaataggattttggtcccattttgttggtttctaggaccgaagtaatgattaagagggacaattgggggcatccgtatttcgttgtcagaggtgaaattcttggatttacgaaagacgaacaactgcgaaagcacttgaccaagagtgttttca
>49e2fd3b718c0889c479dbdb91f136ee\_3
agctccaatagcgtatattaaagttgttgcagttaaaaagctcgtagttggatttcggagtgggccagttggtccgccgcaaggtgtgttactgactggtttgctcttcttcgcaaagactgcgtgtgctctttattgagtgtgcgtaggatttacgacgtttactttgaaaaaattagagtgttcaaagcaggctattgcttgaatacatgagcatggaataatggaataggattttggtccctattttgttggtttctaggaccgaagtaatgattaagagggacaattgggggcatccgtatttcgttgtcagaggtgaaattcttggatttacgaaagacgaacaactgcgaaagcacttgccaagagtgttttca
>4af6228d9201cca1c4c49a358b2102da\_3
agctccaatagcgtatattaaagttgttgcagttaaaaagctcgtagttggatttcggagtgggcccgttggtccgccgcaaggtgtgttactgactggtctgctcttcttcgcaaagactgcgtgtgctctttgttgagtgtgcgtaggatttacgacgtttacttgaaaaaattgagtgttcaaagcaggctattgcttgaatacatgagcatggaataatggaataggactttggtcccattttgttggtttctaggaccgaagtaatgattaagagggacaattgggggcatccgtatttcgttgtcagaggtgaaattcttggatttacgaaagacgaacaactgcgaaagcacttgccaagagtgtttca
>5ce52167bc122c4b813d52afc72f3bd2\_3
agctccaatagcgtatattaaagttgttgcagttaaaaagctcgtagttggatttcggagtgggccagttggtccgccgcaaggtgtgttactgactggtttgctcttcttcgcaaagactgcgtgtgctctttattgagtgtgcgtaggatttacgacgtttactttgaaaaaattagagtgttcaaagcaggctattgcttgaatacatgagcatggaataatggaataggattttggtcccattttgttggtttctaggaccgaagtaatgattaagagggacaattgggggcatccgtatttcgttgtcagaggtgaaattcttggatttacgaaagacgaacaactgcgaaagcacttgccagagtgtttca
>5e08420371f6d0c508ed382aec0dd47c\_3
agctccaatagcgtatattaaagttgttgcagttaaaaagctcgtagttggatttcggagtgggccagttggtccgccgcaaggtgtgttactgactggtttgctcttcttcgcaaagactgcgtgtgctctttgttgagtgtgcgtaggatttacgacgtttactttgaaaaaaattgagtgttcaaagcaggctattgcttgaatacatgagcatggaataatggaataggactttggtcccattttgttggtttctaggaccgaagtaatgattaagagggacaattgggggcatccgtatttcgttgtcagaggtgaaattcttggatttacgaaagacgaacaactgcgaaagcacttgccaagagtgttttca
>5ee0eb537db4499948e09facb432d509\_3
agctccaatagcgtatattaaagttgttgcagttaaaaagctcgtagttggatttcggagtgggccagttggtccgccgcaaggtgtgttactgactggtctgctcttcttcgcaaagactgcgtgtgctctttgttgagtgtgcgtaggatttacgacgtttactttgaaaaaattagagtgttcaaagcaggctattgcttgaatacatgagcatggaataatggaataggactttggtcccattttgttggtttctaggaccgaagtaatgattaagagggacaattgggggcatccgtatttcgttgtcagaggtgaaattcttggatttacgaaagacgaacactgcgaaagcatttgccaagagtgttttca
>6569ff2efd911b49bd934a891433d206\_3
agctccaatagcgtatattaaagttgttgcagttaaaaagctcgtagttggatttcggagtgggccagttggtccgccgcaaggtgtgttactgactggtttgctcttcttcgcaaagactgcgtgtgctctttattgagtgtgcgtaggatttacgacgtttactttgaaaaaattagagtgttcaaagcaggctattgcttgaatacatgagcatggaataatggaataggattttggtccattttgttggtttctaggaccgaagtaatgattaagagggacaattgggggcatccgtatttcgttgtcagaggtgaaattcttggatttacgaaagacgaacaactgcgaaagcacttgccaagagtgttttca
>67a3a4e3be2146a5a94d5d3bf643a09b\_3
agctccaatagcgtatattaaagttgttgcagttaaaaagctcgtagttggatttcggagtgggccagttggtccgccgcaaggtgtgttactgactggtttgctcttcttcgcaaagactgcgtgtgctctttattgagtgtgcgtaggatttacgacgtttactttgaaaaaaattagagtgttcaaagcaggctattgcttgaatacatgagcatggaataatggaataggatttttggtcccattttgttggtttctaggaccgaagtaatgattaagagggacaattgggggcatccgtatttcgtgtcagaggtgaaattcttggatttacgaaagacgaacaactgcgaaagcacttgccaagagtgttttca
>6a1ee9926f079dac659024d89cfd6ef0\_3
agctccaatagcgtatattaaagttgttgcagttaaaaagctcgtagttggatttcggagtgggccagttggtccgccgcaaggtgtgttactgactggtttgctcttcttcgcaaagactgcgtgtgctctttattgagtgtgcgtaggatttacgacgtttactttgaaaaaattagagtgttcaaagcaggctattgcttgaatacatgagcatggaataatggaataggattttggtcccattttgttggtttcttaggaccgaagtaatgattaagagggacaattgggggcatccgtatttcgttgtcagaggtgaaattcttggatttacgaaagacgaacaactgcgaaagcacttgccaagagtgttttca
>6a9ccea1c174a676fd89c032e4f2bd56\_3
agctccaatagcgtatattaaagttgttgcagttaaaaagctcgtagttggatttcggagtgggcccgttggtccgccgcaaggtgtgttactgactggtctgctcttcttcgcaaagactgcgtgtgctctttgttgagtgtgcgtaggatttacgacgtttactttgaaaaaattagagtgttcaaagcaggctattgcttgattacatgagcatggaataatggaataggactttggtcccattttgttggtttctaggaccgaagtaatgattaagagggacaattgggggcatccgtatttcgttgtcagaggtgaaattcttggatttacgaaagacgaacaactgcgaaagcacttgccaagagtgttttca
>6ce07ef533f29955d3610c852bbc7565\_3
agctccaatagcgtatattaaagttgttgcagttaaaaagctcgtagttggatttcggagtgggccagttggtccgccgcaaggtgtgttactgactggtctgctcttcttcgcaaagactgcgtgtgctctttgttgagtgtgcgtaggatttacgacgtttactttgaaaaaattagagtgttcaaagcaggctattgcttgaatacatgagcatggaataatggaataggactttggtcccattttgttggtttctagggccgaagtaatgattaagagggacaattgggggcatccgtatttcgttgtcagaggtgaaatttcttggatttacgaaagacgaacaactgcgaaagcacttgccaagagtgttttca
>7162fe1cec6182d1efd5bbf5124a3e8e\_3
agctccaatagcgtatattaaagttgttgcagttaaaaagctcgtagttggatttcggagtgggccagttggtccgccgcaaggtgtgttactgactggtttgctcttcttcgcaaagactgcgtgtgctctttattgagtgtgcgtaggatttacgacgtttactttgaaaaaattagagtgttcaaagcaggctattgcttgaatacatgagcatggaatatggaataggattttggtcccattttgttggtttctaggaccgaagtaatgattaagagggacaattgggggcatccgtatttcgttgtcagaggtgaaattcttggatttacgaaagacgaacaactgcgaaagcacttgccaagagtgttttca
>73027cf647491f02e990f3dad4fbc323\_3
agctccaatagcgtatattaaagtgttgcagttaaaaagctcgtagttggatttcggagtgggcccgttggtccgccgcaaggtgtgttactgactggtctgctcttcttcgcaaagactgcgtgtgctctttgttgagtgtgcgtaggatttacgacgtttactttgaaaaaattagagtgttcaaagcaggctattgcttgaatacatgagcatggaataatggaataggactttggtcccattttgttggtttctaggaccgaagtaatgattaagagggacaattgggggcatccgtatttcgttgtcagaggtgaaattcttggatttacgaaagacgaacaactgcgaaaagcacttgccaagagtgttttca
>76fc13f393a3dcaf28f63b31ca96513f\_3
agctccaatagcgtatattaaagttgttgcagttaaaaagctcgtagttggatttcggagtgggccagttggtccgccgcaaggtgtgttactgactggtttgctcttcttcgcaaagactgcgtgtgctctttattgagtgtgcgtaggatttacgacgtttactttgaaaaaattagagtgttcaaagcaggctattgcttgaatacatgagcatggaataatggaataggattttggtcccattttgttggtttctaggaccgaagtaatgattaagagggacaattgggggcatccgtatttcgttgtcagaggtgaaattcttggatttacgaaagacgaacaactgcgaaagcacttgccaaagagtgttttca
>7afe5584dbc7f1da1a911276e6d6655e\_3
agctccaatagcgtatattaaagttgttgcagttaaaaagctcgtagttggatttcggagtgggcccgttggtccgccgcaaggtgtgttactgactggtctgctcttcttcgcaaagactgcgtgtgctctttgttgagtgtgcgtaggatttacgacgtttactttgaaaaaattagagtgttcaaagcaggctattgcttgaatacatgagcatggaataatggaataggactttggtcccattttgttggtttctaggaccgaagtaatgattaagagggacaattgggggcatccgtatttcgttgtcagaggtgaaattcttggatttacgaagacgaacaactgcgaaagcacttgccaagagtgttttca
>7cbde82105137176e891edc5389d13ce\_3
agctccaatagcgtatattaaagttgttgcagttaaaaagctcgtagttggatttcggagtgggccagttggtccgccgcaaggtgtgttactgactggtttgctcttcttcgcaaagactgcgtgtgctctttattgagtgtgcgtaggatttacgacgtttactttgaaaaaaattgagtgttcaaagcaggctattgcttgaatacatgagcatggaataatggaataggattttggtcccattttgtggtttctaggaccgaagtaatgattaagagggacaattgggggcatccgtatttcgttgtcagaggtgaaattcttggatttacgaaagacgaacaactgcgaaagcacttgccaagagtgttttca
>7e84726f0151e971cfef9d479d6131dd\_3
agctccaatagcgtatattaaagttgttgcagttaaaaagctcgtagttggatttcggagtgggccagttggtccgccgcaaggtgtgttactgactggtctgctcttcttcgcaaagactgcgtgtgctctttgttgagtgtgcgtaggatttacgacgtttactttgaaaaaattagagtgttcaaagcaggctattgcttgaatacatgagcatggaataatggaataggactttggtcccattttgttggtttctaggaccgaagtaatgattaagagggacaattgggggcatccgtatttcgttgtcagaggtgaaattcttggatttacgaaagacgaacaactgcgaaagcacttgccaagaatgttttca
>7ec0f0e6ad83d84c004422779b128f27\_3
agctccaatagcgtatattaaagttgttgcagttaaaaagctcgtagttggatttcggagtgggcccgttggtccgccgcaaggtgtgttactgactggtctgctcttcttcgcaaagactgcgtgtgctctttgttgagtgtgcgtaggatttacgacgtttactttgaaaaaattgagtgttcaaagcaggctattgcttgaatacatgagcatggaataatggaataggactttggtcccattttgttggtttctaggaccgaagtaatgattaagagggacaattgggggcatccgtatttcgttgtcagaggtgaaatttcttggatttacgaaaagacgaacaactgcgaaagcacttgccaagagtgttttca
>81a032ad101618f15c0ed55ea8aa5ca2\_3
agctccaatagcgtatattaaagttgttgcagttaaaaaagctcgtagttggatttcggagtgggcccgttggtccgccgcaaggtgtgttactgactggtctgctcttcttcgcaaagactgcgtgtgctctttgttgagtgtgcgtaggatttacgacgtttactttgaaaaaaattagagtgttcaaagcaggctattgcttgaatacatgagcatggaataatggaataggactttggtcccattttgttggtttctaggaccgaagtaatgattaagagggacaattgggggcatccgtatttcgttgtcagaggtgaaattcttggatttacgaaagacgaacaactgcgaaagcacttgccaagagtgtttca
>847ba46433c1df64f6dab879304d1127\_3
agctccaatagcgtatattaaagttgttgcagttaaaaagctcgtagttggatttcggagtgggccagttggtccgccgcaaggtgtgttactgactggtctgctcttcttcgcaaagactgcgtgtgctctttgttgagtgtgcgtaggatttacgacgtttactttgaaaaaattagagtgttcaaagcaggctattgcttgaatacatgagcatggaataatggaataggactttggtcccattttgttggtttctaggaccgaagtaatgattaagagggacaattgggggcatccgtatttcgttgtcagaggtgaaattcttggatttacgaaagacgaacaactgcgaaagcatttgccaagagtgttttcat
>8492147757d663c9c43cd351b290de88\_3
agctccaatagtcgtatattaaagttgttgcagttaaaaagctcgtagttggatttcggagtgggcccgttggtccgccgcaaggtgtgttactgactggtctgctcttcttcgcaaagactgcgtgtgctctttgttgagtgtgcgtaggatttacgacgtttactttgaaaaaattagagtgttcaaagcaggctattgcttgaatacatgagcatggaataatggaataggactttggtcccattttgttggtttctaggaccgaagtaatgattaagagggacaattgggggcatccgtatttcgttgtcagaggtgaaattcttggatttacgaaagacgaacaactgcgaaagcacttgccaagagtgttttca
>89556637ebe6b445c2e51b3f52c7edf0\_3
agctccaatagcgtatattaaagttgttgcagttaaaaagctcgtagttggatttcggagtgggcccgttggtccgccgcaaggtgtgttactgactggtctgctcttcttcgcaaagactgcgtgtgctctttgttgagtgtgcgtaggatttacgacgtttactttgaaaaaattagagtgttcaaagcaggctattgcttgaatacatgagcatggaataatggaataggactttggtcccattttgttggtttctaggaccgaagtaatgattaagagggacaattggggcatccgtattcgttgtcagaggtgaaattcttggatttacgaaagacgaacaactgcgaaagcacttgccaagagtgttttca
>8e9a8092cc3fcf3817af0703dd694ff3\_3
agctccaatagcgtatattaaagttgttgtggttaaaaagctcgtagttggatttcggagtgggccagttggtccgccgcaaggtgtgttactgactggtctgctcttcttcgcaaagactgcgtgtgctctttgttgagtgtgcgtaggatttacgacgtttactttgaaaaaaattagagtgttcaaagcaggctattgcttgaatacatgagcatggaataatggaataggactttggtcccattttgttggtttctaggaccgaagtaatgattaagagggacaattgggggcatccgtatttcgttgtcagaggtgaaattcttggatttacgaaagacgaacaactgcgaaagcatttgccaagagtgttttca
>8fa34ddf4d835ab15b7e846a73ac7da1\_3
agctccaatagcgtatattaaagttgttgcagttaaaaagctcgtagttggatttcggagtgggccagttggtccgccgcaaggtgtgttactgactggtctgctcttcttcgcaaagactgcgtgtgctctttgttgagtgtgcgtaggatttacgacgtttactttgaaaaattagagtgttcaaagcaggctattgcttgaatacatgagcatggaataatggaataggactttggtcccattttgttggtttctaggaccgaagtaatgattaagagggacaattgggggcatccgtatttcgttgtcagaggtgaaattcttggatttacgaaagacgaacaactgcgaaagcatttgccaagagtgtttca
>973ce1800549703afbcd4a2428ef8c4f\_3
agctccaatagcgtatattaaagttgttgcagttaaaaagctcgtagttggatttcggagtgggcccgttggtccgccgcaaggtgtgttactgactggtctgctcttcttcgcaaagactgcgtgtgctctttgttgagtgtgcgtaggatttacgacgtttactttgaaaaaaattagagtgttcaaagcaggctattgcttgaatacatgagcatggaataatggaataggactttggtcccattttgttggtttctaggaccgaagtaatgattaagagggacaattgggggcatccgtatttcgttgtcagaggtgaaattcttggatttacgaaagacgaacaactgcgaaagcacttgccaagagtgtttca
>9adc07e6bb71d4286b499e534d3833a5\_3
agctccaatagcgtatattaaagttgttgcagttaaaaagctcgtagttggatttcggagtgggccagttggtccgccgcaaggtgtgttactgactggtttgctcttcttcgcaaagactgcgtgtgctctttattgagtgtgcgtaggatttacgacgtttactttgaaaaaattagagtgttcaaagcaggctattgcttgaatacatgagcatggaataatggaataggattttggtcccattttgttggtttctaggaccgaagtaatgattaagagggacaattgggggcatccgtatttcgttgtcagaggtgaaattcttggatttacgaaagacgaaacaactgcgaaagcacttgccaagagtgttttca
>9ce02f3aabada8bc2c31cacb20587842\_3
agctccaatagcgtatattaaagttgttgcagttaaaaagctcgtagttggatttcggagtgggccagttggtccgccgcaaggtgtgttactgactggtctgctcttcttcgcaaagactgcgtgtgctctttgttgagtgtgcgtaggatttacgacgttttactttgaaaaaaattagagtgttcaaagcaggctattgcttgaatacatgagcatggaataatggaataggactttggtcccattttgttggtttctaggaccgaagtaatgattaagagggacaattgggggcatccgtatttcgttgtcagaggtgaaattcttggatttacgaaagacgaacaactgcgaaagcatttgccaagagtgttttca
>a6efa11c6348896def0dcbc1db9c1d84\_3
agctccaatagcgtatattaaagttgttgcagttaaaaaagctcgtagttggatttcggagtgggccagttggtccgccgcaaggtgtgttactgactggtttgctcttcttcgcaaagactgcgtgtgctctttattgagtgtgcgtaggatttacgacgtttactttgaaaaaattagagtgttcaaagcaggctattgcttgaatacatgagcatggaataatggaataggattttggtcccattttgttggtttctaggaccgaagtaatgattaagagggacaattgggggcatccgtatttcgttgttcagaggtgaaattcttggatttacgaaagacgaacaactgcgaaagcacttgccaagagtgttttca
>a6fd0d16c3995b53af37f1197251e657\_3
agctccaatagcgtatattaaagttgttgcagttaaaaaagctcgtagttggatttcggagtgggccagttggtccgccgcaaggtgtgttactgactggtttgctcttcttcgcaaagactgcgtgtgctctttattgagtgtgcgtaggatttacgacgtttactttgaaaaaattagagtgttcaaagcaggctattgcttgaatacatgagcatggaataatggaataggattttggtcccattttgttggtttctaggaccgaagtaatgattaagagggacaattgggggcatccgtatttcgttgtcagaggtgaaattcttggatttacgaaagacgaacaactgcgaaagcacttagccaagagtgttttca
>a886ec07763d81aa223c6a0084440f1b\_3
agctccaatagcgtatattaaagttgttgcagttaaaaagctcgtagttggatttcggagtgggccagttggtccgccgcaaggtgtgttactgactggtttgctcttcttcgcaaagactgcgtgtgctctttattgagtgtgcgtaggatttacgacgtttactttgaaaaaattgagtgttcaaagcaggctattgcttgaatacatgagcatggaataatggaataggattttggtcccattttgttggtttctaggaccgaagtaatgattaagagggacaattgggggcatccgtatttcgttgtcagaggtgaattcttggatttacgaaagacgaacaactgcgaaagcacttgccaagagtgttttca
>aa50d4df1998c9db3902dfc746658bf9\_3
agctccaatagcgtatattaaagttgttgcagttaaaaagctcgtagttggatttcggagtgggcccgttggtccgccgcaaggtgtgttactgactggtctgctcttcttcgcaaagactgcgtgtgctctttgttgagtgtgcgtaggatttacgacgtttactttgaaaaaattagagtgttcaaagcaggctattgcttgaatacatgagcatggaataatggaataggactttggtcccattttgttggtttctaggaccgaagtaatgattaagagggacaattgggggcatccgtatttcgttgtcagaggtgaaatttcttggatttacgaaaagacgaacaaactgcgaaagcacttgccaagagtgttttca
>aa688caf7d2823bbc3090589011c8c29\_3
agctccaatagcgtatattaaagttgttgcagttaaaaagctcgtagttggatttcggagtgggccagttggtccgccgcaaggtgtgttactgactggtctgctcttcttcgcaaaagactgcgtgtgctctttgttgagtgtgcgtaggatttacgacgtttactttgaaaaaattgagtgttcaaagcaggctattgcttgaatacatgagcatggaataatggaataggactttggtcccattttgttggtttctaggaccgaagtaatgattaagagggacaattggggcatccgtatttcgttgtcagaggtgaaattcttggatttacgaaagacgaacaactgcgaaagcatttgccaagagtgttttca
>b084854f3124889c30a1925516e14b90\_3
agctccaatagcgttatattaaagttgttgcagttaaaaagctcgtagttggatttcggagtgggccagttggtccgccgcaaggtgtgttactgactggtctgctcttcttcgcaaagactgcgtgtgctctttgttgagtgtgcgtaggatttacgacgtttactttgaaaaaattagagtgttcaaagcaggctattgcttgaatacatgagcatggaataatggaataggactttggtcccattttgttggtttctaggaccgaagtaatgattaagagggacaattgggggcatccgtatttcgttgtcagaggtgaaattcttggatttacgaaagacgaacaactgcgaaaagcatttgccaagagtgttttca
>b85dab1e80c4f173e767071a57ca67c1\_3
agctccaatagcgtatattaaagttgttgcagttaaaaagctcgtagttggatttcggagtgggcccgttggtccgccgcaaggtgtgttactgactggtctgctcttcttcgcaaagactgcgtgtgctctttgttgagtgtgcgtaggatttacgacgtttactttgaaaaaattagagtgttcaaagcaggctattgcttgaatacatgagcatggaataatggaataggactttggtcccattttgttggtttctaggaccgaagtaatgattaagagggacaattgggggcatccgtatttcgttgtcagaggtgaaattcttggattttacgaaagacgaacaactgcgaaagcacttgccaagagtgttttca
>baf30643b66ccfee15e2c63a92d335a0\_3
agctccaatagcgtatattaaagttgttgcagttaaaaaagactcgtagttggatttcggagtgggccagttggtccgccgcaaggtgtgttactgactggtttgctcttcttcgcaaagactgcgtgtgctctttattgagtgtgcgtaggatttacgacgtttactttgaaaaaattagagtgttcaaagcaggctattgcttgaatacatgagcatggaataatggaataggattttggtcccattttgttggtttctaggaccgaagtaatgattaagagggacaattgggggcatccgtatttcgttgtcagaggtgaaattcttggatttacgaaagacgaacaactgcgaaagcacttgccaagagtgttttca
>bfec600c9967f361b55fb010a90292f6\_3
agctccaatagcgtatattaaagttgttgcagttaaaaagctcgtagttggatttcggagtgggcccgttggtccgccgcaaggtgtgttactgactggtctgctcttcttcgcaaagactgcgtgtgctctttgttgagtgtgcgtaggatttacgacgtttactttgaaaaaattagagtgttcaaagcaggctattgcttgaatacatgagcatggaataatggaataggactttggtcccattttgttggtttctaggaccgaagtaatgattaagagggacaattggggcatccgtatttcgttgtcagaggtgaattcttggatttacgaaagacgaacaactgcgaaagcacttgccaagagtgttttca
>c2e3731c3476dbc9b34931dea6df917f\_3
agctccaatagcgtatattaaagttgttgcagttaaaaagctcgtagttggatttcggagtgggccagttggtccgccgcaaggtgtgttactgactggtttgctcttcttcgcaaagactgcgtgtgctctttattgagtgtgcgtaggatttacgacgtttactttgaaaaaaattagagtgttcaaagcaggctattgcttgaatacatgagcatggaataatggaataggattttggtcccattttgttggtttctaggaccgaagtaatgattaagagggacaattgggggcatccgtattcgttgtcagaggtgaaattcttggatttacgaaagacgaacaactgcgaaagcacttgccaagagtgttttca
>c8f853b22c44c815caf11bb5c943fa17\_3
agctccaatagcgtatattaaagttgttgcagttaaaaaagctcgtagttggatttcggagtgggccagttggtccgccgcaaggtgtgttactgactggtttgctcttcttcgcaaagactgcgtgtgctctttattgagtgtgcgtaggatttacgacgtttactttgaaaaaattagagtgttcaaagcaggctattgcttgaatacatgagcatggaataatggaataggattttggtcccattttgttggtttctaggaccgaagtaatgattaagagggacaattggggcatccgtatttcgttgtcagaggtgaattcttggatttacgaaagacgaacaactgcgaaagcacttgccaagagtgttttca
>cad84c8f2a948db204e07ea7785302fe\_3
agctccaatagcgtatattaaagtgttgcagttaaaaagctcgtagttggatttcggagtgggcccgttggtccgccgcaaggtgtgttactgactggtctgctcttcttcgcaaagactgcgtgtgctctttgttgagtgtgcgtaggatttacgacgtttactttgaaaaaattagagtgttcaaagcaggctattgcttgaatacatgagcatggaataatggaataggactttggtcccattttgttggtttctaggaccgaagtaatgattaagagggacaattgggggcatccgtatttcgttgtcagaggtgaaattcttggatttacgaaagacgaacaactgcgaaagcacttgccaagagtgttttca
>cb95dde0a0ec02402c17491e9ca842ac\_3
agctccaatagcgtatattaaagttgttgcagttaaaaagctcgtagttggatttcggagtgggcccgttggtccgccgcaaggtgtgttactgactggtctgctcttcttcgcaaagactgcgtgtgctctttgttgagtgtgcgtaggatttacgacgtttactttgaaaaaattagagtgttcaaagcaggctattgcttgaatacatgagcatggaataatggaataggactttggtcccattttgttggtttctaggaccgaagtaatgattaagagggacaattgggggcatccgtatttcgttgtcagaggtgaaattcttggatttacgaaagacgaacaactgcgaaagcacttgccaaggatgttttca
>cb9dd3073dd1fd25f181f535072d4f89\_3
agctccaatagcgtatattaaagttgttgcagttaaaaagctcgtagttggatttcggagtgggccagttggtccgccgcaaggtgtgttactgactggtttgctcttcttcgcaaagactgcgtgtgctctttattgagtgtgcgtaggatttacgacgtttactttgaaaaaattagagtgttcaaagcaggctattgcttgaatacatgagcatggaataatggaataggattttggtcccattttgttggtttctaggaccgaagtaatgattaagagggacaattgggggcatccgtatttcgttgtcagaggtgaattcttggatttacgaaagacgaacaactgcgaagcacttgccaagagtgttttca
>ccc14e6e8b1d96e1c6bf36d60a19aa72\_3
agctccaatagcgtatattaaagttgttgtggttaaaagctcgtagttggatttcggagtgggcccgttggtccgccgcaaggtgtgttactgactggtctgctcttcttcgcaaagactgcgtgtgctctttgttgagtgtgcgtaggatttacgacgtttactttgaaaaaattagagtgttcaaagcaggctattgcttgaatacatgagcatggaataatggaataggactttggtcccattttgttggtttctaggaccgaagtaatgattaagagggacaattgggggcatccgtatttcgttgtcagaggtgaaattcttggatttacgaaagacgaacaactgcgaaagcacttgccaagagtgttttca
>d1a037157bb77753b5d9348d09066ffa\_3
agctccaatagcgtatattaaagttgttgcagttaaaaagctcgtagttggatttcggagtgggccagttggtccgccgcaaggtgtgttactgactggtttgctcttcttcgcaaagactgcgtgtgctctttattgagtgtgcgtaggatttacgacgtttactttgaaaaaattagagtgttcaaagcaggctattgcttgaatacatgagcatggaataatggaataggattttggtcccattttgttggtttctaggaccgaagtaatgattaagagggacaattgggggcatccgtatttcgttgtcagaggtgaaattcttggatttacgaaagacgaacaaactgcgaaagcacttgccaagagtgttttca
>d28d1b7da256e86ad5536aa7e30b101c\_3
agctccaatagcgtatattaaagttgttgcagttaaaaagctcgtagttggatttcggagtgggcccgttggtccgccgcaaggtgtgttactgactggtctgctcttcttcgcaaagactgcgtgtgctctttgttgagtgtgcgtaggatttacgacgtttacttgaaaaaattgagtgttcaaagcaggctattgcttgaatacatgagcatggaataatggaataggactttggtcccattttgttggtttctaggaccgaagtaatgattaagagggacaattgggggcatccgtatttcgttgtcagaggtgaaattcttggatttacgaaagacgaacaactgcgaaagcacttgccaagagtgttttca
>d5520727a1d483468cdf90da5d8ea29b\_3
agctccaattagcgtatattaaagttgttgcagttaaaaagctcgtagttggatttcggagtgggcccgttggtccgccgcaaggtgtgttactgactggtctgctcttcttcgcaaagactgcgtgtgctctttgttgagtgtgcgtaggatttacgacgtttactttgaaaaaattagagtgttcaaagcaggctattgcttgaatacatgagcatggaataatggaataggactttggtcccattttgttggtttctaggaccgaagtaatgattaagagggacaattgggggcatccgtatttcgttgtcagaggtgaaattcttggatttacgaaagacgaacaactgcgaaagcacttgccaagagtgttttca
>da2fb0cd67d885b8f056991ea4208c7e\_3
agctccaatagcgtatattaaagttgttgcagttaaaaagctcgtagttggatttcggagtgggcccgttggtccgccgcaaggtgtgttactgactggtctgctcttcttcgcaaagactgcgtgtgctctttgttgagtgtgcgtaggatttacgacgtttacttgaaaaaaattagagtgttcaaagcaggctattgcttgaatacatgagcatggaataatggaataggactttggtcccattttgttggtttctaggaccgaagtaatgattaagagggacaattgggggcatccgtatttcgttgtcagaggtgaaattcttggatttacgaaagacgaacaactgcgaaagcacttgccaagagtgtttca
>db550a22e3e8146d380b3e24eb4f5f34\_3
agctccaatagcgtatattaaagttgttgcagttaaaaagctcgtagttggatttcggagtgggcccgttggtccgccgcaaggtgtgttactgactggtctgctcttcttcgcaaaagactgcgtgtgctctttgttgagtgtgcgtaggatttacgacgtttactttgaaaaaaattagagtgttcaaagcaggctattgcttgaatacatgagcatggaataatggaataggactttggtcccattttgttggtttctaggaccgaagtaatgattaagagggacaattgggggcatccgtatttcgttgtcagaggtgaaatttcttggatttacgaaagacgaacaactgcgaaagcacttgccaagagtgttttca
>dbd47e89c98c238829cab13856efa35f\_3
agctccaatagcgtatattaaagttgttgcagttaaaaaagctcgtagttggatttcggagtgggccagttggtccgccgcaaggtgtgttactgactggtttgctcttcttcgcaaagactgcgtgtgctctttattgagtgtgcgtaggatttacgacgtttactttgaaaaaattagagtgttcaaagcaggctattgcttgaatacatgagcatggaataatggaataggattttggtcccattttgttggtttctaggaccgaagtaatgattaagagggacaattgggggcgatccgtatttcgttgtcagaggtgaaattcttggatttacgaaagacgaacaactgcgaaagcacttgccaagagtgttttca
>ddd3b9be5d156ac8ec97547d2eac546a\_3
agctccaatagcgtatattaaagttgttgcagttaaaagctcgtagttggatttcggagtgggccagttggtccgccgcaaggtgtgttactgactggtctgctcttcttcgcaaagactgcgtgtgctctttgttgagtgtgcgtaggatttacgacgtttactttgaaaaattagagtgttcaaagcaggctattgcttgaatacatgagcatggaataatggaataggactttggtcccattttgttggtttctaggaccgaagtaatgattaagagggacaattgggggcatccgtatttcgttgtcagaggtgaaattcttggatttacgaaagacgaacaactgcgaaagcatttgccaagagtgttttca
>df351013f9771c1d27c88569e5bd3eb3\_3
agctccaatagcgtatattaaagttgttgcagttaaaaagctcgtagttggatttcggagtgggccagttggtccgccgcaaggtgtgttactgactggtctgctcttcttcgcaaaagactgcgtgtgctctttgttgagtgtgcgtaggatttacgacgtttactttgaaaaaattgagtgttcaaagcaggctattgcttgaatacatgagcatggaataatggaataggactttggtcccattttgttggtttctaggaccgaagtaatgattaagagggacaattgggggcatccgtatttcgttgtcagaggtgaaattcttggatttacgaaagacgaacaactgcgaaagcatttgccaagagtgttttca
>e06949c829456274b3354441ef8ce4ae\_3
agctccaatagcgtatattaaagttgttgcagttaaaaagctcgtagttggatttcggagtgggcccgttggtccgccgcaaggtgtgttactgactggtctgctcttcttcgcaaagactgcgtgtgctctttgttgagtgtgcgtaggatttacgacgtttactttgaaaaaaattagagtgttcaaagcaggctattgcttgaatacatgagcatggaataatggaataggactttggtcccattttgttggtttctaggaccgaagtaatgattaagagggacaattggggcatccgtatttcgttgtcagaggtgaaattcttggatttacgaaagacgaacaactgcgaaagcacttgccaagagtgttttca
>e30152169005619f496c7065e7572e3d\_3
agctccaatagcgtatattaaagttgttgcagttaaaaaagctcgtagttggatttcggagtgggccagttggtccgccgcaaggtgtgttactgactggtctgctcttcttcgcaaagactgcgtgtgctctttgttgagtgtgcgtaggatttacgacgtttactttgaaaaaattagagtgttcaaagcaggctattgcttgaatacatgagcatggaataatggaataggactttggtcccattttgttggtttctaggaccgaagtaatgattaagagggacaattgggggcatccgtatttcgttgtcagaggtgaaattcttggatttacgaaagacgaacaactgcgaaaagcatttgccaagagtgttttca
>f031cb492516c87c94af40d4e7764e72\_3
agctccaatagcgtatattaaagttgttgcagttaaaaagctcgtagttggatttcggagtgggccagttggtccgccgcaaggtgtgttactgactggtctgctcttcttcgcaaagactgcgtgtgctctttgttgagtgtgcgtaggatttacgacgtttactttgaaaaaattagagtgttcaaagcaggctattgcttgaatacatgagcatggaataatggaataggactttggtcccattttgttggtttctaggaccgaagtaatgattaagagggacaattgggggcatccgtatttcgttgtcagagtgaaattcttggatttacgaaagacgaacaactgcgaaagcatttgccaagagtgttttca
>f0b72c2f1459538744729d3ebddc03f6\_3
agctccaatagcgtatattaaagttgttgcagttaaaaagctcgtagttggatttcggagtgggccagttggtccgccgcaaggtgtgttactgactggtctgctcttcttcgcaaagactgcgtgtgctctttgttgagtgtgcgtaggatttacgacgtttactttgaaaaaattagagtgttcaaagcaggctattgcttgaatacatgagcatggaataatggaataggactttggtcccattttgttggtttctaggaccgaagtaatgattaagagggacaattggggcatccgtatttcgtgtcagaggtgaaattcttggatttacgaaagacgaacaactgcgaaagcatttgccaagagtgttttca
>f263974717b88867db53802168fbad07\_3
agctccaatagcgtatattaaagttgttgcagttaaaaagctcgtagttggatttcggagtgggccagttggtccgccgcaaggtgtgttactgactggtctgctcttcttcgcaaagactgcgtgtgctctttgttgagtgtgcgtaggatttacgacgtttactttgaaaaaattgagtgttcaaagcaggctattgcttgaatacatgagcatggaataatggaataggactttggtcccattttgttggtttctaggaccgaagtaatgattaagagggacaattgggggcatccgtatttcgttgtcagaggtgaaattcttggatttacgaaagacgaacaactgcgaaagcatttgccaagagtgtttca
>ff4746cb90782f73320771ef3aa6a03e\_3
agctccaatagcgtatattaaagttgttgcagttaaaaagctcgtagttggatttcggagtgggccagttggtccgccgcaaggtgtgttactgactggtttgctcttcttcgcaaagactgcgtgtgctctttattgagtgtgcgtaggatttacgacgtttactttgaaaaaattagagtgttcaaagcaggctattgcttgaatacatgagcatggaataatggaataggattttggtcccatttttgttggtttctaggaccgaagtaatgattaagagggacaattgggggcatccgtatttcgtgtcagaggtgaaattcttggatttacgaaagacgaacaactgcgaaagcacttgccaagagtgttttca
>ffe3f3e15716c245ca6bd105051bcb6f\_3
agctccaatagcgtatattaaaagttgttgcagttaaaaagctcgtagttggatttcggagtgggccagttggtccgccgcaaggtgtgttactgactggtctgctcttcttcgcaaagactgcgtgtgctctttgttgagtgtgcgtaggatttacgacgtttactttgaaaaaattagagtgttcaaagcaggctattgcttgaatacatgagcatggaataatggaataggactttggtcccattttgttggtttctaggaccgaagtaatgattaagagggacaattgggggcatccgtatttcgttgtcagaggtgaaattcttggatttacgaaagacgaacaactgcgaaaagcatttgccaagagtgttttca
>007de976a54ed3caccd3e27e4ed7d960\_2
agctccaatagcgtatactaaagttgttgcagttaaaaagctcgtagttggatttcggagtgggcccgttggtccgccgcaaggtgtgttactgactggtctgctcttcttcgcaaagactgcgtgtgctctttgttgagtgtgcgtaggatttacgacgtttactttgaaaaaattagagtgttcaaagcaggctattgcttgaatacatgagcatggaataatggaataggactttggtcccattttgttggtttctaggaccgaagtaatgattaagagggacaattgggggcatccgtatttcgtgtcagaggtgaaattcttggatttacgaaagacgaacaactgcgaaagcacttgccaagagtgttttca
>00f8ecd75b3a0734bb81721107ebf54b\_2
agctccaatagcgtatattaaagttgttgcagttaaaaaagctcgtagttggatttcggagtgggccagttggtccgccgcaaggtgtgttactgactggtctgctcttcttcgcaaagactgcgtgtgctctttgttgagtgtgcgtaggatttacgacgtttactttgaaaaaattgagtgttcaaagcaggctattgcttgaatacatgagcatggaataatggaataggactttggtcccattttgttggtttctaggaccgaagtaatgattaagagggacaattgggggcatccgtatttcgttgtcagaggtgaaattcttggatttacgaaagacgaacaactgcgaaagcatttgccaagagtgttttca
>025201d4c475731e1c8f857603f50219\_2
agctccaatagcgtatattaaagttgttgcagttaaaaagctcgtagttggatttcggagtgggccagttggtccgccgcaatgtgtgttactgactggtttgctcttcttcgcaaagactgcgtgtgctctttattgagtgtgcgtaggatttacgacgtttactttgaaaaaattagagtgttcaaagcaggctattgcttgaatacatgagcatggaataatggaataggattttggtcccattttgttggtttctaggaccgaagtaatgattaagagggacaattgggggcatccgtatttcgttgtcagaggtgaaattcttggatttacgaaagacgaacaactgcgaaagcacttgccaagagtgttttca
>05ec0262cad30911458438fbc61a07f0\_2
agctccaatagcgtatattaaagttgttgcagttaaaaaagctcgtagttggatttcggagtgggccagttggtccgccgcaaggtgtgttactgactggtttgctcttcttcgcaaagactgcgtgtgctctttattgagtgtgcgtaggatttacgacgtttactttgaaaaaattagagtgttcaaagcaggctattgcttgaatacatgagcatggaataatggaataggatttggtccattttgttggtttctaggaccgaagtaatgattaagagggacaattgggggcatccgtatttcgttgtcagaggtgaaattcttggatttacgaaagacgaacaactgcgaaagcacttgccaagagtgttttca
>05ef166e5f398f96bbffbc97e25e3d06\_2
agctccaatagcgtatattaaagttgttgcagttaaaagctcgtagttggatttcggagtgggccagttggtccgccgcaaggtgtgttactgactggtctgctcttcttcgcaaagactgcgtgtgctctttgttgagtgtgcgtaggatttacgacgtttactttgaaaaaattagagtgttcaaagcaggctattgcttgaatacatgagcatggaataatggaataggactttggtcccattttgttggtttctaggaccgaagtaatgattaagagggacaattgggggcatccgtatttcgttgtcagaggtgaaattcttggatttacgaaagacgaacaactgcgaaaagcatttgccaagagtgttttca
>065e1d5e3ccae85754896763f5b55e82\_2
agctccaatagcgtatattaaagttgttgcagttaaaaagctcgtagttggatttcggagtgggccagttggtccgccgcaaggtgtgttactgactggtctgctcttcttcgcaaagactgcgtgtgctctttgttgagtgtgcgtaggatttacgacgtttactttgaaaaaattagagtgttcaaagcaggctattgcttgaatacatgagcatggaataatggaataggactttggtcccattttgttggtttctaggaccgaagtaatgattaagagggacaattgggggcatccgtatttcgttgttcagaggtgaaattcttggatttacgaaagacgaacaactgcgaaagcatttgccaagagtgttttca
>069882666538b0e4fb4c259c15e916a5\_2
agctccaatagcgtatattaaagttgttgcagttaaaaagctcgtagttggatttcggagtgggccagttggtccgccgcaaggtgtgttactgactggtctgctcttcttcgcaaagactgcgtgtgctctttgttgagtgtgcgtaggatttacgacgtttactttgaaaaaattagagtgttcaaagcaggctattgcttgaatacatgagcatggaataatggaataggactttggtcccattttgttggtttctaggaccgaagtaatgattaagagggacaattggggcatccgtatttcgttgtcagaggtgaaattcttggatttacgaaagacgaacaactgcgaaaagcatttgccaagagtgtttttca
>06c788cbb3bf23538d73456c3f806764\_2
agctccaatagcgtatattaaagttgttgcagttaaaaaagctcgtagttggatttcggagtgggccagttggtccgccgcaaggtgtgttactgactggtttgctcttcttcgcaaagactgcgtgtgctctttattgagtgtgcgtaggatttacgacgtttactttgaaaaaattagagtgttcaaagcaggctattgcttgaatacatgagcatggaataatggaataggatttggtcccattttgttggtttctagaccgaagtaatgattaagagggacaattgggggcatccgtatttcgttgtcagaggtgaaattcttggatttacgaaagacgaacaactgcgaaagcacttgccaagagtgttttca
>0824fdfd884001edd0ae1599d55b2c60\_2
agctccaatagcgtatattaaagttgttgcagttaaaaagctcgtagttggatttcggagtgggccagttggtccgccgcaaggtgtgttactgactggtctgctcttcttcgcaaagactgcgtgtgctctttgttgagtgtgcgtaggatttacgacgtttactttgaaaaaattagagtgttcaaagcaggctattgcttgaatacatgagcatggaataatggaataggactttggtcccattttgttggtttctaggaccgaagtaatgattaagagggacaattgggggcatccgtatttcgttgtcagaggtgaaattcttggatttacgaaagacgaacaactgcgaaaagcatttgccaagagtgttttcta
>083c2988ee05abc056ebeb6a43a17083\_2
agctccaatagcgtatattaaagttgttgcagttaaaaagctcgtagttggatttcggagtgggccagttggtccgccgcaaggtgtgttactgactggtttgctcttcttcgcaaagactgcgtgtgctctttattgagtgtgcgtaggatttacgacgtttactttgaaaaaattagagtgttcaaagcaggctattgcttgaatacatgagcatggaataatggaataggattttggtcccattttgttggtttctaggaccgaagtaatgattaagagggacaattgggggcatccgtatttcgtgtcagaggtgaaattcttggatttacgaaagacgaacaactgcgaaagcacttgccaagagtgtttttca
>08a54a4101793f2b49bf6f993991b227\_2
agctccaatagcgtatattaaagttgttgcagttaaaaagctcgtagttggatttcggagtgggccagttggtccgccgcaaggtgtgttactgactggtctgctcttcttcgcaaagactgcgtgtgctctttgttgagtgtgcgtaggatttacgacgtttactttgaaaaaattagagtgttcaaagcaggctattgcttgaatacatgagcatggaataatggaataggactttggtcccattttgttggtttctaggaccgaagtaatgattaagagggacaattggggcatccgtatttcgttgtcagaggtgaaattcttggatttacgaaagacgaacaactgcgaaaagcacttgccaagagtgttttca
>08c2014d14244767e51b43bc5fa4c659\_2
agctccaatagcgtatattaaagttgttgcagttaaaaaagctcgtagttggatttcggagtgggcccgttggtccgccgcaaggtgtgttactgactggtctgctcttcttcgcaaaagactgcgtgtgctctttgttgagtgtgcgtaggatttacgacgtttactttgaaaaaattgagtgttcaaagcaggctattgcttgaatacatgagcatggaataatggaataggactttggtcccattttgttggtttctaggaccgaagtaatgattaagagggacaattgggggcatccgtatttcgttgtcagaggtgaaatttcttggatttacgaaagacgaacaactgcgaaagcacttgccaagagtgttttca
>090a4b862d6e529c90b9ef75430c3422\_2
agctccaatagcgtatattaaagttgttgcagttaaaaaagctcgtagttggatttcggagtgggcccgttggtccgccgcaaggtgtgttactgactggtctgctcttcttcgcaaagactgcgtgtgctctttgttgagtgtgcgtaggatttacgacgtttactttgaaaaaattagagtgttcaaagcaggctattgcttgaatacatgagcatggaataatggaataggactttggtcccattttgttggtttctaggaccgaagtaatgattaagagggacaattgggggcatccgtatttcgttgtcagagggtgaaattcttggatttacgaaagacgaacaactgcgaaagcacttgccaagagtgttttca
>09173d89b323db8bb032a89f7f25932b\_2
agctccaatagcgtatattaaagttgttgcagttaaaaagctcgtagttggatttcggagtgggccagttggtccgccgcaaggtgtgttactgactggtttgctcttcttcgcaaagactgcgtgtgctctttattgagtgtgcgtaggatttacgacgtttactttagaaaaaattagagtgttcaaagcaggctattgcttgaatacatgagcatggaataatggaataggattttggtcccattttgttggtttctaggaccgaagtaatgattaagagggacaattgggggcatccgtatttcgttgtcagaggtgaaattcttggatttacgaaagacgaacaactgcgaaagcacttgccaagagtgttttca
>095cb3f9184d523b64f02ed5f634403f\_2
agctccaatagcgtatattaaagttgttgtcagttaaaaagctcgtagttggatttcggagtgggccagttggtccgccgcaaggtgtgttactgactggtctgctcttcttcgcaaagactgcgtgtgctctttgttgagtgtgcgtaggatttacgacgtttactttgaaaaaattagagtgttcaaagcaggctattgcttgaatacatgagcatggaataatggaataggactttggtcccattttgttggtttctaggaccgaagtaatgattaagagggacaattgggggcatccgtatttcgttgtcagaggtgaaattcttggatttacgaaagacgaacaactgcgaaagcatttgccaagagtgttttca
>0afd0dd05905005bba6ca92fa05bbdad\_2
agctccaatagcgtatattaaagttgttgcagttaaaaagctcgtagttggatttcggagtgggccagttggtccgccgcaaggtgtgttactgactggtctgctcttcttcgcaaagactgcgtgtgctctttgttgagtgtgcgtaggatttacgacgtttactttgaaaaaaattagagtgttcaaagcaggctattgcttgaatacatgagcatggaataatggaataggactttggtcccattttgttggtttctaggaccgaagtaatgattaagagggacaattgggggcatccgtattcgttgtcagaggtgaaattcttggatttacgaaagacgaacaactgcgaaagcatttgccaagagtgttttca
>0b46e337d876398894998f9d09931a67\_2
agctccaatagcgtatattaaagttgttgcagttaaaaagctcgtagttggatttcggagtgggccagttggtccgccgcaaggtgtgttactgactggtctgctcttcttcgcaaaagactgcgtgtgctctttgttgagtgtgcgtaggatttacgacgtttactttgaaaaaaattagagtgttcaaagcaggctattgcttgaatacatgagcatggaataatggaataggactttggtcccattttgttggtttctaggaccgaagtaatgattaagagggacaattgggggcatccgtatttcgttgtcagaggtgaaattcttggatttacgaaagacgaacaactgcgaaagcatttgccaagagtgttttca
>0c74f820fc75bc145e8e1fbb085f5afb\_2
agctccaatagcgtatattaaagttgttgcagttaaaaagctcgtagttggatttcggatgggccagttggtccgccgcaaggtgtgttactgactggtctgctcttcttcgcaaagactgcgtgtgctctttgttgagtgtgcgtaggatttacgacgtttactttgaaaaaattagagtgttcaaagcaggctattgcttgaatacatgagcatggaataatggaataggactttggtcccattttgttggtttctaggaccgaagtaatgattaagagggacaattgggggcatccgtatttcgttgtcagaggtgaaattcttggatttacgaaagacgaacaactgcgaaagcatttgccaagagtgttttca
>0e593893c7e5e1685ef54074e9dc7ced\_2
agctccaatagcgtatattaaagttgttgcagttaaaaaagctcgtagttggatttcggagtgggccagttggtccgccgcaaggtgtgttactgactggtttgctcttcttcgcaaagactgcgtgtgctctttattgagtgtgcgtaggatttacgacgtttactttgaaaaaattagagtgttcaaagcaggctattgcttgaatacatgagcatggaataatggaataggattttggtcccattttgttggtttctaggaccgaagtaatgattaagagggacaattgggggcatccgtatttcgttgtcagaggtgaaattcttggatttacgaagacgaacaactgcgaaagcacttgccaagagtgttttca
>0f530f38eecf1c1727761c2fdee99c09\_2
agctccaatagcgtatattaaagttgttgcagttaaaaagctcgtagttggatttcggagtgggccagttggtccgccgcaaggtgtgttactgactggtctgctcttcttcgcaaagactgcgtgtgctctttgttgagtgtgcgtaggatttacgacgtttactttgaaaaaattagagtgttcaaagcaggctattgcttgaatacatgagcatggaataatggaataggactttggtcccattttgttggtttctaggaccgaagtaatgattaagagggacaattgggggcatccgtatttcgttgttcagaggtgaaatttcttggatttacgaaagacgaacaactgcgaaagcatttgccaagagtgttttca
>0f556eaf95c3c83563190e7e4f58851b\_2
agctccaatagcgtatattaaagttgttgcagttaaaaagctcgtagttggatttcggagtgggcccgttggtccgccgcaaggtgtgttactgactggtctgctcttcttcgcaaaagactgcgtgtgctctttgttgagtgtgcgtaggatttacgacgtttactttgaaaaaattagagtgttcaaagcaggctattgcttgaatacatgagcatggaataatggaataggactttggtcccattttgttggtttctaggaccgaagtaatgattaagagggacaattgggggcatccgtatttcgttgtcagaggtgaaattctttggatttacgaaagacgaacaactgcgaaagcacttgccaagagtgttttca
>10881b30427c9302ba05454adad0c1da\_2
agctccaatagcgtatattaaagttgttgcagttaaaaagctcgtagttggatttcggagtgggccagttggtccgccgcaaggtgtgttactgactggtttgctcttcttcgcaaagactgcgtgtgctctttgttgagtgtgcgtaggatttacgacgtttactttgaaaaaattagagtgttcaaagcaggctattgcttgaatacatgagcatggaataatggaataggactttggtcccattttgttggtttctaggaccgaagtaatgattaagagggacaattgggggcatccgtatttcgttgtcagaggtgaaattcttggatttacgaaaagacgaacaactgcgaaagcacttgccaagagtgttttca
>128ebcca345b76c11eb54fe8c4237ed9\_2
agctccaatagcgtatattaaagttgttgcagttaaaaagctcgtagttggatttcggagtgggccagttggtccgccgcaaggtgtgttactgactggtctgctcttcttcgcaaaagactgcgtgtgctctttgttgagtgtgcgtaggatttacgacgtttactttgaaaaaaattagagtgttcaaagcaggctattgcttgaatacatgagcatggaataatggaataggactttggtcccattttgttggtttctaggaccgaagtaatgattaagagggacaattgggggcatccgtatttcgttgtcagaggtgaaatttcttggatttacgaaagacgaacaactgcgaaagcatttgccaagagtgttttca
>1cb2968971fad14a651a84d5953dff64\_2
agctccaatagcgtatattaaagttgttgacagttaaaaagctcgtagttggatttcggagtgggccagttggtccgccgcaaggtgtgttactgactggtctgctcttcttcgcaaagactgcgtgtgctctttgttgagtgtgcgtaggatttacgacgtttactttgaaaaaattagagtgttcaaagcaggctattgcttgaatacatgagcatggaataatggaataggactttggtcccatttttgttggtttctaggaccgaagtaatgattaagagggacaattgggggcatccgtatttcgttgtcagaggtgaaattcttggatttacgaaagacgaacaactgcgaaagcatttgccaagagtgttttca
>1e05aeea18ddd8aeefc4c31b673327fe\_2
agctccaatagcgtatattaaagttgttgcagttaaaaagctcgtagttggatttcggagtgggccagttggtccgccgcaaggtgtgttactgactggtttgctcttcttcgcaaagactgcgtgtgctctttattgagtgtgcgtaggatttacgacgtttactttgaaaaaattagagtgttcaaagcaggctattgcttgaatacatgagcatggaataatggaataggattttggtcccattttgttggtttctaggaccgaagtaatgattaagagggacaattgggggcatccgtatttcgttgtcagaggtgaaattcttggatttacgaaagcgaacaactgcgaaagcacttgccaagagtgttttca
>1e300c18c25784591f1cf2ca47812c8f\_2
agctccaatagcgtatattaaagttgttgcagttaaaaagctcgtagttggatttcggagtgggccagttggtccgccgcaaggtgtgttactgactggtctgctcttcttcgcaaagactgcgtgtgctctttgttgagtgtgcgtaggatttacgacgtttactttgaaaaaattagagtgttcaaagcaggctattgcttgaatacatgagcatggaataatggaataggactttggtcccattttgttggtttcttaggaccgaagtaatgattaagagggacaattgggggcatccgtatttcgttgtcagaggtgaaattcttggatttacgaaagacgaacaactgcgaaagcatttgccaagagtgttttca
>1e4c4bc5c8acd3b225c5421d682faaf6\_2
agctccaatagcgtattattaaagttgttgcagttaaaagctcgtagttggatttcggagtgggcccgttggtccgccgcaaggtgtgttactgactggtctgctcttcttcgcaaagactgcgtgtgctctttgttgagtgtgcgtaggatttacgacgtttactttgaaaaaattagagtgttcaaagcaggctattgcttgaatacatgagcatggaataatggaataggactttggtcccattttgttggtttctaggaccgaagtaatgattaagagggacaattgggggcatccgtatttcgttgtcagaggtgaaattcttggatttacgaaagacgaacaactgcgaaagcacttgccaagagtgttttca
>1e6e622b9b772965f2f4f8e93b2a8b9d\_2
agctccaatagcgtatattaaagttgttgcagttaaaagctcgtagttggatttcggagtgggccagttggtccgccgcaaggtgtgttactgactggtttgctcttcttcgcaaagactgcgtgtgctctttattgagtgtgcgtaggatttacgacgtttactttgaaaaaattagagtgttcaaagcaggctattgcttgaatacatgagcatggaataatggaataggattttggtcccattttgttggtttctaggaccgaagtaatgattaagagggacaattgggggcatccgtatttcgttgtcagaggtgaaattcttggatttacgaaagacgaacaactgcgaaaagcacttgccaagagtgttttca
>1eff9403e10765e6c854fc3433c2e0f9\_2
agctccaatagcgtatattaaagttgttgcagttaaaaaagctcgtagttggatttcggagtgggccagttggtccgccgcaaggtgtgttactgactggtttgctcttcttcgcaaagactgcgtgtgctctttattgagtgtgcgtaggatttacgacgtttactttgaaaaaaattgagtgttcaaagcaggctattgcttgaatacatgagcatggaataatggaataggattttggtcccattttgttggtttctaggaccgaagtaatgattaagagggacaattgggggcatccgtatttcgttgtcagaggtgaaattcttggatttacgaaagacgaacaactgcgaaagcacttgccaagagtgttttca
>1f114076e84b21fba2d23a43b188bfdf\_2
agctccaatagcgtatattaaagttgttgcagttaaaaagctcgtagttggatttcggagtgggccagttggtccgccgcaaggtgtgttactgactggtttgctcttcttcgcaaagactgcgtgtgctctttattgagtgtgcgtaggatttacgacgtttactttgaaaaaattgagtgttcaaagcaggctattgcttgaatacatgagcatggaataatggaataggattttggtcccattttgttggtttctaggaccgaagtaatgattaagagggacaattgggggcatccgtatttcgttgtctagaggtgaaattcttggatttacgaaagacgaacaactgcgaaagcacttgccaagagtgttttca
>1f9d371775d6d6c3ba803b49e643e87d\_2
agctccaatagcgtatattaaagttgttgcagttaaaaagctcgtagttggatttcggagtgggccagttggtccgccgcaaggtgtgttactgactggtttgctcttcttcgcaaagactgcgtgtgctctttattgagtgtgcgtaggatttacgacgtttactttgaaaaaattagagtgttcaaagcaggctattgcttgaatacatgagcatggaataatggaataggattttggtcccattttgttggtttctaggaccgaagtaatgattaagagggacaattgggggcatccgtatttcgttgtcagaggtgaaattcttggatttacgaaagacgaacaactgcgaagcacttgccaagagtgtttca
>22b61b7b4d146473be629349aa70bfcd\_2
agctccaatagcgtatattaaagttgttgcagttaaaaaagctcgtagttggatttcggagtgggcccgttggtccgccgcaaggtgtgttactgactggtctgctcttcttcgcaaagactgcgtgtgctctttgttgagtgtgcgtaggatttacgacgtttactttgaaaaaaattagagtgttcaaagcaggctattgcttgaatacatgagcatggaataatggaataggactttggtcccattttgttggtttctaggaccgaagtaatgattaagagggacaattgggggcatccgtatttcgttgtcagaggtgaaatttcttggatttacgaaagacgaacaactgcgaaagcacttgccaagagtgttttca
>26cb7edbfb153b5f15fc8c0e65c3572b\_2
ggctccaatagcgtatattaaagttgttgcagttaaaaagctcgtagttggatttcggagtgggccagttggtccgccgcaaggtgtgttactgactggtttgctcttcttcgcaaagactgcgtgtgctctttattgagtgtgcgtaggatttacgacgtttactttgaaaaaattagagtgttcaaagcaggctattgcttgaatacatgagcatggaataatggaataggattttggtcccattttgttggtttctaggaccgaagtaatgattaagagggacaattgggggcatccgtatttcgttgtcagaggtgaaattcttggatttacgaaagacgaacaactgcgaaagcacttgccaagagtgttttca
>27cbc7f65a764e6a0fb45be60b9baa85\_2
agctccaatagcgtatattaaagttgttgcagttaaaaagctcgtagttggatttcggagtgggccagttggtccgccgcaaggtgtgttactgactggtttgctcttcttcgcaaagactgcgtgtgctctttgttgagtgtgcgtaggatttacgacgtttactttggaaaaaattagagtgttcaaagcaggctattgcttgaatacatgagcatggaataatggaataggactttggtcccattttgttggtttctaggaccgaagtaatgattaagagggacaattgggggcatccgtatttcgttgtcagaggtgaaattcttggatttacgaaagacgaacaactgcgaaagcacttgccaagagtgttttca
>292bffec3c2c3955c7c3f108258743b8\_2
agctccaatagcgtatattaaagttgttgcagttaaaaagctcgtagttggatttcggagtgggccagttggtccgccgcaaggtgtgttactgactggtctgctcttcttcgcaaagactgcgtgtgctctttgttgagtgtgcgtaggatttacgacgtttactttgaaaaaattagagtgttcaaagcaggctattgcttgaatacatgagcatggaataatggaataggactttggtcccatttgttggtttctaggaccgaagtaatgattaagagggacaattgggggcatccgtatttcgttgtcagaggtgaaattcttggatttacgaaagacgaacaactgcgaaagcatttgccaagagtgttttca
>295e2d9ab12e841a343aadf532be0efa\_2
agctccaatagcgtatattaaagttgttgcagttaaaaagctcgtagttggatttcggagtgggcccgttggtccgccgcaaggtgtgttactgactggtctgctcttcttcgcaaaagactgcgtgtgctctttgttgagtgtgcgtaggatttacgacgtttactttgaaaaaattagagtgttcaaagcaggctattgcttgaatacatgagcatggaataatggaataggactttggtcccattttgttggtttctaggaccgaagtaatgattaagagggacaattgggggcatccgtatttcgttgtcaggaggtgaaatttcttggatttacgaaaagacgaacaactgcgaaagcacttgccaagagtgttttca
>2ad656092e0cad0a1f0e5bf62b6b8cc8\_2
agctccaatagcgtatattaaagttgttgcagttaaaaagctcgtagttggatttcggagtgggcccgttggtccgccgcaaggtgtgttactgactggtctgctcttcttcgcaaagactgcgtgtgctctttgttgagtgtgcgtaggatttacgacgtttactttgaaaaaaattagagtgttcaaagcaggctattgcttgaatacatgagcatggaataatggaataggactttggtcccattttgttggtttctagggccgaagtaatgattaagagggacaattgggggcatccgtatttcgttgtcagaggtgaaattcttggatttacgaaagacgaacaactgcgaaagcacttgccaagagtgttttca
>2ccd8075ca3c38362be301ed8d149bd0\_2
agctccaatagcgtatattaaagttgttgcagttaaaaagctcgtagttggatttcggagtgggccagttggtccgccgcaaggtgtgttactggactggtttgctcttcttcgcaaagactgcgtgtgctctttattgagtgtgcgtaggatttacgacgtttactttgaaaaaattagagtgttcaaagcaggctattgcttgaatacatgagcatggaataatggaataggattttggtcccattttgttggtttctaggaccgaagtaatgattaagagggacaattgggggcatccgtatttcgttgtcagaggtgaaattcttggatttacgaaagacgaacaactgcgaaagcacttgccaagagtgtttca
>2d852d6fbc7461611967b14c69417444\_2
agctccaatagcgtatattaaagttgttgcagttaaaaagctcgtagttggatttcggagtgggccagttggtccgccgcaaggtgtgttactgactggtttgctcttcttcgcaaagactgcgtgtgctctttattgagtgtgcgtaggatttacgacgtttactttgaaaaaattagagtgttcaaagcaggctattgcttgaatacatgagcatggaataatggaataggattttggtcccattttgttggtttctaggaccgaagtaatgattaagagggacaattgggggcatccgtatttcgttgtcagaggtgaaattcttggatttacgaagacgaacaactgcgaaagcacttgccaagagtgttttca
>2f8a1426e59410bc56aa9c19be92065a\_2
agctccaatagcgtatattaaagttgttgcagttaaaaagctcgtagttggatttcggagtgggccagttggtccgccgcaaggtgtgttactgactggtttgctcttcttcgcaaagactgcgtgtgctctttattgagtgtgcgtaggatttacgacgtttactttgaaaaaaattagagtgttcaaagcaggctattgcttgaatacatgagcatggaataatggaataggattttggtcccattttgttggtttctaggaccgaagtaatgattaagagggacaattggggcatccgtatttcgttgtcagaggtgaaattcttggatttacgaaagacgaacaactgcgaaagcacttgccaagagtgtttca
>302e1a36542419bd0a4fb25dcaace222\_2
agctccaatagcgtatattaaagttgttgcagttaaaaagctcgtagttggatttcggagtgggccagttggtccgccgcaaggtgtgttactgactggtctgctcttcttcgcaaagactgcgtgtgctctttgttgagtgtgcgtaggatttacgacgtttactttgaaaaaattgagtgttcaaagcaggctattgcttgaatacatgagcatggaataatggaataggactttggtcccatttttgttggtttctaggaccgaagtaatgattaagagggacaattgggggcatccgtatttcgttgtcagaggtgaaattcttggatttacgaaagacgaacaactgcgaaagcatttgccaagagtgttttca
>309c29c50e6238c684781b3350baa558\_2
agctccaatagcgtatattaaagttgttgcagttaaaaagctcgtagttggatttcggagtgggcccgttggtccgccgcaaggtgtgttactgactggtctgctcttcttcgcaaagactgcgtgtgctctttgttgagtgtgcgtaggatttacgacgtttactttgaaaaaattagagtgttcaaagcaggctattgcttgaatacatgagcatggaataatggaataggactttggtcccattttgttggtttctaggtaccgaagtaatgattaagagggacaattgggggcatccgtatttcgttgtcagaggtgaaattcttggatttacgaaagacgaacaactgcgaaagcacttgccaagagtgttttca
>35bac5ce1b0ad595c4e930c921089184\_2
agctccaatagcgtatattaaagttgttgcggttaaaaagctcgtagttggatttcggagtgggcccgttggtccgccgcaaggtgtgttactgactggtctgctcttcttcgcaaagactgcgtgtgctctttgttgagtgtgcgtaggatttacgacgtttactttgaaaaaattagagtgttcaaagcaggctattgcttgaatacatgagcatggaataatggaataggactttgatcccattttgttggtttctaggaccgaagtaatgattaagagggacaattgggggcatccgtatttcgttgtcagaggtgaaattcttggatttacgaaagacgaacaactgcgaaagcacttgccaagagtgttttca
>367a1507f9b4463abb8fe7fc273ffad9\_2
agctccaatagcgtatattaaagttgttgcagttaaaaagctcgtagttggatttcggagtgggccagttggtccgccgcaaggtgtgttactgactggtttgctcttcttcgcaaagactgcgtgtgctctttattgagtgtgcgtaggatttacgacgtttactttgaaaaaattagagtgttcaaagcaggctattgcttgaatacatgagcatggaataatggaataggattttggtcccattttgttggtttctaggaccgaagtaatgattaagagggacaattgggggcagtccgtatttcgttgtcagaggtgaaattcttggatttacgaaagacgaacaactgcgaaagcacttgccaagagtgttttca
>3836e8f09935f50345a72f49b680bd9a\_2
agctccaatagcgtatattaaagttgttgcagttaaaaaagctcgtagttggatttcggagtgggccagttggtccgccgcaaggtgtgttactgactggtttgctcttcttcgcaaagactgcgtgtgctctttattgagtgtgcgtaggatttacgacgtttactttgaaaaaattagagtgttcaaagcaggctattgcttgaatacatgagcatggaataatggaataggattttggtcccattttgttggtttctaggaccgaagtaatgattaagagggacaattgggggcatccgtatttcgttgtcagagtgaaattcttggatttacgaaagacgaacaactgcgaaagcacttgccaagagtgttttca
>38c7d9e3ec6eebdaab0d4190bf0d61b2\_2
agctccaatagcgtatattaaagttgttgcagttaaaaagctcgtagttggatttcggagtgggcccgttggtccgccgcaaggtgtgttactgactggtctgctcttcttcgcaaagactgcgtgtgctctttgttgagtgtgcgtaggatttacgacgtttactttgaaaaaattagagtgttcaaagcaggctattgcttgaatacatgagcatggaataatggaataggactttggtcccattttgttggtttctaggaccgaagtaatgattaagagggacaattgggggcgatccgtatttcgttgtcagaggtgaaattcttggatttacgaaagacgaacaactgcgaaagcacttgccaagagtgtttca
>38cdd843d0b87e8d7dd0d4218c63d6db\_2
agctccaatagcgtatattaaagttgttgcagttaaaaagctcgtagttggatttcggagtgggccagttggtccgccgcaaggtgtgttactgactggtttgctcttcttcgcaaagactgcgtgtgctctttattgagtgtgcgtaggatttacgacgtttactttgaaaaaattagagtgttcaaagcaggctattgcttgaatacatgagcatggaataatggaataggattttggtcccattttgttggtttctaggaccgaagtaatgattaagagggacaattgggggcatccgtatttcgttgtcagatgtgaaattcttggatttacgaaagacgaacaactgcgaaagcacttgccaagagtgttttca
>3b0e5dd1bc823b191b678ee24dfc8059\_2
agctccaatagcgtatattaaagttgttgcagttaaaaagctcgtagttggattttcggagtgggcccgttggtccgccgcaaggtgtgttactgactggtctgctcttcttcgcaaagactgcgtgtgctctttgttgagtgtgcgtaggatttacgacgtttactttgaaaaaattagagtgttcaaagcaggctattgcttgaatacatgagcatggaataatggaataggactttggtcccattttgttggtttctaggaccgaagtaatgattaagagggacaattgggggcatccgtatttcgttgtcagaggtgaaattcttggatttacgaaagacgaacaactgcgaaagcatttgccaagagtgttttca
>3b6305bfe1a878697061d1795b2513d3\_2
agctccaatagcgtatattaaagttgttgtcagttaaaaagctcgtagttggatttcggagtgggcccgttggtccgccgcaaggtgtgttactgactggtctgctcttcttcgcaaagactgcgtgtgctctttgttgagtgtgcgtaggatttacgacgtttactttgaaaaaattagagtgttcaaagcaggctattgcttgaatacatgagcatggaataatggaataggactttggtcccattttgttggtttctaggaccgaagtaatgattaagagggacaattgggggcatccgtatttcgttgtcagaggtgaaattcttggatttacgaaagacgaacaactgcgaaagcacttgccaagagtgttttca
>3d0a96bf2948c5a58e896e47dbc5b550\_2
agctccaatagcgtatattaaagttgttgcagttaaaaagctcgtagttggatttcggagtgggccagttggtccgccgcaaggtgtgttactgactggtttgctcttcttcgcaaagactgcgtgtgctctttattgagtgtgcgtaggatttacgacgtttactttgaaaaaattagagtgttcaaagcaggctattgcttgaatacatgagcatggaataatggaataggattttggtcccattttgttggtttctaggaccgaagtaatgattaagagggacaattgggggcatccgtatttcgttgtcagaggtgaaattcttggatttacgtaaagacgaacaactgcgaaagcacttgccaagagtgttttca
>3dd05a40a4096aa9cbb1851a4a60fa9f\_2
agctccaatagcgtatattaaagttgttgcagttaaaaagctcgtagttggatttcggagtgggccagttggtccgccgcaaggtgtgttactgactggtttgctcttcttcgcaaagactgcgtgtgctctttattgagtgtgcgtaggatttacgacgtttactttgaaaaaattagagtgttcaaagcaggctattgcttgaatacatgagcatggaataatggaataggattttggtcccatttttgttggtttctaggaccgaagtaatgattaagagggacaattgggggcgatccgtatttcgttgtcagaggtgaaattcttggatttacgaaagacgaacaactgcgaaagcacttgccaagagtgttttca
>3eb87f258b9daaa18bd5eabb421b6f3e\_2
agctccaatagcgtatattaaagttgttgcagttaaaaagctcgtagttggatttcggagtgggccagttggtccgccgcaaggtgtgttactgactggtttgctcttcttcgcaaagactgcgtgtgctctttattgagtgtgcgtaggatttacgacgtttactttgaaaaaattagagtgttcaaagcaggctattgcttgaatacatgagcatggaataatggaataggattttggtcccattttgttggtttctaggaccgaagtaatgattaagagggacaattggggcatccgtatttcgttgtcagaggtgaaattcttggatttacgaaagacgaacaactgcgaaagcacttgccaagagtgtttttca
>3f2118b9bda5772186e2abd7ea5d3936\_2
agctccaatagcgtatattaaagttgttgcagttaaaaagctcgtagttggatttcggagtgggcccgttggtccgccgcaaggtgtgttactgactggtctgctcttcttcgcaaagactgcgtgtgctctttgttgagtgtgcgtaggatttacgacgtttactttgaaaaaattagagtgttacaaagcaggctattgcttgaatacatgagcatggaataatggaataggactttggtcccattttgttggtttctaggaccgaagtaatgattaagagggacaattgggggcatccgtatttcgttgtcagaggtgaaattcttggatttacgaaagacgaacaactgcgaaagcacttgccaagagtgttttca
>3f424cd217589568d192aa3a154d3c3b\_2
agctccaatagcgtatattaaagttgttgcagttaaaaagctcgtagttggatttcggagtgggccagttggtccgccgcaaggtgtgttactgactggtttgctcttcttcgcaaagactgcgtgtgctctttattgagtgtgcgtaggatttacgacgtttactttgaaaaaattagagtgttcaaagcaggctattgcttgaatacatgagcatggaataatggaataggattttggtcccattttgttggtttctaggaccgaagtaatgattaagagggacaattgggggcatccgtatttcgttgtcagaggtgaaattctttggatttacgaaagacgaacaactgcgaaagcacttgccaagagtgttttca
>40ae3283a36b5d762a14a18d58050737\_2
agctccaatagcgtatattaaagttgttgcagttaaaaagctcgtagttggagtttcggagtgggccagttggtccgccgcaaggtgtgttactgactggtctgctcttcttcgcaaagactgcgtgtgctctttgttgagtgtgcgtaggatttacgacgtttactttgaaaaaattagagtgttcaaagcaggctattgcttgaatacatgagcatggaataatggaataggactttggtcccattttgttggtttctaggaccgaagtaatgattaagagggacaattgggggcatccgtatttcgttgtcagaggtgaaattcttggatttacgaaagacgaacaactgcgaaaagcatttgccaagagtgttttca
>40ebb88c680137f85902423d5748b741\_2
agctccaatagcgtatattaaagttgttgcagttaaaaaagctcgtagttggatttcggagtgggcccgttggtccgccgcaaggtgtgttactgactggtctgctcttcttcgcaaagactgcgtgtgctctttgttgagtgtgcgtaggatttacgacgtttactttgaaaaaattagagtgttcaaagcaggctattgcttgaatacatgagcatggaataatggaataggactttggtcccattttgttggtttctaggaccgaagtaatgattaagagggacaattgggggcatccgtattcgttgtcagaggtgaaattcttggatttacgaaagacgaacaactgcgaaagcacttgccaagagtgttttca
>41d7da31f4801ce2b1434f1c8683e3e1\_2
agctccaatagcgtatattaaagttgttgcagttaaaaagctcgtagttggatttcggagtgggccagttggtccgccgcaaggtgtgttactgactggtttgctcttcttcgcaaagactgcgtgtgctctttattgagtgtgcgtaggatttacgacgtttactttgaaaaaattagagtgttcaaagcaggctattgcttgaatacatgagcatggaataatggaataggattttggtcccattttgttggtttctaggaccgaagtaatgattaagagggacaattggggcatccgtatttcgttgtcagaggtgaaattcttggatttacgaaaagacgaacaactgcgaaagcacttgccaagagtgttttca
>429a8aa8bb525b232f4c7ef3f4fe08bf\_2
agctccaatagcgtatattaaagttgttgcagttaaaaagctcgtagttggatttcggagtgggcccgttggtccgccgcaaggtgtgttactgactggtctgctcttcttcgcaaagactgcgtgtgctctttgttgagtgtgcgtaggatttacgacgtttactttgaaaaaattagagtgttcaaagcaggctattgcttgaatacatgagcatggaataatggaataggactttggtcccattttgttggtttctaggaccgaagtaatgattaagagggacaattggggcatccgtatttcgttgtcagaggtgaaattcttggatttacgaaaagacgaacaactgcgaaaagcacttgccaagagtgttttca
>460c8209c10178e5c05adb489f8faf4e\_2
agctccaatagcgtatattaaagttgttgcagttaaaaagctcgtagttggatttcggagtgggccagttggtccgccgcaaggtgtgttactgactggtttgctcttcttcgcaaagactgcgtgtgctctttattgagtgtgcgtaggatttacgacgtttactttgaaaaaattgagtgttcaaagcaggctattgcttgaatacatgagcatggaataatggaataggattttggtcccattttgttggtttctaggaccgaagtaatgattaagagggacaattgggggcatccgtatttcgttgtcagaggtgaaattcttggatttacgaaagacgaacaactgcgaaagcacttgccaagagtagttttca
>4714440a6e88e4d7c47ca477e765dcac\_2
agctccactagcgtatattaaagttgttgcagttaaaaagctcgtagttggatttcggagtgggcccgttggtccgccgcaaggtgtgttactgactggtctgctcttcttcgcaaagactgcgtgtgctctttgttgagtgtgcgtaggatttacgacgtttactttgaaaaaattagagtgttcaaagcaggctattgcttgaatacatgagcatggaataatggaataggactttggtcccattttgttggtttctaggaccgaagtaatgattaagagggacaattgggggcatccgtatttcgttgtcagaggtgaaattcttggatttacgaaagacgaacaactgcgaaagcacttgccaagagtgttttca
>479abeb846d511eb5710295dec15a607\_2
agctccaatagcgtatattaaagttgttgcagttaaaaagctcgtagttggatttcggagtgggcccgttggtccgccgcaaggtgtgttactgactggtctgctcttcttcgcaaagactgtgtgtgctctttgttgagtgtgcgtaggatttacgacgtttactttgaaaaaattagagtgttcaaagcaggctattgcttgaatacatgagcatggaataatggaataggactttggtcccattttgttggtttctaggaccgaagtaatgattaagagggacaattgggggcatccgtatttcgttgtcagaggtgaaattcttggatttacgaaagacgaacaactgcgaaagcacttgccaagagtgttttca
>47dda60279af6bb9f74948b0e15919af\_2
agctccaatagcgtatattaaagttgttgcagttaaaaagctcgtagttggatttcggagtgggccagttggtccgccgcaaggtgtgttactgactggtctgctcttcttcgcaaagactgcgtgtgctctttgttgagtgtgcgtaggatttacgacgtttactttgaaaaaattagagtgttcaaagcaggctattgcttgaatacatgagcatggaataatggaataggactttggtcccattttgttggtttctaggaccgaagtaatgattaagagggacaattgggggcatccgtatttcgttgtcagaggtgaaattcttggatttacgaagacgaacaactgcgaaagcatttgccaagagtgttttca
>4802b7679e8ba0e2e67e0d0d949472b3\_2
agctccaatagcgtatattaaagttgttgcagttaaaaaagctcgtagttggatttcggagtgggccagttggtccgccgcaaggtgtgttactgactggtttgctcttcttcgcaaagactgcgtgtgctctttattgagtgtgcgtaggatttacgacgtttactttgaaaaaattgagtgttcaaagcaggctattgcttgaatacatgagcatggaataatggaataggattttggtcccattttgttggtttctaggaccgaagtaatgattaagagggacaattgggggcatccgtatttcgttgtcagaggtgaaattcttggatttacgaaagacgaacaactgcgaaagcacttgccaagagtgtttca
>4845d5fa4c583c2f43769b751462a284\_2
agctccaatagcgtatattaaagttgttgcagttaaaaagctcgtagttggatttcggagtgggccagttggtccgccgcaaggtgtgttactgactggtttgctcttcttcgcaaagactgcgtgtgctctttattgagtgtgcgtaggattttacgacgtttactttgaaaaaattagagtgttcaaagcaggctattgcttgaatacatgagcatggaataatggaataggattttggtcccattttgttggtttctaggaccgaagtaatgattaagagggacaattgggggcatccgtatttcgttgtcagaggtgaaattcttggatttacgaaagacgaacaactgcgaaagcacttgccaagagtgttttca
>49b6171bbe66add3e28cf49a870db7a7\_2
agctccaatagcgtatattaaagttgttgcagttaaaaagctcgtagttggatttcggagtgggccagttggtccgccgcaaggtgtgttactgactggtttgctcttcttcgcaaagactgcgtgtgctctttattgagtgtgcgtaggatttacgacgtttactttgaaaaattagagtgttcaaagcaggctattgcttgaatacatgagcatggaataatggaataggattttggtcccattttgttggtttctaggaccgaagtaatgattaagagggacaattggggcatccgtatttcgttgtcagaggtgaaattcttggatttacgaaagacgaacaactgcgaaagcacttgccaagagtgttttca
>4c4f199968fe0f6883a529b4546c61ef\_2
agctccaatagcgtatattaaagttgttgcagttaaaaagctcgtagttggatttcggagtgggcccgttggtccgccgcaaggtgtgttactgactggtctgctcttcttcgcaaagactgcgtgtgctctttgttgagtgtgcgtaggatttacgacgtttactttgaaaaaattagagtgttcaaagcaggctattgcttgaatacatgagcatggaataatggaataggactttggtcccattttgttggtttctaggaccgaagtaatgattaatagggacaattgggggcatccgtatttcgttgtcagaggtgaaattcttggatttacgaaagacgaacaactgcgaaagcacttgccaagagtgttttca
>4cc6cae6878514bc3945f355e5a8cc9c\_2
agctccaatagcgtatattaaagttgttgcagttaaaaagctcgtagttggatttcggagtgggccagttggtccgccgcaaggtgtgttactgactggtctgctcttcttcgcaaagactgcgtgtgctctttgttgagtgtgcgtaggatttacgacgtttactttgaaaaaaattagagtgttcaaagcaggctattgcttgaatacatgagcatggaataatggaataggactttggtcccatttttgttggtttctaggaccgaagtaatgattaagagggacaattgggggcatccgtatttcgttgtcagaggtgaaattcttggatttacgaaagacgaacaactgcgaaagcatttgccaagagtgttttca
>4d1bb7a5b949e91b15abe56dfa5cc6e6\_2
agctccaatagcgtatattaaagttgttgcagttaaaaagctcgtagttggatttcggagtgggccagttggtccgccgcaaggtgtgttactgactggtttgctcttcttcgcaaagactgcgtgtgctctttattgagtgtgcgtaggatttacgacgtttactttgaaaaaattgagtgttcaaagcaggctattgcttgaatacatgagcatggaataatggaataggatttttggtcccattttgttggtttctaggaccgaagtaatgattaagagggacaattgggggcatccgtatttcgttgtcagaggtgaaattcttggatttacgaaagacgaacaactgcgaaagcacttgccaagagtgttttca
>4d943c169f1895e46b3c8158d6b2e069\_2
agctccaatagcgtatattaaagttgttgcagttaaaaagctcgtagttggatttcggagtgggccagttggtccgccgcaaggtgtgttactgactggtttgctcttcttcgcaaagactgcgtgtgctctttattgagtgtgcgtaggatttacgacgtttactttgaaaaaattagagtgttcaaagcaggctattgcttgaatacatgagcatggaataatggaataggattttggtcccattttgttggtttctaggaccgaagtaatgattaagagggacaattgggggcatccgtatttcgttgtcagaggtgaaattcttggaattacgaaagacgaacaactgcgaaagcacttgccaagagtgttttca
>50372c00e4b965e4dd94e8ac4a88fa16\_2
agctccaatagcgtatattaaagttgttgcagttaaaaagctcgtagttggatttcggagtgggccagttggtccgccgcaaggtgtgttactgactggtttgctcttcttcgcaaagactgcgtgtgctctttattgagtgtgcgtaggatttacgacgtttactttgaaaaaattagagtgttcaaagcaggctattgcttgaatacatgagcatggaataatggaataggatttttggtcccattttgttggtttctaggaccgaagtaatgattaagagggacaattggggcatccgtatttcgttgtcagaggtgaaattcttggatttacgaaagacgaacaactgcgaaagcacttgccaagagtgttttca
>517940505776f728de0afae981e1b7dd\_2
agctccaatagcgtatattaaagttgttgcagttaaaaagctcgtagttggattttcggagtgggcccgttggtccgccgcaaggtgtgttactgactggtctgctcttcttcgcaaagactgcgtgtgctctttgttgagtgtgcgtaggatttacgacgtttactttgaaaaaattagagtgttcaaagcaggctattgcttgaatacatgagcatggaataatggaataggactttggtcccattttgttggtttctaggaccgaagtaatgattaagagggacaattgggggcatccgtatttcgttgtcagaggtgaaattcttggatttacgaaagacgaacaactgcgaaagcacttgccaagagtgtttca
>5274f0e765b92b9c85f23d4bf44bc008\_2
agctccaatagcgtatattaaagttgttgcagttaaaaagctcgtagttggatttcggagtgggccagttggtccgccgcaaggtgtgttactgactggtttgctcttcttcgcaaagactgcgtgtgctctttattgagtgtgcgtaggatttacgacgtttactttgaaaaaaattagagtgttcaaagcaggctattgcttgaatacatgagcatggaataatggaataggattttggtcccattttgttggtttctaggaccgaagtaatgattaagagggacaattgggggcatccgtatttcgtgtcagaggtgaaattcttggatttacgaaagacgaacaactgcgaaagcacttgccaagagtgttttca
>537e4ec9d60a17cf3456799bdde984c6\_2
agctccaatagcgtatattaaagttgttgcagttaaaaagctcgtagttggatttcggagtgggccagttggtccgccgcaaggtgtgttactgactggtctgctcttcttcgcaaagactgcgtgtgctctttgttgagtgtgcgtaggatttacgacgtttactttgaaaaaattagagtgttcaaagcaggctattgcttgaatacatgagcacggaataatggaataggactttggtcccattttgttggtttctaggaccgaagtaatgattaagagggacaattgggggcatccgtatttcgttgtcagaggtgaaattcttggatttacgaaagacgaacaactgcgaaagcatttgccaagagtgttttca
>53f9f65e58a6479ebe3c099b16f24995\_2
agctccaatagcgtatattaaagttgttgcagttaaaaagctcgtagttggatttcggagttgggcccgttggtccgccgcaaggtgtgttactgactggtctgctcttcttcgcaaagactgcgtgtgctctttgttgagtgtgcgtaggatttacgacgtttactttgaaaaaattagagtgttcaaagcaggctattgcttgaatacatgagcatggaataatggaataggactttggtcccattttgttggtttctaggaccgaagtaatgattaagagggacaattgggggcatccgtatttcgttgtcagaggtgaaattcttggatttacgaaagacgaacaactgcgaaagcacttgccaagagtgttttca
>562feafdc059d6c69703414ebe8882e1\_2
agctccaatagcgtatattaaagttgttgcagttaaaaagctcgtagttggatttcggagtgggccagttggtccgccgcaaggtgtgttactgactggtttgctcttcttcgcaaagactgcgtgtgctctttattgagtgtgcgtaggatttacgacgtttactttagaaaaaattagagtgttcaaagcaggctattgcttgaatacatgagcatggaataatggaataggattttggtcccattttgttggtttctaggaccgaagtaatgattaagagggacaattgggggcatccgtatttcgttgtcagaggtgaattcttggatttacgaaagacgaacaactgcgaaagcacttgccaagagtgttttca
>583b974a7cdbc6971cdce8158f999b9c\_2
agctccaatagcgtatattaaagttgttgcagttaaaaagctcgtagttggatttcggagtgggcccgttggtccgccgcaaggtgtgttactgactggtctgctcttcttcgcaaagactgcgtgtgctctttgttgagtgtgcgtaggatttacgacgtttactttagaaaaaattagagtgttcaaagcaggctattgcttgaatacatgagcatggaataatggaataggactttggtcccattttgttggtttctaggaccgaagtaatgattaagagggacaattgggggcatccgtatttcgttgtcagaggtgaaattcttggatttacgaaagacgaacaactgcgaaagcacttgccaagagtgtttca
>594f1a3e00802df032a02afce29ef620\_2
agctccaatagcgtatattaaagttgttgcagttaaaaagctcgtagttggatttcggagtgggccagttggtccgccgcaaggtgtgttactgactggtctgctcttcttcgcaaagactgcgtgtgctctttgttgagtgtgcgtaggatttacgatgtttactttgaaaaaattagagtgttcaaagcaggctattgcttgaatacatgagcatggaataatggaataggactttggtcccattttgttggtttctaggaccgaagtaatgattaagagggacaattgggggcatccgtatttcgttgtcagaggtgaaattcttggatttacgaaagacgaacaactgcgaaagcatttgccaagagtgttttca
>5aaaa4436a3ecb67aadb8c68dec5ec43\_2
agctccaatagcgtatattaaagttgttgcagttaaaaagctcgtagttggatttcggagtgggcccgttggtccgccgcaaggtgtgttactgactggtctgctcttcttcgcaaagactgcgtgtgctctttgttgagtgtgcgtaggatttacgacgtttactttgaaaaaattagagtgttcaaagcaggctattgcttgaatacatgagcatggaataatggaataggactttggtcccattttgttggtttctaggaccgaagtaatgattaagagggacaattgggggcgatccgtatttcgttgtcagaggtgaaattcttggatttacgaaagacgaacaactgcgaaagcacttgccaagagtgttttca
>5b9e23043632525b130fe9ff0469dae0\_2
agctccaatagcgtatattaaagttgttgcagttaaaagctcgtagttggatttcggagtgggcccgttggtccgccgcaaggtgtgttactgactggtctgctcttcttcgcaaagactgcgtgtgctctttgttgagtgtgcgtaggatttacgacgtttactttgaaaaaattagagtgttcaaagcaggctattgcttgaatacatgagcatggaataatggaataggactttggtcccattttgttggtttctaggaccgaagtaatgattaagagggacaattgggggcatccgtatttcgttgtcagaggtgaaattcttggatttacgaaagacgaacaactgcgaaaagcacttgccaagagtgttttca
>5dcc08b6f49a093890bea495fc57a304\_2
agctccaatagcgtatattaaagttgttgcagttaaaagctcgtagttggatttcggagtgggcccgttggtccgccgcaaggtgtgttactgactggtctgctcttcttcgcaaagactgcgtgtgctctttgttgagtgtgcgtaggatttacgacgtttactttgaaaaaattagagtgttcaaagcaggctattgcttgaatacatgagcatggaataatggaataggactttggtcccattttgttggtttctaggaccgaagtaatgattaagagggacaattgggggcatccgtatttcgttgtcagaggtgaaatttcttggatttacgaaaagacgaacaactgcgaaagcacttgccaagagtgttttca
>5dfa19b1af68a76b6f1646b0508699e8\_2
agctccaatagcgtatattaatgttgttgcagttaaaaagctcgtagttggatttcggagtgggccagttggtccgccgcaaggtgtgttactgactggtctgctcttcttcgcaaagactgcgtgtgctctttgttgagtgtgcgtaggatttacgacgtttactttgaaaaaattagagtgttcaaagcaggctattgcttgaatacatgagcatggaataatggaataggactttggtcccattttgttggtttctaggaccgaagtaatgattaagagggacaattgggggcatccgtatttcgttgtcagaggtgaaattcttggatttacgaaagacgaacaactgcgaaagcatttgccaagagtgttttca
>5e22fdf0e57123432f47d221aa9cf788\_2
agctccaatagcgtatattaaagttgttgcagttaaaaagctcgtagttggatttcggagtgggccagttggtccgccgcaaggtgtgttactgactggtttgctcttcttcgcaaagactgcgtgtgctctttattgagtgtgcgtaggatttacgacgtttactttgaaaaaattagagtgttcaaagcaggctattgcttgaatacatgagcatggaataatggaataggattttggtcccattttgttggtttctaggaccgaagtaatgattaagagggacaattgggggcatccgtatttcgttgtcagaggtgaaattcttggatttacgaaagacggaacaactgcgaaagcacttgccaagagtgttttca
>5e4af4b0826c81eba2850af96cb1ec63\_2
agctccaatagcgtatattaaagttgttgcagttaaaaaagctcgtagttggatttcggagtgggccagttggtccgccgcaaggtgtgttactgactggtttgctcttcttcgcaaagactgcgtgtgctctttattgagtgtgcgtaggatttacgacgtttactttgaaaaaattagagtgttcaaagcaggctattgcttgaatacatgagcatggaataatggaataggattttggtcccattttgttggtttctaggaccgaagtaatgattaagagggacaattgggggcatccgtatttcgttgtcagaggtgaaattcttggattttacgaaagacgaacaactgcgaaagcacttgccaagagtgttttca
>602bfa31d2ce255ded86bde455754855\_2
agctccaatagcgtatattaaagctgttgcagttaaaaagctcgtagttggatttcggagtgggccagttggtccgccgcaaggtgtgttactgactggtctgctcttcttcgcaaagactgcgtgtgctctttgttgagtgtgcgtaggatttacgacgtttactttgaaaaaattagagtgttcaaagcaggctattgcttgaatacatgagcatggaataatggaataggactttggtcccattttgttggtttctaggaccgaagtaatgattaagagggacaattgggggcatccgtatttcgttgtcagaggtgaaattcttggatttacgaaagacgaacaactgcgaaagcatttgccaagagtgttttca
>6120febccbac72a37ce66bfd7afebc6b\_2
agctccaatagcgtatattaaagttgttgcagttaaaaaagctcgtagttggatttcggagtgggcccgttggtccgccgcaaggtgtgttactgactgggtctgctcttcttcgcaaagactgcgtgtgctctttgttgagtgtgcgtaggatttacgacgtttactttgaaaaaattagagtgttcaaagcaggctattgcttgaatacatgagcatggaataatggaataggactttggtcccattttgttggtttctaggaccgaagtaatgattaagagggacaattgggggcatccgtatttcgttgtcagaggtgaaattcttggatttacgaaagacgaacaactgcgaaagcacttgccaagagtgtttca
>6193ae6e35a473d381edde691303f13e\_2
agctccaatagcgtatattaaagttgttgcagttaaaaagctcgtagttggatttcggagtgggccagttggtccgccgcaaggtgtgttactgactggtctgctcttcttcgcaaagactgcgtgtgctctttgttgagtgtgcgtaggatttacgacgtttacttgaaaaaattgagtgttcaaagcaggctattgcttgaatacatgagcatggaataatggaataggactttggtcccattttgttggtttctaggaccgaagtaatgattaagagggacaattgggggcatccgtatttcgttgtcagaggtgaaattcttggatttacgaaagacgaacaactgcgaaagcatttgccaagagtgttttca
>6282aabd542e3b93610fc09911c10f22\_2
agctccaatagcgtatattaaagttgttgcagttaaaaagctcgtagttggatttcggagtgggcccgttggtccgccgcaaggtgtgttactgactggtctgctcttcttcgcaaagactgcgtgtgctctttgttgagtgtgcgtaggatttacgacgtttactttgaaaaaattgagtgttcaaagcaggctattgcttgaatacatgagcatggaataatggaataggactttggtcccattttgttggtttctaggaccgaagtaatgattaagagggacaattggggcatccgtatttcgttgtcagaggtgaaattcttggatttacgaaagacgaacaactgcgaaagcacttgccaagagtgtttca
>643c98c076945e12074061e01e1cae1d\_2
agctccaatagcgtatattaaagttgttgcagttaaaaagctcgtagttggatttcggagtgggtccgttggtccgccgcaaggtgtgttactgactggtctgctcttcttcgcaaagactgcgtgtgctctttgttgagtgtgcgtaggatttacgacgtttactttgaaaaaattagagtgttcaaagcaggctattgcttgaatacatgagcatggaataatggaataggactttggtcccattttgttggtttctaggaccgaagtaatgattaagagggacaattgggggcatccgtatttcgttgtcagaggtgaaattcttggatttacgaaagacgaacaactgcgaaagcacttgccaagagtgttttca
>6463e8348fe8e8a3d4e016d84d549439\_2
agctccaatagcgtatattaaagttgttgcagttaaaaaagctcgtagttggatttcggagtgggccagttggtccgccgcaaggtgtgttactgactggtttgctcttcttcgcaaagactgcgtgtgctctttattgagtgtgcgtaggatttacgacgtttactttgaaaaaattagagtgttcaaagcaggctattgcttgaatacatgagcatggaataatggaataggatttggtcccattttgttggtttctaggaccgaagtaatgattaagagggacaattggggcatccgtatttcgttgtcagaggtgaaattcttggatttacgaaagacgaacaactgcgaaagcacttgccaagagtgttttca
>64a191b2a9dad1b77b9e9a11165df774\_2
agctccaatagcgtatattaaagttgttgcagttaaaaagctcgtagttggatttcggagtgggccagttggtccgccgcaaggtgtgttactgactggtctgctcttcttcgcaaagactgcgtgtgctctttgttgagtgtgcgtaggatttacgacgtttactttgaaaaaaattagagtgttcaaagcaggctattgcttgaatacatgagcatggaataatggaataggactttggtcccattttgttggtttctaggaccgaagtaatgattaagagggacaattgggggcatccgtatttcgtgtcagaggtgaaattcttggatttacgaaagacgaacaactgcgaaagcatttgccaagagtgttttca
>64ef29a40ef3b35531ab8f48f889627d\_2
agctccaatagcgtatattaaagttgttgcagttaaaaagctcgtagttggatttcggagtgggccagttggtccgccgcaaggtgtgttactgactggtttgctcttcttcgcaaagactgcgtgtgctctttattgagtgtgcgtaggatttacgacgtttactttgaaaaaattagagtgttcaaagcaggctattgcttgaatacatgagcatggaataatggaataggattttggtcccattttgttggtttctaggaccgaagtaatgattaagagggacaattgggggcatccgtatttcgttgtcggaggtgaaattcttggatttacgaaagacgaacaactgcgaaagcacttgccaagagtgttttca
>6562aa3fc19a9939c04a840878695be5\_2
agctccaatagcgtatattaaagttgttgcagttaaaaaagctcgtagttggatttcggagtgggccagttggtccgccgcaaggtgtgttactgactggtttgctcttcttcgcaaagactgcgtgtgctctttattgagtgtgcgtaggatttacgacgtttactttgaaaaaattagagtgttcaaagcaggctattgcttgaatacatgagcatggaataatggaataggattttggtcccattttgttggtttctaggaccgaagtaatgattaagagggacaattgggggcatccgtatttcgttgttcagaggtgaaattcttggatttacgaaagacgaacaactgcgaagcacttgccaagagtgttttca
>661c782a5a432a004aede6a370ce164a\_2
agctccaatagcgtatgttaaagttgttgcagttaaaaagctcgtagttggatttcggagtgggccagttggtccgccgcaaggtgtgttactgactggtctgctcttcttcgcaaagactgcgtgtgctctttgttgagtgtgcgtaggatttacgacgtttactttgaaaaaattagagtgttcaaagcaggctattgcttgaatacatgagcatggaataatggaataggactttggtcccattttgttggtttctaggaccgaagtaatgattaagagggacaattgggggcatccgtatttcgttgtcagaggtgaaattcttggatttacgaaagacgaacaactgcgaaagcatttgccaagagtgttttca
>664bc016e92438b94b8afedccf9155c8\_2
agctccaatagcgtatattaaaagttgttgcagttaaaaagctcgtagttggatttcggagtgggcccgttggtccgccgcaaggtgtgttactgactggtctgctcttcttcgcaaagactgcgtgtgctctttgttgagtgtgcgtaggatttacgacgtttactttgaaaaaattagagtgttcaaagcaggctattgcttgaatacatgagcatggaataatggaataggactttggtcccattttgttggtttctaggaccgaagtaatgattaagagggacaattgggggcatccgtatttcgttgtcagaggtgaaattcttggatttacgaaagacgaacaactgcgaaagcacttgccaagagtgttttca
>66b1fad648c6c6226e26369c7237b82f\_2
agctccaatagcgtatattaaagttgttgcagttaaaaagctcgtagttggatttcggagtgggccagttggtccgccgcaaggtgtgttactgactggtctgctcttcttcgcaaagactgcgtgtgctctttgttgagtgtgcgtaggatttacgacgtttactttgaaaaaaattagagtgttcaaagcaggctattgcttgaatacatgagcatggaataatggaataggactttggtcccattttgttggtttctaggaccgaagtaatgattaagaggacaattgggggcatccgtatttcgttgtcagaggtgaaattcttggatttacgaaagacgaacaactgcgaaagcatttgccaagagtgtttca
>67a8bfca99af983742006fd2926626a9\_2
agctccaatagcgtatattaaagttgttgcagttaaaaaagctcgtagttggatttcggagtgggccagttggtccgccgcaaggtgtgttactgactggtctgctcttcttcgcaaagactgcgtgtgctctttgttgagtgtgcgtaggatttacgacgtttactttgaaaaaattagagtgttcaaagcaggctattgcttgaatacatgagcatggaataatggaataggactttggtcccattttgttggtttctaggaccgaagtaatgattaagagggacaattgggggcatccgtattcgttgtcagaggtgaaattcttggatttacgaaagacgaacaactgcgaaagcatttgccaagagtgttttca
>67dbd6acd5b1ad9bdd8d58f1fd81f881\_2
agctccaatagcgtatattaaagttgttgcagttaaaaagctcgtagttggatttcggagtgggccagttggtccgccgcaaggtgtgttactgactggtttgctcttcttcgcaaagactgcgtgtgctctttattgagtgtgcgtaggatttacgacgtttactttgaaaaaattagagtgttcaaagcaggctattgcttgaatacatgagcatggaataatggaataggattttggtcccattttgtggtttctaggaccgaagtaatgattaagagggacaattgggggcatccgtatttcgttgtcagaggtgaaattcttggatttacgaaagacgaacaactgcgaaagcacttgccaagagtgtttttca
>68dbeea12c74cd423b703090ee1515b4\_2
agctccaatagcgtatattaaagttgttgcagttaaaaagctcgtagttggatttcggagtgggccagttggtccgccgcaaggtgtgttactgactggtttgctcttcttcgcaaagactgcgtgtgctctttattgagtgtgcgtaggatttacgacgtttactttgaaaaaaattagagtgttcaaaagcaggctattgcttgaatacatgagcatggaataatggaataggattttggtcccattttgttggtttctaggaccgaagtaatgattaagagggacaattgggggcatccgtatttcgttgtcagaggtgaaattcttggatttacgaaagacgaacaactgcgaaagcacttgccaagagtgttttca
>68f3e54cee43ba9d4a30fa2fd574a0d9\_2
agctccaatagcgtatattaaagttgttgcagttaaaaagctcgtagttggatttcggagtgggccagttggtccgccgcaaggtgtgttactgactggtttgctcttcttcgcaaagactgcgtgtgctctttattgagtgtgcgtaggatttatgacgtttactttgaaaaaattagagtgttcaaagcaggctattgcttgaatacatgagcatggaataatggaataggattttggtcccattttgttggtttctaggaccgaagtaatgattaagagggacaattgggggcatccgtatttcgttgtcagaggtgaaattcttggatttacgaaagacgaacaactgcgaaagcacttgccaagagtgttttca
>69527b89ab4e7f38319c68d045b71b99\_2
agctccaatagcgtatattaaagttgttgcagttaaaaagctcgtagttggatttcggagtgggccagttggtccgccgcaaggtgtgttactgactggtctgctcttcttcgcaaagactgcgtgtgctctttgttgagtgtgcgtaggatttacgacgtttactttgaaaaaattagagtgttcaaagcaggctattgcttgaatacatgagcatggaataatggaataggactttggtcccattttgttggtttctaggaccgaagtaatgattaagagggacaattgggggcatccgtatttcgttgtcagaggtgaaattcttggatttacgaaagacgaacaactgcgaaaagcatttgccaagagtgtttttca
>6a1addb8d6bb9edfe8b65c8868f39e42\_2
agctccaatagcgtatattaaagttgttgcagttaaaaagctcgtagttggatttcggagtgggccagttggtccgccgcaaggtgtgttactgactggtttgctcttcttcgcaaagactgcgtgtgctctttattgagtgtgcgtaggatttacgacgtttactttgaaaaaattagagtgttcaaagcaggctattgcttgaatacatgagcatggaataatggaataggatttggtcccatttgttggtttctaggaccgaagtaatgattaagagggacaattgggggcatccgtatttcgttgtcagaggtgaaattcttggatttacgaaagacgaacaactgcgaaagcacttgccaagagtgttttca
>6a804c37dcd3a3a30512d1c8ef4318fd\_2
agctccaatagcgtatattaaagttgttgcagttaaaaagctcgtagttggatttcggagtgggccagttggtccgccgcaaggtgtgttactgactggtttgctcttcttcgcaaagactgcgtgtgctctttattgagtgtgcgtaggatttacgacgtttacttgaaaaaattagagtgttcaaagcaggctattgcttgaatacatgagcatggaataatggaataggattttggtcccattttgttggtttctaggaccgaagtaatgattaagagggacaattgggggcatccgtatttcgttgtcagaggtgaaattcttggatttacgaaagacgaacaactgcgaaagcacttgccaagagtgttttca
>6ab07999ef57aaf969719030814ee2d1\_2
agctccaatagcgtatattaaagttgttgcagttaaaaaagctcgtagttggatttcggagtgggcccgttggtccgccgcaaggtgtgttactgactggtctgctcttcttcgcaaagactgcgtgtgctctttgttgagtgtgcgtaggatttacgacgtttacttgaaaaaaattagagtgttcaaagcaggctattgcttgaatacatgagcatggaataatggaataggactttggtcccattttgttggtttctaggaccgaagtaatgattaagagggacaattgggggcatccgtatttcgttgtcagaggtgaaattcttggatttacgaaagacgaacaactgcgaaagcacttgccaagagtgtttca
>6d72edc9530ba1bd2482084b2f16913d\_2
agctccaatagcgtatattaaagttgttgcagttaaaaagctcgtagttggatttcggagtgggccagttggtccgccgcaaggtgtgttactgactggtttgctcttcttcgcaaagactgcgtgtgctctttattgagtgtgcgtaggatttacgacgtttactttgaaaaaattagagtgttcaaagcaggctattgcttgaatacatgagcatggaataatggaataggattttggtcccattttgtggtttctaggaccgaagtaatgattaagagggacaattggggcatccgtatttcgttgtcagaggtgaaattcttggatttacgaaagacgaacaactgcgaaagcacttgccaagagtgttttca
>6ea4e7aca4e662a0db8c8455703f0eee\_2
agctccaatagcgtatattaaagttgttgcagttaaaaagctcgtagttggatttcggagtgggccagttggtccgccgcaaggtgtgttactgactggtctgctcttcttcgcaaagactgcgtgtgctctttgttgagtgtgcgtaggatttacgacgtttactttgaaaaaattagagtgttcaaagcaggctattgcttgaatacatgagcatggaataatggaataggactttggtcccattttgttggtttctaggaccgaagtaatgattaagagggacaattgggggcatccgtatttcgtgtcagaggtgaaattctggatttacgaaagacgaacaactgcgaaagcacttgccaagagtgttttca
>6ed7df45a36d607aa2c45e7d640f2fbb\_2
agctccaatagcgtatattaaagttgttgcagttaaaaagctcgtagttggatttcggagtgggccagttggtccgccgcaaggtgtgttactgactggtttgctcttcttcgcaaagactgcgtgtgctctttattgagtgtgcgtaggatttacgacgtttactttgaaaaaaattgagtgttcaaagcaggctattgcttgaatacatgagcatggaataatggaataggattttggtcccattttgttggtttctaggaccgaagtaatgattaagagggacaattgggggcatccgtatttcgttgtcagaggtgaaattcttggatttacgaaagacgaacaactgcgaaagcacttgccaagagtgtttca
>6f97d0a942305110cc422d30688caaba\_2
agctcaatagcgtatattaaagttgttgcagttaaaaagctcgtagttggatttcggagtgggccagttggtccgccgcaaggtgtgttactgactggtctgctcttcttcgcaaagactgcgtgtgctctttgttgagtgtgcgtaggatttacgacgtttactttgaaaaaattagagtgttcaaagcaggctattgcttgaatacatgagcatggaataatggaataggactttggtcccattttgttggtttctaggaccgaagtaatgattaagagggacaattgggggcatccgtatttcgttgtcagaggtgaaattcttggatttacgaaagacgaacaactgcgaaaagcatttgccaagagtgttttca
>70794019a05fbe3fbcd302d15567ede5\_2
agctccaatagcgtatattaaagttgttgcagttaaaaaagctcgtagttggatttcggagtgggccagttggtccgccgcaaggtgtgttactgactggtttgctcttcttcgcaaagactgcgtgtgctctttattgagtgtgcgtaggatttacgacgtttactttgaaaaaattagagtgttcaaagcaggctattgcttgaatacatgagcatggaataatggaataggattttggtcccattttgttggtttctaggaccgaagtaatgattaagagggacaattgggggcatccgtatttcgttgtcagaggtgaaattcttggatttacgaaagacgaacaactgcgaaagcacttgccaagagtagttttca
>71cdaec7698c2a6b3cc6cd731cb16f3f\_2
agctccaatagcgtatattaaagttgttgcagttaaaaaagctcgtagttggatttcggagtgggccagttggtccgccgcaaggtgtgttactgactggtttgctcttcttcgcaaagactgcgtgtgctctttattgagtgtgcgtaggatttacgacgttttactttgaaaaaattagagtgttcaaagcaggctattgcttgaatacatgagcatggaataatggaataggattttggtcccattttgttggtttctaggaccgaagtaatgattaagagggacaattgggggcatccgtatttcgttgtcagaggtgaaattcttggatttacgaaagacgaacaactgcgaaagcacttgccaagagtgtttca
>72664971c6fe716eb639ae10d2a1aee3\_2
agctccaatagcgtatattaaagttgttgcagttaaaaaagctcgtagttggatttcggagtgggccagttggtccgccgcaaggtgtgttactgactggtttgctcttcttcgcaaagactgcgtgtgctctttattgagtgtgcgtaggatttacgacgtttactttgaaaaaattagagtgttcaaagcaggctattgcttgaatacatgagcatggaataatggaataggattttggtcccattttgttggtttctaggaccgaagtaatgattaagagggacaattgggggcatccgtatttcgttgtcagaggtgaaattcttggatttacgaaagacgaacaactgcgaaagcacttgccaagagtgttttaca
>7376d259516fbfae767737d880f6170d\_2
agctccaatagcgtatattaaagttgttgcagttaaaaagctcgtagttggatttcggagtgggccagttggtccgccgcaaggtgtgttactgactggtttgctcttcttcgcaaagactgcgtgtgctctttgttgagtgtgcgtaggatttacgacgtttactttgaaaaaattagagtgttcaaagcaggctattgcttgaatacatgagcatggaataatggaataggactttggtcccattttgttggtttctaggaccgaagtaatgattaagagggacaattgggggcatccgtatttcgttgtcagaggtgaaattcttggatttacgaaagacgaacaactgcgaaagcatttgccaagagtgttttca
>73912b85b89a1f2b133853d38135e303\_2
agctccaatagcgtatattaaagttgttgcagttaaaaagctcgtagttggatttcggagtgggccagttggtccgccgcaaggtgtgttactgactggtctgctcttcttcgcaaagactgcgtgtgctctttgttgagtgtgcgtaggatttacgacgtttactttgaaaaaattgagtgttcaaagcaggctattgcttgaatacatgagcatggaataatggaataggactttggtcccattttgttggtttctaggaccgaagtaatgattaagagggacaattgggggcatccgtatttcgttgtcagaggtgaaattcttggatttacgaaagacgaacaactgacgaaagcatttgccaagagtgttttca
>74b0686ea8de6e9aed9a385a4b20217c\_2
agctccaatagcgtatattaaagttgttgcagttaaaaagctcgtagttggatttcggagtgggccagttggtccgccgcaaggtgtgttactgactggtttgctcttcttcgcaaagactgcgtgtgctctttattgagtgtgcgtaggatttacgacgtttactttgaaaaaattagagtgttcaaagcaggctattgcttgaatacatgagcatggaataatggaataggatttggtccattttgttggtttctaggaccgaagtaatgattaagagggacaattgggggcatccgtatttcgttgtcagaggtgaaattcttggatttacgaaagacgaacaactgcgaaagcacttgccaagagtgttttca
>75072f4c67c5ca8650c32335e02b2ad4\_2
agctccaatagcgtatattaaagttgttgcagttaaaaagctcgtagttggatttcggagtgggcccgttggtccgccgcaaggtgtgttactgactggtctgctcttcttcgcaaagactgcgtgtgctctttgttgagtgtgcgtaggatttacgacgtttactttgaaaaaattagagtgttcaaagcaggctattgcttgaatacatgagcatggaataatggaataggactttggtcccattttgttggtttctaggaccgaagtaatgattaagagggacaattgggggcatccgtatttcgttgtcagaggtgaaattctttggatttacgaaagacgaacaactgcgaagcacttgccaagagtgttttca
>75f7d1fe46a86cff0b53d8872c9c1b40\_2
agctccaatagcgtatattaaagttgttgcagttaaaaaagctcgtagttggatttcggagtgggcccgttggtccgccgcaaggtgtgttactgactggtctgctcttcttcgcaaagactgcgtgtgctctttgttgagtgtgcgtaggatttacgacgtttactttgaaaaaattagagtgttcaaagcaggctattgcttgaatacatgagcatggaataatggaataggactttggtcccattttgttggtttctaggaccgaagtaatgattaagagggacaattggggcatccgtatttcgttgtcagaggtgaaattcttggatttacgaaagacgaacaactgcgaaagcacttgccaagagtgtttca
>761bdba41993195c1af5373e4e8dc930\_2
agctccaatagcgtatattaaagttgttgcagttaaaaaagctcgtagttggatttcggagtgggccagttggtccgccgcaaggtgtgttactgactggtttgctcttcttcgcaaaagactgcgtgtgctctttattgagtgtgcgtaggatttacgacgtttactttgaaaaaattagagtgttcaaagcaggctattgcttgaatacatgagcatggaataatggaataggattttggtcccattttgttggtttctaggaccgaagtaatgattaagagggacaattgggggcatccgtatttcgttgtcagaggtgaaattcttggatttacgaaagacgaacaactgcgaaagcacttgccaagagtgttttca
>76d2484350a95dd1beb3b23fe5d9d55b\_2
agctccaatagcgtatattaaagttgttgcagttaaaaagactcgtagttggatttcggagtgggccagttggtccgccgcaaggtgtgttactgactggtttgctcttcttcgcaaagactgcgtgtgctctttattgagtgtgcgtaggatttacgacgtttactttgaaaaaaattagagtgttcaaagcaggctattgcttgaatacatgagcatggaataatggaataggattttggtcccattttgttggtttctaggaccgaagtaatgattaagagggacaattgggggcatccgtatttcgttgtcagaggtgaaattcttggatttacgaaagacgaacaactgcgaaagcacttgccaagagtgttttca
>7729a086595e4f5c75b1091313796168\_2
agctccaatagcatatattaaagttgttgcagttaaaaagctcgtagttggatttcggagtgggcccgttggtccgccgcaaggtgtgttactgactggtctgctcttcttcgcaaagactgcgtgtgctctttgttgagtgtgcgtaggatttacgacgtttactttgaaaaaattagagtgttcaaagcaggctattgcttgaatacatgagcatggaataatggaataggactttggtcccattttgttggtttctaggaccgaagtaatgattaagagggacaattgggggcatccgtatttcgttgtcagaggtgaaattcttggatttacgaaagacgaacaactgcgaaagcacttgccaagagtgttttca
>78dc5456507f1ce51eacae1d7c90783e\_2
agctccaatagcgtatattaaagttgttgcagttaaaaagctcgtagttggatttcggagtgggccagttggtccgccgcaaggtgtgttactgactggtctgctcttcttcgcaaagactgcgtgtgctctttgttgagtgtgcgtaggatttacgacgtttactttgaaaaaattagagtgttcaaagcaggctattgcttgaatacatgagcatggaataatggaataggactttggtcccattttgttggtttctaggaccgaagtaatgattaagagggacaattgggggcatccgtatttcgttggtcagaggtgaaattcttggatttacgaaagacgaacaactgcgaaagcatttgccaagagtgttttca
>7bfcdc0e5d74be47c4f2566f76477486\_2
agctccaatagcgtatattaaagttgttgcagttaaaaaagctcgtagttggatttcggagtgggccagttggtccgccgcaaggtgtgttactgactggtttgctcttcttcgcaaagactgcgtgtgctctttattgagtgtgcgtaggatttacgacgtttactttgaaaaaattagagtgttcaaagcaggctattgcttgaatacatgagcatggaataatggaataggattttggtcccattttgtttggtttctaggaccgaagtaatgattaagagggacaattgggggcatccgtatttcgttgtcagaggtgaaattcttggatttacgaaagacgaacaactgcgaaagcacttgccaagagtgttttca
>805c49e77a4fdd6f20a5898ce6d282a6\_2
agctccaatagcgtatattaaagttgttgcagttaaaaaagctcgtagttggatttcggagtgggccagttggtccgccgcaaggtgtgttactgactggtttgctcttcttcgcaaagactgcgtgtgctctttattgagtgtgcgtaggatttacgacgtttactttgaaaaaattagagtgttcaaagcaggctattgcttgaatacatgagcatggaataatggaataggattttggtcccattttgttggtttctaggaccgaagtaatgattaagagggacaattgggggcatccgtatttcgttgtcagaggtgaaattcttggatttacgaaagacgaacaactgcgaaagcacttgaccaagagtgttttca
>83961caadb79fd0cd90a3b3e37555a16\_2
agctccaatagcgtatattaaagttgttgcagttaaaaagctcgtagttggatttcggagtgggcccgttggtccgccgcaaggtgtgttactgactggtctgctcttcttcgcaaagactgcgtgtgctctttgttgagtgtgcgtaggatttacgacgtttactttgaaaaaattagagtgttcaaagcaggctattgcttgaatacatgagcatggaataatggaataggactttggtcccattttgttggtttctaggaccgaagtaatgattaagagggacaattgggggcatccgtatttcattgtcagaggtgaaattcttggatttacgaaagacgaacaactgcgaaagcacttgccaagagtgttttca
>874926cf4cb27f39a3787157bbe4c518\_2
agctccaatagcgtatattaaagttgttgcagttaaaaagctcgtagttggatttcggagtgggccagttggtccgccgcaaggtgtgttactgactggtctgctcttcttcgcaaagactgcgtgtgctctttgttgagtgtgcgtaggatttacgacgtttactttgaaaaaattagagtgttcaaagcaggctattgcttgaatacatgagcatggaataatggaataggactttggtcccattttgttggtttctaggaccgaagtaatgattaagagggacaattgggggcatccgtatttcgttgtcagaggttgaaattcttggatttacgaaagacgaacaactgcgaaagcacttgccaagagtgttttca
>89a716c3ebf41ceeb75d144265e1551c\_2
agctccaatagcgtatattaaagttgttgcagttaaaaagctcgtagttggatttcggagtgggcccgttggtccgccgcaaggtgtgttactgactggtctgctcttcttcgcaaagactgcgtgtgctctttgttgagtgtgcgtaggatttacgacgtttactttgaaaaaattgagtgttcaaagcaggctattgcttgaatacatgagcatggaataatggaataggactttggtcccattttgttggtttctaggaccgaagtaatgattaagagggacaattggggcatccgtatttcgttgtcagaggtgaaattcttggatttacgaaagacgaacaactgcgaaagcacttgccaagagtgttttca
>8a3e99053cf90b09b3e9986625277c93\_2
agctccaatagcgtatattaaagttgttgcagttaaaaagctcgtagttggatttcggagtgggccagttggtccgccgcaaggtgtgttactgactggtttgctcttcttcgcaaagactgcgtgtgctctttattgagtgtgcgtaggatttacgacgtttactttgaaaaaattgagtgttcaaagcaggctattgcttgaatacatgagcatggaataatggaataggattttggtcccattttgttggtttctaggtaccgaagtaatgattaagagggacaattgggggcatccgtatttcgttgtcagaggtgaaattcttggatttacgaaagacgaacaactgcgaaagcacttgccaagagtgttttca
>8a4b4084b722c5bd11601e541fee7d28\_2
agctccaatagcgtatattaaagttgttgcagttaaaaagctcgtagttggatttcggagtgggccagttggtccgccgcaaggtgtgttactgactggtttgctcttcttcgcaaagactgcgtgtgctctttattgagtgtgcgtaggatttacgacgtttactttgaaaaaattagagtgttcaaagcaggctattgcttgaatacatgagcatggaataatggaataggattttggtcccattttgttggtttctaggaccgaagtaatgattaagagggacaattgggggcatccgtatttcgttgtctagaggtgaaattcttggatttacgaaagacgaacaactgcgaaagcacttgccaagagtgttttca
>8b9bb3dc2ec01baaaa76e0bc741e8b04\_2
agctccaatagcgtatattaaagttgttgcagttaaaaagctcgtagttggatttcggagtgggcccgttggtccgccgcaaggtgtgttactgactggtctgctcttcttcgcaaagactgcgtgtgctctttgttgagtgtgcgtaggatttacgacgtttactttgaaaaaaattagagtgttcaaagcaggctattgcttgaatacatgagcatggaataatggaataggactttggtcccattttgttggtttctaggaccgaagtaatgattaagagggacaattgggggcatccgtatttcgttgtcagaggtgaaattctttggatttacgaaagacgaacaactgcgaaagcacttgccaagagtgtttttca
>8bcdff56ff9cb3a0743f81499db94a8f\_2
agctccaatagcgtatattaaagttgttgcagttaaaaagctcgtagttggatttcggagtgggcccgttggtccgccgcaaggtgtgttactgactggtctgctcttcttcgcaaagactgcgtgtgctctttgttgagtgtgcgtaggatttacgacgtttactttgaaaaaattagagtgttcaaagcaggctattgcttgaatacatgagcatggaataatggaataggactttggtcccattttgttggtttctaggaccgaagtaatgattaagagggacaattgggggcatccgtatttcgttgtcagaggtgaaattcttggatttacgaaagacgaacaactgcgaaagcacttgccaagagtgttttaca
>8c35d50fb59c3bf0218906f9321f5bff\_2
agctccaatagcgtatattaaagttgttgcagttaaaaagctcgtagttggatttcggagtgggccagttggtccgccgcaaggtgtgttactgactggtctgctcttcttcgcaaagactgcgtgtgctctttgttgagtgtgcgtaggatttacgacgtttactttgaaaaaattagagtgttcaaagcaggctattgcttgaatacatgagcatggaataatggaataggactttggtcccattttgttggtttctaggaccgaagtaatgattaagagggacaattggggcgatccgtatttcgttgtcagaggtgaaattcttggatttacgaaagacgaacaactgcgaaagcatttgccaagagtgttttca
>8c4921fbc26062aa1fe96166ca40b9af\_2
agctccaatagcgtatattaaagttgttgcagttaaaaagctcgtagttggatttcggagtgggccagttggtccgccgcaaggtgtgttactgactggtctgctcttcttcgcaaagactgcgtgtgctctttgttgagtgtgcgtaggatttacgacgtttactttgaaaaaattagagtgttcaaagcaggctattgcttgaatacatgagcatggaataatggaataggactttggtcccattttgttggtttctaggaccgaagtaatgattaagagggacaattgggggcatccgtatttcgttgtcagaggtgaaattcttggatttacgaaagacgaacaactgcgaaagcatttgccaagagtgtttcta
>8f4adf647a0b2551399d56007f0805be\_2
agctccaatagcgtatattaaagttgttgcagttaaaaagctcgtagttggatttcggagtgggcccgttggtccgccacaaggtgtgttactgactggtctgctcttcttcgcaaagactgcgtgtgctctttgttgagtgtgcgtaggatttacgacgtttactttgaaaaaattagagtgttcaaagcaggctattgcttgaatacatgagcatggaataatggaataggactttggtcccattttgttggtttctaggaccgaagtaatgattaagagggacaattgggggcatccgtatttcgttgtcagaggtgaaattcttggatttacgaaagacgaacaactgcgaaagcacttgccaagagtgttttca
>8fb145488438f54e659a5ab449f5a2aa\_2
agctccaatagcgtatattaaagttgttgcagttaaaaaagctcgtagttggatttcggagtgggcccgttggtccgccgcaaggtgtgttactgactggtctgctcttcttcgcaaagactgcgtgtgctctttgttgagtgtgcgtaggatttacgacgtttactttgaaaaaattgagtgttcaaagcaggctattgcttgaatacatgagcatggaataatggaataggactttggtcccattttgttggtttctaggaccgaagtaatgattaagagggacaattgggggcatccgtatttcgttgtcagaggtgaaattcttggatttacgaaagacgaacaactgcgaaagcacttgccaagagtgtttca
>912896e0590a1440cc2e169f31a34ff9\_2
agctccaatagcgtatattaaagttgttgcagttaaaaagctcgtagttggatttcggagtgggccagttggtccgccgcaaggtgtgttactgactggtctgctcttcttcgcaaagactgcgtgtgctctttgttgagtgtgcgtaggatttacgacgtttactttgaaaaaaattagagtgttcaaagcaggctattgcttgaatacatgagcatggaataatggaataggactttggtcccattttgttggtttctaggaccgaagtaatgattaagagggacaattgggggcatccgtatttcgttgtcagaggtgaaattcttggatttacgaaagacgaacaactgcgaaagcacttgccaagagtgtttca
>91a455c88b9f17057253957ba21fd176\_2
agctccaatagcgtatattaaagttgttgcagttaaaaagactcgtagttggatttcggagtgggccagttggtccgccgcaaggtgtgttactgactggtctgctcttcttcgcaaagactgcgtgtgctctttgttgagtgtgcgtaggatttacgacgtttactttgaaaaaattagagtgttcaaagcaggctattgcttgaatacatgagcatggaataatggaataggactttggtcccattttgttggtttctaggaccgaagtaatgattaagagggacaattgggggcatccgtatttcgttgtcagaggtgaaattcttggatttacgaaagacgaacaactgcgaaagcatttgccaagagtgttttca
>93c591b6ec8f5bae9538f9cf9609cf10\_2
agctccaatagcgtatattaaagttgttgcaagttaaaaagctcgtagttggatttcggagtgggccagttggtccgccgcaaggtgtgttactgactggtttgctcttcttcgcaaagactgcgtgtgctctttattgagtgtgcgtaggatttacgacgtttactttgaaaaaattagagtgttcaaagcaggctattgcttgaatacatgagcatggaataatggaataggattttggtcccattttgttggtttctaggaccgaagtaatgattaagagggacaattgggggcatccgtatttcgttgtcagaggtgaaattcttggatttacgaaagacgaacaactgcgaaagcacttgccaagagtgttttca
>9443a836dd398e897cdbac0bf9bd655e\_2
agctccaatagcgtatattaaagttgttgcagttaaaaaagctcgtagttggatttcggagtgggccagttggtccgccgcaaggtgtgttactgactggtctgctcttcttcgcaaagactgcgtgtgctctttgttgagtgtgcgtaggatttacgacgtttactttgaaaaaattgagtgttcaaagcaggctattgcttgaatacatgagcatggaataatggaataggactttggtcccattttgttggtttctaggaccgaagtaatgattaagagggacaattgggggcatccgtatttcgttgtcagaggtgaaattcttggatttacgaaagacgaacaactgcgaaagcatttgccaagagtgtttca
>94ab7912a02ed75e0da37437092e1452\_2
agctccaatagcgtatattaaagttgttgcagttaaaaaagctcgtagttggatttcggagtgggccagttggtccgccgcaaggtgtgttactgactggtttgctcttcttcgcaaagactgcgtgtgctctttattgagtgtgcgtaggatttacgacgtttactttgaaaaaattagagtgttcaaagcaggctattgcttgaatacatgagcatggaataatggaataggattttggtcccattttgttggtttctaggaccgaagtaatgattaagagggacaattggggcgatccgtatttcgttgtcagaggtgaaattcttggatttacgaaagacgaacaactgcgaaagcacttgccaagagtgttttca
>95ecc10015d474f3b0b739e301fe1758\_2
agctccaatagcgtatattaaagttgttgcagttaaaaagctcgtagttggatttcggagtgggccagttggtccgccgcaaggtgtgttactgactggtttgctcttcttcgcaaagactgcgtgtgctctttgttgagtgtgcgtaggatttacgacgtttactttgaaaaaattgagtgttcaaagcaggctattgcttgaatacatgagcatggaataatggaataggactttggtcccattttgttggtttctaggaccgaagtaatgattaagagggacaattgggggcatccgtatttcgttgtcagaggtgaaattcttggatttacgaaagacgaacaactgcgaaagcacttgccaagagtgttttca
>9665ab449e1de42df20d2c3f90e7dd82\_2
agctccaatagcgtatattaaagttgttgcagttaaaaagctcgtagttggatttcggagtgggccagttggtccgccgcaaggtgtgttattgactggtttgctcttcttcgcaaagactgcgtgtgctctttattgagtgtgcgtaggatttacgacgtttactttgaaaaaattagagtgttcaaagcaggctattgcttgaatacatgagcatggaataatggaataggattttggtcccattttgttggtttctaggaccgaagtaatgattaagagggacaattgggggcatccgtatttcgttgtcagaggtgaaattcttggatttacgaaagacgaacaactgcgaaagcacttgccaagagtgttttca
>987673035215d0a106906f0bbe6014d2\_2
tagctccaatagcgtatattaaagttgttgcagttaaaaagctcgtagttggatttcggagtgggcccgttggtccgccgcaaggtgtgttactgactggtctgctcttcttcgcaaagactgcgtgtgctctttgttgagtgtgcgtaggatttacgacgtttactttgaaaaaattagagtgttcaaagcaggctattgcttgaatacatgagcatggaataatggaataggactttggtcccattttgttggtttctaggaccgaagtaatgattaagagggacaattgggggcatccgtatttcgttgtcagaggtgaaattcttggatttacgaaagacgaacaactgcgaaagcacttgccaagagtgttttca
>988d88f7bf16ea32860b1894231ef422\_2
agctccaatagcgtatattaatgttgttgcagttaaaaaagctcgtagttggatttcggagtgggcccgttggtccgccgcaaggtgtgttactgactggtctgctcttcttcgcaaagactgcgtgtgctctttgttgagtgtgcgtaggatttacgacgtttactttgaaaaaaattagagtgttcaaagcaggctattgcttgaatacatgagcatggaataatggaataggactttggtcccattttgttggtttctaggaccgaagtaatgattaagagggacaattgggggcatccgtatttcgttgtcagaggtgaaattcttggatttacgaaagacgaacaactgcgaaagcacttgccaagagtgttttca
>9a10c5aaabbadd4210184a3b12848149\_2
agctccaatagcgtatattaaagttgttgtggttaaaaagctcgtagttggatttcggagtgggcccgttggtccgccgcaaggtgtgttactgactggtctgctcttcttcgcaaagactgcgtgtgctctttgttgagtgtgcgtaggatttacgacgtttactttgaaaaaattagagtgttcaaagcaggctattgcttgaatacatgagcatggaataatggaataggactttggtcccattttgttggtttctaggaccgaagtaatgattaagagggacaattgggggcatccgtatttcgttgtcagaggtgaaattcttggatttacgaaagacgaacaactgcgaaagcacttgccaagaatgttttca
>9a3fe4f0a039cc4377daf77e5de0c7da\_2
agctccaatagcgtatattaaagttgttgcagttaaaaagctcgtagttggatttcggagtgggcccgttggtccgccgcaaggtgtgttactgactggtctgctcttcttcgcaaagactgcgtgtgctctttgttgagtgtgcgtaggatttatgacgtttactttgaaaaaattagagtgttcaaagcaggctattgcttgaatacatgagcatggaataatggaataggactttggtcccattttgttggtttctaggaccgaagtaatgattaagagggacaattgggggcatccgtatttcgttgtcagaggtgaaattcttggatttacgaaagacgaacaactgcgaaagcacttgccaagagtgttttca
>9aa8c6e4b8f3aa7530f5fc2469422a6e\_2
agctccaatagcgtatattaaagttgttgcagttaaaaagctcgtagttggatttcggagtgggccagttggtccgccgcaaggtgtgttactgactggtttgctcttcttcacaaagactgcgtgtgctctttattgagtgtgcgtaggatttacgacgtttactttgaaaaaattagagtgttcaaagcaggctattgcttgaatacatgagcatggaataatggaataggattttggtcccattttgttggtttctaggaccgaagtaatgattaagagggacaattgggggcatccgtatttcgttgtcagaggtgaaattcttggatttacgaaagacgaacaactgcgaaagcacttgccaagagtgttttca
>9af21ac08469cebd087bc0c63a7fb078\_2
agctccaatagcgtatattaaagttgttgcagttaaaaagctcgtagttggatttcggagtgggccagttggtccgccgcaaggtgtgttactgactggtttgctcttcttcgcaaagactgcgtgtgctctttattgagtgtgcgtaggatttacgacgtttactttgaaaaaaattagagtgttcaaagcaggctattgcttgaatacatgagcatggaataatggaataggattttggtcccattttgttggtttctaggtaccgaagtaatgattaagagggacaattgggggcatccgtatttcgttgtcagaggtgaaattcttggatttacgaaagacgaacaactgcgaaagcacttgccaagagtgttttca
>9c472ae2d626f062b1bc59aa42d73129\_2
agctccaatagcgtatattaaagttgttgcagttaaaaagctcgtagttggatttcggagtgggcccgttggtccgccgcaaggtgtgttactgactggtctgctcttcttcgcaaagactgcgtgtgctctttgttgagtgtgcgtaggatttacgacgtttactttgaaaaaattagagtgttcaaagcaggctattgcttgaatacatgagcatggaataatggaataggactttggtcccattttgttggtttctaggaccgaagtaatgattaagagggacaattgggggcatccgtattcgttgtcagaggtgaaattcttggatttacgaaagacgaacaactgcgaaagcacttgccaagagtgtttttca
>9e0549ca0182e13ca7fd628edfc426c4\_2
agctccaatagcgtatattaaagttgttgcagttaaaaagctcgtagttggatttcggagtggccccgttggtccgccgcaaggtgtgttactgactggtctgctcttcttcgcaaagactgcgtgtgctctttgttgagtgtgcgtaggatttacgacgtttactttgaaaaaattagagtgttcaaagcaggctattgcttgaatacatgagcatggaataatggaataggactttggtcccattttgttggtttctaggaccgaagtaatgattaagagggacaattgggggcatccgtatttcgttgtcagaggtgaaattcttggatttacgaaagacgaacaactgcgaaagcacttgccaagagtgttttca
>9e97287964301153848a46085720228b\_2
agctccaatagcgtatattaaagttgttgcagttaaaaagctcgtagttggatttcggagtgggcccgttggtccgccgcaaggtgtgttactgactggtctgctcttcttcgcaaagactgcgtgtgctctttgttgagtgtgcgtaggatttacgacgtttactttgaaaaaattgagtgttcaaagcaggctattgcttgaatacatgagcatggaataatggaataggactttggtcccattttgttggtttctaggaccgaagtaatgattaagagggacaattgggggcatccgtatttcgttgtcaggaggtgaaatttcttggatttacgaaagacgaacaactgcgaaagcacttgccaagagtgttttca
>9f8e8ee5faeb1a5007ba8a4799a3f38f\_2
agctccaatagcgtatattaaagttgttgcagttaaaaagctcgtagttggatttcggagtgggccagttggtccgccgcaaggtgtgttactgactggtttgctcttcttcgcaaagactgcgtgtgctctttattgagtgtgcgtaggatttacgacgtttactttgaaaaaaattagagtgttcaaagcaggctattgcttgaatacatgagcatggaataatggaataggattttggtcccattttgttggtttctaggaccgaagtaatgattaagagggacaattggggcatccgtatttcgttgtcagaggtgaaattcttggatttacgaaagacgaacaactgcgaaagcacttgccaagagtgttttca
>a6306e48b1faf9bcf9ebfe96f1536efa\_2
agctccaatagcgtatattaaagttgttgcagttaaaaagctcgtagttggatttcggagtgggccagttggtccgccgcaaggtgtgttactgactggtttgctcttcttcgcaaagactgcgtgtgctctttattgagtgtgcgtaggatttacgacgtttactttgaaaaaattagagtgttcaaagcaggctattgcttgaatacatgagcatggaataatggaataggattttggtcccattttgttggtttctaggaccgaagtaatgattaagagggacaattgggggcatccgtatttcgttgtcagaggtgaaattcttggatttacgaaagacgaacaactgcgaaaagcacttgccaagagtgttttca
>a78450109135bfa45ccaec186cd98a01\_2
agctccaatagcgtatattaaagttgttgcagttaaaaagctcgtagttggatttcggagtgggccagttggtccgccgcaaggtgtgttactgactggtttgctcttcttcgcaaagactgcgtgtgctctttgttgagtgtgcgtaggatttacgacgtttactttgaaaaaattagagtgttcaaagcaggctattgcttgaatacatgagcatggaataatggaataggactttggtcccatttttgttggtttctaggaccgaagtaatgattaagagggacaattgggggcatccgtatttcgttgtcagaggtgaaattcttggatttacgaaagacgaacaactgcgaaagcacttgccaagagtgttttca
>aaec0da50c6192a9a6ec52f72fd2271f\_2
agctccaatagcgtatattaaagttgttgcagttaaaaagctcgtagttggatttcggagtgggccagttggtccgccgcaaggtgtgttactgactggtctgctcttcttcgcaaagactgcgtgtgctctttgttgagtgtgcgtaggatttacgacgtttactttgaaaaaattagagtgttcaaagcaggctattgcttgaatacatgagcatggaataatggaataggactttggtcccattttgttggtttctaggaccgaagtaatgattaagagggacaattgggggcatccgtatttcgttgtcagaggtgaaattcttggatttacgaaagacgaacaaactgcgaaagcatttgccaagagtgttttca
>ab78fe8c9677adaa28010f42cf2d5400\_2
agctccaatagcgtatattaaagttgttgcagttaaaaagctcgtagttggatttcggagtgggcccgttggtccgccgcaaggtgtgttactgactggtctgctcttcttcgcaaagactgcgtgtgctctttgttgagtgtgcgtaggatttacgacgtttactttgaaaaaaattagagtgttcaaagcaggctattgcttgaatacatgagcatggaataatggaataggactttggtcccattttgttggtttctaggaccgaagtaatgattaagagggacaattggggcatccgtatttcgttgtcagaggtgaaattcttggatttacgaaaagacgaacaactgcgaaaagcacttgccaagagtgttttca
>ac322ce7a2d75ad2128c562db0b3479f\_2
agctccaatagcgtatattaaagttgttgcagttaaaaaagctcgtagttggatttcggagtgggcccgttggtccgccgcaaggtgtgttactgactggtctgctcttcttcgcaaagactgcgtgtgctctttgttgagtgtgcgtaggatttacgacgttttactttgaaaaaattagagtgttcaaagcaggctattgcttgaatacatgagcatggaataatggaataggactttggtcccattttgttggtttctaggaccgaagtaatgattaagagggacaattgggggcatccgtatttcgttgtcagaggtgaaattcttggatttacgaaagacgaacaactgcgaaagcacttgccaagagtgttttca
>ace734a0f38e0e67886dd360aa386cfe\_2
agctccaatagcgtatattaaagttgttgcagttaaaaagctcgtagttggatttcggagtgggccagttggtccgccgcaaggtgtgttactgactggtctgctcttcttcgcaaagactgcgtgtgctctttgttgagtgtgcgtaggatttacgacgtttactttagaaaaaattagagtgttcaaagcaggctattgcttgaatacatgagcatggaataatggaataggactttggtcccattttgttggtttctaggaccgaagtaatgattaagagggacaattgggggcatccgtatttcgttgtcagaggtgaaattcttggatttacgaaagacgaacaactgcgaaagcatttgccaagagtgttttca
>ae13d4eebdca1e29c5d1d4d13d184c26\_2
agctccaatagcgtatattaaagttgttgcagttaaaaaagctcgtagttggatttcggagtgggccagttggtccgccgcaaggtgtgttactgactggtttgctcttcttcgcaaagactgcgtgtgctctttattgagtgtgcgtaggatttacgacgtttactttgaaaaaattagagtgttcaaagcaggctattgcttgaatacatgagcatggaataatggaataggattttggtcccattttgttggtttctaggaccgaagtaatgattaagagggacaattgggggcatccgtatttcgttgtcagaggtgaaattcttggatttacgaaagacgaacaactgcgaaagcaacttgccaagagtgttttca
>ae83cfbec941a267d20bda8bf2138311\_2
agctctaatagcgtatattaaagttgttgcagttaaaaagctcgtagttggatttcggagtgggcccgttggtccgccgcaaggtgtgttactgactggtctgctcttcttcgcaaagactgcgtgtgctctttgttgagtgtgcgtaggatttacgacgtttactttgaaaaaattagagtgttcaaagcaggctattgcttgaatacatgagcatggaataatggaataggactttggtcccattttgttggtttctaggaccgaagtaatgattaagagggacaattgggggcatccgtatttcgttgtcagaggtgaaattctttggatttacgaaagacgaacaactgcgaaagcacttgccaagagtgttttca
>aed413d355598a39cb8909330f26c703\_2
agctccaatagcgtatattaaagttgttgcagttaaaaagctcgtagttggatttcggagtggggccagttggtccgccgcaaggtgtgttactgactggtctgctcttcttcgcaaagactgcgtgtgctctttgttgagtgtgcgtaggatttacgacgtttactttgaaaaaattagagtgttcaaagcaggctattgcttgaatacatgagcatggaataatggaataggactttggtcccattttgttggtttctaggaccgaagtaatgattaagagggacaattgggggcatccgtatttcgttgtcagaggtgaaattcttggatttacgaaagacgaacaactgcgaaagcatttgccaagagtgttttca
>af3097c5bf75221dd0320734518a50d3\_2
agctccaatagcgtatattaaagttgttgcagttaaaaagctcgtagttggatttcggagtgggcccgttggtccgccgcaaggtgtgttactgactggtctgctcttcttcgcaaagactgcgtgtgctctttgttgagtgtgcgtaggatttacgacgtttactttgaaaaaattagagtgttcaaagcaggctattgcttgaatacatgagcatggaataatggaataggactttggtcccattttgttggtttctaggaccgaagtaatgattaagagggacaattgggggcatccgtatttcgttgtcaggaggtgaaattctttggatttacgaaagacgaacaactgcgaaagcacttgccaagagtgttttca
>af5231aa136ee413f814511591bd7d9c\_2
agctccaatagcgtatattaaagttgttgcagttaaaaaagctcgtagttggatttcggagtgggccagttggtccgccgcaaggtgtgttactggactggtttgctcttcttcgcaaagactgcgtgtgctctttattgagtgtgcgtaggatttacgacgtttactttgaaaaaattagagtgttcaaagcaggctattgcttgaatacatgagcatggaataatggaataggattttggtcccattttgttggtttctaggaccgaagtaatgattaagagggacaattgggggcatccgtatttcgttgtcagaggtgaaattcttggatttacgaaagacgaacaactgcgaaagcacttgccaagagtgttttca
>b0a521f7a0ac77efb4528688b5f6b9b4\_2
agctccaatagcgtatattaaagttgttgcagttaaaaaagctcgtagttggatttcggagtgggccagttggtccgccgcaaggtgtgttactgactggtttgctcttcttcgcaaagactgcgtgtgctctttattgagtgtgcgtaggatttacgacgtttactttgaaaaaattagagtgttcaaagcaggctattgcttgaatacatgagcatggaataatggaataggattttggtcccattttgttggtttctaggaccgaagtaatgattaagagggacaattgggggcatccgtatttcgttgtcagaggtgaaattcttggatttacgaaagacgaacaactgacgaaagcacttgccaagagtgttttca
>b131d1b14284d58139e093e499a6347e\_2
agctccaatagcgtatattaaagttgttgcagttaaaaaagctcgtagttggatttcggagtgggccagttggtccgccgcaaggtgtgttactgactggtttgctcttcttcgcaaagactgcgtgtgctctttattgagtgtgcgtaggatttacgacgtttactttgaaaaaattagagtgttcaaagcaggctattgcttgaatacatgagcatggaataatggaataggatttggtcccattttgttggtttctaggaccgaagtaatgattaagagggacaattgggggcatccgtatttcgttgtcagaggtgaaattcttggatttacgaaagacgaacaactgcgaaagcacttgccaagagtgtttca
>b17a094dde212c867a90d3288c4e09d5\_2
agctccaatagcgtatattaaagttgttgcagttaaaaagctcgtagttggatttcggagtgggccagttggtccgccgcaaggtgtgttactgactggtttgctcttcttcgcaaagactgcgtgtgctctttattgagtgtgcgtaggatttacgacgtttactttgaaaaaattgagtgttcaaagcaggctattgcttgaatacatgagcatggaataatggaataggattttggtcccattttgttggtttctaggaccgaagtaatgattaagagggacaattggggcatccgtatttcgttgtcagaggtgaaattcttggatttacgaaagacgaacaactgcgaaagcacttgccaagagtgttttca
>b375c77b85c1ee979b580f2de57ecee6\_2
agctccaatagcgtatattaaagttgttgcagttaaaaagctcgtagttggatttcggagtgggccagttggtccgccgcaaggtgtgttactgactggtttgctcttcttcgcaaagactgcgtgtgctctttattgagtgtgcgtaggatttacgacgtttactttgaaaaaattgagtgttcaaagcaggctattgcttgaatacatgagcatggaataatggaataggatttggtcccattttgttggtttctaggaccgaagtaatgattaagagggacaattgggggcatccgtatttcgttgtcagaggtgaaattcttggatttacgaaagacgaacaactgcgaaagcacttgccaagagtgttttca
>b46ad1e4db162be5a24d719da897b7aa\_2
agctccaatagcgttatattaaagttgttgcagttaaaaagctcgtagttggatttcggagtgggccagttggtccgccgcaaggtgtgttactgactggtctgctcttcttcgcaaagactgcgtgtgctctttgttgagtgtgcgtaggattttacgacgtttactttgaaaaaattagagtgttcaaagcaggctattgcttgaatacatgagcatggaataatggaataggactttggtcccattttgttggtttctaggaccgaagtaatgattaagagggacaattgggggcatccgtatttcgttgtcagaggtgaaattcttggatttacgaaagacgaacaactgcgaaaagcatttgccaagagtgttttca
>b6430103a0c092c65d32d911b2f153c6\_2
agctccaatagcgtatattaaagttgttgcagttaaaaagctcgtagttggatttcggagtgggccagttggtccgccgcaaggtgtgttactgactggtctgctcttcttcgcaaagactgcgtgtgctctttgttgagtgtgcgtaggatttacgacgtttactttgaaaaaattagagtgttcaaagcaggctattgcttgaatacatgagcatggaataatggaataggactttggtcccattttgttggtttctaggaccgaagtaatgattaagaggacaattgggggcatccgtatttcgttgtcagaggtgaaattcttggatttacgaaagacgaacaactgcgaaagcatttgccaagagtgtttca
>b65ed90520ad95fc964c914563e546ba\_2
agctccaatagcgtatattaaagttgttgcagttaaaaagctcgtagttggatttcggagtgggccagttggtccgccgcaaggtgtgttactgactggtctgctcttcttcgcaaagactgcgtgtgctctttgttgagtgtgcgtaggatttacgacgtttactttgaaaaaattagagtgttcaaagcaggctattgcttgaatacatgagcatggaataatggaataggactttggtcccattttgttggtttctaggaccgaagtaatgattaagagggacaattgggggcatccgtatttcgttgtcagaggtgaaattcttggatttacgaaagtacgaacaactgcgaaagcatttgccaagagtgttttca
>b8bb5897316146ffcf076361050ec60d\_2
agctccaatagcgtatattaaagttgttgcagttaaaaaagctcgtagttggatttcggagtgggcccgttggtccgccgcaaggtgtgttactgactggtctgctcttcttcgcaaagactgcgtgtgctctttgttgagtgtgcgtaggatttacgacgtttactttgaaaaaaattagagtgttcaaagcaggctattgcttgaatacatgagcatggaataatggaataggactttggtcccatttttgttggtttctaggaccgaagtaatgattaagagggacaattgggggcatccgtatttcgttgtcagaggtgaaattcttggatttacgaaagacgaacaactgcgaaagcacttgccaagagtgttttca
>bb730550cd1b9cb82f6d92b21495f223\_2
agctccaatagcgtatattaaagttgttgcagttaaaaagctcgtagttggatttcggagtgggccagttggtccgccgcaaggtgtgttactgactggtctgctcttcttcgcaaagactgcgtgtgctctttgttgagtgtgcgtaggatttacgacgtttactttgaaaaaattgagtgttcaaagcaggctattgcttgaatacatgagcatggaataatggaataggactttggtcccattttgttggtttctaggaccgaagtaatgattaagagggacaattgggggcatccgtatttcgtgtcagaggtgaaattcttggatttacgaaagacgaacaactgcgaaagcacttgccaagagtgttttca
>bcbf69e5d6a77dba6683248388367641\_2
agctccaatagcgtatattaaagttgttgcagttaaaaagctcgtagttggatttcggagtgggcccgttggtccgccgcaaggtgtgttactgactggtctgctcttcttcgcaaagactgcgtgtgctctttgttgagtgtgcgtaggatttacgacgtttactttgaaaaaattagagtgttcaaagcaggctattgcttgaatacatgagcatggaataatggaataggactttggtcccattttgttggtttctaggaccgaagtaatgattaagagggacaattgggggcatccgtatttcgttgtcagaggtgaaatttcttggatttacgaaagatgaacaactgcgaaagcacttgccaagagtgttttca
>bdb1e22da83287444f337b46ec1e630b\_2
agctccaatagcgtatattaaagttgttgcagttaaaaagctcgtagttggatttcggagtgggccagttggtccgccgcaaggtgtgttactgactggtttgctcttcttcgcaaagacagcgtgtgctctttattgagtgtgcgtaggatttacgacgtttactttgaaaaaattagagtgttcaaagcaggctattgcttgaatacatgagcatggaataatggaataggattttggtcccattttgttggtttctaggaccgaagtaatgattaagagggacaattgggggcatccgtatttcgttgtcagaggtgaaattcttggatttacgaaagacgaacaactgcgaaagcacttgccaagagtgttttca
>bfdbd7b1c95c74d7ab9ce70f34ab890d\_2
agctccaatagcgtatattaaagttgttgcagttaaaaagctcgtagttggatttcggagtgggccagttggtccgccgcaaggtgtgttactgactggtctgctcttcttcgcaaaagactgcgtgtgctctttgttgagtgtgcgtaggatttacgacgtttactttgaaaaaattagagtgttcaaagcaggctattgcttgaatacatgagcatggaataatggaataggactttggtcccattttgttggtttctaggaccgaagtaatgattaagagggacaattggggcatccgtatttcgttgtcagaggtgaaattcttggatttacgaaagacgaacaactgcgaaagcatttgccaagagtgttttca
>c08e29e9126093ae4c350ae8d957e1c4\_2
agctccaatagcgtatattaaagttgttgcagttaaaaagctcgtagttggatttcggagtgggccagttggtccgccgcaaggtgtgttactgactggtttgctcttcttcgcaaagactgcgtgtgctctttattgagtgtgcgtaggatttacgacgtttactttgaaaaaattgagtgttcaaagcaggctattgcttgaatacatgagcatggaataatggaataggattttggtcccattttgttggtttctaggaccgaagtaatgattaagagggacaattgggggcatccgtatttcgttgtcagaggtgaaattcttggatttacgaaagacgaacaactgcgaaagcacttgccaagagtgttttcat
>c18d57567d5ec6ffbdc9f55e20fa50df\_2
agctccaatagcgtatattaaagttgttgcagttaaaaagctcgtagttggatttcggagtgggccagttggtccgccgcaaggtgtgttactgactggtttgctcttcttcgcaaagactgcgtgtgctctttattgagtgtgcgtaggatttacgacgtttactttgaaaaaattagagtgttcaaagcaggctattgcttgaatacatgagcatggaataatggaataggactttggtcccattttgttggtttctaggaccgaagtaatgattaagagggacaattgggggcatccgtatttcgttgtcagaggtgaaattcttggatttacgaaagacgaacaactgcgaaagcacttgccaagagtgttttca
>c27dac6ce14b855c7aee55d67a788271\_2
agctccaatagcgtatattaaagttgttgcagttaaaagctcgtagttggatttcggagtgggccagttggtccgccgcaaggtgtgttactgactggtctgctcttcttcgcaaagactgcgtgtgctctttgttgagtgtgcgtaggatttacgacgtttactttgaaaaaattagagtgttcaaagcaggctattgcttgaatacatgagcatggaataatggaataggactttggtcccattttgttggtttctaggaccgaagtaatgattaagagggacaattgggggcatccgtatttcgttgtcagaggtgaaattcttggatttacgaaagacgaacaactgcgaaagcacttgccaagagtgttttca
>c3864314d5daa08f37e34091ccbe0054\_2
agctccaatagcgtatattaaagttgttgcagttaaaaagctcgtagttggatttcggagtgggccagttggtccgccgcaaggtgtgttactgactggtctgctcttcttcgcaaagactgcgtgtgctctttgttgagtgtgcgtaggatttacgacgtttactttgaaaaaattagagtgttcaaagcaggctattgcttgaatacatgagcatggaataatggaataggactttggtcccattttgttggtttctaggaccgaagtaatgattaagagggacaattggggggcatccgtatttcgttgtcagaggtgaaattcttggatttacgaaagacgaacaactgcgaaagcatttgccaagagtgttttca
>c3fb4b2cb0a0a9d83c773bdecd99f0ab\_2
agctccaatagcgtatattaaagttgttgcagttaaaaagctcgtagttggatttcggagtgggccagttggtccgccgcaaggtgtgttactgactggtctgctcttcttcgcaaagactgcgtgtgctctttgttgagtgtgcgtaggatttacgacgtttaactttgaaaaaattagagtgttcaaagcaggctattgcttgaatacatgagcatggaataatggaataggactttggtcccattttgttggtttctaggaccgaagtaatgattaagagggacaattgggggcatccgtatttcgttgtcagaggtgaaattcttggatttacgaaagacgaacaactgcgaaagcatttgccaagagtgttttca
>c7af752d9588b4d923a923dc334d7b19\_2
agctccaatagcgtatattaaagttgttgcagttaaaaagctcgtagttggatttcggagtgggcccgttggtccgccgcaaggtgtgttactgactggtctgctcttcttcgcaaagactgcgtgtgctctttgttgagtgtgcgtaggatttacgacgtttactttgaaaaaattagagtgttcaaagcaggctattgcttgaatacatgagcatggaataatggaataggactttggtcccattttgttggtttctaggaccgaagtaatgattaagagggacaattgggggcatccgtatttcgttgtcagaggtgaaattcttggatttacgaaagacgaacaactgcgaaaagcatttgccaagagtgttttca
>c89d70de1fd0195fc5229afee5cc9b5c\_2
agctccaatagcgtatattaaagttgttgcagttaaaaagctcgtagttggatttcggagtgggcccgttggtccgccgcaaggtgtgttactgactggtctgctcttcttcgcaaagactgcgtgtgctctttgttgagtgtgcgtaggatttacgacgtttactttgaaaaaattagagtgttcaaagcaggctattgcttgaatacatgagcatggaataatggaataggactttggtcccatttttgttggtttctaggaccgaagtaatgattaagagggacaattgggggcatccgtatttcgttgtcagaggtgaaattcttggatttacgaaagacgaacaactgcgaaagcatttgccaagagtgttttca
>c905ab7ac4e948bdead29e0b31248a5f\_2
agctccaatagcgtatattaaagttgttgcagttaaaaagctcgtagttggatttcggagtgggccagttggtccgccgcaaggtgtgttactgactggtttgctcttcttcgcaaagactgcgtgtgctctttattgagtgtgcgtaggatttacgacgtttactttgaaaaaaattagagtgttcaaagcaggctattgcttgaatacatgagcatggaataatggaataggatttttggtcccatttttgttggtttctaggaccgaagtaatgattaagagggacaattgggggcatccgtatttcgttgtcagaggtgaaattcttggatttacgaaagacgaacaactgcgaaagcacttgccaagagtgttttca
>c9522bd56872fabac4764dc4ea984c8f\_2
agctccaatagcgtatattaaagttgttgcagttaaaaagctcgtagtttggatttcggagtgggccagttggtccgccgcaaggtgtgttactgactggtctgctcttcttcgcaaagactgcgtgtgctctttgttgagtgtgcgtaggatttacgacgtttactttgaaaaaattagagtgttcaaagcaggctattgcttgaatacatgagcatggaataatggaataggactttggtcccattttgttggtttctaggaccgaagtaatgattaagagggacaattgggggcatccgtatttcgttgtcagaggtgaaattcttggatttacgaaagacgaacaactgcgaaagcatttgccaagagtgttttca
>ca234d034a04244f08eb9bb331ca8f8f\_2
agctccaatagcgtatattaaagttgttgcagttaaaaaagctcgtagttggatttcggagtgggcccgttggtccgccgcaaggtgtgttactgactggtctgctcttcttcgcaaagactgcgtgtgctctttgttgagtgtgcgtaggatttacgacgtttactttgaaaaaattagagtgttcaaagcaggctattgcttgaatacatgagcatggaataatggaataggactttggtcccattttgttggtttctaggaccgaagtaatgattaagagggacaattgggggcatccgtatttcgttgtcagaggtgaaattcttggatttacgaaagacgaacaactgcgaaagcacttgccaagagtgttttcat
>cba3ddecac579c9a30ad642f106297b7\_2
agctccaatagcgtatattaaagttgttgcggttaaaaagctcgtagttggatttcggagtgggcccgttggtccgccgcaaggtgtgttactgactggtctgctcttcttcgcaaagactgcgtgtgctctttgttgagtgtgcgtaggatttacgacgtttactttgaaaaaaattagagtgttcaaagcaggctattgcttgaatacatgagcatggaataatggaataggactttggtcccattttgttggtttctaggaccgaagtaatgattaagagggacaattgggggcatccgtatttcgttgtcagaggtgaaattcttggatttacgaaagacgaacaactgcgaaagcacttgccaagagtgttttca
>d1a4d089d54dfecedb1733baef3d5168\_2
agctccaatagcgtatattaaagttgttgcagttaaaaagctcgtagttggatttcggagtgggccagttggtccgccgcaaggtgtgttactgactggtttgctcttcttcgcaaagactgcgtgtgctctttattgagtgtgcgtaggatttacgacgtttactttgaaaaaattgagtgttcaaagcaggctattgcttgaatacatgagcatggaataatggaataggattttggtcccattttgttggttctaggaccgaagtaatgattaagagggacaattgggggcatccgtatttcgttgtcagaggtgaaattcttggatttacgaaagacgaacaactgcgaaagcacttgccaagagtgttttca
>d3186f901585b6451fa5b92f224ec2eb\_2
agctccaatagcgtatattaaagttgttgcagttaaaaagctcgtagttggatttcggagtgggccagttggtccgccgcaaggtgtgttactgactggtctgctcttcttcgcaaagactgcgtgtgctctttgttgagtgtgcgtaggatttacgacgtttactttgaaaaaaattagagtgttcaaagcaggctattgcttgaatacatgagcatggaataatggaataggactttggtcccattttgttggtttctaggaccgaagtaatgattaagagggacaattgggggcatccgtatttcgttgtcagaggtgaaattcttggatttacgaaagacgaacaactagcgaaagcatttgccaagagtgttttca
>d529c83fd63a5be3765c0cea1cf8da4a\_2
agctccaatagcgtatattaaagttgttgcagttaaaaagctcgtagttggatttcggagtgggccagttggtccgccgcaaggtgtgttactgactggtttgctcttcttcgcaaagactgcgtgtgctctttattgagtgtgcgtaggatttacgacgtttactttgaaaaaattgagtgttcaaagcaggctattgcttgaatacatgagcatggaataatggaataggattttggtcccattttgttggtttctaggaccgaagtaatgattaagagggacaattggggcatccgtatttcgttgtcagaggtgaaattcttggatttacgaaagacgaacaactgcgaaagcacttgccaagagtagttttca
>d5d48b3e6f46488457365bc1e86ebab1\_2
agctccaatagcgtatactaaagttgttgcagttaaaaagctcgtagttggatttcggagtgggcccgttggtccgccgcaaggtgtgttactgactggtctgctcttcttcgcaaagactgcgtgtgctctttgttgagtgtgcgtaggatttacgacgtttactttgaaaaaattagagtgttcaaagcaggctattgcttgaatacatgagcatggaataatggaataggactttggtcccattttgttggtttctaggaccgaagtaatgattaagagggacaattggggcatccgtatttcgttgtcagaggtgaaattcttggatttacgaaagacgaacaactgcgaaagcacttgccaagagtgttttca
>d60e1963fcb32b4059a780a0801bbc45\_2
agctccaatagcgtatattaaagttgttgcagttaaaaagctcgtagttggatttcggagtgggccagttggtccgccgcaaggtgtgttactgactggtttgctcttcttcgcaaagactgcgtgtgctctttgttgagtgtgcgtaggatttacgacgtttactttgaaaaaaattagagtgttcaaagcaggctattgcttgaatacatgagcatggaataatggaataggactttggtcccattttgttggtttctaggaccgaagtaatgattaagagggacaattgggggcatccgtatttcgttgtcagaggtgaaattcttggatttacgaaagacgaacaactgcgaaagcacttgccaagagtgtttca
>d6b35a657a36fa95c81c19ccdd498716\_2
agctccaatagcgtatattaaagttgttgcagttaaaaagctcgtagttggatttcggagtgggccagttggtccgccgcaaggtgtgttactgactggtctgctcttcttcgcaaagactgcgtgtgctctttgttgagtgtgcgtaggatttacgacgtttactttgaaaaaattagagtgttcaaagcaggctattgcttgaatacatgagcatggaataatggaataggactttggtcccattttgttggtttctaggaccgaagtaatgattaagagggacaattgggggcatccgtatttcgttgtcagaggtgaaattctggatttacgaaagacgaacaactgcgaaagcatttgccaagagtgttttca
>d6cb445fd1e2c1a411a36a67e70490a8\_2
agctccaatagcgtatattaaagttgttgcagttaaaaaagctcgtagttggatttcggagtgggccagttggtccgccgcaaggtgtgttactgactggtttgctcttcttcgcaaagactgcgtgtgctctttattgagtgtgcgtaggattttacgacgtttactttgaaaaaattagagtgttcaaagcaggctattgcttgaatacatgagcatggaataatggaataggattttggtcccattttgttggtttctaggaccgaagtaatgattaagagggacaattgggggcatccgtatttcgttgtcagaggtgaaattcttggatttacgaaagacgaacaactgcgaaagcacttgccaagagtgttttca
>d7489bbe5441c443a3c94cc3314517da\_2
agctccaatagcgtatattaaagttgttgcagttaaaaagctcgtagttggatttcggagtgggcccgttggtccgccgcaaggtgtgttactgactggtctgctcttcttcgcaaagactgcgtgtgctctttgttgagtgtgcgtaggatttacgacgtttactttgaaaaaattagagtgttcaaagcaggctattgcttgaatacatgagcatggaataatggaataggactttggtcccattttgttggtttctaggaccgaagtaatgattaagagggacaattggggcatccgtatttcgttgtcagaggtgaaattcttggatttacgaaagacgaacaactgcgaaagcacttgccaagagtgtttttca
>d77dd8a9bf0e1ba343e45def03095ca5\_2
agctccaatagcgtatattaaagttgttgcagttaaaaaagctcgtagttggatttcggagtgggccagttggtccgccgcaaggtgtgttactgactggtctgctcttcttcgcaaagactgcgtgtgctctttgttgagtgtgcgtaggatttacgacgtttactttgaaaaaattagagtgttcaaagcaggctattgcttgaatacatgagcatggaataatggaataggactttggtcccattttgttggtttctaggaccgaagtaatgattaagagggacaattggggcatccgtatttcgttgtcagaggtgaaattcttggatttacgaaagacgaacaactgcgaaagcatttgccaagagtgtttca
>d9911bbdcc4fa85e63bc2c430eeffe04\_2
agctccaatagcgtatattaaagttgttgcagttaaaaagctcgtagttggatttcggagtgggcccgttggtccgccgcaaggtgtgttactgactggtctgctcttcttcgcaaagactgcgtgtgctctttgttgagtgtgcgtaggatttacgacgtttactttgaaaaattagagtgttcaaagcaggctattgcttgaatacatgagcatggaataatggaataggactttggtcccattttgttggtttctaggaccgaagtaatgattaagagggacaattgggggcatccgtatttcgttgtcagaggtgaaattcttggatttacgaaagacgaacaactgcgaaagcacttgccaagagtgtttca
>da33a1a32e823ffe352b6e25d693cbf0\_2
agctccaatagcgtatattaaagttgttgcagttaaaaagctcgtagttggatttcggagtgggccagttggtccgccgcaaggtgtgttactgactggtctgctcttcttcgcaaagactgcgtgtgctctttgttgagtgtgcgtaggatttacgacgtttactttgaaaaaaattgagtgttcaaagcaggctattgcttgaatacatgagcatggaataatggaataggactttggtcccatttttgttggtttctaggaccgaagtaatgattaagagggacaattgggggcatccgtatttcgttgtcagaggtgaaattcttggatttacgaaagacgaacaactgcgaaagcatttgccaagagtgttttca
>da6f2a96fe15fb2ff10d442840ede77f\_2
agctccaatagcgtatattaaagttgttgcagttaaaaagctcgtagttggatttcggagtgggccagttggtccgccgcaaggtgtgttactgactggtttgctcttcttcgcaaagactgcgtgtgctctttattgagtgtgcgtaggatttacgacgtttactttgaaaaaaattagagtgttcaaagcaggctattgcttgaatacatgagcatggaataatggaataggattttggtcccattttgttggtttctaggaccgaagtaatgattaagagggacaattgggggcatccgtatttcgttgtcagaggtgaaattcttggatttacgaaagacgaacaactgcgaaagcacttgaccaagagtgttttca
>daf8cbbc5a15e12d768d59215e48fc9c\_2
agctccaatagcgtatattaaagttgttgcagttaaaaagctcgtagttggatttcggagtgggcccgttggtccgccgcaaggtgtgttactgactggtctgctcttcttcgcaaagactgcgtgtgctctttgttgagtgtgcgtaggatttacgacgtttactttgaaaaaattagagtgttcaaagcaggctattgcttgaatacatgagcatggaataatggaataggactttggtcccattttgttggtttctaggaccgaagtaatgattaagagggacaattgggggcatccgtatttcgtgtcagaggtgaaatttcttggatttacgaaagacgaacaactgcgaaagcacttgccaagagtgttttca
>db0f1d941873d252e77c7cfe4b9931ea\_2
agctccaatagcgtatattaaagttgttgcagttaaaaagctcgtagttggatttcggagtgggccagttggtccgccgcaaggtgtgttactgactggtttgctcttcttcgcaaagactgcgtgtgctctttattgagtgtgcgtaggatttacgacgtttactttgaaaaaattagagtgttacaaagcaggctattgcttgaatacatgagcatggaataatggaataggattttggtcccattttgttggtttctaggaccgaagtaatgattaagagggacaattgggggcatccgtatttcgttgtcagaggtgaaattcttggatttacgaaagacgaacaactgcgaaagcacttgccaagagtgttttca
>dd80997cba1c293b18afc182ac080098\_2
agctccaatagcgtatattaaagttgttgcagttaaaaagctcgtagttggatttcggagtgggcccgttggtccgccgcaaggtgtgttactgactggtctgctcttcttcgcaaagactgcgtgtgctctttgttgagtgtgcgtaggatttacgacgtttactttgaaaaaattagagtgttcaaagcaggctattgcttgaatacatgagcatggaataatggaataggactttggtcccattttgttggtttctaggaccgaagtaatgattaagagggacaattgggggcatccgtatttcgttgtcagaggtgaaattcttggatttacgaaagacgaacaactgcgaaagcaacttgccaagagtgttttca
>df9617f6255d84818c66819ed0f61184\_2
agctccaatagcgtatattaaagttgttgcagttaaaaagctcgtagttggatttcggagtgggccagttggtccgccgcaaggtgtgttactgactggtctgctcttcttcgcaaagactgcgtgtgctctttgttgagtgtgcgtaggatttacgacgtttactttgaaaaaattagagtgttcaaagcaggctattgcttgaatacatgagcatggaataatggaataggactttggtcccattttgttggtttctaggaccgaagtaatgattaagagggacaattgggggcatccgtatttcgttgtcagaggtgaaattcttggatttacgaaagacgaaacaactgcgaaagcatttgccaagagtgttttca
>e02c316d41a707eeb9885b0d2305f014\_2
agctccaatagcgtatattaaagttgttgcagttaaaaagctcgtagttggatttcggagtgggcccgttggtccgccgcaaggtgtgttactgactggtctgctcttcttcgcaaagactgcgtgtgctctttgttgagtgtgcgtaggatttacgacgtttactttgaaaaaattagagtgttcaaagcaggctattgcttgaatacatgagcatggaataatggaataggactttggtcccattttgttggtttcttaggaccgaagtaatgattaagagggacaattgggggcatccgtatttcgttgtcagaggtgaaattcttggatttacgaaagacgaacaactgcgaaagcacttgccaagagtgttttca
>e13c3db731ac74de26f670be31c746ed\_2
agctccaatagcgtatattaaagttgttgcagttaaaaagctcgtagttggatttcggagtgggccagttggtccgccgcaaggtgtgttactgactggtctgctcttcttcgcaaagactgcgtgtgctctttgttgagtgtgcgtaggatttacgacgtttactttgaaaaaattgagtgttcaaagcaggctattgcttgaatacatgagcatggaataatggaataggactttggtcccattttgtggtttctaggaccgaagtaatgattaagagggacaattgggggcatccgtatttcgttgtcagaggtgaaattcttggatttacgaaagacgaacaactgcgaaagcatttgccaagagtgttttca
>e1e60318b2d7defc6c35c49942ef1478\_2
agctccaatagcgtatactaaagttgttgcggttaaaaagctcgtagttggatttcggagtgggcccgttggtccgccgcaaggtgtgttactgactggtctgctcttcttcgcaaagactgcgtgtgctctttgttgagtgtgcgtaggatttacgacgtttactttgaaaaaaattagagtgttcaaagcaggctattgcttgaatacatgagcatggaataatggaataggactttggtcccattttgttggtttctaggaccgaagtaatgattaagagggacaattgggggcatccgtatttcgttgtcagaggtgaaattcttggatttacgaaagacgaacaactgcgaaagcacttgccaagagtgttttca
>e2e31aadc7a77285b46ab115382b7027\_2
agctccaatagcgtatattaaagttgttgcagttaaaaagctcgtagttggatttcggagtgggccagttggtccgccgcaaggtgtgttactgactggtctgctcttcttcgcaaagactgcgtgtgctctttgttgagtgtgcgtaggatttacgacgtttactttgaaaaaattagagtgttcaaagcaggctattgcttgaatacatgagcatggaataatggaataggactttggttccattttgttggtttctaggaccgaagtaatgattaagagggacaattgggggcatccgtatttcgttgtcagaggtgaaattcttggatttacgaaagacgaacaactgcgaaagcatttgccaagagtgttttca
>e3f074a8548dc210aface5de99b4d24e\_2
agctccaatagcgtatattaaagttgttgcagttaaaaagctcgtagttggatttcggagtgggccagttggtccgccgcaaggtgtgttactgactggtttgctcttcttcgcaaagactgcgtgtgctctttattgagtgtgcgtaggatttacgacgtttactttgaaaaaattagagtgttcaaagcaggctattgcttgaatacatgagcatggaataatggaataggattttggtcccattttgttggtttctaggaccgaagtaatgattaagagggacaattgggggcatccgtatttcgttgtcagaggtgaaattcttggatttacgaaagacgaacaactgcgaaagcacttgccaagagtgtttcta
>e48cbe6dd17a0c58115e07e9791ffd0a\_2
agctccaatagcgtatattaaagttgttgcagttaaaaagctcgtagttggatttcggagtgggcccgttggtccgccgcaaggtgtgttactgactggtctgctcttcttcgcaaagactgcgtgtgctctttgttgagtgtgcgtaggatttacgacgtttactttgaaaaaattagagtgttcaaagcaggctattgcttgaatacatgagcatggaataatggaataggactttggtcccattttgttggtttctaggaccgaagtaatgattaagagggacaattgggggcatccgtatttcgttgtcagagggtgaaattcttggatttacgaaagacgaacaactgcgaaagcacttgccaagagtgttttca
>e5937824f0305aaf9aa38537027a2e6e\_2
agctccaatagcgtatattaaagttgttgcagttaaaaagctcgtagttggatttcggagtgggccagttggtccgccgcaaggtgtgttactgactggtttgctcttcttcgcaaagactgcgtgtgctctttattgagtgtgcgtaggatttacgacgtttactttgaaaaaattagagtgttcaaagcaggctattgcttgaatacatgagcatggaataatggaataggattttggtcccattttgttggtttctaggaccgaagtaatgattaagagggacaattgggggcatccgtattcgttgtcagaggtgaaattcttggatttacgaaagacgaacaactgcgaaagcacttgccaagagtgtttca
>e68a99b008fcacf715e3434d7a3bbbc1\_2
agctccaatagcgtatattaaagttgttgcagttaaaaagctcgtagttggatttcggagtgggcccgttggtccgccgcaaggtgtgttactgactggtctgctcttcttcgcaaagactgcgtgtgctctttgttgagtgtgcgtaggatttacgacgtttactttgaaaaaattagagtgttcaaagcaggctattgcttgaatacatgagcatggaataatggaataggactttggtcccattttgttggtttctaggaccgaagtaatgattaagagggacaattgggggcatccgtatttcgttgtcagaggtgaaatttcttggatttacggaagacgaacaactgcgaaagcacttgccaagagtgttttca
>e73d998a1503f90c45d3fe080811721e\_2
agctccaatagcgtatattaaagttgttgcagttaaaaagctcgtagttggatttcggagtgggccagttggtccgccgcaaggtgtgttactgactggtctgctcttcttcgcaaagactgcgtgtgctctttgttgagtgtgcgtaggatttacgacgtttactttgaaaaaattagagtgttcaaagcaggctattgcttgaatacatgagcatggaataatggaataggactttggtcccattttgttggtttctaggaccgaagtaatgattaagagggacaagttgggggcatccgtatttcgttgtcagaggtgaaattcttggatttacgaaagacgaacaactgcgaaagcatttgccaagagtgttttca
>e8d2b98b67456111679216a2dc86ed72\_2
agctccaatagcgtatattaaagttgttgcagttaaaaagctcgtagttggagtttcggagtgggcccgttggtccgccgcaaggtgtgttactgactggtctgctcttcttcgcaaagactgcgtgtgctctttgttgagtgtgcgtaggatttacgacgtttactttgaaaaaattagagtgttcaaagcaggctattgcttgaatacatgagcatggaataatggaataggactttggtcccattttgttggtttctaggaccgaagtaatgattaagagggacaattgggggcatccgtatttcgttgtcagaggtgaaattcttggatttacgaaagacgaacaactgcgaaagcacttgccaagagtgttttca
>e9d9d90558f049e2989c8361d0e76db0\_2
agctccaatagcgtatattaaagttgttgcagttaaaaagctcgtagttggatttcggagtgggccagttggtccgccgcaaggtgtgttactgactggtctgctcttcttcgcaaagactgcgtgtgctctttgttgagtgtgcgtaggatttacgacgtttactttgaaaaaattagagtgttcaaagcaggctattgcttgaatacatgagcatggaataatggaataggactttggtcccatttttgttggtttctaggaccgaagtaatgattaagagggacaattgggggcatccgtatttcgttgtcagaggtgaaatttcttggatttacgaaagacgaacaactgcgaaagcatttgccaagagtgttttca
>ec34b1953021b227bf7dae6fd97a945f\_2
agctccaatagcgtatattaaagttgttgcagttaaaaagctcgtagttggatttcggagtgggccagttggtccgccgcaaggtgtgttactgactggtctgctcttcttcgcaaagactgcgtgtgctctttgttgagtgtgcgtaggatttacgacgtttactttgaaaaaattagagtgttcaaagcaggctattgcttgaatacatgagcatggaataatggaataggactttggtcccattttgttggtttctaggaccgaagtaatgattaagagggacaattgggggcatccgtatttcgttgtcagaggttgaaattcttggatttacgaaagacgaacaactgcgaaagcatttgccaagagtgttttca
>ec53b14afac74da6db6689fa47ee0b2f\_2
agctccaatagcgtatattaaagttgttgcggttaaaaagctcgtagttggatttcggagtgggccagttggtccgccgcaaggtgtgttactgactggtttgctcttcttcgcaaagactgcgtgtgctctttattgagtgtgcgtaggatttacgacgtttactttgaaaaaattagagtgttcaaagcaggctattgcttgaatacatgagcatggaataatggaataggattttggtcccattttgttggtttctaggaccgaagtaatgattaagagggacaattgggggcatccgtatttcgttgtcagaggtgaaattcttggatttacgaaagacgaacaactgcgaaagcacttgccaagagtgttttca
>ed549ba5572102b2d8e542c97199b001\_2
agctccaatagcgtatactaaagttgttgcggttaaaaagctcgtagttggatttcggagtgggccagttggtccgccgcaaggtgtgttactgactggtctgctcttcttcgcaaagactgcgtgtgctctttgttgagtgtgcgtaggatttacgacgtttactttgaaaaaaattagagtgttcaaagcaggctattgcttgaatacatgagcatggaataatggaataggactttggtcccattttgttggtttctaggaccgaagtaatgattaagagggacaattgggggcatccgtatttcgttgtcagaggtgaaattcttggatttacgaaagacgaacaactgcgaaagcatttgccaagagtgttttca
>edd3a32c7c30b137e713eeaf87632869\_2
agctccaatagcgtatattaaagttgttgcagttaaaaagctcgtagttggatttcggagtgggccagttggtccgccgcaaggtgtgttactgactggtttgctcttcttcgcaaagactgcgtgtgctctttattgagtgtgcgtaggatttacgacgtttactttgaaaaaattagagtgttcaaagcaggctattgcttgaatacatgagcatggaataatggaataggattttggtcccattttgttggtttctaggaccgaagtaatgattaagagggacaattgggggcatccgtatttcgttgttcagaggtgaaattcttggatttacgaaagacgaacaactgcgaaagcacttgccaagagtgtttca
>f11fb7890a488da7682d4ac17c46d3f0\_2
agctccaatagcgtatattaaagttgttgcagttaaaaagctcgtagttggatttcggagtgggcccgttggtccgccgcaaggtgtgttactgactggtctgctcttcttcgcaaagactgcgtgtgctctttgttgagtgtgcgtaggatttacgacgtttactttgaaaaaattagagtgttcaaagcaggctattgcttgaatacatgagcatggaataatggaataggactttggtcccattttgttggtttctaggaccgaagtaatgattaagagggacaattgggggcatccgtatttcgttgtcagaggtgaaattcttggatttacgaaaagacgaacaactgcgaaaagcacttgccaagagtgttttca
>f3512fa264ae06f72ee235e5877f545d\_2
agctccaatagcgtatactaaagttgttgcggttaaaaagctcgtagttggatttcggagtgggcccgttggtccgccgcaaggtgtgttactgactggtctgctcttcttcgcaaagactgcgtgtgctctttgttgagtgtgcgtaggatttacgacgtttactttgaaaaaattagagtgttcaaagcaggctattgcttgaatacatgagcatggaataatggaataggactttggtcccattttgttggtttctaggaccgaagtaatgattaagagggacaattggggcatccgtatttcgttgtcagaggtgaaattcttggatttacgaaagacgaacaactgcgaaagcacttgccaagagtgttttca
>f37221690349bb18214b916345ef3989\_2
agctccaatagcgtatattaaagttgttgcagttaaaaagctcgtagttggatttcggagtgggccagttggtccgccgcaaggtgtgttactgactggtctgctcttcttcgcaaagactgcgtgtgctctttgttgagtgtgcgtaggatttacgacgtttactttgaaaaaattagagtgttcaaagcaggctattgcttgaatacatgagcatggaataatggaataggactttggtcccattttgtggtttctaggaccgaagtaatgattaagagggacaattgggggcatccgtatttcgttgtcagaggtgaaattcttggatttacgaaagacgaacaactgcgaaagcatttgccaagagtgttttca
>f4b0e876ff692ed3e0cc0132e0024b5a\_2
agctccaatagcgtatattaaagttgttgcagttaaaaagctcggtagttggatttcggagtgggccagttggtccgccgcaaggtgtgttactgactggtctgctcttcttcgcaaagactgcgtgtgctctttgttgagtgtgcgtaggatttacgacgtttactttgaaaaaattagagtgttcaaagcaggctattgcttgaatacatgagcatggaataatggaataggactttggtcccattttgttggtttctaggaccgaagtaatgattaagagggacaattgggggcatccgtatttcgttgtcagaggtgaaattcttggatttacgaaagacgaacaactgcgaaaagcatttgccaagagtgttttca
>f5177b4bb9675d5fdde8397df00f3a23\_2
agctccaatagcgtatattaaagttgttgcagttaaaaagctcgtagttggatttcggagtgggccagttggtccgccgcaaggtgtgttactgactggtttgctcttcttcgcaaagactgcgtgtgctctttattgagtgtgcgtaggatttacgacgtttactttgaaaaaattagagtgttcaaagcaggctattgcttgaatacatgagcatggaataatggaataggattttggtcccattttgttggtttctaggaccgaagtaatgattaagaggacaattggggcatccgtatttcgttgtcagaggtgaaattcttggatttacgaaagacgaacaactgcgaaagcacttgccaagagtgttttca
>f72660b5edf981b81691c278f8905521\_2
agctccaatagcgtatattaaagttgttgcagttaaaaagctcgtagttggatttcggagtgggccagttggtccgccgcaaggtgtgttactgactggtttgctcttcttcgcaaagactgcgtgtgctctttattgagtgtgcgtaggatttacgacgtttactttgaaaaaaattgagtgttcaaagcaggctattgcttgaatacatgagcatggaataatggaatggattttggtcccattttgttggtttctaggaccgaagtaatgattaagagggacaattgggggcatccgtatttcgttgtcagaggtgaaattcttggatttacgaaagacgaacaactgcgaaagcacttgccaagagtgttttca
>f745c5c79574d2aa499d6646c94b9565\_2
agctccaatagcgtatattaaagttgttgcagttaaaaagctcgtagttggatttcggagtgggccagttggtccgccgcaaggtgtgttactgactggtctgctcttcttcgcaaagactgcgtgtgctctttgttgagtgtgcgtaggatttacgacgtttactttgaaaaaaattagagtgttcaaagcaggctattgcttgaatacatgagcatggaataatggaataggactttggtcccattttgttggtttctaggaccgaagtaatgattaagagggacaattgggggcatccgtatttcgtgtcagaggtgaaattcttggatttacgaaagacgaacaactgcgaaagcacttgccaagagtgttttca
>f74b2d8281ef716b4a8feb9d92e61d65\_2
agctccaatagcgtatattaaagttgttgcagttaaaaagctcgtagttggatttcggagtgggccagttggtccgccgcaaggtgtgttactgactggtctgctcttcttcgcaaagactgcgtgtgctctttgttgagtgtgcgtaggatttacgacgtttactttgaaaaaattagagtgttcaaagcaggctattgcttgaatacatgagcatggaataatggaataggactttggtcccattttgttggtttctaggaccgaagtaatgattaagagggacaattggggcatccgtattcgttgtcagaggtgaaattcttggatttacgaaagacgaacaactgcgaaagcatttgccaagagtgttttca
>f79a488d1e753760580023a38e925e96\_2
agctccaatagcgtatattaaagttgttgcagttaaaaagctcgtagttggatttcggagtgggcccgttggtccgccgcaaggtgtgttactgactggtctgctcttcttcgcaaagactgcgtgtgctctttgttgagtgtgcgtaggatttacgacgtttactttgaaaaaattagagtgttcaaagcaggctattgcttgaatacatgagcatggaataatggaataggactttggtcccattttgttggtttctaggaccgaagtaatgattaagagggacaattgggggcatccgtatttcgttggtcagaggtgaaattcttggatttacgaaagacgaacaactgcgaaagcacttgccaagagtgttttca
>f8cedd303b702d923262413eda451540\_2
agctccaatagcgtatattaaagttgttgcagttaaaaaagctcgtagttggatttcggagtgggccagttggtccgccgcaaggtgtgttactgactggtttgctcttcttcgcaaagactgcgtgtgctctttattgagtgtgcgtaggatttacgacgtttactttgaaaaaattagagtgttcaaagcaggctattgcttgaatacatgagcatggaataatggaataggattttggtcccatttttgttggtttctaggaccgaagtaatgattaagagggacaattgggggcatccgtatttcgttgtcagaggtgaaattcttggatttacgaaagacgaacaactgcgaaagcacttgccaagagtgttttca
>f96380171d4b8bba6e09b3371444768f\_2
agctccaatagcgtatattaaagttgttgcagttaaaaaagctcgtagttggatttcggagtgggccagttggtccgccgcaaggtgtgttactgactggtctgctcttcttcgcaaagactgcgtgtgctctttgttgagtgtgcgtaggatttacgacgtttactttgaaaaaaattagagtgttcaaagcaggctattgcttgaatacatgagcatggaataatggaataggactttggtcccattttgttggtttctaggaccgaagtaatgattaagagggacaattgggggcatccgtatttcgtgtcagaggtgaaattctggatttacgaaagacgaacaactgcgaaagcatttgccaagagtgttttca
>fbec450b095580d4caef4f5ec2c7b8e3\_2
agctccaatagcgtatattaaagttgttgcagttaaaaaagctcgtagttggatttcggagtgggccagttggtccgccgcaaggtgtgttactgactggtttgctcttcttcgcaaagactgcgtgtgctctttattgagtgtgcgtaggatttacgacgtttactttgaaaaaattagagtgttcaaagcaggctattgcttgaatacatgagcatggaataatggaataggattttggtcccattttgttggtttctaggaccgaagtaatgattaagagggacaattggggcatccgtatttcgttgtcagaggtgaaattcttggattttacgaaagacgaacaactgcgaaagcacttgccaagagtgttttca
>fc3fdf4a734a4ed805f09f4bc718bcf1\_2
agctccaatagcgtatattaaagttgttgcagttaaaaagctcgtagttggatttcggagtgggccagttggtccgccgcaaggtgtgttactgactggtctgctcttcttcgcaaagactgcgtgtgctctttgttgagtgtgcgtaggatttacgacgtttactttgaaaaaaattagagtgttcaaagcaggctattgcttgaatacatgagcatggaataatggaataggactttggtcccattttgttggtttctaggaccgaagtaatgattaagagggacaattgggggcatccgtatttcgtgtcagaggtgaaattctggatttacgaaagacgaacaactgcgaaagcatttgccaagagtgttttca
>fc98cc4ccd2d680804dfbb8099984b85\_2
agctccaatagcgtatattaaagttgttgcagttaaaaaagctcgtagttggatttcggagtgggccagttggtccgccgcaaggtgtgttactgactggtctgctcttcttcgcaaagactgcgtgtgctctttgttgagtgtgcgtaggatttacgacgtttactttgaaaaaaattgagtgttcaaagcaggctattgcttgaatacatgagcatggaataatggaataggactttggtcccattttgttggtttctaggaccgaagtaatgattaagagggacaattgggggcatccgtatttcgttgtcagaggtgaaattcttggatttacgaaagacgaacaactgcgaaagcatttgccaagagtgttttca
>fca2083ffda8d54f59fdb27deabb194e\_2
agctccaatagcgtatattaaagttgttgcagttaaaaaagctcgtagttggatttcggagtgggccagttggtccgccgcaaggtgtgttactgactggtttgctcttcttcgcaaagactgcgtgtgctctttattgagtgtgcgtaggatttacgacgttttactttgaaaaaaattagagtgttcaaagcaggctattgcttgaatacatgagcatggaataatggaataggattttggtcccattttgttggtttctaggaccgaagtaatgattaagagggacaattgggggcatccgtatttcgttgtcagaggtgaaattcttggatttacgaaagacgaacaactgcgaaagcacttgccaagagtgttttca
>fcc9da966e16daa203b8baafd482e41a\_2
agctccaatagcgtatattaaagttgttgcagttaaaaaagctcgtagttggatttcggagtgggccagttggtccgccgcaaggtgtgttactgactggtttgctcttcttcgcaaagactgcgtgtgctctttattgagtgtgcgtaggatttacgacgtttactttgaaaaaaattagagtgttcaaagcaggctattgcttgaatacatgagcatggaataatggaataggatttggtcccattttgttggtttctaggaccgaagtaatgattaagagggacaattgggggcatccgtatttcgttgtcagaggtgaaattcttggatttacgaaagacgaacaactgcgaaagcacttgccaagagtgttttca
>fd66f0e2864ac909ec78f09e9b56c785\_2
agctccaatagcgtatattaaagttgttgcagttaaaaagctcgtagttggatttcggagtgggccagttggtccgccgcaaggtgtgttactgactggtttgctcttcttcgcaaagactgcgtgtgctctttattgagtgtgcgtaggatttacgacgtttactttgaaaaaattagagtgttcaaagcaggctattgcttgaatacatgagcatggaataatggaataggattttggtcccattttgttggtttctaggaccgaagtaatgattaagagggacaattgggggcatccgtattcgttgtcagaggtgaaattcttggatttacgaaagacgaacaactgcgaaagcacttgccaagatgttttca
>fe5e2ba7b96e72f6cb6f0bb3d350a517\_2
agctccaatagcgtatattaaagttgttgcagttaaaaaagctcgtagttggatttcggagtgggcccgttggtccgccgcaaggtgtgttactgactggtctgctcttcttcgcaaagactgcgtgtgctctttgttgagtgtgcgtaggatttacgacgtttactttgaaaaaaattagagtgttcaaagcaggctattgcttgaatacatgagcatggaataatggaataggactttggtcccattttgttggtttctaggaccgaagtaatgattaagagggacaattgggggcatccgtatttcgttgtcagaggtgaaattcttggatttacgaaaagacgaacaactgcgaaagcacttgccaagagtgttttca
>fe683bb71c6251e888f917d5cc5e833a\_2
agctccaatagcgtatatttaaagttgttgcagttaaaaagctcgtagttggatttcggagtgggcccgttggtccgccgcaaggtgtgttactgactggtctgctcttcttcgcaaagactgcgtgtgctctttgttgagtgtgcgtaggatttacgacgtttactttgaaaaaattagagtgttcaaagcaggctattgcttgaatacatgagcatggaataatggaataggactttggtcccattttgttggtttctaggaccgaagtaatgattaagagggacaattgggggcatccgtatttcgttgtcagaggtgaaattcttggatttacgaaagacgaacaactgcgaaaagcacttgccaagagtgttttca
>fea23392ccf4245472709c7b89234ae8\_2
agctccaatagcgtatattaaagttgttgcagttaaaaagctcgtagttggatttcggagtggggcccgttggtccgccgcaaggtgtgttactgactggtctgctcttcttcgcaaagactgcgtgtgctctttgttgagtgtgcgtaggatttacgacgtttactttgaaaaaattagagtgttcaaagcaggctattgcttgaatacatgagcatggaataatggaataggactttggtcccattttgttggtttctaggaccgaagtaatgattaagagggacaattgggggcatccgtatttcgttgtcagaggtgaaattcttggatttacgaaagacgaacaactgcgaaagcacttgccaagagtgttttca
>ff034faaa2d2673ae4f1811bc04cf889\_2
agctccaatagcgtatattaaagttgttgcagttaaaaaagctcgtagttggatttcggagtgggccagttggtccgccgcaaggtgtgttactgactggtttgctcttcttcgcaaagactgcgtgtgctctttattgagtgtgcgtaggatttacgacgtttactttgaaaaaattagagtgttcaaagcaggctattgcttgaatacatgagcatggaataatggaataggattttggtcccattttgttggtttctaggaccgaagtaatgattaagagggacaattgggggcatccgtatttcgttgtcagaggtgaaattcttggatttacgaaagacgaacactgcgaaagcacttgccaagagtgttttca
>000ce0710dccc88fb2313a8494df1fd1\_1
agctccaatagcgtatattaaagttgttgcagttaaaaagctcgtagttggatttcggagtgggccagttggtccgccgcaaggtgtgttactgactggtttgctcttcttcgcaaagactgcgtgtgctctttattgagtgtgcgtaggatttacgacgtttactttgaaaaaattagagtgttcaaagcaggctattgcttgaatacatgagcatggaataatggaataggattttggtcccattttgttggtttctaggaccgaagtaatgattaagagggacaattgggggcatccgtatttcgttgtcagaggtgaaatttcttggatttacgaaagacgaacaactgcgaaagcacttgccaagagtgtttca
>00d1db192b433c32e796090e86e57d1e\_1
agctccaatagcgtatattaaagttgttgtggttaaaaagctcgtagttggatttcggagtgggcccgttggtccgccgcaaggtgtgttactgactggtctgctcttcttcgcaaagactgcgtgtgctctttgttgagtgtgcgtaggatttacgacgtttactttgaaaaaattagagtgctcaaagcaggctattgcttgaatacatgagcatggaataatggaataggactttggtcccattttgttggtttctaggaccgaagtaatgattaagagggacaattgggggcatccgtatttcgttgtcagaggtgaaattcttggatttacgaaagacgaacaactgcgaaagcatttgccaagagtgttttca
>01083741146b7fbb42c0e3be9bddf8cc\_1
agctccaatagcgtatattaaagttgttgcagttaaaaagctcgtagttggatttcggagtgggccagttggtccgccgcaaggtgtgttactgactggtttgctcttcttcgcaaagactgcgtgtgctctttattgagtgtgcgtaggatttacgacgtttactttgaaaaaattagagttttcaaagcaggctattgcttgaatacatgagcatggaataatggaataggattttggtcccattttgttggtttctaggaccgaagtaatgattaagagggacaattgggggcatccgtatttcgttgtcagaggtgaaattcttggatttacgaaagacgaacaactgcgaaagcacttgccaagagtgttttca
>014c3bf6798b6fb63179b6a741362d42\_1
agctccaatagcgtatattaaagttgttgcagtttaaaagctcgtagttggatttcggagtgggccagttggtccgccgcaaggtgtgttactgactggtttgctcttcttcgcaaagactgcgtgtgctctttgttgagtgtgcgtaggatttacgacgtttactttgaaaaaattagagtgttcaaagcaggctattgcttgaatacatgagcatggaataatggaataggactttggtcccattttgttggtttctaggaccgaagtaatgattaagagggacaattgggggcatccgtatttcgttgtcagaggtgaaattcttggatttacgaaagacgaacaactgcgaaagcacttgccaagagtgttttca
>01bc86c2981e246854450d697f5607b4\_1
agctccaatagcgtatattaaagttgttgcagttaaaaagctcgtagttggatttcggagtgggccagttggtccgccgcaaggtgtgttactgactggtctgctcttcttcgcaaagactgcgtgtgctctttgttgagtgtgcgtaggatttacgacgtttactttgaaaaaattagagtgttcaaagcaggctattgcttgaatacatgagcatggaataatggaataggactttggtcccattttgttggtttctaggaccgaagtaatgattaagagggacaattgggggcatccgtatttcgtagtcagaggtgaaattcttggatttacgaaagacgaacaactgcgaaagcatttgccaagagtgttttca
>02754055a00e8c7f0937db63db0fbbb6\_1
agctccaatagcgtatcttaatgttgttgcagttaaagctcgtagttggatttcggagtgggcccgttggtccgccgcaaggtgtgttactgactggtctgctcttcttcgcaaagactgcgtgtgctctttgttgagtgtgcgtaggatttacgacgtttactttgaaaaaattagagtgttcaaagcaggctattgcttgaatacatgagcatggaataatggaataggactttggtcccattttgttggtttctaggaccgaagtaatgattaagagggacaattgggggcatccgtatttcgttgtcagaggtgaaattcttggatttacgaaagacgaacaactgcgaaagcacttgccaagagtgttttca
>028f38eaf22cf619a024ccb07592b6bd\_1
agctccaatagcgtatattaaagttgttgcagttaaaaagctcgtagttggatttcggagtgggccagttggtccgccgcaaggtgtgttactgactggtttgctcttcttcgcaaagactgcgtgtgctctttattgagtgtgcgtaggatttacgacgtttactttgaaaaaaattagagtgttcaaagcaggctattgcttgaatacatgagcatggaataatggaataggattttggtcccattttgttggtttctaggaccgaagtaatgattaagagggacaattgggggcatccgtatttcgttgtcagaggtgaaattcttggatttacgaaagcgaacaactgcgaaagcacttgccaagagtgttttca
>0291a5e5ba95f71a433ae78af26981fc\_1
agctccaatagcgtatattaaagttgttgcagttaaaaagctcgtagttggatttcggagtgggcccgttggtccgccgcaaggtgtgttactgactggtctgctcttcttcgcaaagactgcgtgtgctctttgttgagtgtgcgtaggatttacgacgtttactttgaaaaaattgagtgttcaaagcaggctattgcttgaatacatgagcatggaataatggaataggactttggtcccattttgttggtttctaggaccgaagtaatgattaagagggacaattgggggcatccgtatttcgtgtcagaggtgaaatttcttggatttacgaaagacgaacaactgcgaaagcacttgccaagagtgttttca
>029d3e78dc1cb1a70491284ae94c36a6\_1
agctccaatagcgtatattaaagttgttgcagttaaaaaagctcgtagttggatttcggagtgggccagttggtccgccgcaaggtgtgttactggactggtttgctcttcttcgcaaagactgcgtgtgctctttattgagtgtgcgtaggatttacgacgtttactttgaaaaaattagagtgttcaaagcaggctattgcttgaatacatgagcatggaataatggaataggattttggtcccattttgttggtttctaggaccgaagtaatgattaagagggacaattgggggcatccgtatttcgttgtcagaggtgaaattcttggattttacgaaagacgaacaactgcgaaagcacttgccaagagtgttttca
>02fccf70834e1d84ffb111c97a25ac4e\_1
agctccaatagcgtatattaaagttgttgcagttaaaaagctcgtagttggatttcggagtgggccagttggtccgccgcaaggtgtgttactggactggtttgctcttcttcgcaaagactgcgtgtgctctttattgagtgtgcgtaggatttacgacgtttactttgaaaaaattagagtgttcaaagcaggctattgcttgaatacatgagcatggaataatggaataggattttggtcccattttgttggtttctaggaccgaagtaatgattaagagggacaattgggggcatccgtatttcgttgtcagaggtgaaattcttggatttacgaaagacgaacaactgcgaagcacttgccaagagtgttttca
>03130b89c4e51e440ddc2c437690760a\_1
agctccaatagcgtatattaaagttgttgcagttaaaaagctcgtagttggatttcggagtgggcccgttggtccgccgcaaggtgtgttactgactggtctgctcttcttcgcaaagactgcgtgtgctctttgttgagtgtgcgtaggatttacgacgtttactttgaaaaaattagagtgttcaaagcaggctattgcttgaatacatgagcatggaataatggaataggactttggtcccattttgttggtttctaggaccgaagtaatgactaagagggacaattgggggcatccgtatttcgttgtcagaggtgaaattcttggatttacgaaagacgaacaactgcgaaagcacttgccaagagtgttttca
>031bf2d5fa1e2cd32137f3dd6f039d11\_1
agctccaatagcgtatattaaagttgttgcagttaaaaagctcgtagttggatttcggagtgggccagttggtccgccgcaaggtgtgttactgactggtttgctcttcttcgcaaagactgcgtgtgctatttattgagtgtgcgtaggatttacgacgtttactttgaaaaaattagagtgttcaaagcaggctattgcttgaatacatgagcatggaataatggaataggattttggtcccattttgttggtttctaggaccgaagtaatgattaagagggacaattgggggcatccgtatttcgttgtcagaggtgaaattcttggatttacgaaagacgaacaactgcgaaagcacttgccaagagtgttttca
>03aefb2067f9d00fcd78d49a7515a4fa\_1
agctccaatagcgtatattaaagttgttgcagttaaaaagctcgtagttggatttcggagtgggccagttggtccgccgcaaggtgtgttactgactggtctgctcttcttcgcaaaagactgcgtgtgctctttgttgagtgtgcgtaggatttacgacgtttactttgaaaaaaattagagtgttcaaagcaggctattgcttgaaatacatgagcatggaataatggaataggactttggtcccattttgttggtttctaggaccgaagtaatgattaagagggacaattgggggcatccgtatttcgttgtcagaggtgaaattcttggatttacgaaagacgaacaactgcgaaagcatttgccaagagtgttttca
>05b2fa40cd6857412086161ee1c0f4d0\_1
agctccaatagcgtatattaaagttgttgcagttaaaaagctcgtagttggatttcggagtgggcccgttggtccgccgcaaggtgtgttactggactggtctgctcttcttcgcaaagactgcgtgtgctctttgttgagtgtgcgtaggatttacgacgtttactttgaaaaaattagagtgttcaaagcaggctattgcttgaatacatgagcatggaataatggaataggactttggtcccattttgttggtttctaggaccgaagtaatgattaagagggacaattgggggcatccgtatttcgttgtcagaggtgaaattcttggatttacgaaagacgaacaactgcgaaagcacttgccaagagtgttttca
>063366d9b42d97ea6dea0ba9692e5c77\_1
agctccaatagcgtatattaaagttgttgcagttaaaaagctcatagttggatttcggagtgggcccgttggtccgccgcaaggtgtgttactgactggtctgctcttcttcgcaaagactgcgtgtgctctttgttgagtgtgcgtaggatttacgacgtttactttgaaaaaattagagtgttcaaagcaggctattgcttgaatacatgagcatggaataatggaataggactttggtcccattttgttggtttctaggaccgaagtaatgattaagagggacaattgggggcatccgtatttcgttgtcagaggtgaaattcttggatttacgaaagacgaacaactgcgaaagcacttgccaagagtgttttca
>06469928d9de8920da98dd8c3dfdce37\_1
agctccaacagcgtatattaaagttgttgcagttaaaaagctcgtagttggatttcggagtgggcccgttggtccgccgcaaggtgtgttactgactggtctgctcttcttcgcaaagactgcgtgtgctctttgttgagtgtgcgtaggatttacgacgtttactttgaaaaaattagagtgttcaaagcaggctattgcttgaatacatgagcatggaataatggaataggactttggtcccattttgttggtttctaggaccgaagtaatgattaagagggacaattgggggcatccgtatttcgttgtcagaggtgaaattcttggatttacgaaagacgaacaactgcgaaagcacttgccaagagtgttttca
>074283d940df76c5ebcd677762e1de64\_1
agctccaatagcgtatattaaagttgttgcagttaaaaagctcgtagttggatttcggagtgggccagttggtccgccgcaaggtgtgttactgactggtctgctcttcttcgcaaagactgcgtgtgctctttgttgagtgtgcgtaggatttacgacgtttactttgaaaaaattagagtgttcaaagcaggctattgcttgaatacatgagcatggaataatggaataggactttggtcccattttgttggtttctaggaccgaagtaatgattaagagggacaattgggggcatccgatttcgttgtcagaggtgaaattcttggatttacgaaagacgaacaactgcgaaagcacttgccaagagtgttttca
>0777103e953ebe314e4ddd009f57e6d2\_1
agctccaatagcgtatattaaagttgttgcagttaaaagctcgtagttggatttcggagtgggcccgttggtccgccgcaaggtgtgttactgactggtctgctcttcttcgcaaagactgcgtgtgctctttgttgagtgtgcgtaggatttacgacgtttactttgaaaaaattagagtgttcaaagcaggctattgcttgaatacatgagcatggaataatggaataggactttggtcccattttgttggtttctaggaccgaagtaatgattaagagggacaattggggcatccgtatttcgttgtcagaggtgaaattcttggatttacgaaagacgaacaactgcgaaagcacttgccaagagtgttttca
>0867158afc4e1e7d9265dcb7cbbf80ba\_1
agctccaatagcgtatattaaagttgttgcagttaaaaaagctcgtagttggatttcggagtgggccagttggtccgccgcaaggtgtgttactgactggtttgctcttcttcgcaaagactgcgtgtgctctttattgagtgtgcgtaggatttacgacgtttactttgaaaaaattgagtgttcaaagcaggctattgcttgaatacatgagcatggaataatggaataggattttggtcccattttgttggtttctaggaccgaagtaatgattaagagggacaattgggggcatccggtatttcgttgtcagaggtgaaattcttggatttacgaaagacgaacaactgcgaaagcacttgccaagagtgttttca
>08bc30f39c42fa82b273bb19d664e8fc\_1
agctccaatagcgtatattaaagttgttgcagttaaaaagctcgtagttggatttcggagtgggccagttggtccgccgcaaggtgtgttactgactggtttgctcttcttcgcaaagactgcgtgtgctctttattgagtgtgcgtaggatttacgacgtttactttgaaaaaattagagtgttcaaagcaggctattgcttgaatacatgagcatggaataatggaataggatttggtcccatttgttggtttctaggaccgaagtaatgattaagagggacaattggggcatccgtatttcgttgtcagaggtgaaattcttggatttacgaaagacgaacaactgcgaaagcacttgccaagagtgttttca
>09bce4180abaa300f52f0adaa1156b01\_1
agctccaatagcgtatattaaagttgttgcagttaaaaagctcgtagttggatttcggagtgggcccgttggtccgccgcaaggtgtgttactgactggtctgctcttcttcgcaaagactgcgtgtgctctttgttgagtgtgcgtaggatttacgacgtttactttgaaaaaattagagtgttcaaagcaggctattgcttgaatacatgagcatggaataatggaataggactttggtcccattttgttggtttctaggaccgaagtaatgattaagagggacagttgggggcatccgtatttcgttgtcagaggtgaaattcttggatttacgaaagacgaacaactgcgaaagcatttgccaagagtgttttca
>09ca69406e934be5a124a612510d8f9e\_1
agctccaatagcgtatattaaagttgttgcagttaaaaagctcgtaagttggatttcggagtgggcccgttggtccgccgcaaggtgtgttactgactggtctgctcttcttcgcaaagactgcgtgtgctctttgttgagtgtgcgtaggatttacgacgtttactttgaaaaaattagagtgttcaaagcaggctattgcttgaatacatgagcatggaataatggaataggactttggtcccattttgttggtttctaggaccgaagtaatgattaagagggacaattgggggcatccgtatttcgttgtcagaggtgaaattcttggatttacgaaagacgaacaactgcgaaagcacttgccaagagtgttttca
>0a42594e391083e541562bbbc882bdad\_1
agctccaatagcgtatattaaagttgttgcagttaaaaagctcgtagttggatttcggagtgggcccgttggtccgccgcaaggtgtgttactgactggtctgctcttcttcgcaaagactgcgtgtgctctttgttgagtgtgcgtaggatttacgacgtttactttgaaaaaattagagtgttcaaagtaggctattgcttgaatacatgagcatggaataatggaataggactttggtcccattttgttggtttctaggaccgaagtaatgattaagagggacaattgggggcatccgtatttcgttgtcagaggtgaaattcttggatttacgaaagacgaacaactgcgaaagcacttgccaagagtgttttca
>0a7ffc249b05aaff269db51ce66cbced\_1
agctccaatagcgtatattaaagttgttgcagttaaaaagctcgtagttggatttcggagtgggcccgttggtccgccgcaaggtgtgttactgactggtctgctcttcttcgcaaagactgcgtgtgctctttgttgagtgtgcgtaggatttacgacgtttactttgaaaaaaattgagtgttcaaagcaggctattgcttgaatacatgagcatggaataatggaataggactttggtcccattttgttggtttctaggaccgaagtaatgattaagagggacaattgggggcatccgtattcgttgtcagaggtgaaattcttggatttacgaaagacgaacaactgcgaaagcacttgccaagagtgttttca
>0b4a45e2c1d406c2279eeb90e035bc95\_1
agctccaatagcgtatattaaagttgttgcagttaaaaagctcgtagttggatttcggagtgggcccgttggtccgccgcaaggtgtgttactgactggtctgctcttcttcgcaaagactgcgtgtgctctttgttgagtgtgcgtaggatttacgacgtttactttgaaaaaattagagtgttcaaagcaggctattgcttgaatacatgagcatggaataatggaataggactttggtcccattttgttggtttctagggccgaagtaatgattaagagggacaattggggcatccgtatttcgttgtcagaggtgaaattcttggatttacgaaagacgaacaactgcgaaagcacttgccaagagtgttttca
>0b7be9e12a92b5a525915f08b570f336\_1
agctccaatagcgtatattaaagttgttgcagttaaaaaagctcgtagttggatttcggagtgggccagttggtccgccgcaaggtgtgttactgactggtttgctcttcttcgcaaagactgcgtgtgctctttattgagtgtgcgtaggatttacgacgtttactttgaaaaaattagagtgttcaaagcaggctattgcttgaatacatgagcatggaataatggaataggattttggtcccattttgttggtttctaggaccgaagtaatgattaagaggacaattggggcatccgtatttcgttgtcagaggtgaaattcttggatttacgaaagacgaacaactgcgaaagcacttgccaagagtgttttca
>0bd057e97d205efc80e51d812881c50b\_1
agctccaatggcgtatattaaagttgttgcagttaaaaagctcgtagttggatttcggagtgggcccgttggtccgccgcaaggtgtgttactgactggtctgctcttcttcgcaaagactgcgtgtgctctttgttgagtgtgcgtaggatttacgacgtttactttgaaaaaattagagtgttcaaagcaggctattgcttgaatacatgagcatggaataatggaataggactttggtcccattttgttggtttctaggaccgaagtaatgattaagagggacaattgggggcatccgtatttcgttgtcagaggtgaaatttcttggatttacgaaagacgaacaactgcgaaagcacttgccaagagtgttttca
>0c0398becf61c0702184034a57eb5062\_1
agctccaatagcgtatatttaagttgttgcagttaaaaagctcgtagttggatttcggagtgggcccgttggtccgccgcaaggtgtgttactgactggtctgctcttcttcgcaaagactgcgtgtgctctttgttgagtgtgcgtaggatttacgacgtttactttgaaaaaattagagtgttcaaagcaggctattgcttgaatacatgagcatggaataatggaataggactttggtcccattttgttggtttctaggaccgaagtaatgattaagagggacaattgggggcatccgtatttcgttgtcagaggtgaaattcttggatttacgaaagacgaacaactgcgaaagcacttgccaagagtgttttca
>0c11caca11d4144c9c4357f8fe412f62\_1
agctccaatagcgtatattaaagttgttgcagtttaaaaagctcgtagttggatttcggagtgggccagttggtccgccgcaaggtgtgttactgactggtttgctcttcttcgcaaagactgcgtgtgctctttgttgagtgtgcgtaggatttacgacgtttactttgaaaaaattagagtgttcaaagcaggctattgcttgaatacatgagcatggaataatggaataggactttggtcccattttgttggtttctaggaccgaagtaatgattaagagggacaattgggggcatccgtatttcgttgtcagaggtgaaattcttggatttacgaaagacgaacaactgcgaaagcacttgccaagagtgttttca
>0c21b7c8b47b6c2f702aafeb94e6ad3f\_1
agctccaatagcgtatattaaagttgttgcagttaaaaaagctcgtagttggatttcggagtgggccagttggtccgccgcaaggtgtgttactgactggtttgctcttcttcgcaaagactgcgtgtgctctttattgagtgtgcgtaggatttacgacgtttactttgaaaaaattagagtgttcaaagcaggctattgcttgaatacatgagcatggaataatggaataggattttggtcccattttgttggtttctaggaccgaagtaatgattgagagggacaattgggggcatccgtatttcgttgtcagaggtgaaattcttggatttacgaaagacgaacaactgcgaaagcacttgccaagagtgtttca
>0d143f270e9c6c6417584be3b04a89d4\_1
agctccaatagcgtatattaaagttgttgcagttaaaaagctcgtagttggatttcggagtgggccagttggtccgccgcaaggtgtgttactgactggtctgctcttcttcgcaaagactgcgtgtgctctttgttgagtgtgcgtaggatttacgacgtttactttgaaaaaattagagtgttcaaagcaggctattgcttgaatacatgagcatggaataatggaataggactttggtcccattttgttggtttctaggaccgaagtaatgattaagagggacaattgggggcatccgtatttcgttgtcagaggtgaaattcttggatttacgtaaagacgaacaactgcgaaaagcatttgccaagagtgttttca
>0d1c8cdf0fb12d5d81c9251d074e27da\_1
agctccaatagcgtatattaaagttgttgcagttaaaaagctcgtagttggatttcggagtgggccagttggtccgccgcaaggtgtgttactgactggtttgctcttcttcgcaaagactgcgtgtgctctttgttgagtgtgcgtaggatttacgacgtttactttgaaaaaattagagtgttcaaagcaggctattgcttgaatacatgagcatggaataatggaataggactttggtcccattttgttggtttctaggaccgaagtaatgattaagagggacaattgggggcatccgtatttcgttgtcagaggtggaaattcttggatttacgaaagacgaacaactgcgaaagcacttgccaagagtgttttca
>0d9a022046fdc28775cb71543fb98e01\_1
[truncated: 173,574 more chars]
